# The critical helping hand of water: theory shows the way to obtain elusive, granular information about kinetic asymmetry driven systems†

**DOI:** 10.1039/d5sc03256c

**Published:** 2025-07-21

**Authors:** Priyam Bajpai, Shrivatsa Thulasiram, Kumar Vanka

**Affiliations:** a Physical and Materials Chemistry Division, CSIR-National Chemical Laboratory Pune-411008 India k.vanka@ncl.res.in; b Academy of Scientific and Innovative Research (AcSIR) Ghaziabad-201002 India; c Indian Institute of Science Education and Research Pune-411008 India

## Abstract

Kinetic asymmetry is crucial in chemical systems where the selective synthesis of one product over another, or the acceleration of specific reaction(s) is necessary. However, obtaining precise information with current experimental methods about the behavior of such systems as a function of time, substrate concentration and other relevant factors, is not possible. Computational chemistry provides a powerful means to address this problem. The current study unveils a two-pronged computational approach: (i) full quantum chemical studies with density functional theory (DFT), followed by (ii) stochastic simulations with a validated Gillespie algorithm (GA) (using representative model systems where necessary), to study the behavior of a kinetic asymmetry driven unidirectional molecular motor (1-phenylpyrrole2,2′-dicarboxylic acid) (*Nature*, 2022, **604** (7904), 80–85). Our approach allows us to understand what is really taking place in the system, underlining the crucial role played by water molecules in facilitating the rotation of the motor. It is seen that water lubricates the motion by increasing the rotation rate constant of the final step by, remarkably, more than ten orders of magnitude! These insights further serve to explain the efficient rotation of the very recently reported gel-embedded molecular motor (*Nature*, 2025, **637** (8046), 594–600), providing an upper limit for the allowed rotation barrier in such systems, and thus also casts light into the functioning of bio-molecular motors. The current work therefore provides a template for carefully and properly studying a wide variety of important, kinetic asymmetry driven systems in the future.

## Abstract 2

Through computational studies, we show that powerful hidden factors, such as the water effect on the rotation of a molecular motor, can be captured. Our approach can be employed to understand a wide range of kinetic asymmetry driven systems.

## Graphical abstract

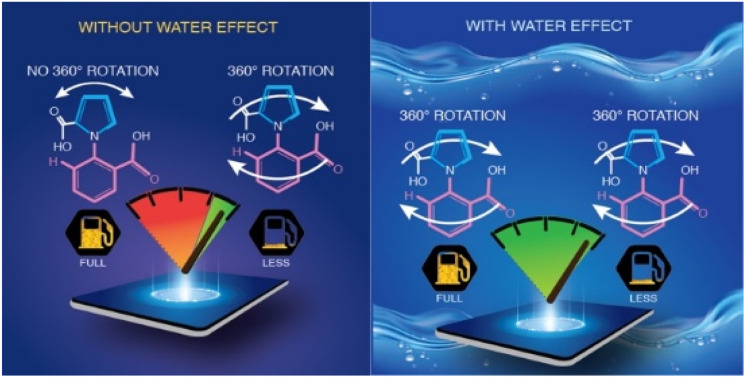

## Introduction

One of the most important developments in recent years has been the recognition of the power of kinetic asymmetry to produce important results in different areas of chemistry. Kinetic asymmetry^1^ refers to the unequal reactivity or rate of progress of different reaction pathways in a one-pot chemical system, often due to the influence of a catalyst, or the chirality of the structure of the reacting molecules, or of external conditions. Kinetic asymmetry has found application in various fields in chemistry, of nonequilibrium chemical science,^2^ including photovoltaics,^3^ artificial photosynthesis,^4^ catalysis,^5^ asymmetric synthesis,^6^ deracemization processes,^7^ chemical reaction networks (systems chemistry),^8^ chemical oscillators,^9^ electric field driven catalysis^10^ and the origin of life.^11^ In more complex systems, kinetic asymmetry was seen to play a crucial role in non-equilibrium thermodynamics,^12^ the study of dissipative systems,^13^ and in the field of molecular motors.^14^

However, it is also worth noting that the application of kinetic asymmetry is still in its infancy, and that there are enormous possibilities of exploiting its potential in the future. What is necessary for this to happen is to thoroughly understand how it operates, and what the natural constraints and limitations are to its applications across the chemical space. The most obvious constraint is due to the fact that the bias of one process over another in a one pot system of competing processes is often not considerable, and the quantification of the degree of advantage that kinetic asymmetry offers in any given chemical step is very important, if one is to properly understand its influence. Such quantification requires a comprehensive and granular understanding of each step, and unfortunately, this is beyond the existing experimental methods that are available today. Alternatively, one could consider computational approaches such as *ab initio* molecular dynamics simulations, but they are too expensive and time consuming to envisage at the current moment.

What, then, is the solution? We contend that the answer lies in careful computational investigations that are able to (i) first obtain proper information about the chemical reactions, as well as the potential energy surfaces, through full quantum chemical studies with state-of-the-art methods such as density functional theory (DFT) and (ii) exploit the information thus gained to obtain an understanding of the dynamics of the processes taking place through further studies. These can take the form of stochastic simulations with well-established methods such as the exact Gillespie algorithm.^15^ It is this two-pronged computational approach that can provide information and insights crucial to fully understanding how kinetic asymmetry operates in chemical systems, thereby opening the portal to their systematic improvement.

**Fig. 1:**
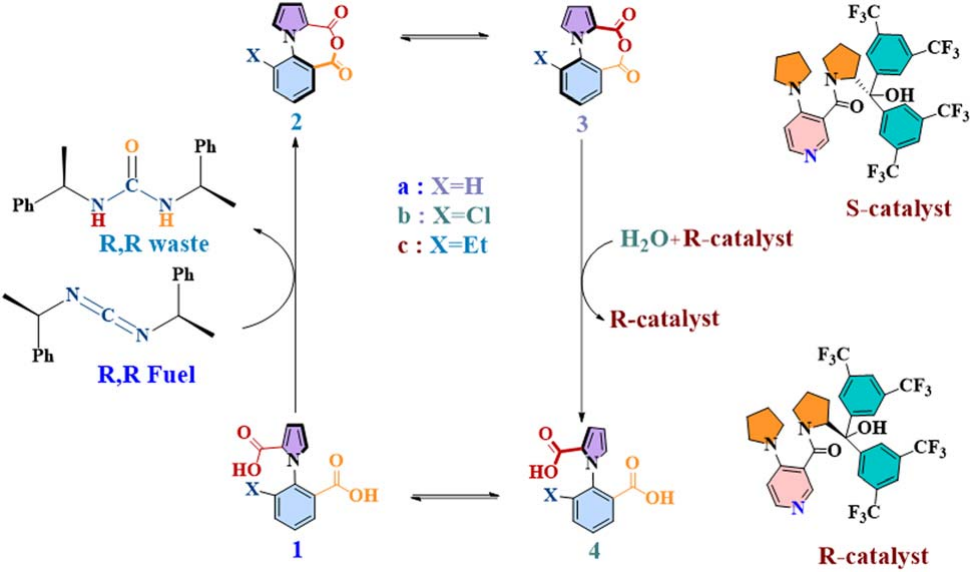
The chemical cycle of a continuously operating, chemically fueled 360° rotary motor (1a) in forward direction.

This is the objective that has shaped the current work, where we have focused on one of the most important examples of kinetic asymmetry that have been demonstrated in recent years: a unidirectional molecular motor, reported in 2022 by David Leigh and co-workers.^16^ 360° autonomous unidirectional rotation of a pyrrole rotor takes place around a benzene stator in 1-phenylpyrrole2,2-dicarboxylic acid. The system exploits the principles of kinetic asymmetry *via* an “information ratchet”, coupling two steps of dynamic stochastic processes with two other steps where chemical energy is harnessed by the rotor. This is illustrated in Fig. 1 below. There are two atropisomeric conformations, 1a and 4a, which react at different rates with a chiral carbodiimide “fuel” molecule, thereby creating kinetic asymmetry and driving the reaction cycle clockwise towards the anhydrous species 2a, in equilibrium, with its atropisomeric conformer 3a. The second kinetic gating step occurs here, with a chiral catalyst preferentially hydrating one atropisomeric conformer (3a) over another (2a), and giving rise to 4a. At this stage of the cycle, instead of reacting with the carbodiimide fuel, 4a can rotate along the C–N bond axis to yield the original 1a atropisomer conformer, thus completing 360° rotation. The rotor–stator molecule would be expected to complete directed 360° rotations indefinitely, as long as fuel lasted in the system.

One notes, upon studying the system, that the steps where kinetic asymmetry is introduced: 1a–2a and 3a–4a, are competitive with their reversible counterparts. This is also evidenced from the fact that the authors report that out of every four turns, it is possible that there is one “wrong” turn, *i.e.*, the motor could rotate in the undesired reverse direction. Notably, this information was obtained not by studying the actual molecular machine but from an analogue (with ethyl at the ortho position of the benzene ring). In other words, the degree of undesired rotational behavior in the actual motor is unknown. Furthermore, the fact that the molecular machine completes 360° rotation is also based on indirect evidence – by studying its chloride analogue. This is not to downplay the importance of this development: this is a remarkable motor molecule, consisting of only 17 non-hydrogen atoms, which displays directionality in a set of one-pot reactions: a towering accomplishment. Furthermore, the fact that this concept has great potential for transduction applications is evidenced by the very recent report with this motor by Leigh, Giuseppone and coworkers, where they demonstrate that the motor can be modified to coordinate to a crosslinked polymer gel, and then power the contraction and re-expansion of the gel through directional rotation.^17^ Additionally, Leigh and coworkers have also reported optimization of the chiral fuel and chiral catalyst in order to increase the efficiency of the unidirectional rotation.^18^ A further work has just appeared, showing the rotational behavior of a two rotor variant of this motor.^19^ Nevertheless, it is clear that a precise understanding of the operation of the molecular machine is still elusive.

Moreover, while the kinetic gating steps are elegant, it is also important to understand quantitatively the role that water may be playing in this system. This is because, as will be explained in Section (iii) in the results and discussion, the experimental results obtained with the chloride analogue of the motor indicate to us that there is a clear “lubricating” effect of water on the behavior of the motor. Indeed, previous reports have highlighted the fact that explicit water molecules can assist in the kinetics of motor behavior if hydrogen bonding groups are present in the motor,^20,21^ but quantifying the water effect is extremely difficult from quantum chemical studies.

**Fig. 2:**
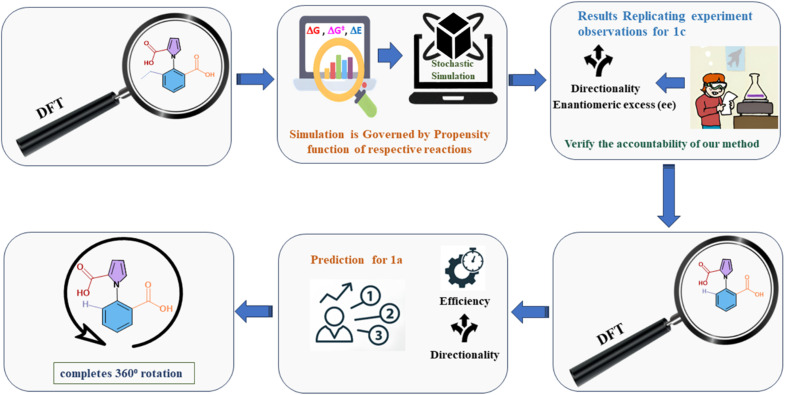
Flow chart of the two-pronged (DFT-Gillespie algorithm) scheme employed to study the ethyl, chloride and hydrogen analogues of the 1-phenylpyrrole 2,2-dicarboxylic acid molecular motor.

The current work addresses these issues by adopting the aforementioned two-pronged computational approach. We have combined a careful understanding of the exact mechanism taking place at each step, with stochastic studies with the Gillespie algorithm (GA). The procedure followed is shown in Fig. 2 below. We note that there have been several theoretical^22^ and computational studies^23^ investigating molecular motors, but the issue of understanding the subtle competing effects (and limitations) of kinetic asymmetry has been addressed here for the first time. We also note that there are a few previous reports where the approach of DFT calculations followed by stochastic simulations has been attempted,^24^ but the use of DFT followed by the reliable exact Gillespie algorithm has been attempted here for the first time (to the best of our knowledge). The current work serves as a demonstration of the power of the two-pronged computational approach that we have proposed and executed. This can be employed to understand the role and the efficiency of kinetic asymmetry in a wide variety of systems in the future.

## Results and discussion

### DFT calculations with 1c (ethyl case)

**Fig. 3:**
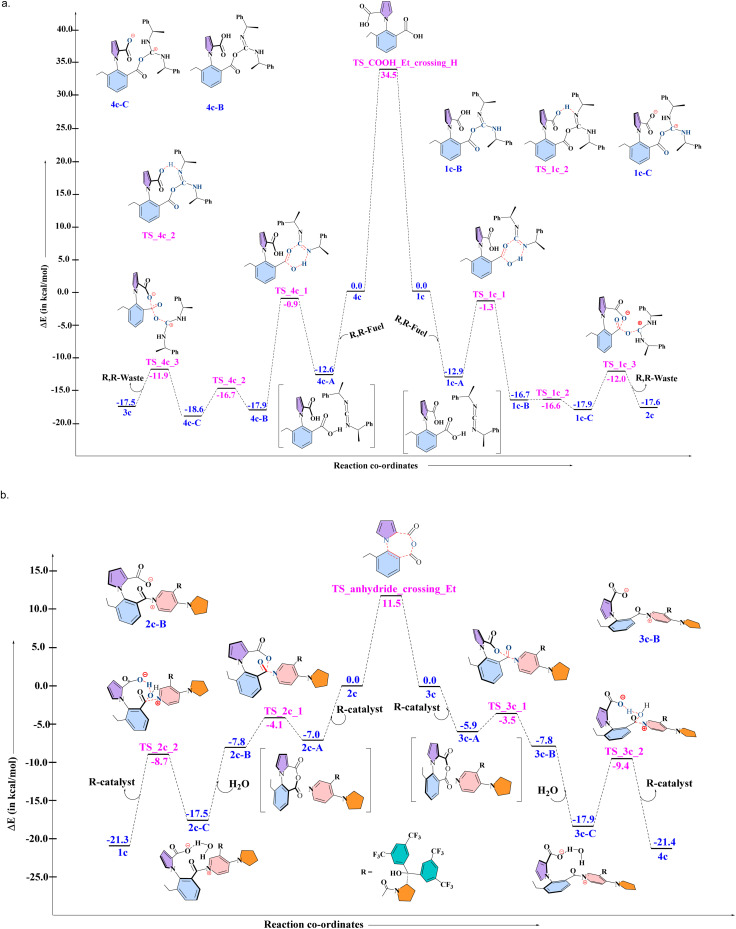
The electronic energy values obtained from DFT, for the combination: 1c, R, R-fuel, for the steps leading to (a) clockwise rotation (1c to 2c), and anticlockwise rotation (4c to 3c) and for the combination: 3c, R-catalyst, for the steps leading to (b) clockwise rotation (3c to 4c), and anticlockwise rotation (2c to 1c).

As mentioned in the introduction, the lack of single molecule experiments makes it impossible to identify the behavior of the actual 1-phenylpyrrole2,2-dicarboxylic acid molecular motor (1a). However, the nature of three out of the four steps that the motor takes has been analyzed experimentally^16^ by employing an analogue of 1a. This was labelled 1c, with an ethyl group instead of hydrogen at the ortho position of the phenyl ring stator, which blocked the last of the four steps of the 360° rotation, and was found experimentally to lead to the buildup of enantiomeric excess of one atropisomer over the other. These experimental results provide us with an excellent opportunity to test the robustness of our proposed DFT-Gillespie algorithm approach, by comparing the % ee values that our approach yields with the experimentally reported outcomes. Therefore, first, detailed quantum chemical (QM) computational studies with density functional theory (DFT) were conducted, in order to evaluate the complete mechanism for the multiple steps involved in the rotation cycle of 1c, when interacting with the chiral fuel and catalyst. The results are shown in Fig. 3a below for the reaction of 1c and 4c reacting with the R, R-fuel, and in Fig. 3b for the reaction of 2c and 3c reacting with the R-catalyst and water, for the combination of 1c, R, R-fuel, and R-catalyst. We note that this comprehensive mechanistic study is itself an improvement of the understanding of the different chemical processes taking place, indicating a three-step fuel–motor reaction, and a two-step reaction involving the catalyst (see Fig. 3). This enhances the understanding over the experimentally reported reaction steps for this system.^16^ The results corroborate the experimental observation of double kinetic gating in this case, indicating a kinetic bias for the chiral fuel induced reaction and for the chiral catalyst. The DFT calculations have been done at the PBE-D3/TZVP level of theory. As the results from the next (stochastic simulation) section will show, this level of theory provides the most suitable comparison to experiment, not only for this motor, fuel and catalyst combination, but also for the other combinations reported in the experimental studies. We also note that we have found that comparing the differences in the electronic energies (Δ*E*_s_ and ΔΔ*E*_s_) provided better comparison to experiment than free energies (Δ*G*_s_ and ΔΔ*G*_s_) for all levels of theory. This is due to the fact that very small changes in the relative energies can have large impacts on the enantioselectivity, and Δ*E*_s_ and ΔΔ*E*_s_ capture the relative changes in energy more accurately than Δ*G*_s_ and ΔΔ*G*_s_, since several approximations are made in order to obtain the free energies for chemical reactions in Turbomole and Gaussian,^25^ which make the comparison of small changes in Δ*G*_s_ and ΔΔ*G*_s_ less reliable than the corresponding small changes in Δ*E*_s_ and ΔΔ*E*_s_. The primary issue is the fact that the translational entropy is incorrectly calculated while determining the thermochemical information from the geometry optimization calculations in softwares such as Gaussian and Turbomole. The Sackur–Tetrode equation, which forms the basis for determining the translational entropy, requires an input for the volume available to the molecule in question, and here, the softwares evaluate the volume from the ideal gas equation, *i.e.* by assuming that the molecule, regardless of its nature, is a part of an ideal gas system.^25^ This leads to enhancement of the translational entropy values, and thus to erroneous values for the entropy, and therefore to the free energy values. This problem is avoided if the electronic energies are considered without the free energy corrections. Hence, it is the differences in the electronic energies that we have focused upon when doing the stochastic simulations with the Gillespie algorithm, discussed in the next section. The corresponding free energy profiles are shown in the Fig. S1 and S2 in the ESI†.

### Stochastic simulations with the obtained DFT results

**Fig. 4:**
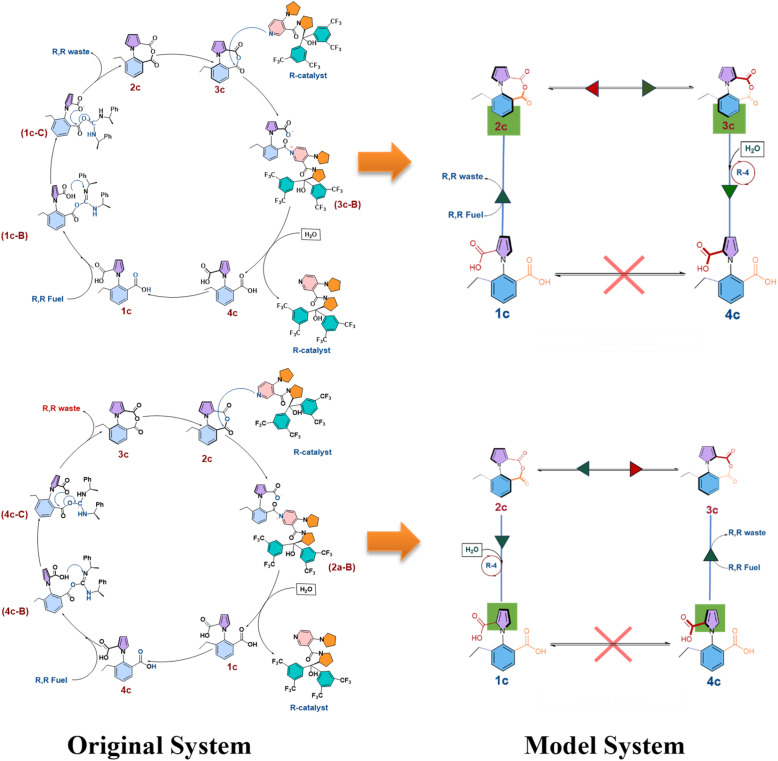
The simplified model system employed for stochastic simulations of 1c.

The next step of the two-pronged approach involved taking the information from the DFT calculations and using them in stochastic simulations with the exact Gillespie algorithm. What the exact Gillespie algorithm requires are the rate constants for the different reactions in the system. These reaction rates were determined by converting the barriers obtained from the DFT calculations to rate constants, by using the Arrhenius equation. Subsequently, a model system was developed for the exact system, as shown in Fig. 4 below.

Effectively, (i) the three step process from 1c to 2c was replaced by a single step (likewise for 4c to 3c), and (ii) the two step process for converting 2c to 1c with the aid of the chiral catalyst and water was replaced by a single step (likewise for converting 3c to 4c). The single step rate constants in the model system were obtained by determining the efficiency of the three-step process by using the energetic span model (ESM),^26^ and then envisaging a single step reaction having the same efficiency, and then determining the activation barrier (and thus the rate constant) for such a single step reaction (see Section S2 in the ESI†). The values of the barriers thus obtained are shown in Table 1 below.

**Table 1:** The barriers for single step reactions, in place of the multiple step processes reported in Fig. 3 above, determined by equating the efficiency of the multiple step processes by an equivalent one step process, with the aid of the energetic span model (ESM)

Fuel and catalyst	1c reacting fuel (Δ*E*^‡^ in kcal mol^−1^)	4c reacting fuel (Δ*E*^‡^ in kcal mol^−1^)	3c reacting catalyst and water (Δ*E*^‡^ in kcal mol^−1^)	2c reacting catalyst and water (Δ*E*^‡^ in kcal mol^−1^)
R, R-fuel and R-catalyst	11.6	11.7	8.5	8.8

This approach allowed us to retain the reliability of the Gillespie algorithm, while making the method more tractable by reducing the number of reactions to be handled by the system. Another modification that was made was that the interconversion by rotation from 1c to 4c, or *vice versa*, was not allowed, since it has been experimentally noted that this rotation step does not take place. Indeed, this is the reason that the species 1c and 4c could be independently observed experimentally, and the % ee evaluated for the same.^16^ Thus, for the combination of R, R-fuel and R-catalyst, a total of six chemical reactions were considered in the Gillespie algorithm approach. This stochastic approach allowed us to determine the change of the different species in the system, until all of the R–R fuel, and all of the intermediate species 2c and 3c had been exhausted. This signaled the end of the reaction. The amount of 1c and 4c that remained at this point allowed us to evaluate the % ee for the system. This procedure was repeated ten times, in order to take into account, the stochasticity of the system, and the average % ee was then calculated for this case. We note that the standard deviation was found to be only ± 1.0%, indicating that each individual run of the stochastic algorithm provided a reliable estimate for the % ee for the system. In the same manner, the % ee was then determined for the following cases: R, R-fuel and R-catalyst, DIC and R-catalyst, DIC and S-catalyst, R, R-fuel and DMAP, and DIC and DMAP. We note here that we have not calculated the barriers for all the cases involving DMAP and DIC, as well as the S-catalyst. In order to compare to all the experimental cases where these entities are involved, we have done the following:

(i) For the cases where achiral fuel or catalyst were employed, the ΔΔ*E*^‡^ was taken to be 0 (which is the expected value).

(ii) For the cases with chirality of catalyst reversed from the R-catalyst that we have studied and mentioned in the Fig. 3 above, we have taken the opposite values for 1c and 4c.

The values obtained are collected together in Table 2 above, along with the experimentally reported values^.^^16^ Considering that even a slight change in the ΔΔ*E*^‡^ leads to considerable change in the values, we note that the numbers for almost all the cases are quite similar to the ones that have been experimentally reported. For instance, if the ΔΔ*E*^‡^ value for the reaction of the R-catalyst with 2c is increased by 0.5 kcal mol^−1^, and if the ΔΔ*E*^‡^ for the reaction of the R, R-fuel with 4c is increased by 0.3 kcal mol^−1^, this leads to change in the % ee from the obtained 34.8% to 78.0%! This is because of the exponential nature of the Arrhenius equation. Therefore, the agreement shown in Table 2 for the different cases can be considered excellent. Benchmark calculations with alternative functionals and basis sets confirmed that PBE-D3/TZVP offers excellent agreement with experimental data, in comparison to other functional and basis set combinations (see Table S9 and Fig. S7 in the ESI†), likely due to cancellation of systematic errors in PBE-D3/TZVP.^27^ Hence, PBE-D3/TZVP was employed throughout this study. We also note that single point calculations (see Tables S1–S4 in the ESI† for Δ*E*^‡^ and Δ*E* values at different level of theory for 1c system) at other, different levels of theory for all the steps of the cycle shown Fig. 3, lead to calculated % ee values that are widely different (>66%) from the obtained experimental values (see Tables S10–S14 in the ESI†), which further indicates that the PBE-D3/TZVP-Gillespie algorithm approach adopted here is optimal for considering the molecular motor system in question.

**Table 2:** Values of Δ*E*^‡^ (in kcal mol^−1^) of different sets of reactions calculated by DFT (level of theory – PBE-D3/TZVP (COSMO: *ε* = 50.28)) and the % ee, determined from stochastic simulations (Gillespie algorithm) for the 1c molecule

Fuel and catalyst	1c reacting fuel (Δ*E*^‡^ in kcal mol^−1^)	4c reacting fuel (Δ*E*^‡^ in kcal mol^−1^)	3c reacting catalyst and water (Δ*E*^‡^ in kcal mol^−1^)	2c reacting catalyst and water (Δ*E*^‡^ in kcal mol^−1^)	ee (pred.) (in%)	ee (exp.) (in%)^16^
R, R-fuel and R-catalyst	11.6	11.7	8.5	8.8	34.3 ± 1.2	41.0
DIC and R-catalyst	11.7	11.7	8.5	8.8	26.4 ± 0.8	35.0
DIC and S-catalyst	11.7	11.7	8.8	8.5	−28.5 ± 1.1	−35.0
DIC and DMAP	11.7	11.7	8.8	8.8	−0.3 ± 1.5	0.0
R, R-fuel and DMAP	11.6	11.7	8.8	8.8	9.1 ± 1.0	7.0

We note an important point here: one could consider the possibility that the anhydride rotation step is very fast in comparison to the other competing chemical reactions. This would be possible if water molecules accelerated the 2c–3c rotation or *vice versa*, since, as mentioned in the introduction, water molecules can accelerate rotational behavior in a machine. In such a scenario, the code would have to be modified to have the number of molecules of 2c and 3c the same at every step – as a reflection of the rapid equilibration of the system due to the fast rotation between the two species (see Fig. S4 and S5 in the ESI†). The Gillespie algorithm was run with this modification, and the obtained ee results for the different cases that had been considered and shown in Table 2 were tabulated (see Table S8 in the ESI†). What was observed is that making this change had a negligible effect on the ee values. This indicates that it is not possible to determine whether water molecules accelerate the anhydride interconversion step or not: such an accelerating effect appears to be minimal in nature. However, it is indeed possible to determine whether water molecules would accelerate the rotation between the atropisomer conformations 1b and 4b, for the chloride analogue of the molecular motor, as will be demonstrated in the next section.

### DFT calculations for 1b (chloride analogue) + stochastic simulations for 1b

**Fig. 5:**
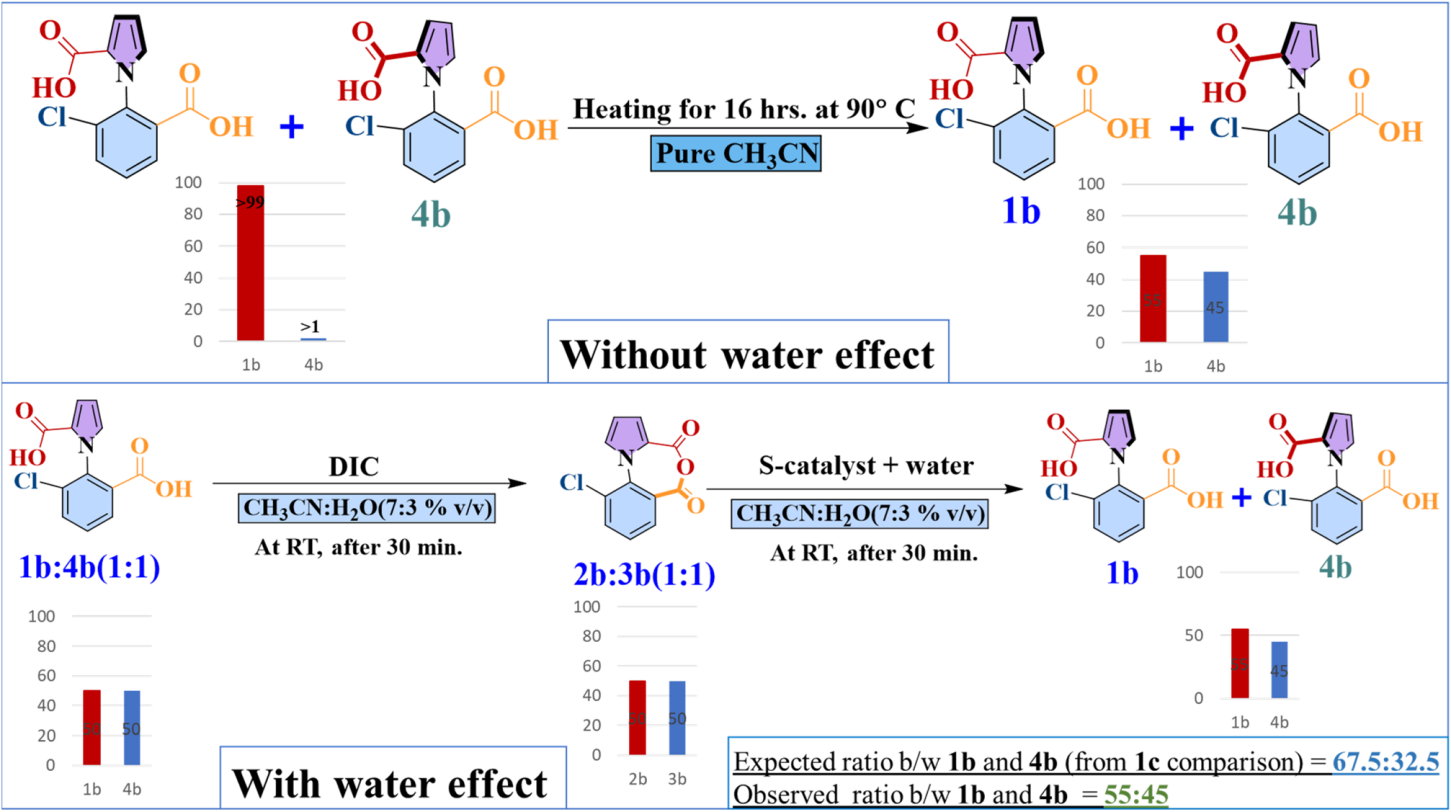
A summary of the experiments with the chloride analogue, 1b, of the molecular motor reported by Leigh and coworkers,^16^ which shows that the presence of water facilitates 1b–4b rotational behavior.

In their 2022 report of the unidirectional molecular motor Leigh and coworkers reported experiments with the chloride analogue 1b.^16^ As shown in Fig. 5 below, the rotation of 1b was explored in neat CH_3_CN solvent, and it was found that it took 16 hours and 90 °C to convert 99% of 1b to 55%. However, subsequently, during the stepwise process of reacting 1b with achiral fuel and with the chiral S-catalyst, it was found that at room temperature and only in half an hour, the ratio of 1b to 4b had become 55% : 45%. The expected 1b : 4b ratio should have been close to 67.5% : 32.5%, which was the ratio obtained by the fuel catalyst combination for 1c (we note that the rotation is now anti-clockwise, because the S-catalyst is being employed). Now, since the ratio for the 1b case is, instead, 55% : 45%, this indicates that there is 1b–4b rotation. This rotation now takes place much faster for 1b than when only neat CH_3_CN was employed as the solvent: it occurs in only half an hour, and just at room temperature, not 90 °C. Why? The answer lies in the fact that the solvent employed now is not neat CH_3_CN, but 70% CH_3_CN and 30% water. In other words, the addition of water makes the rotation of 1b to 4b possible.

This “lubricating” effect of water has been experimentally observed before in rotating molecular machines, when hydrogen bonding groups were present in the rotating arms of the motor,^20^ as is the case in the current system. It has been hypothesized that water molecules effect favorable changes in the transmission coefficient associated with the rotation process, while not affecting the enthalpy of the process.^20^ It is also possible that a three-dimensional chain of water molecules connects the two hydrogen bonding groups and facilitates the rotation.^20^ Capturing such effects with quantum calculations is a very formidable task, which explains why, till date, no quantitative estimates with quantum chemical studies have been reported on the effect of water on rotational processes in molecular machines. However, the two-pronged method that we have developed and showcased in the current work allows us to bypass these problems, because one can estimate the effective barrier of rotation present in this 1b–4b system by employing the Gillespie algorithm and determining what the rotation barrier must be in order to obtain a ratio of 55% : 45% for 1b : 4b. This is what was done. The barriers for the other steps were taken from the calculations for the 1c case, since the chloride functional group is not involved in the other chemical reactions that are part of the kinetic gating steps. After the barrier for the 1b–4b rotation was systematically changed, it was found that the desired ratio of 55% : 45% was obtained when the barrier was 13.9 kcal mol^−1^ (see Fig. S8 in the ESI† for further information).

Now, DFT calculations were done to find the direct rotational barrier for 1b–4b rotation without the effect of water. The barrier was found to be 28.9 kcal mol^−1^ – a value that matches closely with that reported by Leigh and coworkers (Δ*E*^‡^ value calculated by us: 28.9 kcal mol^−1^, reported by Leigh and coworkers: 27.3 kcal mol^−1^, at the ωb97xd/6-31+g(d,p) level of theory.^16^) What this means is that one can now quantify how much of a reduction in the barrier to rotation takes place when water molecules are present: 15.0 kcal mol^−1^ (28.9 kcal mol^−1^ − 13.9 kcal mol^−1^). From the Arrhenius equation, it is seen that this corresponds to an increase in the rate constant for the rotation by more than ten orders of magnitude! It is this extraordinary effect of water that allows the rotation, so inhibited in neat CH_3_CN, to occur in a facile manner in the 70% CH_3_CN, 30% water mixture.

The chloride analogue of the molecular motor thus presents an interesting bridge between the ethyl case (1c) and the actual molecular motor case (1a). In 1c, the 1c–4c rotation is entirely blocked. Why? This is because the direct rotational barrier in the absence of the water effect is calculated to be 34.5 kcal mol^−1^ (Δ*E*^‡^ = 34.5 kcal mol; compares well with the value reported by Leigh and coworkers: 33.3 kcal mol^−1^).^16^ Since the effect of water has been hypothesized to involve coordination though hydrogen bonding between the rotating arms,^20^*i.e.* not involving the ethyl or the chloride functional groups, the reduction in barrier to the rotation that would take place here should be similar to that of the chloride case: 15.0 kcal mol^−1^. This means that the effective barrier for the ethyl case would be 19.5 kcal mol^−1^. When we did simulations with this as the 4c–1c rotational barrier, we found that out of 15 000 possible reaction steps, the 4c–1c (or 1c–4c) rotational steps took place only 7 times on average! This is tantamount to stating that the rotation was blocked for the 1c case. Moreover, the ee that was calculated for this scenario (see Table S15 in the ESI†) (34.6% for the combination of R, R-fuel and R-catalyst) is almost exactly the same as that obtained for the 1c case discussed in Section (ii) above (34.3%). Thus, the effect of water does not alter the reality for the 1c case, which matches experimental observations, and provides a further validation of the approach that we have taken.

What this also means is that for the case of the actual motor 1a, where the direct rotational barrier without the water effect was calculated to be 15.6 kcal mol^−1^, the effective barrier for rotation in the presence of water would be only 0.6 kcal mol^−1^. In other words, the rotation would be nearly a barrierless process for the case of the actual molecular motor. The ramifications of this are discussed in the next section.

### DFT calculations for 1a (hydrogen analogue) + stochastic simulations for 1a

**Fig. 6:**
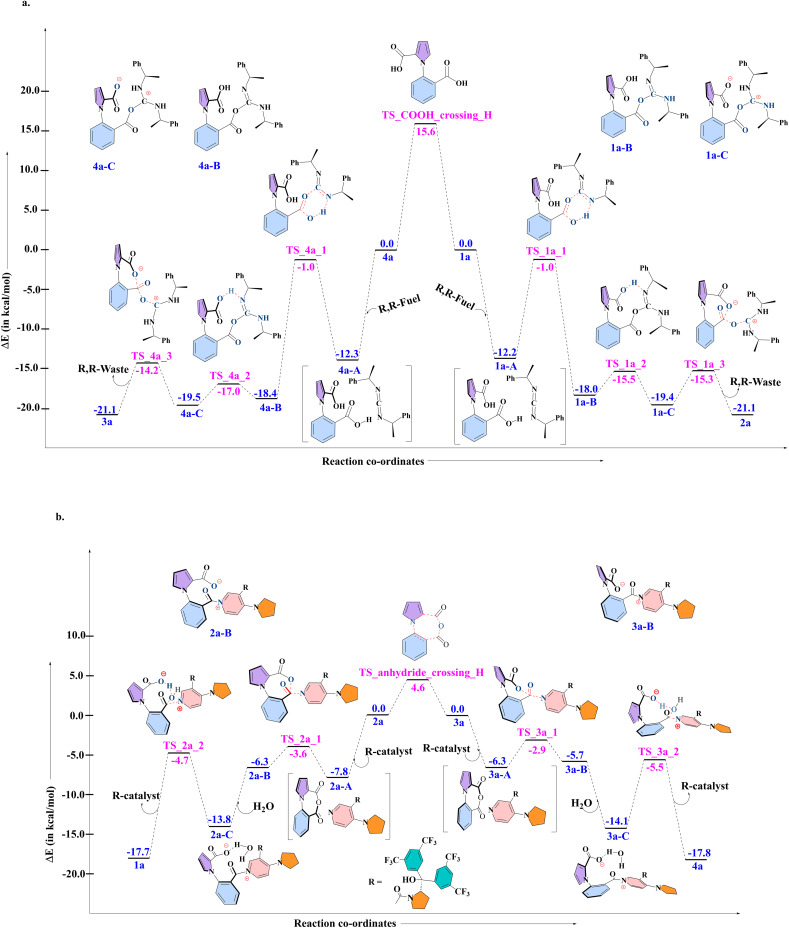
The electronic energy values obtained from DFT, for the combination: 1a, R, R-fuel, for the steps leading to (a) clockwise rotation (1a to 4a), and anticlockwise rotation (4a to 1a) and for the combination: 3a, R-catalyst, for the steps leading to (b) clockwise rotation (3a to 4a), and anticlockwise rotation (2a to 1a).

The previous three sections served the purpose of showcasing our two-pronged approach: DFT followed by stochastic simulations to computationally determine the % ee of the system 1c, in order to compare to available experimental data and thereby validate our approach. As seen, this was done successfully. Then, the crucial role of water in making the rotation from 4b to 1b a facile process was explored. Now, the next step is to apply our validated two-pronged approach to study the actual molecular motor that has been experimentally proposed: 1a. As for 1c, DFT calculations were done for 1a, at the PBE-D3/TZVP level of theory for both of the cycles shown in Fig. 6 below. The Δ*E* values for the different steps were found to be similar to those obtained for 1c, with the notable difference of the conversion of the intermediate 2a to 3a and *vice versa*. The corresponding free energy profiles are shown in the Fig. S9 and S10 in the ESI.† The barrier for this (4.6 kcal mol^−1^) was found to be considerably lower than for the analogous step in 1c (11.5 kcal mol^−1^). This is due to steric factors–the transition state between the two conformers assumes a planar configuration, which extracts a greater price from the ethyl case 1c as compared to the hydrogen 1a. What this implies is that the interconversion between 2a and 3a will be considerably more facile in the case of the actual molecular motor. Also, importantly, as seen in Section (iii), the fact that water reduces the effective barrier for the rotation by 15.0 kcal mol^−1^ showed that the rotation would essentially be a barrierless process for this motor.

The stochastic simulations with the exact Gillespie algorithm were conducted for the system, following the approach outlined and employed in the previous sections. However, here, the interconversion between 2a and 3a was seen to be so rapid that this became by far the dominant reaction in the system, with the other reactions firing only occasionally. This problem was addressed by adopting the strategy employed earlier for the 1c case: the code was modified to have the number of molecules of 2a and 3a the same at every step – as a reflection of the rapid equilibration of the system due to the fast rotation between the two species. Making this modification had the salutary effect of speeding up the Gillespie algorithm process without affecting the reliability of the outcome. This is evidenced by results that were obtained for the 1c, case, as discussed in Section (ii) above. In addition to this, since the rotation from 4a to 1a has been found to be a near barrierless process, the same modification was also done for the rotation reaction steps: of removing the 4a–1a and 1a–4a rotations as competing reactions and finding the amount of 1a and 4a by dividing the total number of molecules (1a + 4a) by 2, to obtain the number of the 1a and 4a species at any point in the reaction. The physical picture this corresponds to is the following: the dominant reaction in the system would be the rapid interconversion between 1a and 4a. However, the reaction of the chiral fuel with the two species 1a and 4a would set the reaction cycle in motion, and from the Curtin Hammett principle, would lead to greater amount of 2a formation over 3a. Equilibration would occur at this stage as well between 2a and 3a, but this would still lead to the kinetic gating effect of greater 1a–2a–3a movement over 4a–3a–2a. Now, the second kinetic gating step of the chiral catalyst and water would ensure greater buildup of 4a over 1a, which after the equilibration of the 4a and 1a atropisomer conformations, would lead to unidirectional 360° rotation.

**Fig. 7:**
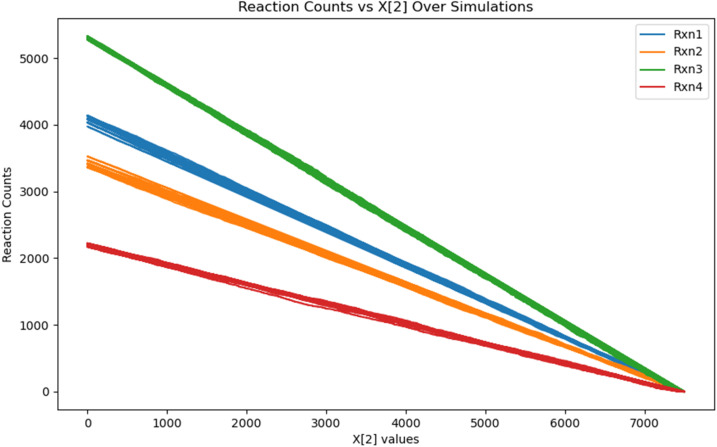
The graph of different reactions occurring in the system as a function of change of fuel. Rxn1: the conversion of 1a–2a; Rxn2: the conversion of 4a–3a; Rxn3: the conversion of 3a–4a; Rxn4: the conversion of 2a–1a. The water effect has been incorporated in the Gillespie algorithm simulations.

The stochastic simulations with the exact Gillespie algorithm confirm this. As shown in the Fig. 7 below, the rate of the reaction 1a–2a is greater than that of the reaction 4a–3a, and the rate of the reaction 3a–4a is greater than that of the reaction 2a–1a. Also, it is clear that the rates of the reactions are independent of the concentration of the fuel, corroborating experimental findings.^17^ The ratio of the clockwise to anticlockwise 360° rotation was found to be 65% : 35%, which is similar to that predicted by Leigh and coworkers in their 2022 report^16^ of 71% : 29%, which was based on the experimental results obtained from the ethyl analogue of the motor, not the actual molecular motor.

Hence, the molecular motor does indeed behave as a motor which has been predicted experimentally. What is important to note, however, is the great influence of water on its behavior. In the presence of the water effect, the results of different reactions fired per simulation are shown in Table 3 below. Our two-pronged approach allows us to determine what the outcome of the molecular motor system would have been had not water played the lubricating role. In that case, the barrier for the 1a–4a and 4a–1a rotations would have been 15.6 kcal mol^−1^. Upon introducing these two rotation reactions into the system and conducting the stochastic simulation studies, we found that the molecular motor would not have completed the 360° rotation for almost all of the concentration range of the fuel.

**Table 3:** Values of Δ*E*^‡^ (in kcal mol^−1^) of different sets of reactions calculated by DFT (PBE-D3/TZVP) (COSMO: *ε* = 50.28), as well as the information of the rotation of the motor gained from the Gillespie algorithm, for *n* = 100. The water effect has been included in the Gillespie algorithm simulations

Type of reaction	Δ*E*^‡^ (in kcal mol^−1^)	Δ*E*^‡^_rot_ (in kcal mol^−1^) (without considering water effect)	Δ*E*^‡^_rot_ (in kcal mol^−1^) (with water effect)	Number of times reactions fired (per simulation)
1a + R, R-fuel → 2a + R, R waste	11.2	15.6	0.6	4092
4a + R, R-fuel → 3a + R, R waste	11.3			3408
3a + water + R-catalyst → 4a + R-catalyst	8.6			5308
2a + water + R-catalyst → 1a + R-catalyst	9.1			2192

This is illustrated in Table S20 and Fig. S13 in the ESI.† We also performed single point calculations at different levels of theory to obtain Δ*E* and Δ*E*^‡^ values for each reaction in the 1a system (see Tables S16–S19 in the ESI†). We then employed the Gillespie algorithm to obtain the behavior of 1a at each level of theory with single point calculations energy values (see Tables S21–S25†).

### Effect of water on the dynamics of the gel embedded motor, and on biological systems

The insight into the crucial role played by water in facilitating the rotation also allows us to understand the upper bound of the effective rotational barrier in modified motor systems. Leigh, Giuseppone and coworkers have very recently reported a modified version of the molecular motor, embedding it into a gel matrix.^17^ What they observed was that upon addition of the fuel, the system contracted the strands of the gel, taking about 5–60 hours to complete the contraction process. This contraction was a result of the 360° rotation of the motor. The reason it took so much time to rotate is because of the buildup of force resisting the rotation, from the twisting strands of the gel. This resistance had the effect of increasing the rotational barrier of the final step. Further evidence of this resistance is demonstrated by the fact that reversing the rotation – *i.e.* re-expanding the gel took only 0–5 hours. Now, is it possible to determine the upper limit to which the rotational barrier can increase, with the motor still being able to rotate?

The answer can be obtained from our two-pronged approach. Taking the non-embedded molecular motor, we find that, despite resistance, the rotation can take place up to an effective barrier value of 18.0 kcal mol^−1^. This value was obtained by changing the barrier until the stochastic simulations showed that the 360° rotation was no longer taking place. Since there is a reduction of the actual barrier to rotation by 15.0 kcal mol^−1^, due to the lubricating effect of water, this means that the rotational barrier for the system in this gel-embedded matrix can go up to a maximum of 33.0 kcal mol^−1^ (33.0 = 18.0 + 15.0 kcal mol^−1^). Further increase in resistance of the twisted strands of the gel, *i.e.* further increase in the barrier, would lead to stoppage of the rotation. It is also clear that if the water effect had not been present, then the resisting force from the twisted strands would soon have increased the rotational barrier above the 15.6 kcal mol^−1^ that has been calculated for the molecular motor 1a without water assistance, and thus the 4a–1a rotation, seen only to take place at very low concentrations of fuel without the water effect for 1a, would not have occurred at all. Hence, the current work sheds light on the ability of the motor to endure resistance and continue rotating, thanks to the extraordinary facilitation provided by water.

Additionally, the finding from this work that the rotational rate constant is increased by more than ten orders of magnitude due to the influence of water also allows one to speculate on the evolution of life. Biomolecular machines are crucial for life, and all biomotors have evolved in the presence of water. Indeed, the gel embedding that was experimentally done for the motor was in order to mimic biomolecular machines such as myosin, which are embedded in muscle filaments in the body. Moreover, a new class of chiral azaindole – phenyl ethanoic acid rotary motors, mimicking motor proteins, has been recently reported by the Leigh group.^28^ Our current work indicates that the effect of water is harnessed by this biomimetic system for 360° rotation. The current work also suggests that rotary biomotors such as prokaryotic flagella, which are made up of protein subunits of flagellin, consisting of amino acids that are capable of hydrogen bonding in water, are likely to gain considerable assistance from the surrounding molecules of water in executing rotatory motion. Such significant accelerating effects in water can provide an explanation for the ubiquity and proliferation of rotating molecular machines in biological systems.

## Conclusion

In the past ten years (2015–2024), there have been more than 161 000 papers published in the area of kinetic asymmetry in chemical systems, and more than 50 000 papers published in the area of unidirectional molecular motors.‡‡All the data reported is taken from the source: https://app.dimensions.ai/ These numbers show how significantly the field of kinetic asymmetry has grown in recent years, and how widely its principles have been exploited experimentally in developing interesting new applications. Importantly, the application of kinetic asymmetry to unidirectional molecular motors has given rise to some of the most elegant examples in this area in the recent past. However, despite this surge of experimental interest, there still exists a lack of a computational approach that can provide precise understanding of the behavior of such systems, and how the behavior would be affected by the change in all the influencing factors: information that is beyond the reach of current day experimental methods.

The current computational work unveils a strategy that can be adapted to all one pot chemical systems employing kinetic asymmetry. Specifically, a two-pronged approach has been proposed: the evaluation of the rate constants of competing reactions through DFT calculations, followed by use of this information in stochastic simulations with the exact Gillespie algorithm. The efficacy of this approach has been demonstrated here for one of the most elegant recent examples of kinetic asymmetry: a molecular motor based on 1-phenylpyrrole 2,2′-dicarboxylic acid that can undergo 360° rotatory motion in one direction through a double kinetic gating mechanism.^16^ Having first validated our two-pronged approach with the ethyl analogue of this molecular machine, by successfully comparing to reported % ee results, we have then proceeded to determine the influence of water in assisting the rotation of the motor in the final step, by investigating the chloride analogue of the molecular machine. The results indicated that water increased the rotational rate constant of the final step by more than ten orders of magnitude! This remarkable favorable effect of water had the happy consequence of making the rotational barrier for the final step nearly barrierless for the actual molecular motor. When the validated stochastic model was then employed to evaluate its behavior, it was found that the motor behaved in a consistent manner over the entire concentration range of the fuel, and displayed a 65% : 35% directionality, coming close to experimental predictions for the same.^16^

**Fig. 8:**
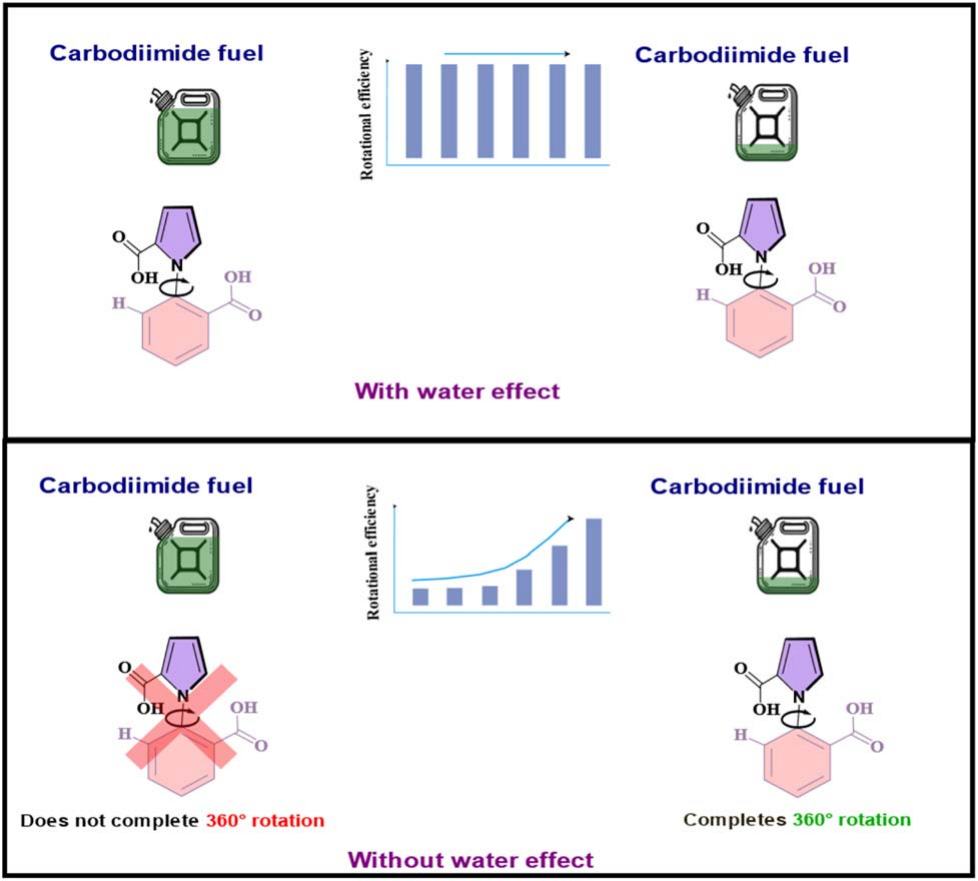
The contrast between the reality: with the water effect present and the alternative where the water effect absent.

It is, however, instructive to see what the behavior of the motor would have been like without this extraordinary lubricating effect of water. When stochastic simulations were done with the water effect absent, it was found that the motor would not have rotated 360° at all, but would have oscillated between two atropisomeric conformers, 1a–4a, through the intermediates 2a and 3a, for the majority of the fuel concentration range, and only managed to execute the complete rotation when the fuel concentration became very low. The stark contrast between the two scenarios: with and without the effect of water, is illustrated in Fig. 8 below. Additionally, it was seen that without the powerful facilitating effect of water, the recently reported gel-embedded molecular motor^17^ would not work at all. Thus, the effect of water is seen to emerge as the *pièce de résistance*, without which the functioning of the machine would be severely compromised. Moreover, since such recent biomimetic motor work focuses on understanding how biomolecular machines evolved, the current work also allows us to speculate on the crucial role water must have played in developing molecular machines. Thus, the current computational investigation allows us to peer deep into the workings of the unidirectional, rotating molecular machine, and glean important effects that are elusive to experiment.

This demonstration makes it clear that the two-pronged approach that we have showcased can make a strong impact on systems that are based on dynamic kinetic resolution,^29^ which is an integral part of synthesizing new molecular motors and switches. It can also be very useful for studying systems related to endergonic synthesis based on ratchet mechanisms, where the yield is often quite low,^30^ by finding the reasons leading to low yield. Moreover, since kinetic asymmetry plays an important role in non-equilibrium systems, the current approach can shed insight into fields as varied as asymmetric catalysis,^31^ chiral synthesis,^32^ and the origin of life.^33^ Indeed, the potential of the two-pronged approach revealed here is diverse and far-reaching!

## Methods

### Computational details

All the calculations in this study have been performed with density functional theory (DFT), with the aid of the Turbomole 7.5 suite of programs,^34^ using the PBE functional,^35^ along with dispersion correction (DFT-D3).^36^ The TZVP^37^ basis set for all other atoms has been employed. The resolution of identity (RI),^38^ along with the multipole accelerated resolution of identity (marij)^39^ approximations have been employed for an accurate and efficient treatment of the electronic Coulomb term in the DFT calculations. A solvent correction was incorporated with optimization calculations using the COSMO model,^40^ with acetonitrile (CH_3_CN): water (H_2_O) (7 : 3(% v/v)) (*ε* = 50.28, determined by taking the weighted average of the dielectric constants of acetonitrile (*ε* = 37.5) and water (*ε* = 80.1)) as the solvent. Conformational analysis for searching for the more stable conformers has been done with the help of the CREST^41^ software and GFN2-xTB.^42,43^ The free energy (Δ*G*) values, were calculated with zero-point energy corrections, and with internal energy and entropic contributions included through frequency calculations on the optimized minima, with the temperature taken to be 283.15 K. Harmonic frequency calculations were performed for all stationary points to confirm them as local minima or transition state structures. The translational entropy term in the all-calculated structures was corrected through a free volume correction introduced by Mammen *et al.*^44^ This volume correction was done in orders to account for the unreasonable enhancement in translational entropy that is generally observed in computational softwares. However, as discussed in the results and discussion section, it is the differences in the electronic energies (Δ*E*_s_ and ΔΔ*E*_s_) that we have focused upon when doing the stochastic simulations with the Gillespie algorithm.

In order to computationally obtain the diastereoselectivity of 1c in the presence of chiral fuel and chiral catalyst, as well as computationally predict the 360° rotation in 1a, we employed Python based codes for chemical reaction simulations using the exact Gillespie algorithm.^15^ The Gillespie algorithm is pivotal in simulating chemical systems with numerous reactions, allowing for the random selection of events based on reaction probabilities and the generation of time intervals for each event. The central idea of the Gillespie algorithm is that, at a given moment in time, in the next time step, the choice of one firing of one among all the competing reactions in the system is done by comparing the relative probabilities of the competing reactions. These relative probabilities are calculated by determining their “propensity functions”: the product of their reaction parameters (analogous to rate constants) with the concentrations of the different reactant species at that point in time. As the concentrations of the different species change with time, so do the propensity functions of the different competing reactions, and thus, the number of firings of the different reactions over time. This, therefore, provides an excellent means of determining which reactions predominate at different points of time in the system, with the change in the concentration of the different species. We note that modified model systems have been employed, where necessary, in place of the real system, in order to expedite the calculations with the Gillespie algorithm. All modifications have been validated by comparing the computationally obtained results, for the stochastic simulations with 1c, with the corresponding values obtained experimentally (see the results and discussion section).

## Author contributions

K. V. conceptualised the work. P. B. performed all the DFT calculations. S. T. and P. B. developed the code.

## Conflicts of interest

There are no conflicts to declare.

## Supplementary Material

SC-016-D5SC03256C-s001

## Data Availability

The authors declare that all supporting data are available in the ESI† and from the corresponding author upon reasonable request. All the codes used in this manuscript are available on GitHub (https://github.com/priyam1720/Molecular-machines).
