# Supplementary material for: The critical helping hand of water: theory shows the way to obtain elusive, granular information about kinetic asymmetry driven systems
Source: Chem Sci. 2025 Jul 21;16(33):14940–55. doi: 10.1039/d5sc03256c (PMC12291277; doi:10.1039/d5sc03256c)
Supplement: SC-016-D5SC03256C-s001 [file SC-016-D5SC03256C-s001.pdf]

Supplementary Information (SI) for Chemical Science.
This journal is © The Royal Society of Chemistry 2025

Supporting Information

The Critical Helping Hand of Water: Theory Shows the Way to Obtain Elusive, Granular Information about Kinetic Asymmetry Driven Systems

*Priyam Bajpai,[ab] Shrivatsa Thulasiram[c] and Kumar Vanka [ab]**

[a]Physical and Materials Chemistry Division, CSIR-National Chemical Laboratory, Pune-411008, India.

[b]Academy of Scientific and Innovative Research (AcSIR), Ghaziabad- 201002, India

[c] Indian Institute of Science Education and Research, Pune-411008, India

*Corresponding: k.vanka@ncl.res.in

Table of Contents

1. Free energy profile diagram for 360° rotation for **1c** and **4c** **3 - 4**
2. Energetic span model (ESM) **5 - 6**
3. Single point calculations for 360° rotation cycle of **1c** and **4c** **7-11**
4. Stochastic simulations for the **1c** system **12-13**
5. % ee calculations by stochastic simulations for the **1c** system without the effect of water **14-20**
6. Stochastic simulations for **1b** and % ee calculations for **1c** with water effect **21-22**
7. Free energy profile diagram for 360° rotation for **1a** and **4a** **23-24**
8. Single point calculations for 360° rotation cycle of **1a** and **4a** **25-29**
9. Stochastic simulations for the **1a** system, with the effect of water included **30-31**
10. Stochastic simulations for the **1a** system, without the effect of water **32-39**
11. Volume conversions **40**
12. Direct vs machine-catalysed hydration of R, R fuel **41**
13. Alternative mechanism to convert **1a** into **4a** by reacting with **2a** and **3a** **42**
14. NCI and BCP analysis of rotational barrier of **1a** and **1c** molecules **43-44**
15. References **45**
16. Cartesian coordinates **46- 331**

1. **Free energy profile diagram for 360° rotation for 1c and 4c.**

Starting with the clockwise direction of movement, (Figure S1) **1c** reacts with the fuel to form an adduct **1c-A**, which is 9.5 kcal/mol more stable than the initial species. **1c-A** converts into **1c-B** *via* a 6-membered transition state (ΔG‡ = 10.9 kcal/mol). Subsequently, **1c-B** transforms into the third intermediate, **1c-C**, through a simple intramolecular proton transfer (ΔG‡ = -1.5 kcal/mol). Finally, **1c-C** converts into **2c** through ring-closing, with a barrier (ΔG‡) of 6.2 kcal/mol.

In case of anticlockwise direction of movement, **4c** reacts with fuel to form an adduct **4c-A**, which is 8.9 kcal/mol more stable than the initial species. **4c-A** converts into **4c-B** *via* a 6-membered transition state (ΔG‡ = 11.3 kcal/mol). Subsequently, **4c-B** transforms into the third intermediate, **4c-C**, through a simple intramolecular proton transfer (ΔG‡ = -1.0 kcal/mol). Finally, **4c-C** converts into **3c** through ring-closing, with a barrier (ΔG‡) of 6.8 kcal/mol.

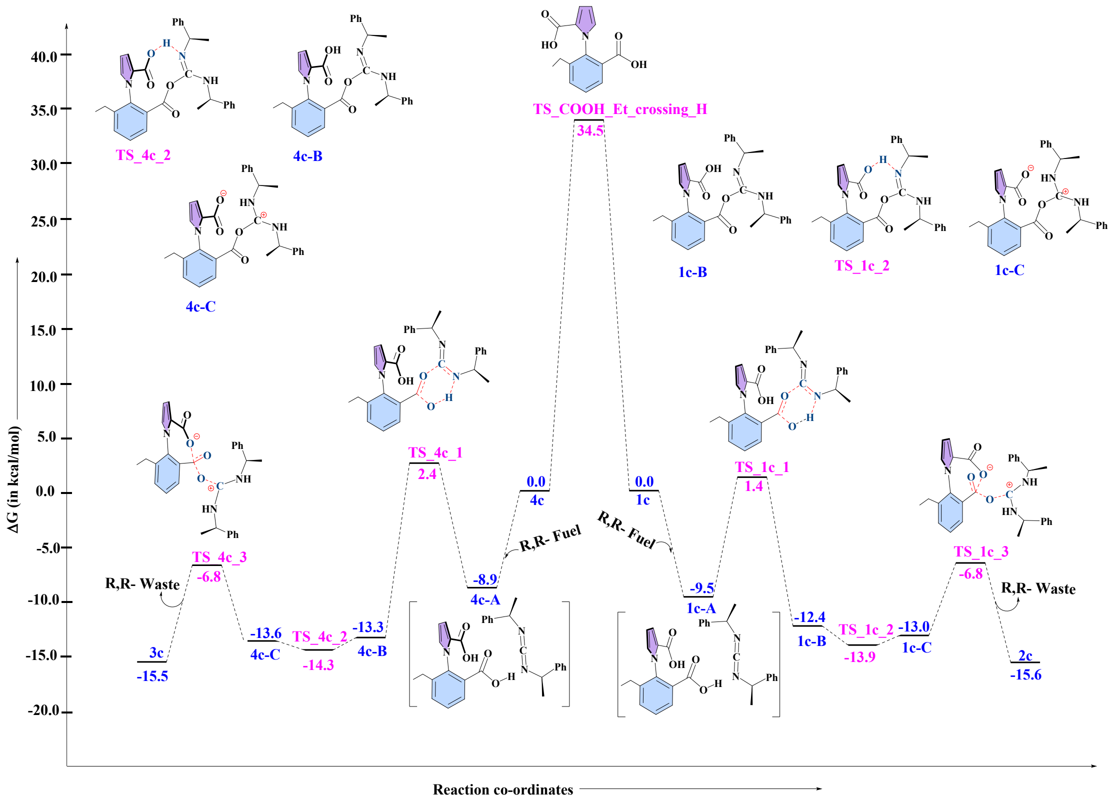

Figure S1. The free energy profile diagram of **1c**/**4c** reacting with R,R – Fuel to form **2c**/**3c**. Level of theory: PBE-D3/TZVP (COSMO: ε =50.28). Temperature: 283.15 K).

For the clockwise rotation, (Figure S2), the conversion of **2c** to **3c** involves a rotation, with a barrier of 11.4 kcal/mol. Subsequently, **3c** forms an adduct **3c-A** with the R-catalyst, which is 3.1 kcal/mol

more stable than the initial species. **3c-A** undergoes a ring-opening reaction in the presence of the R- catalyst to produce the intermediate, **3c-B**, with a barrier (ΔG‡) of 2.8 kcal/mol. **3c-B** reacts with water to form intermediate **3c-C**, which is 6.8 kcal/mol more stable than water and **3c-B**. **3c-C** converts into **4c** and regenerates the R-4 catalyst, with a barrier (ΔG‡) of 6.9 kcal/mol. Finally, **4c** transforms into **1c** through a simple C-N bond rotation, with a barrier (ΔG‡) of 34.5 kcal/mol to complete 360° rotation.

For the anticlockwise rotation, **3c** converts into **2c**, with a barrier of 11.4 kcal/mol. Subsequently, **2c** forms an adduct **2c-A** with R-catalyst which is 3.7 kcal/mol more stable than initial species. **2c-A** undergoes a ring-opening reaction in the presence of the R- catalyst to produce the intermediate, **2c-B**, with a barrier (ΔG‡) of 3.0 kcal/mol. **2c-B** reacts with water to form intermediate **2c-C**, which is 6.6 kcal/mol more stable than water and **2c-B**. **2c-C** converts into **1c** and regenerates the R-4 catalyst, with a barrier (ΔG‡) of 7.7 kcal/mol. Finally, **1c** transforms into **4c** through a simple C-N bond rotation, with a barrier (ΔG‡) of 34.5 kcal/mol, to complete 360° rotation.

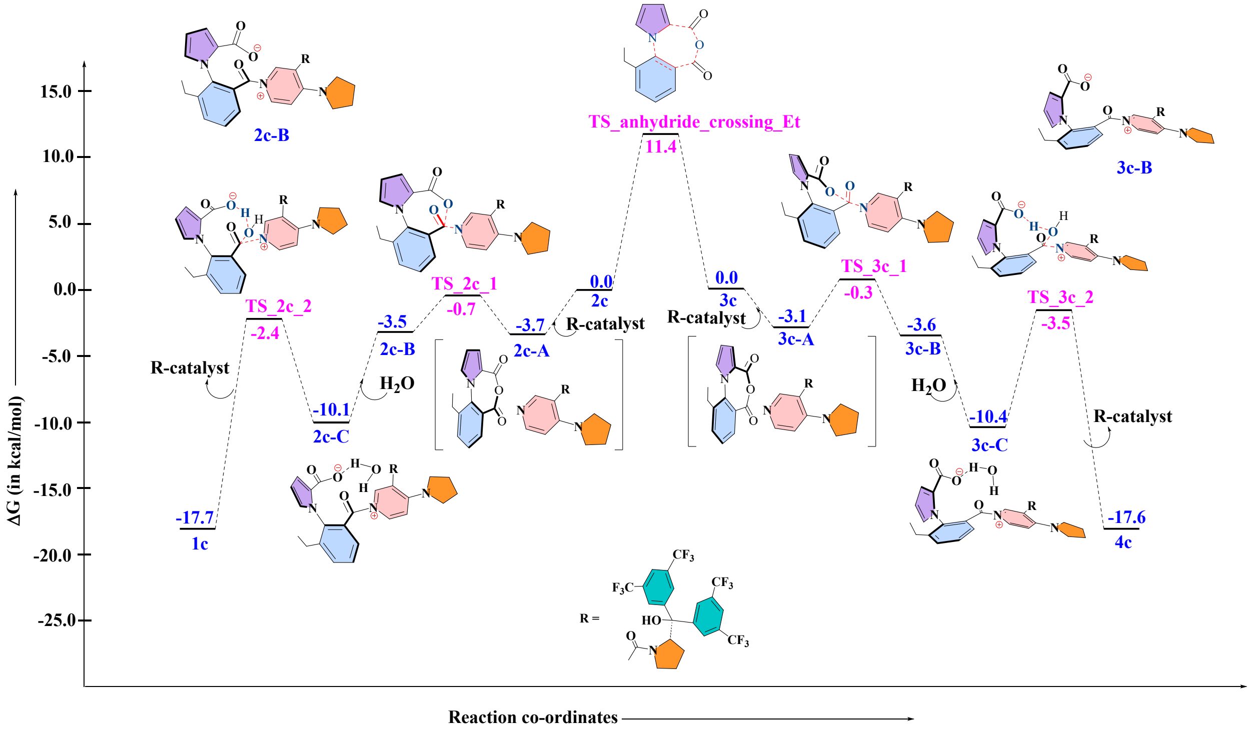

Figure S2. The free energy profile diagram of **3c**/**2c** reacting with R,R – Fuel to form **4c**/**1c**. Level of theory: PBE-D3/TZVP (COSMO: ε =50.28); temperature: 283.15 K.

2. Energetic span model (ESM)

The Turnover frequency (TOF) of the catalytic cycle can be calculated through the energetic span model (ESM) as developed by Shaik and co-workers.[1–4]The ESM imparts an easy method to determine the turnover frequencies (TOFs) of catalytic cycles based on their computed energy profiles. In most cases, the TOF is calculated by the TOF-determining transition state (TDTS), the TOF-determining intermediate (TDI), and by the reaction energy, ΔG_r, as shown below

$$TOF = \frac{kBT}{h} e^{-\delta E/RT}$$

where δE is the energy span and is defined as the Gibbs energy difference between the TDTS and the TDI, with the addition of the ΔG_r when the TDTS appears before the TDI. δE is the effective activation energy barrier of the global reaction. The TDTS and TDI are the intermediate and the transition state, respectively, that maximize δE, according to Equation 1.

$$\delta E = \begin{cases} TDTS - TDTI, & \text{if TDTS appears after TDI} \\ TFTS - TDI + \Delta Gr, & \text{if TDTS appears before TDI} \end{cases} \quad (1)$$

This model has been employed to calculate the TOFs (at 283.15 K for hydrogen substituted and 283.15 K for the ethyl substituted motor molecule). The ESM can be applied in a user-friendly way with the recently developed AUTOF computer program.[1-4]

We divided the complete cycle into two parts. The first part involves converting diacid to anhydride using a chiral R,R-fuel. The second part involves hydrolyzing the anhydride back to diacid using a chiral R- catalyst and water. Efficiency is calculated using the ESM model for each part, considering their respective kinetic and thermodynamic values.

Although both parts are multi-step processes, in the stochastic modeling we have treated each as a single-step process. This was done in order to create a model for the reaction cycle that would be more easy to evaluate with the exact Gillespie algorithm.(See Figure S3) What is important is that the barrier for the modeled single step process had to be correctly evaluated. This was determined as follows: we evaluated the barrier for the single-step process by first finding the

efficiency of the multi-step process with the ESM approach, and then determining the barrier at which the single-step process would, by use of the same ESM approach, provide an efficiency that would match the efficiency of the multi-step process.

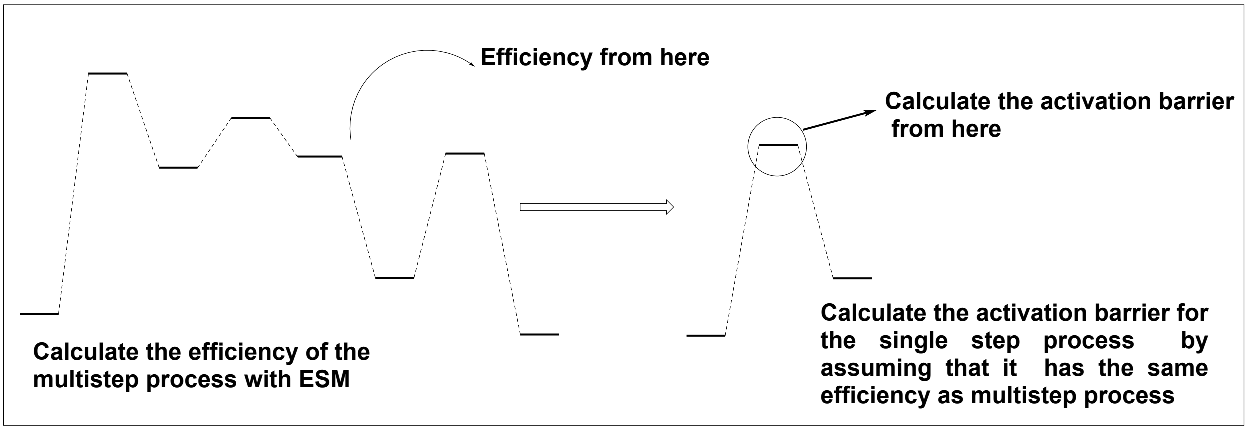

Figure S3. The strategy that was employed to determine the barrier of activation for the modeled single step process from the multistep process with the help of ESM.

3. **Single point calculations for 360° rotation cycle of ethyl substituted motor system (1c and 4c)**

After performing full DFT calculations at the PBE-D3/TZVP level (COSMO: ε = 50.28), we conducted single-point calculations for ethyl substituted motor system using the def2-TZVP[5] basis set and five different levels of theory: B3LYP[6], M06-2X[7], PBE0[8], TPSSH[9], and WB97X-D[10]. The results are provided below in Tables S1-4. Solvent effects and dispersion corrections (D3) were also incorporated in the single-point calculations.

Table S1. Thermodynamic and kinetic parameters for the conversion of 1c to 2c with the R,R-Fuel.

S.no	Level of theory	R,R-fuel +1c ➔ 1c-A	1c-A➔ 1c-B		1c-B➔ 1c-C		1c-C➔ 2c +R,R-waste		(For single step)
		ΔE	ΔE‡	ΔE	ΔE‡	ΔE	ΔE‡	ΔE	ΔE‡
1	PBE-D3/TZVP (COSMO: ε=50.28),	-12.9	11.6	-3.8	0.1	-1.2	5.9	0.3	11.6
2	B3LYP-D3/def2-TZVP (COSMO: ε=50.28) //PBE-D3/TZVP (COSMO: ε=50.28)	-11.9	14.0	-2.0	0.7	-2.1	8.5	-0.3	14.0
3	M06-2x-D3/def2-TZVP (COSMO: ε=50.28) //PBE-D3/TZVP (COSMO: ε=50.28)	-10.0	15.6	-9.1	0.3	-0.8	9.5	1.6	15.6
4	PBE0-D3/def2-TZVP (COSMO: ε=50.28) //PBE-D3/TZVP (COSMO: ε=50.28)	-12.0	13.9	-8.1	0.4	-1.5	9.0	-0.5	13.9
5	TPSSH-D3/def2-TZVP	-11.4	12.9	-4.9	0.6	-1.7	6.3	0.9	12.9

	(COSMO: ε=50.28) //PBE-D3/TZVP (COSMO: ε=50.28)								
**6**	WB97X-D-D3/def2-TZVP (COSMO: ε=50.28) //PBE-D3/TZVP (COSMO: ε=50.28)	**-10.0**	**14.2**	**-7.5**	**0.3**	**-2.5**	**9.0**	**-1.0**	**14.2**

Table S2. Thermodynamic and kinetic parameters for the conversion of 4c to 3c with the R,R-fuel.

**S.no**	**Level of theory**	**R,R-fuel +4c ➔ 4c-A**	**4c-A➔ 4c-B**		**4c-B➔ 4c-C**		**4c-C➔ 3c +R,R-waste**		**(For single step)**
		**ΔE**	**ΔE‡**	**ΔE**	**ΔE‡**	**ΔE**	**ΔE‡**	**ΔE**	**ΔE‡**
**1**	PBE-D3/TZVP (COSMO: ε=50.28),	**-12.6**	**11.7**	**-5.3**	**1.2**	**-0.7**	**6.7**	**1.1**	**11.7**
**2**	B3LYP-D3/def2-TZVP (COSMO: ε=50.28) //PBE-D3/TZVP (COSMO: ε=50.28)	**-11.9**	**14.9**	**-3.8**	**2.6**	**-1.3**	**10.7**	**0.7**	**14.9**
**3**	M06-2x-D3/def2-TZVP (COSMO: ε=50.28) //PBE-D3/TZVP (COSMO: ε=50.28)	**-10.5**	**17.5**	**-8.6**	**2.4**	**-1.2**	**11.7**	**2.0**	**17.5**
**4**	PBE0-D3/def2-TZVP (COSMO: ε=50.28) //PBE-D3/TZVP (COSMO: ε=50.28)	**-11.9**	**14.6**	**-9.4**	**1.6**	**-0.7**	**9.4**	**0.1**	**14.6**
**5**	TPSSH-D3/def2-TZVP (COSMO: ε=50.28) //PBE-	**-11.2**	**13.2**	**-6.4**	**2.3**	**-1.1**	**7.9**	**0.1**	**13.2**

	D3/TZVP (COSMO: ε=50.28)								
**6**	WB97X-D-D3/def2-TZVP (COSMO: ε=50.28) //PBE-D3/TZVP (COSMO: ε=50.28)	**-10.2**	**15.3**	**-8.7**	**3.1**	**-1.8**	**11.7**	**-0.3**	**15.3**

Table S3. Thermodynamic and kinetic parameters for the conversion of 3c to 4c with the R-catalyst and water.

**S.no**	**Level of theory**	**R-catalyst +3c ➔ 3c-A**	**3c-A➔ 3c-B**		**3c-B+ water ➔ 3c-C**	**3c-C➔ 4c + R-catalyst**		**(For single step)**
		**ΔE**	**ΔE‡**	**ΔE**	**ΔE**	**ΔE‡**	**ΔE**	**ΔE‡**
**1**	PBE-D3/TZVP (COSMO: ε=50.28),	**-5.9**	**2.4**	**-1.9**	**-10.1**	**8.5**	**-3.5**	**8.5**
**2**	B3LYP-D3/def2-TZVP (COSMO: ε=50.28) //PBE-D3/TZVP (COSMO: ε=50.28)	**-6.0**	**6.7**	**0.2**	**-10.9**	**12.8**	**5.4**	**12.8**
**3**	M06-2x-D3/def2-TZVP (COSMO: ε=50.28) //PBE-D3/TZVP (COSMO: ε=50.28)	**-5.7**	**5.2**	**0.6**	**-10.2**	**11.4**	**-7.9**	**11.4**
**4**	PBE0-D3/def2-TZVP (COSMO: ε=50.28) //PBE-D3/TZVP (COSMO: ε=50.28)	**-5.4**	**4.6**	**0.3**	**-10.5**	**11.3**	**-7.2**	**11.3**
**5**	TPSSH-D3/def2-TZVP	**-5.1**	**4.5**	**0.4**	**-9.5**	**10.8**	**-7.1**	**10.8**

	(COSMO: ε=50.28) //PBE-D3/TZVP (COSMO: ε=50.28)							
**6**	WB97X-D-D3/def2-TZVP (COSMO: ε=50.28) //PBE-D3/TZVP (COSMO: ε=50.28)	**-5.6**	**6.9**	**1.1**	**-10.1**	**13.2**	**-7.8**	**13.2**

Table S4. Thermodynamic and kinetic parameters for conversion of 2c to 1c with R-catalyst and water.

**S.no**	**Level of theory**	**R-catalyst +2c ➔ 2c-A**	**2c-A➔ 2c-B**		**2c-B+ water ➔ 2c-C**	**2c-C➔ 1c + R-catalyst**		**(For single step)**
		**ΔE**	**ΔE‡**	**ΔE**	**ΔE**	**ΔE‡**	**ΔE**	**ΔE‡**
**1**	PBE-D3/TZVP (COSMO: ε=50.28),	**-7.0**	**2.9**	**-0.8**	**-9.7**	**8.8**	**-3.8**	**8.8**
**2**	B3LYP-D3/def2-TZVP (COSMO: ε=50.28) //PBE-D3/TZVP (COSMO: ε=50.28)	**-7.0**	**7.2**	**0.5**	**-10.1**	**13.7**	**-5.5**	**13.7**
**3**	M06-2x-D3/def2-TZVP (COSMO: ε=50.28) //PBE-D3/TZVP (COSMO: ε=50.28)	**-6.9**	**5.4**	**0.4**	**-8.9**	**12.8**	**-8.2**	**12.8**
**4**	PBE0-D3/def2-TZVP (COSMO: ε=50.28) //PBE-D3/TZVP (COSMO: ε=50.28)	**-6.5**	**5.3**	**0.9**	**-9.7**	**11.8**	**-7.3**	**11.8**
**5**	TPSSH-D3/def2-TZVP	**-6.2**	**5.3**	**1.1**	**-8.6**	**11.4**	**-7.3**	**11.4**

	(COSMO: ε=50.28) //PBE-D3/TZVP (COSMO: ε=50.28)							
**6**	WB97X-D-D3/def2-TZVP (COSMO: ε=50.28) //PBE-D3/TZVP (COSMO: ε=50.28)	**-6.7**	**6.8**	**0.6**	**-9.2**	**<u>13.9</u>**	**-7.3**	**13.9**

4. Stochastic simulations for the 1c system.

$R = K[A]$

First order kinetics $\frac{-\ dna}{V * NA *\ dt} = \mathbf{K} \frac{na}{V *\ NA}$

$$\frac{-\ dna}{dt} = \mathbf{K} * na$$

$$R = K[A][B]$$

Second order kinetics[10] $\frac{-\ dna}{V * NA *\ dt} = \mathbf{K} \frac{na}{V *\ NA} * \frac{nb}{V *\ NA}$

$$\frac{-\ dna}{dt} = \mathbf{K} \frac{na * nb}{V * NA}$$

For the volume of 10 $^{\wedge-18}$ L , the second order reaction is 6,00,000 times slower than the first order reaction.

As discussed in the main manuscript, we made some changes in Figure S4, which are reflected in Figure S5: two reactions: **1c** and **4c** with the fuel taken, as before, as single reactions, and two reactions: **2c** and **3c** with the catalyst, taken, as before, as single reactions, with the concentrations of **2c** and **3c** taken at any time to be X/2. In other words, 4 reactions were considered in this approach. That this is conceptually correct is shown by looking at the results of the Gillespie algorithm with all six reactions included: the simulations were considerably slowed down by the dominating interconversion between **2c** and **3c** whose only effect was to create equality between the two species, without taking the system forward with respect to the other reactions involved. Converting this to the modified four reaction system had the effect of including the implicit equilibration between the species **2c** and **3c**, without altering the outcome that would have occurred for the six reaction system. In other words, this approach had the salutary effect of speeding up the Gillespie algorithm process without affecting the reliability of the outcome of the process. excluding anhydride rotation barrier and including diacid rotational barrier (**1c** ➔ **4c** or vice versa) is used when we performed stochastic simulations for the **1c** system with values obtained by single point calculations at a different level of theory. ***All the codes used in this manuscript are available on GitHub. (https://github.com/priyam1720/Molecular-machines)***

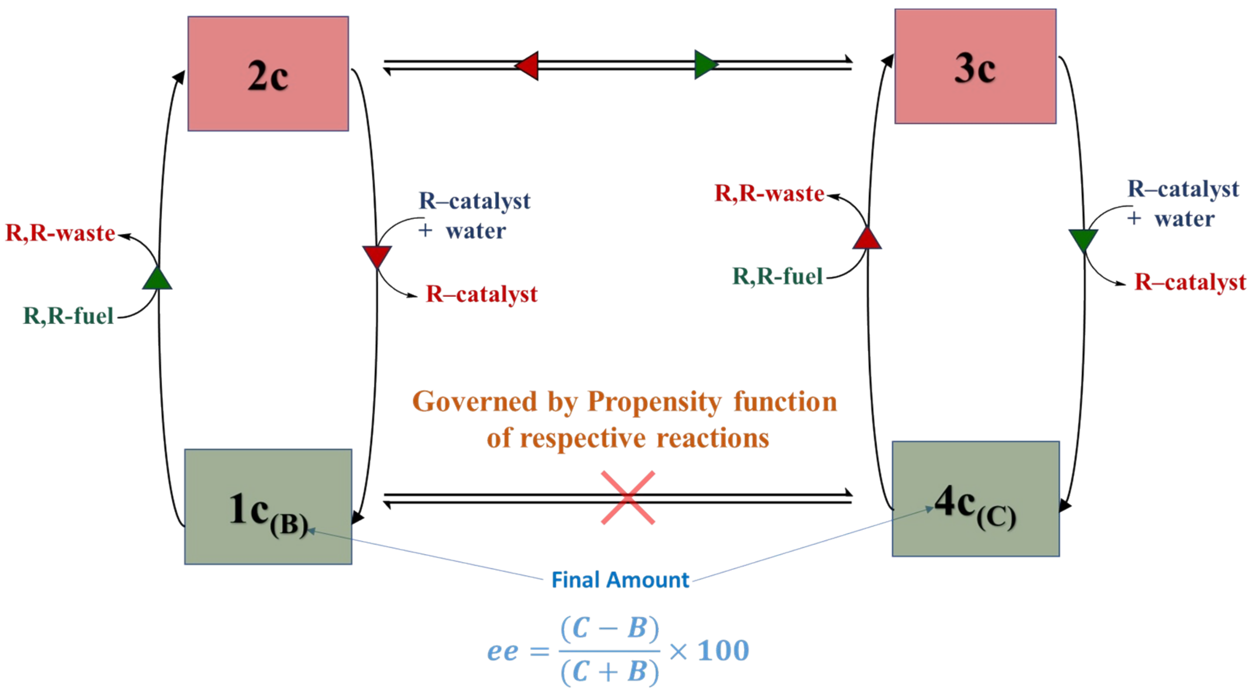

Figure S4. The simplified model system with reactions included when employing stochastic simulations for **1c** at at the PBE-D3/TZVP level (COSMO: ε = 50.28) of theory.

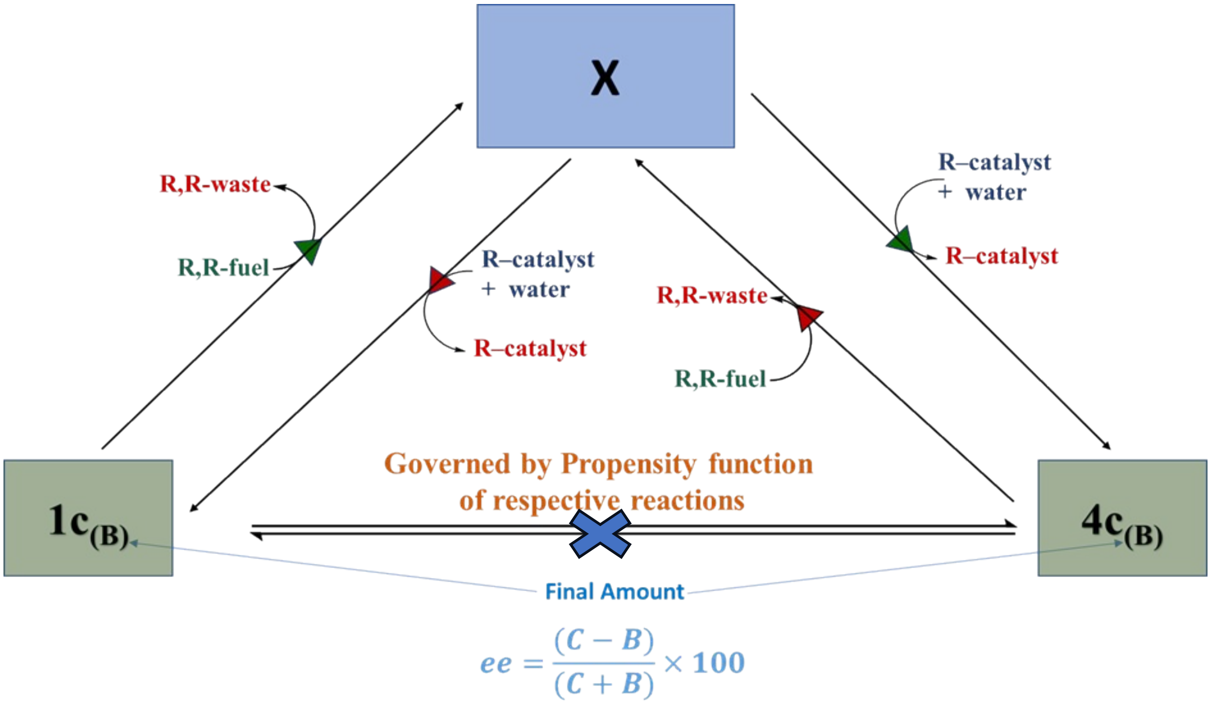

Figure S5. The simplified model system with reactions included when employed stochastic simulations for **1c** without including anhydride rotation reactions.

5. **% ee calculations by stochastic simulations for the 1c system without considering water effect.**

We ran the stochastic simulations (n=10 times) for the values of **ΔG‡** and **ΔΔG‡** determined from DFT calculations at the PBE-D3/TZVP (COSMO: ε=50.28) level of theory for the **1c** system. The values attached in Table S6 and simulation results are attached in Table S7 respectively.

Table S5. **Values of ΔΔE‡ (in kcal/mol) of different reactions calculated by DFT (Level of theory - PBE-D3/TZVP (COSMO: ε=50.28)) for the 1c system.**

S.no	Type of reaction	ΔE‡ (in kcal/mol)	ΔΔE‡ (in kcal/mol)
**1**	1c+R, R-fuel ➔ 2c + R, R waste	11.6	
**2**	4c+R, R-fuel ➔ 3c + R, R waste	11.7	**0.1**
**3**	3c + water + R-catalyst ➔ 4c + R-catalyst	8.5	
**4**	2c + water + R-catalyst ➔ 1c + R-catalyst	8.8	**0.3**
**5**	1c+DIC ➔ 2c + DIU	11.7	
**6**	4c+DIC ➔ 3c + DIU	11.7	**0.0**
**7**	3c + water + DMAP ➔ 4c + DMAP	8.8	
**8**	2c + water + DMAP ➔ 1c + DMAP	8.8	**0.0**
**9**	2c + water + S-catalyst ➔ 1c + S-catalyst	8.8	
**10**	3c + water + S-catalyst ➔ 4c +S-catalyst	8.5	**-0.3**

Table S6. Values of ΔΔG‡ (in kcal/mol) of different reactions calculated by DFT (level of theory - PBE-D3/TZVP (COSMO: ε=50.28)) for the 1c system.

S.no	Type of reaction	ΔG‡ (in kcal/mol)	ΔΔG‡ (in kcal/mol)
**1**	1c+R, R-fuel ➔ 2c + R, R waste	10.9	
**2**	4c+R, R-fuel ➔ 3c + R, R waste	11.3	**0.4**
**3**	3c + water + R-catalyst ➔ 4c + R-catalyst	6.9	
**4**	2c + water + R-catalyst ➔ 1c + R-catalyst	7.7	**0.8**
**5**	1c+DIC ➔ 2c + DIU	11.3	
**6**	4c+DIC ➔ 3c + DIU	11.3	**0.0**
**7**	3c + water + DMAP ➔ 4c + DMAP	7.7	
**8**	2c + water + DMAP ➔ 1c + DMAP	7.7	**0.0**
**9**	2c + water + S-catalyst ➔ 1c + S-catalyst	7.7	
**10**	3c + water + S-catalyst ➔ 4c +S-catalyst	6.9	**-0.8**

Table S7. Values of ΔG‡ (in kcal/mol) of different reactions calculated by DFT (level of theory - PBE-D3/TZVP (COSMO: ε=50.28)) and %ee calculated by stochastic simulation for the 1c system.

Fuel and Catalyst	1c reacting fuel ($\Delta G^{\ddagger}$ in kcal/mol)	4c reacting fuel ($\Delta G^{\ddagger}$ in kcal/mol)	3c reacting catalyst ($\Delta G^{\ddagger}$ in kcal/mol)	2c reacting catalyst ($\Delta G^{\ddagger}$ in kcal/mol)	ee (pred.) (in %)	ee (exp.) (in%)[12]
R,R- Fuel and R-Catalyst	10.9	11.3	6.9	7.7	78.6±0.7	41
DIC and R-Catalyst	11.3	11.3	6.9	7.7	60.7±1.2	35
DIC and S-Catalyst	11.3	11.3	7.7	6.9	-61.8±0.7	-35
DIC and DMAP	11.3	11.3	7.7	7.7	-0.2±1.3	0
R,R- Fuel and DMAP	10.9	11.3	7.7	7.7	34.6±0.9	7

Values in Table S7 are calculated by the code designed which is based on Figure S4

Table S8. Values of $\Delta E^{\ddagger}$ (in kcal/mol) of different reactions calculated by DFT (level of theory - PBE-D3/TZVP (COSMO: ε=50.28)) and %ee calculated by stochastic simulation for the 1c system without considering anhydride rotation in the simulations.

Fuel and Catalyst	1c reacting fuel ($\Delta E^{\ddagger}$ in kcal/mol)	4c reacting fuel ($\Delta E^{\ddagger}$ in kcal/mol)	3c reacting catalyst ($\Delta E^{\ddagger}$ in kcal/mol)	2c reacting catalyst ($\Delta E^{\ddagger}$ in kcal/mol)	ee (pred.) (in %)	ee (exp.) (in%)[12]
R,R- Fuel and R-Catalyst	11.6	11.7	8.5	8.8	33.8±1.1	41
DIC and R-Catalyst	11.7	11.7	8.5	8.8	25.9±0.9	35
DIC and S-Catalyst	11.7	11.7	8.8	8.5	-25.4±1.0	-35
DIC and DMAP	11.7	11.7	8.8	8.8	0.6±1.2	0
R,R- Fuel and DMAP	11.6	11.7	8.8	8.8	9.4±1.0	7

After performing full DFT calculations at the PBE-D3/TZVP level (COSMO: ε = 50.28), we compared the computed results with those obtained using a set of alternative DFT methods, including both hybrid and GGA functionals. Further DFT calculations were performed for the ethyl-substituted motor system using different combinations of functionals and basis sets for the reactions **1c-A** → **1c-B** and **4c-A** → **4c-B**, as these reactions determine the % ee when the chiral fuel (R, R-fuel) and the achiral DMAP catalyst are employed.

The different levels of theory employed are: PBE0-D3/TZVP, B3LYP-D3/TZVP, TPSSH-D3/TZVP, PBE-D3/def2-TZVP, PBE0-D3/def2-TZVP, and BLYP[6]-D3/TZVP. Solvent effects were also incorporated in the DFT calculations.

After obtaining the values of **ΔE‡** for reactions **1c-A** → **1c-B** and **4c-A** → **4c-B** (provided in Table S9), we designed the stochastic model as shown in Figure S6. The **ΔE‡** values for **DMAP** and water reacting with **2c** and **3c** were taken as 8.8 kcal/mol each. Rotation between **1c** → **4c** and *vice versa* is blocked. The %ee value for PBE/TZVP is taken from Table S8, as it was calculated using the same code employed for different levels of theory.

Stochastic simulations were performed (n = 10), and the % error associated with each level of theory—compared to experimental results—is shown in Figure S7. The formula for calculating the % error is provided below.

$$\%\ ee\ error = \frac{ee(pred)(in\ \%) - ee(exp.)(in\ \%)}{ee(exp.)(in\%)} \times 100$$

Table S9. Values of ΔE‡ (in kcal/mol) of reactions 1c-A➔ 1c-B and 4c-A➔ 4c-B calculated by DFT and the percentage error associated with different level of theories in % ee produced in R,R- fuel and DMAP case

Level of Theory	1c-A➔ 1c-B (ΔE‡ in kcal/mol)	4c-A➔ 4c-B (ΔE‡ in kcal/mol)	ee (exp.) (in%)[12]	ee (pred.) (in %)	% error associated with level of theory

**PBE/TZVP**	11.6	11.7	7	9.4±1.0	34
**PBE/def2-TZVP**	11.6	11.6	7	0.0±1.3	-100
**PBE0/def2-TZVP**	14.1	14.3	7	17.7±0.9	153
**TPSSH/TZVP**	12.6	13.2	7	49.9±1.1	613
**PBE0/TZVP**	13.9	14.5	7	49.9±1.1	613
**B-LYP/TZVP**	12.0	13.0	7	70.8±0.7	911
**B3LYP/TZVP**	13.9	15.4	7	87.1±0.8	1144

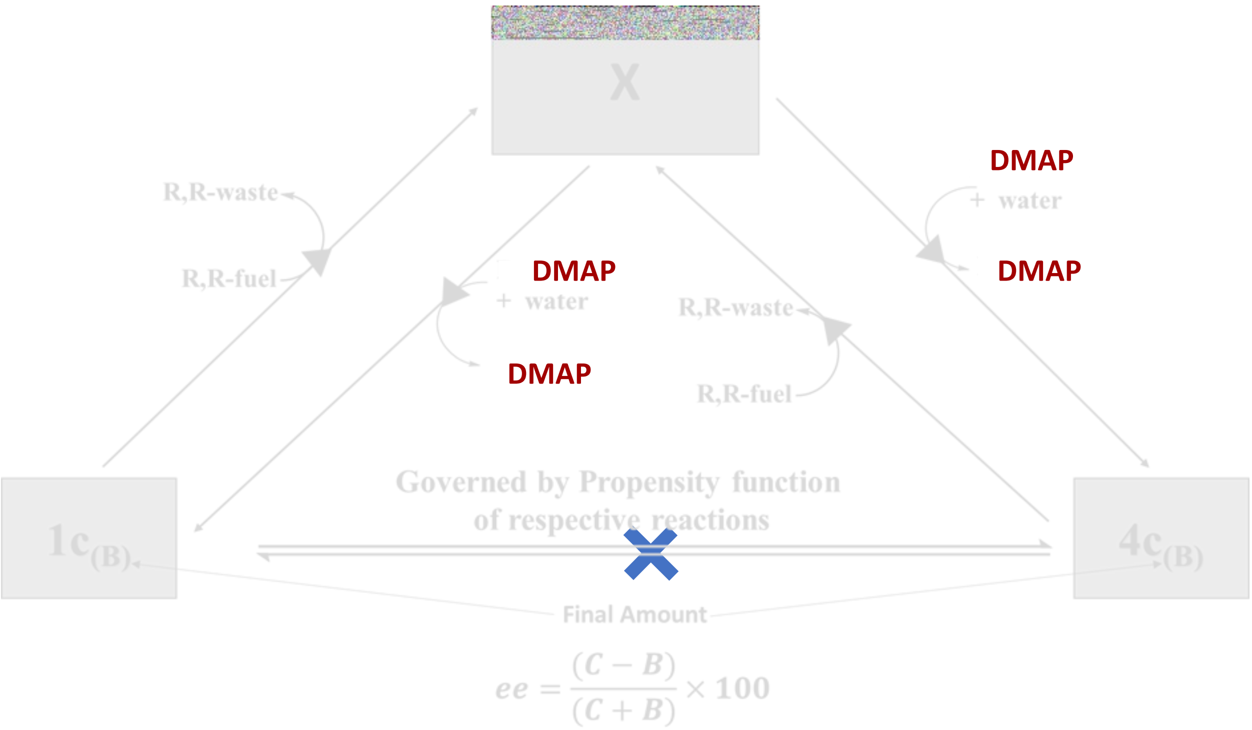

Figure S6. The simplified model system with reactions included when employed stochastic simulations for **1c** without including anhydride rotation reactions for different levels of theory.

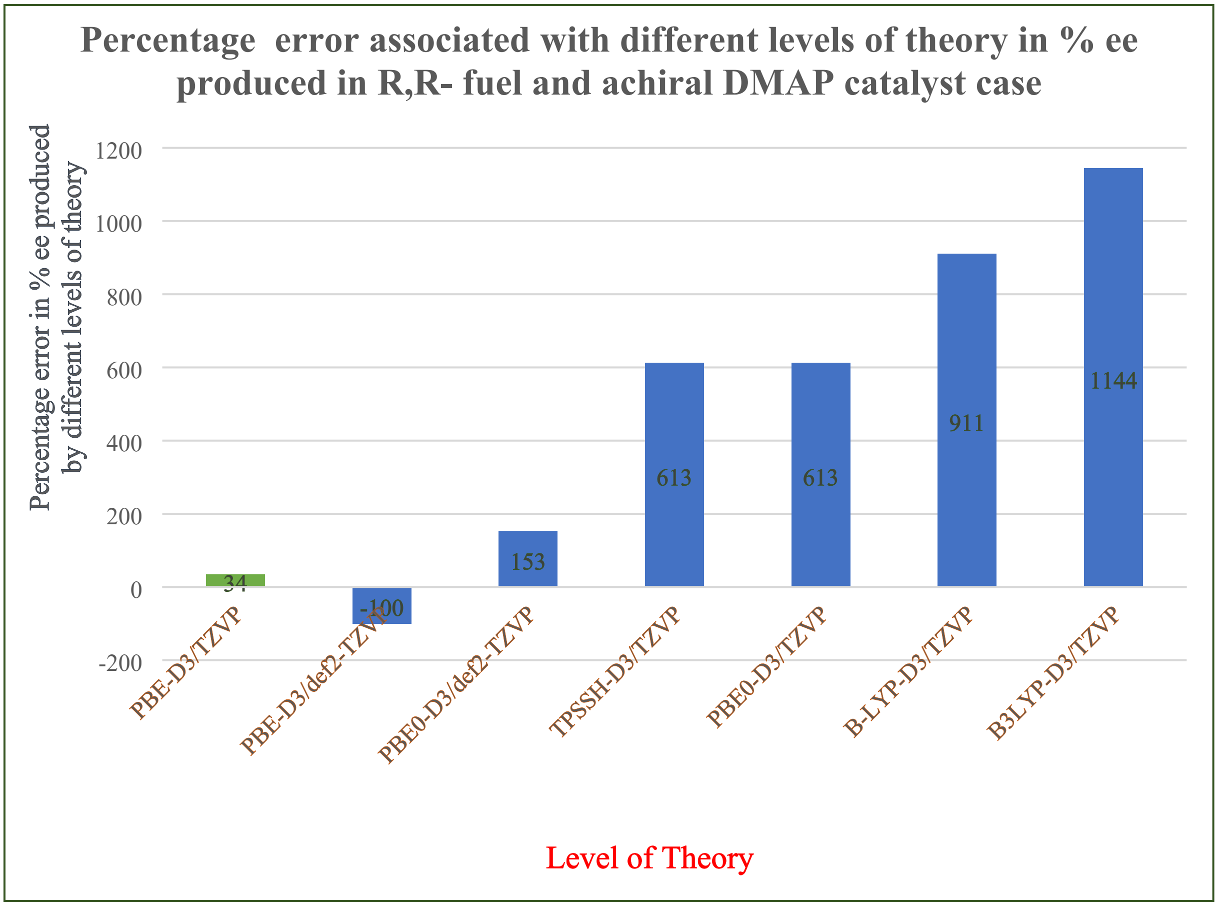

Figure S7. Percentage error associated with different levels of theory in % ee produced in the R,R- fuel and DMAP case.

The system exhibits notable sensitivity to the choice of functional and basis set, with computed energetics and selectivity varying across the tested methods. Among them, PBE-D3/TZVP provided the best agreement with experimental data due to cancellation of systematic errors, justifying its selection for all reported calculations.

We also ran the stochastic simulations (n=10 times) for the values of $\Delta E^{\ddagger}$, $\Delta\Delta E^{\ddagger}$ calculated by single point calculations at different level of theory for the **1c** system. Values and simulation results are attached in Table S10 -S14.

Table S10. Values of $\Delta E^{\ddagger}$ and $\Delta\Delta E^{\ddagger}$ (in kcal/mol) of different reactions calculated by DFT (level of theory: (B3-LYP-D3/def2-TZVP(COSMO:ε=50.28)//PBE-D3/TZVP (COSMO: ε=50.28); temperature =283.15K).

Type of reaction	$\Delta E^{\ddagger}$ (in kcal/mol)	$\Delta\Delta E^{\ddagger}$ (in kcal/mol)	$\Delta\Delta E^{\ddagger}_{anhydride\_rot}$ (in kcal/mol)	ee (pred.) (in %)	ee (exp.) (in%)[12]
1c+R, R-fuel → 2c + R, R waste	14.0		12.8	92.4±0.5	41
4c+R, R-fuel → 3c + R, R waste	14.9	**0.9**			
3c + water + R-catalyst → 4c + R-catalyst	12.8				
2c + water + R-catalyst → 1c + R-catalyst	13.7	**0.9**			

Table S11. Values of $\Delta E^{\ddagger}$ and $\Delta\Delta E^{\ddagger}$ (in kcal/mol) of different reactions calculated by DFT (level of theory: (M062X-D3//def2-TZVP(COSMO: ε=50.28)//PBE-D3/TZVP(COSMO: ε=50.28); temperature =283.15K).

Type of reaction	$\Delta E^{\ddagger}$ (in kcal/mol)	$\Delta\Delta E^{\ddagger}$ (in kcal/mol)	$\Delta\Delta E^{\ddagger}_{anhydride\_rot}$ (in kcal/mol)	ee (pred.) (in %)	ee (exp.) (in%)[12]
1c+R, R-fuel → 2c + R, R waste	15.6		13.3	99.5±0.2	41
4c+R, R-fuel → 3c + R, R waste	17.5	**1.9**			
3c + water + R-catalyst → 4c + R-catalyst	11.4				
2c + water + R-catalyst → 1c + R-catalyst	12.8	**1.4**			

Table S12. Values of $\Delta E^{\ddagger}$ and $\Delta\Delta E^{\ddagger}$ (in kcal/mol) of different set of reactions calculated by DFT (Level of theory – (PBE0-D3//def2-TZVP (COSMO: ε=50.28)//PBE-D3/TZVP(COSMO: ε=50.28); temperature: 283.15K).

Type of reaction	$\Delta E^{\ddagger}$ (in kcal/mol)	$\Delta\Delta E^{\ddagger}$ (in kcal/mol)	$\Delta\Delta E^{\ddagger}_{anhydride\_rot}$ (in kcal/mol)	ee (pred.) (in %)	ee (exp.) (in%)[12]
1c+R, R-fuel → 2c + R, R waste	13.9		12.2	78.7±0.9	41
4c+R, R-fuel → 3c + R, R waste	14.6	**0.7**			

3c + water + R-catalyst ➔ 4c + R-catalyst	11.3				
2c + water + R-catalyst ➔ 1c + R-catalyst	11.8	**0.5**			

Table S13. Values of ΔE‡ and ΔΔE‡ (in kcal/mol) of different set of reactions calculated by DFT (level of theory: (TPSSH-D3//def2-TZVP (COSMO: ε=50.28)//PBE-D3/TZVP (COSMO: ε=50.28), temperature =283.15K).

**Type of reaction**	**ΔE‡ (in kcal/mol)**	**ΔΔE‡ (in kcal/mol)**	**$\Delta\Delta E^{\ddagger}_{anhydride\_rot}$ (in kcal/mol)**	**ee (pred.) (in %)**	**ee (exp.) (in%)[12]**
1c+R, R-fuel ➔ 2c + R, R waste	12.9		11.7	67.4±1.2	41
4c+R, R-fuel ➔ 3c + R, R waste	13.2	**0.3**			
3c + water + R-catalyst ➔ 4c + R-catalyst	10.8				
2c + water + R-catalyst ➔ 1c + R-catalyst	11.4	**0.6**			

Table S14. Values of ΔE‡ and ΔΔE‡ (in kcal/mol) of different set of reactions calculated by DFT (level of theory(WB97X-D-D3//def2-TZVP (COSMO: ε=50.28)//PBE-D3/TZVP (COSMO: ε=50.28), temperature: 283.15K).

**Type of reaction**	**ΔE‡ (in kcal/mol)**	**ΔΔE‡ (in kcal/mol)**	**$\Delta\Delta E^{\ddagger}_{anhydride\_rot}$ (in kcal/mol)**	**ee (pred.) (in %)**	**ee (exp.) (in%)[12]**
1c+R, R-fuel ➔ 2c + R, R waste	14.1		13.8	92.4±0.6	41
4c+R, R-fuel ➔ 3c + R, R waste	15.3	**1.2**			
3c + water + R-catalyst ➔ 4c + R-catalyst	13.2				
2c + water + R-catalyst ➔ 1c + R-catalyst	13.9	**0.7**			

6. **Stochastic simulations for 1b and % ee calculations for 1c with water effect.**

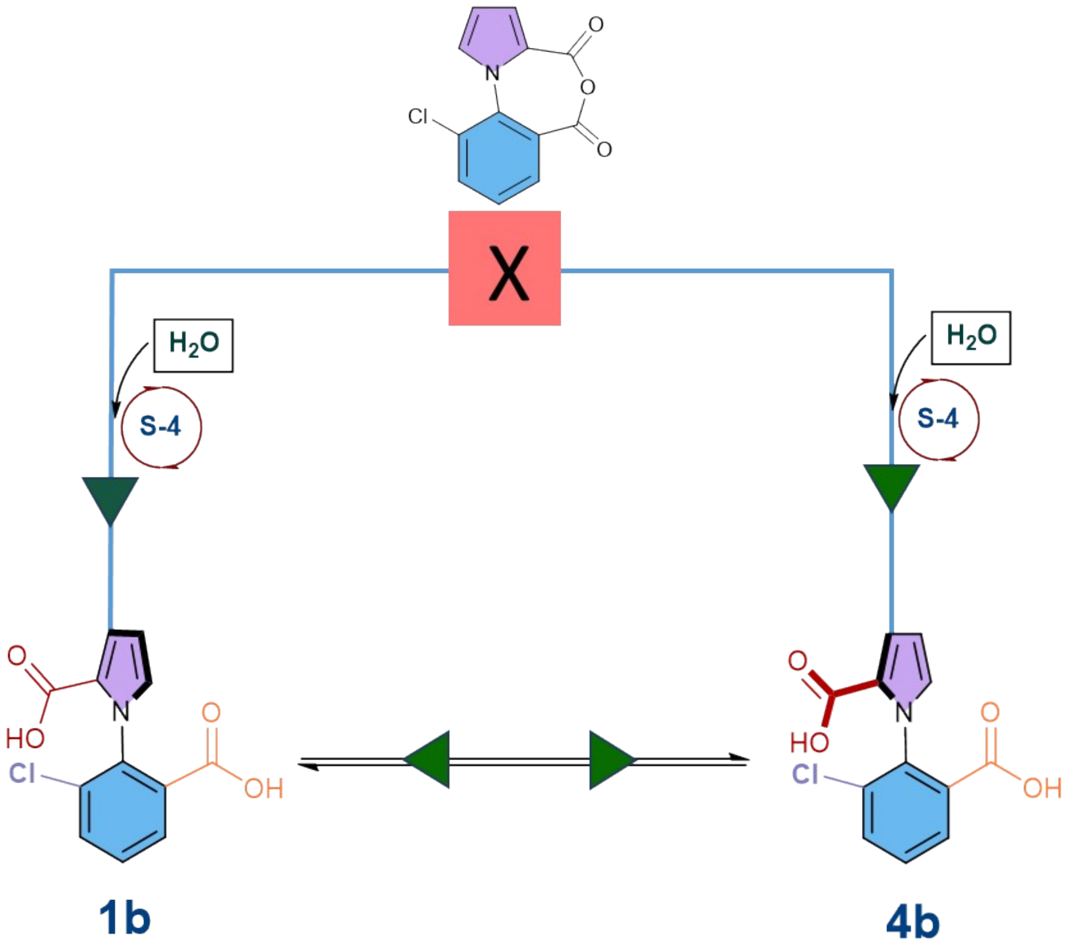

Rxn1 = 1b➔ 4b
Rxn2 = 4b➔ 1b
Rxn3 = 3b + Water +R-catalyst ➔ 4b+ R-catalyst
Rxn4 = 2b + Water +R-catalyst ➔ 1b+ R-catalyst

Figure S8. The simplified model system with reactions included when employing stochastic simulations for **1b**.

As shown in Figure S8 ,The interconversion between the two atropoisomeric conformations of the anhydride, **2b** and **3b**, would be very rapid, and therefore, **2b** and **3b** were replaced by a single entity, **X**. This was done because, at any point of time, the concentrations of **2b** and **3b** would quickly equilibrate to the same value. So the concentration of **2b** at any point during the stochastic simulations was taken to be **X/2**, and the same concentration was given to **3b**, to represent the rapid equilibration step, and thus to save time and computational expense, without affecting the accuracy of the silumations, as had been demonstrated for the ethyl case **1c**. We ran the code for n = 100 simulations.

We modified the code used in the 1b case slightly. Instead of 4 reactions, 6 reactions were considered in the **1c** case to study the dynamics of the ethyl system in the presence of water, as presented in Table S15. The six reactions included in the code are as follows: two reactions—**1c** and **4c**—with the fuel, treated as single-step reactions as before; two reactions—conversion of **1c** to **4c** and vice versa; and two reactions—**2c** and **3c**—with the catalyst, also treated as single-step reactions, where the concentrations of **2c** and **3c** at any time are taken to be X/2. In summary, six reactions were incorporated in this approach.

Table S15. Values of $\Delta E^{\ddagger}$ (in kcal/mol) of different reactions calculated by DFT (level of theory - PBE-D3/TZVP (COSMO: ε=50.28)) and %ee calculated by stochastic simulation for the 1c system without considering anhydride rotation in the simulations but considering water effect.($\Delta E^{\ddagger}_{rot(with\ water\ effect)}$ = 19.5 kcal/mol and we ran the code for n = 10 simulations.)

Fuel and Catalyst	1c reacting fuel ($\Delta E^{\ddagger}$ in kcal/mol)	4c reacting fuel ($\Delta E^{\ddagger}$ in kcal/mol)	3c reacting catalyst ($\Delta E^{\ddagger}$ in kcal/mol)	2c reacting catalyst ($\Delta E^{\ddagger}$ in kcal/mol)	ee (pred.) (in %)	ee (exp.) (in%)[12]
R,R- Fuel and R- Catalyst	11.6	11.7	8.5	8.8	34.6±0.9	41
DIC and R- Catalyst	11.7	11.7	8.5	8.8	25.6±1.0	35
DIC and S- Catalyst	11.7	11.7	8.8	8.5	-24.6±1.0	-35
DIC and DMAP	11.7	11.7	8.8	8.8	0.9±1.2	0
R,R- Fuel and DMAP	11.6	11.7	8.8	8.8	9.1±0.8	7

7. Free energy profile diagram for 360° rotation for 1a and 4a.

Starting with the clockwise direction, (Figure S9) **1a** reacts with fuel to form an adduct: **1a-A**, which is 8.8 kcal/mol more stable than the initial species. **1a-A** converts into **1a-B** *via* a 6-membered transition state ($\Delta G^{\ddagger}$ = 10.6 kcal/mol). Subsequently, **1a-B** transforms into the third intermediate, **1a-C**, through a simple intramolecular proton transfer ($\Delta G^{\ddagger}$ = 1.2 kcal/mol). Finally, **1a-C** converts into **2a** through ring-closing, with a barrier ($\Delta G^{\ddagger}$) of 4.5 kcal/mol.

In case of anticlockwise direction, **4a** reacts with fuel to form an adduct **4a-A**, which is 9.0 kcal/mol more stable than the initial species. **4a-A** converts into **4a-B** *via* a 6-membered transition state ($\Delta G^{\ddagger}$ = 11.1 kcal/mol). Subsequently, **4a-B** transforms into the third intermediate, **4a-C**, through a simple intramolecular proton transfer ($\Delta G^{\ddagger}$ = -1.3 kcal/mol). Finally, **4a-C** converts into **3a** through ring-closing, with a barrier ($\Delta G^{\ddagger}$) of 5.4 kcal/mol.

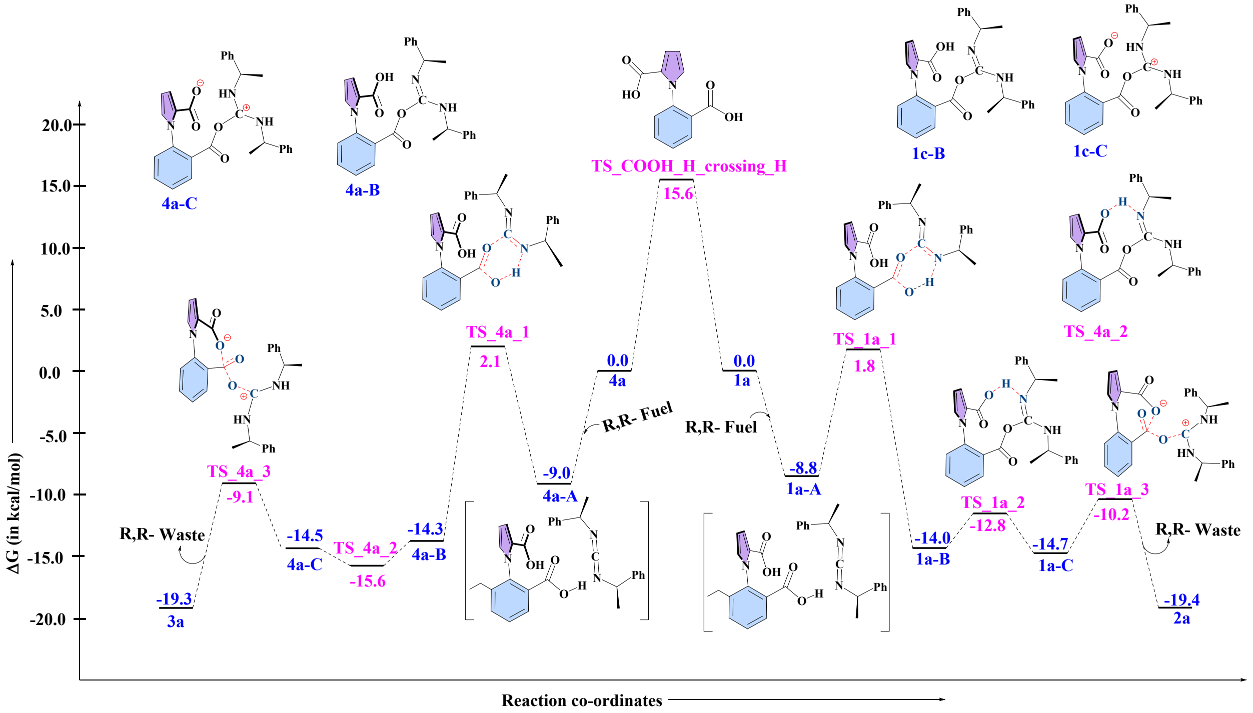

Figure S9. The free energy profile diagram of **1a**/**4a** reacting with R,R – Fuel to form **2a**/**3a**. Level of theory: PBE-D3/TZVP (COSMO: ε=50.28); temperature: 283.15 K.

For the clockwise rotation, (Figure S10) the conversion of **2a** to **3a** involves a rotation, with a barrier of 4.1 kcal/mol. Subsequently, **3a** forms an adduct **3a-A**, with the R-catalyst which is 3.1

kcal/mol more stable than the initial species. **3a-A** undergoes a ring-opening reaction in the presence of the R- catalyst to produce the intermediate, **3a-B**, with a barrier (ΔG‡) of 3.5 kcal/mol. **3a-B** reacts with water to form intermediate **3a-C**, which is 5.3 kcal/mol more stable than water and **3a-B**. **3a-C** converts into **4a** and regenerates the **R-4** catalyst, with a barrier (ΔG‡) of 6.9 kcal/mol. Finally, **4a** transforms into **1a** through a simple C-N bond rotation, with a barrier (ΔG‡) of 15.6 kcal/mol to complete the 360° rotation.

For the anticlockwise rotation, **3a** converts into **2a** with a barrier of 4.1 kcal/mol. Subsequently, **2a** forms an adduct **2a-A** with R-catalyst, which is 4.6 kcal/mol more stable than the initial species. **2a-A** undergoes a ring-opening reaction in the presence of the R-catalyst to produce the intermediate **2a-B**, with a barrier (ΔG‡) of 4.2 kcal/mol. **2a-B** reacts with water to form intermediate **2a-C**, which is 4.3 kcal/mol more stable than water and **2a-B**. **2a-C** converts into **1a** and regenerates the **R-4** catalyst, with a barrier (ΔG‡) of 7.3 kcal/mol. Finally, **1a** transforms into **4a** through a simple C-N bond rotation, with a barrier (ΔG‡) of 15.6 kcal/mol to complete the 360° rotation.

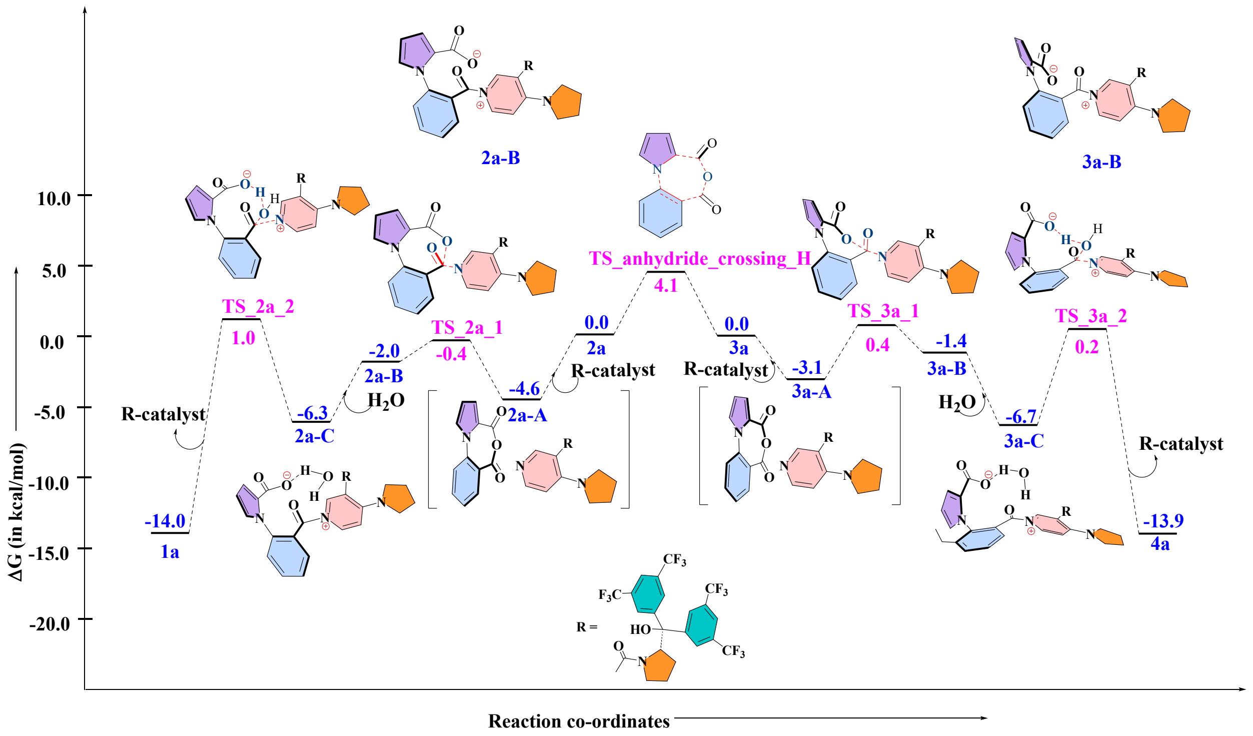

Figure S10. The Free energy profile diagram of **3a**/**2a** reacting with R,R – Fuel to form **4a**/**1a**. Level of theory: PBE-D3/TZVP (COSMO: ε=50.28); temperature = 283.15 K.

8. Single point calculations for 360° rotation cycle of 1a and 4a.

After performing full DFT calculations at the PBE-D3/TZVP level (COSMO: ε = 50.28), we conducted single-point calculations for **1a** motor system using the def2-TZVP basis set and five different levels of theory: B3LYP, M06-2X, PBE0, TPSSH, and WB97X-D. The results are provided below in Tables S16-19. Solvent effects and dispersion corrections (D3) were also incorporated in the single-point calculations.

Table S16. Thermodynamic and kinetic parameters for the conversion of 1a to 2a with the R,R-fuel.

S.no	Level of theory	R,R-fuel +1a ➔ 1a-A	1a-A➔ 1a-B		1a-B➔ 1a-C		1a-C➔ 2a +R,R-waste		(For single step)
		ΔE	ΔE‡	ΔE	ΔE‡	ΔE	ΔE‡	ΔE	ΔE‡
**1**	PBE-D3/TZVP (COSMO: ε=50.28),	**-12.2**	**11.2**	**-5.8**	**2.5**	**-1.4**	**4.1**	**-1.7**	**11.2**
**2**	B3LYP-D3/def2-TZVP (COSMO: ε=50.28) //PBE-D3/TZVP (COSMO: ε=50.28)	**-11.2**	**13.8**	**-4.0**	**2.8**	**-2.4**	**7.0**	**-2.4**	**13.8**
**3**	M06-2x-D3/def2-TZVP (COSMO: ε=50.28) //PBE-D3/TZVP (COSMO: ε=50.28)	**-9.4**	**15.8**	**-10.4**	**3.3**	**-0.9**	**7.7**	**-0.7**	**15.8**
**4**	PBE0-D3/def2-TZVP (COSMO: ε=50.28) //PBE-D3/TZVP (COSMO: ε=50.28)	**-11.2**	**13.6**	**-9.8**	**2.9**	**-1.7**	**5.6**	**-2.8**	**13.6**
**5**	TPSSH-D3/def2-TZVP (COSMO: ε=50.28) //PBE-D3/TZVP (COSMO:	**-10.6**	**12.5**	**-6.8**	**2.5**	**-1.9**	**4.6**	**-3.0**	**12.5**

	ε=50.28)								
**6**	WB97X-D-D3/def2-TZVP (COSMO: ε=50.28) //PBE-D3/TZVP (COSMO: ε=50.28)	**-9.1**	**13.2**	**-9.7**	**3.0**	**-2.6**	**7.5**	**-3.1**	**13.2**

Table S17. Thermodynamic and kinetic parameters for the conversion of 4a to 3a with the R,R-fuel.

**S.no**	**Level of theory**	**R,R-fuel +4a → 4a-A**	**4a-A→ 4a-B**		**4a-B→ 4a-C**		**4a-C→ 3a +R,R-waste**		**(For single step)**
		**ΔE**	**ΔE‡**	**ΔE**	**ΔE‡**	**ΔE**	**ΔE‡**	**ΔE**	**ΔE‡**
**1**	PBE-D3/TZVP (COSMO: ε=50.28),	**-12.3**	**11.3**	**-6.1**	**1.4**	**-1.1**	**5.3**	**-1.6**	**11.3**
**2**	B3LYP-D3/def2-TZVP (COSMO: ε=50.28) //PBE-D3/TZVP (COSMO: ε=50.28)	**-11.6**	**14.7**	**-4.3**	**2.8**	**-1.8**	**9.0**	**-2.4**	**14.7**
**3**	M06-2x-D3/def2-TZVP (COSMO: ε=50.28) //PBE-D3/TZVP (COSMO: ε=50.28)	**-9.5**	**16.6**	**-9.6**	**2.7**	**-1.8**	**8.0**	**-0.5**	**16.6**
**4**	PBE0-D3/def2-TZVP (COSMO: ε=50.28) //PBE-D3/TZVP (COSMO: ε=50.28)	**-11.7**	**14.4**	**-9.8**	**2.0**	**-1.3**	**5.7**	**-2.7**	**14.4**
**5**	TPSSH-D3/def2-TZVP (COSMO: ε=50.28) //PBE-D3/TZVP (COSMO: ε=50.28)	**-11.0**	**13.1**	**-6.8**	**2.5**	**-1.6**	**5.7**	**-2.9**	**13.1**

**6**	WB97X-D-D3/def2-TZVP (COSMO: ε=50.28) //PBE-D3/TZVP (COSMO: ε=50.28)	**-9.8**	**14.7**	**-9.4**	**3.2**	**-2.3**	**8.6**	**-2.9**	**14.7**

Table S18. Thermodynamic and kinetic parameters for the conversion of 3a to 4a with the R-catalyst and water.

**S.no**	**Level of theory**	**R-catalyst +3a ➔ 3a-A**	**3a-A➔ 3a-B**		**3a-B+ water ➔ 3a-C**	**3a-C➔ 4a + R-catalyst**		**(For single step)**
		**ΔE**	**ΔE‡**	**ΔE**	**ΔE**	**ΔE‡**	**ΔE**	**ΔE‡**
**1**	PBE-D3/TZVP (COSMO: ε=50.28),	**-6.3**	**3.4**	**0.6**	**-8.4**	**8.6**	**-3.7**	**8.6**
**2**	B3LYP-D3/def2-TZVP (COSMO: ε=50.28) //PBE-D3/TZVP (COSMO: ε=50.28)	**-6.6**	**8.3**	**3.9**	**-9.8**	**12.9**	**-5.9**	**12.9**
**3**	M06-2x-D3/def2-TZVP (COSMO: ε=50.28) //PBE-D3/TZVP (COSMO: ε=50.28)	**-7.0**	**7.1**	**4.9**	**-9.6**	**12.2**	**-8.4**	**12.2**
**4**	PBE0-D3/def2-TZVP (COSMO: ε=50.28) //PBE-D3/TZVP (COSMO: ε=50.28)	**-6.2**	**6.0**	**3.8**	**-9.0**	**11.4**	**-7.6**	**11.4**
**5**	TPSSH-D3/def2-TZVP (COSMO: ε=50.28) //PBE-D3/TZVP (COSMO: ε=50.28)	**-5.8**	**6.1**	**3.6**	**7.9**	**11.0**	**7.4**	**11.0**

6	WB97X-D-D3/def2-TZVP (COSMO: ε=50.28) //PBE-D3/TZVP (COSMO: ε=50.28)	-6.3	8.3	5.0	-9.5	13.4	-8.1	13.4

Table S19. Thermodynamic and kinetic parameters for the conversion of 2a to 1a with the R-catalyst and water.

**S.no**	**Level of theory**	**R-catalyst +2a ➔ 2a-A**	**2a-A➔ 2a-B**		**2a-B+ water ➔ 2a-C**	**2a-C➔ 1a + R-catalyst**		**(For single step)**
		**ΔE**	**ΔE‡**	**ΔE**	**ΔE**	**ΔE‡**	**ΔE**	**ΔE‡**
**1**	PBE-D3/TZVP (COSMO: ε=50.28),	**-7.8**	**4.2**	**1.5**	**-7.5**	**9.1**	**-3.9**	**9.1**
**2**	B3LYP-D3/def2-TZVP (COSMO: ε=50.28) //PBE-D3/TZVP (COSMO: ε=50.28)	**-8.0**	**9.0**	**3.9**	**-9.1**	**14.7**	**-5.2**	**14.7**
**3**	M06-2x-D3/def2-TZVP (COSMO: ε=50.28) //PBE-D3/TZVP (COSMO: ε=50.28)	**-7.9**	**7.0**	**3.8**	**-9.2**	**15.5**	**-7.0**	**15.5**
**4**	PBE0-D3/def2-TZVP (COSMO: ε=50.28) //PBE-D3/TZVP (COSMO: ε=50.28)	**-7.6**	**6.7**	**4.0**	**-7.8**	**12.3**	**-7.5**	**12.3**
**5**	TPSSH-D3/def2-TZVP (COSMO: ε=50.28) //PBE-D3/TZVP (COSMO: ε=50.28)	**-7.2**	**6.8**	**3.9**	**-6.8**	**12.0**	**-7.5**	**12.0**

**6**	WB97X-D-D3/def2-TZVP (COSMO: ε=50.28) //PBE-D3/TZVP (COSMO: ε=50.28)	**-7.4**	**8.0**	**3.9**	**-7.8**	**14.3**	**-7.7**	**14.3**

9. Stochastic simulations for the 1a system, with the effect of water included.

As Shown in Figure S11, the interconversion between the two atropoisomeric conformations of the anhydride, **2a** and **3a**, was seen to be very rapid, and therefore, **2a** and **3a** were replaced by a single entity, **X**. This was done because, at any point of time, the concentrations of **2a** and **3a** would quickly equilibrate to the same value. So the concentration of **2a** at any point during the stochastic simulations was taken to be **X/2**, and the same concentration was given to **3a**, to represent the rapid equilibration step, and thus to save time and computational expense, without affecting the accuracy of the silumations, as had been demonstrated for the ethyl case **1c**. Importantly, a replacement similar to **X** was done for the species **1a** and **4a**. This is because the barrier for the interconversion between **1a** and **4a** had been determined to be only 0.5 kcal/mol, after the effect of water had been included (see Section (iii) of Results and Discussions in the main manuscript). Hence, as for the anyhdride atropoisomers, **1a** and **4a** will also rapidly equilibrate, and have the same concentration at any point of time during the stochastic simulations. Code was run for n=100 simulations.

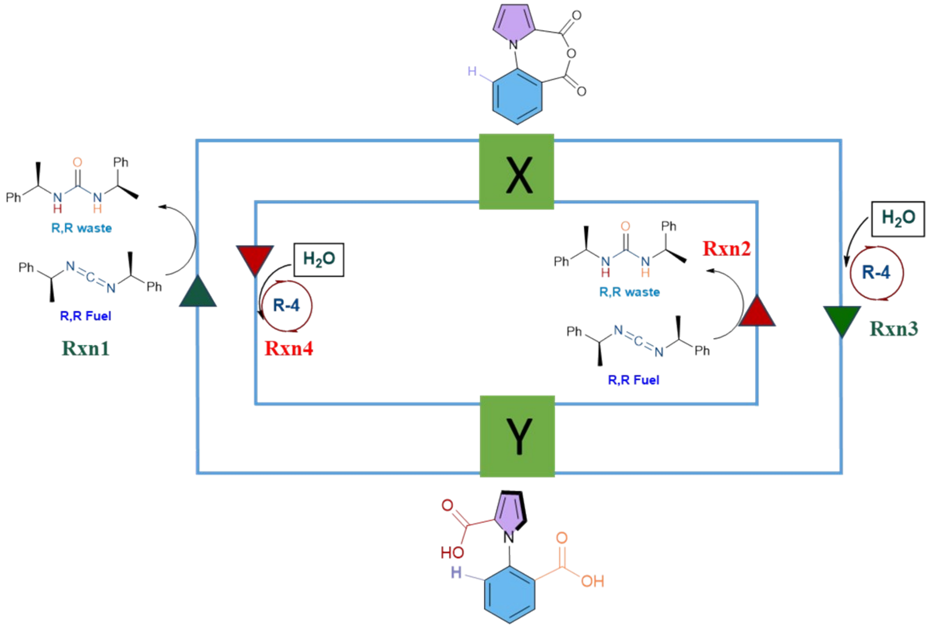

Rxn1 = 1a+ R,R fuel ➔ 2a
Rxn2 = 4a+ R,R fuel ➔ 3a
Rxn3 = 3a + Water +R-catalyst ➔ 4a+ R-catalyst
Rxn4 = 2a + Water +R-catalyst ➔ 1a+ R-catalyst

Figure S11. The simplified model system with reactions included when employing stochastic simulations for **1a**. The effect of water is included.

After the simulation we calculated the number of every reaction fired in every simulation on an average and calculate the maximum directional efficiency(MDE) by this formula

MDE(forward)=$\frac{a}{a+b} * 100$

MDE(reverse)=$\frac{b}{a+b} * 100$

where a = **1a**+R,R-Fuel ➔**2a** +R,R-waste

b= **2a**+R-catalyst+water ➔**1a** +R-catalyst+water

10. Stochastic simulations for the 1a system, without the effect of water.

1a system is simulted with the help of stochastic simulations as shown in Figure S12 . Stochastic simulations with the Gillespie algorithm have been done in order to determine the behaviour of the molecular motor in the absence of the lubricating effect of water.

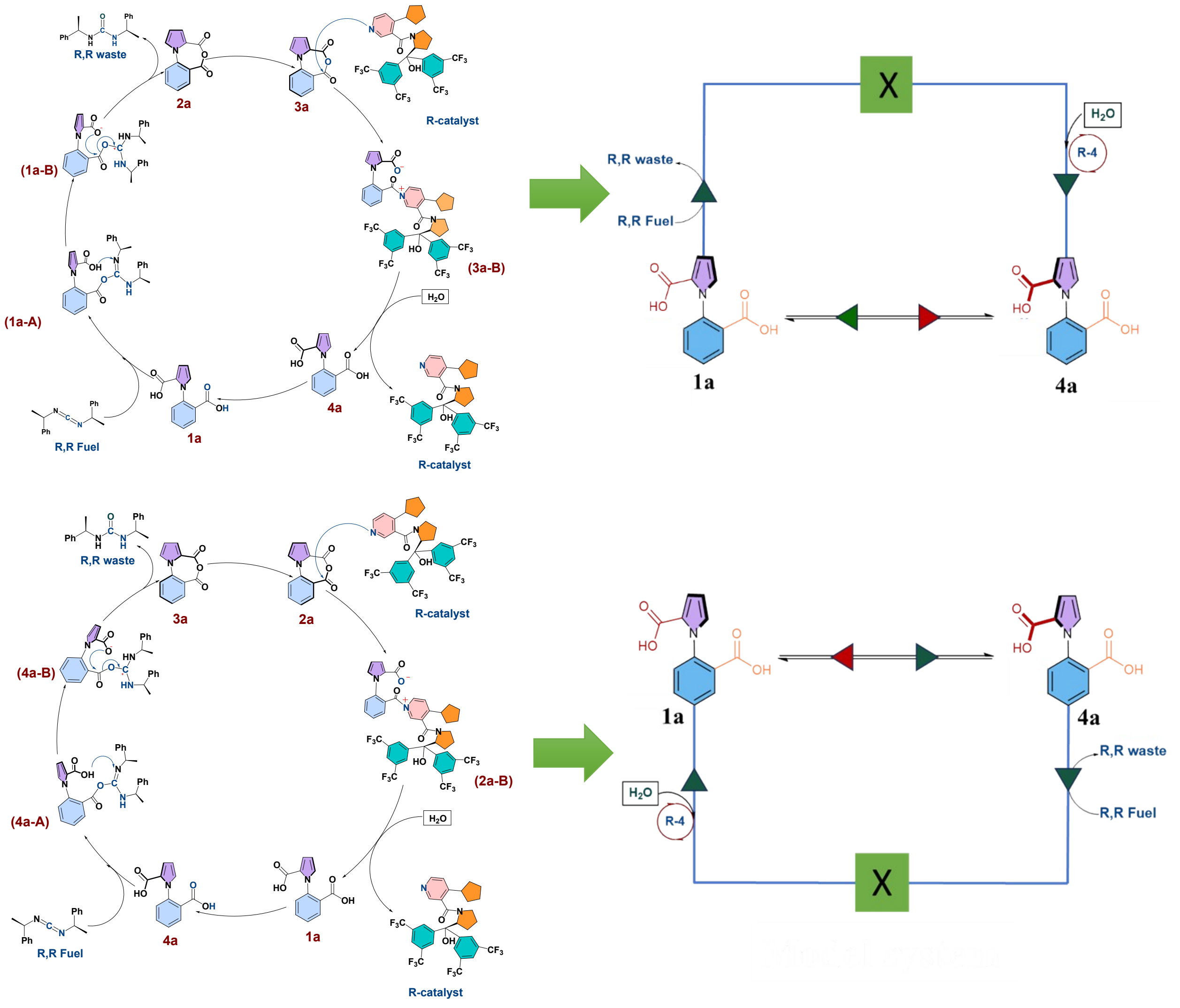

Original System **Model System**

Figure S12. The simplified model system employed for stochastic simulations of **1a**.

The difference in the number of reactions **4a-1a** and **1a-4a** rotation is very small for almost the entire fuel concentration range. It is only when the concentration of the fuel becomes very low that

the **4a-1a** rotation reaction begins to dominate. What this means is that, for almost all of the fuel concentration range, the molecular motor would have consumed the fuel, and used the chemical energy thus gained to only travel from **1a-2a-3a-4a** and then back from **4a-3a-2a-1a**, without completing the 360° rotation. Simulation results are shown in Figure S13, which is based on the values presented in Table S20.The reason for this is that the introduction of the two rotation reactions creates an issue of competition between the bimolecular and unimolecular reactions. In order for the system to successfully complete 360° rotation, it must choose the bimolecular **1a-2a** reaction over unimolecular **1a-4a** rotation and in the same cycle, then choose unimolecular **4a-1a** over bimolecular **4a-3a**. Since the difference in the barriers between the two bimolecular reactions is very small, and the barriers for the two unimolecular reactions is exactly the same, this is very unlikely. The more likely outcome is the choice of the bimolecular reactions over the unimolecular when the concentration of the fuel is not negligible. Therefore, it is only when the fuel concentration becomes very low that the completion of the cycle becomes feasible, as seen from the stochastic simulations. Fortunately, the water effect nullifies this competition between the uni- and bimolecular reactions, thereby making the kinetic gating steps effective.

Table S20. Values of $\Delta E^{\ddagger}$ and $\Delta\Delta E^{\ddagger}$ (in kcal/mol) of different sets of reactions calculated by DFT (PBE-D3/TZVP (COSMO: ε=50.28), as well as the information of the rotation of the motor gained from the Gillespie algorithm without incorporating water effect.

Type of reaction	$\Delta E^{\ddagger}$ (in kcal/mol)	$\Delta\Delta E^{\ddagger}$ (in kcal/mol)	$\Delta E^{\ddagger}_{rot}$ (in kcal/mol)	4a→1a rotation	1a→4a rotation	Net 4a→1a rotation
**1a**+R, R-fuel ➔ **2a** + R, R waste	11.2	0.1	15.6	1415	977	438
**4a**+R, R-fuel ➔ **3a** + R, R waste	11.3					
**3a** + water + R-catalyst ➔ **4a** + R-catalyst	8.6	0.5				
**2a** + water + R-catalyst ➔ **1a** + R-catalyst	9.1					

a.

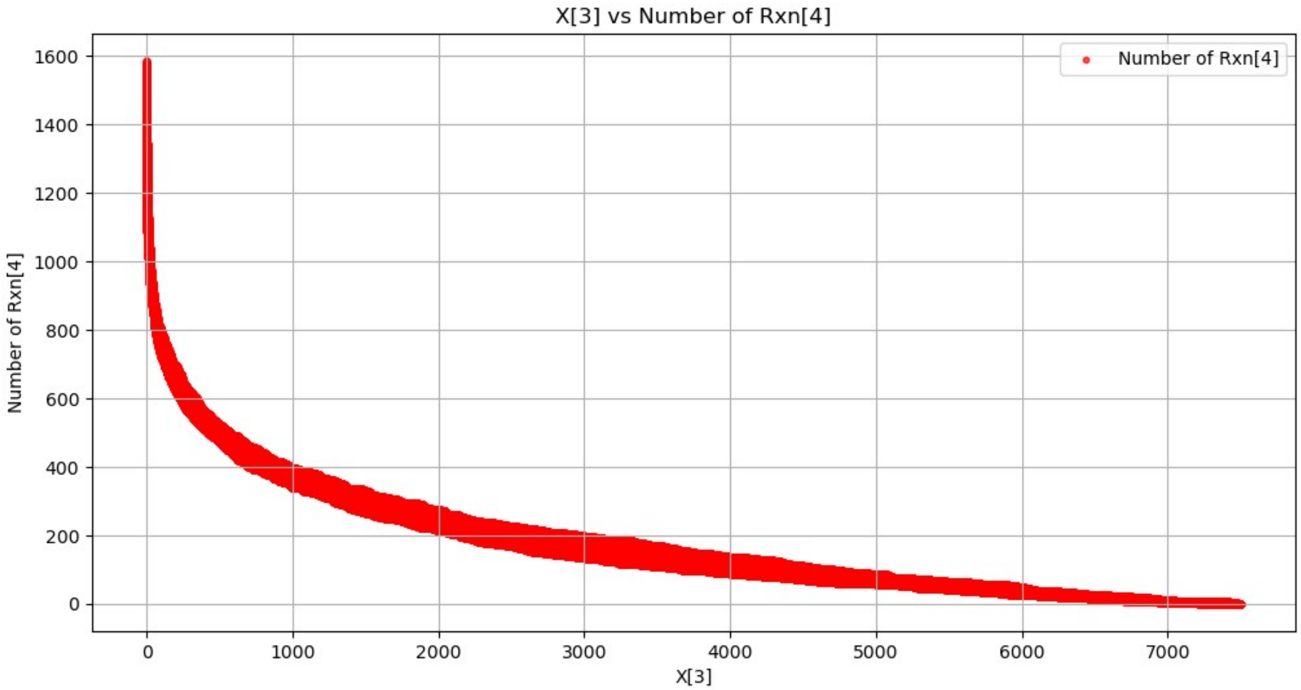
X[3] vs Number of Rxn[4]
Number of Rxn[4]
1600
1400
1200
1000
800
600
400
200
0
Number of Rxn[4]
0
1000
2000
3000
4000
5000
6000
7000
X[3]

b.

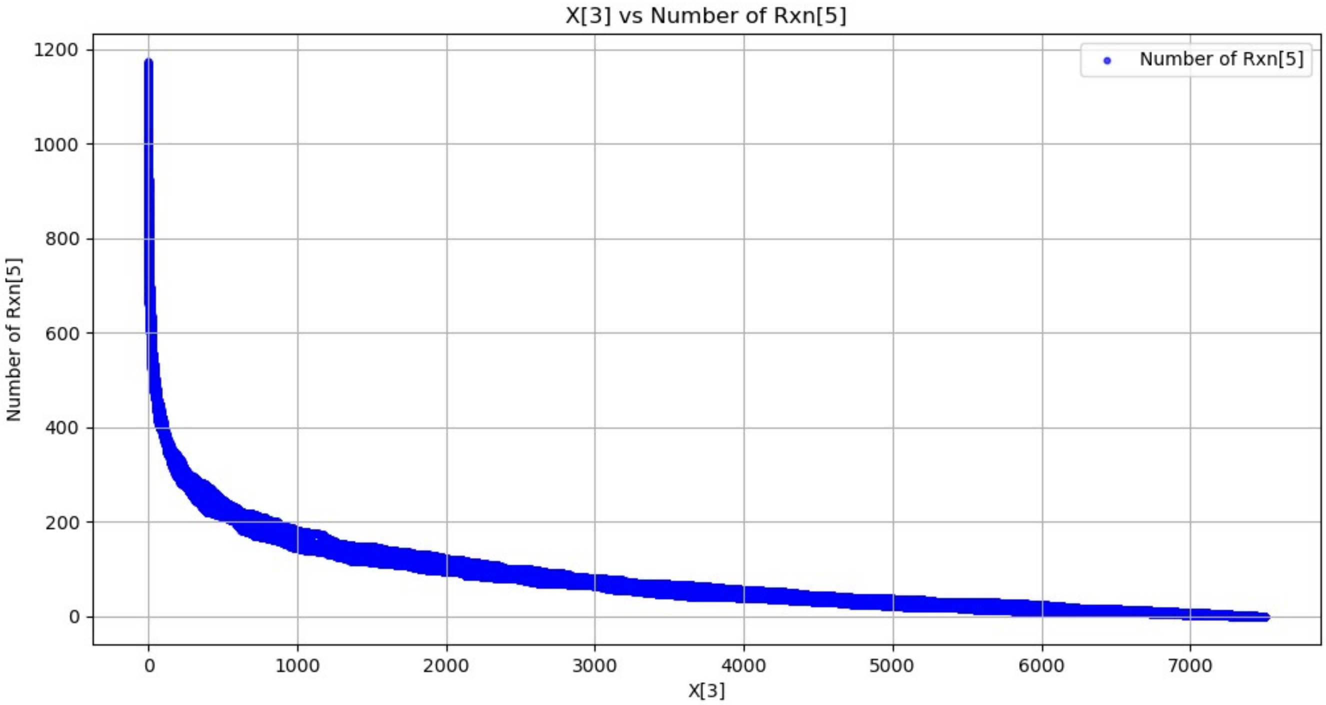
X[3] vs Number of Rxn[5]
Number of Rxn[5]
1200
1000
800
600
400
200
0
Number of Rxn[5]
0
1000
2000
3000
4000
5000
6000
7000
X[3]

c.

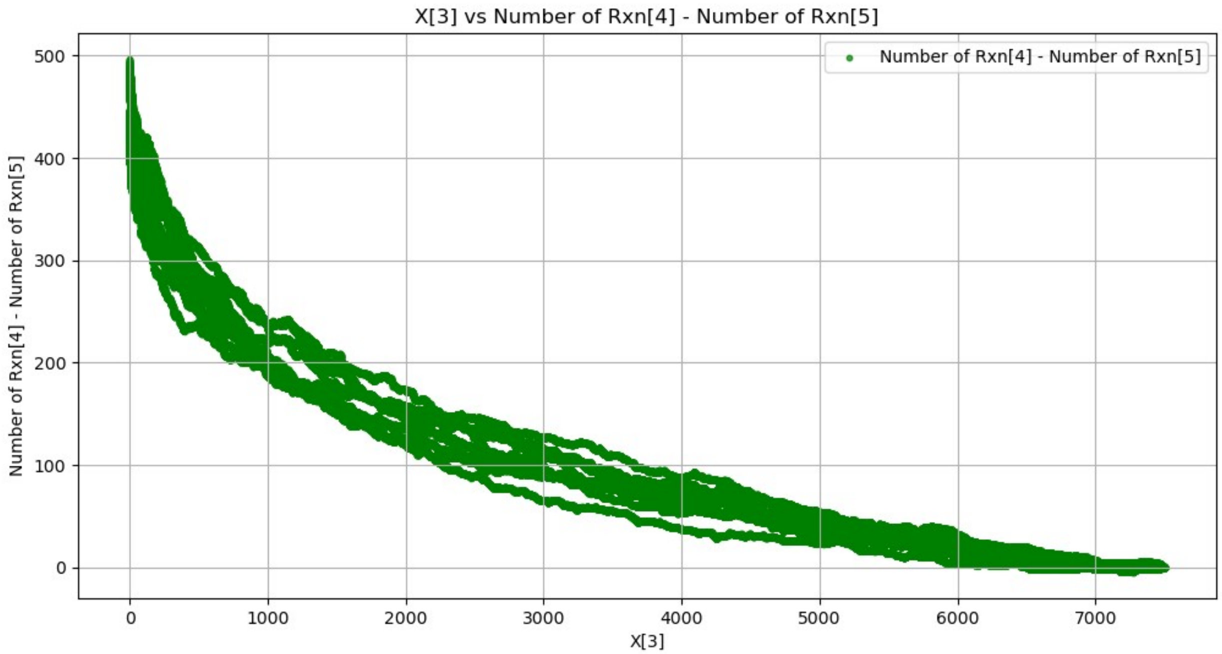

Figure S13. The cumulative graph of **(a)** the number of reactions **4a** → **1a** fired vs the concentration of fuel X [3], **(b)** the number of reactions **1a** → **4a** fired vs the concentration of fuel X [3] and **(c)** the difference of the number of reactions **4a** → **1a** and **1a** → **4a** fired vs the concentration of fuel X [3]; for n=10 simulations. This is for the hypothetical case where the water effect is absent.

Instead of plotting the graph of the difference of the number of reactions **4a** → **1a** and **1a** → **4a** fired vs the concentration of fuel X [3] , we plot the graph of between difference of number of reactions **4a** → **1a** (Rxn[4]) fired and number of reactions **1a** → **4a** (Rxn[5]) fired per fuel molecule vs the concentration of fuel X [3] (see **Figures S14**, **S15** and **S16** for single run data for the motor).

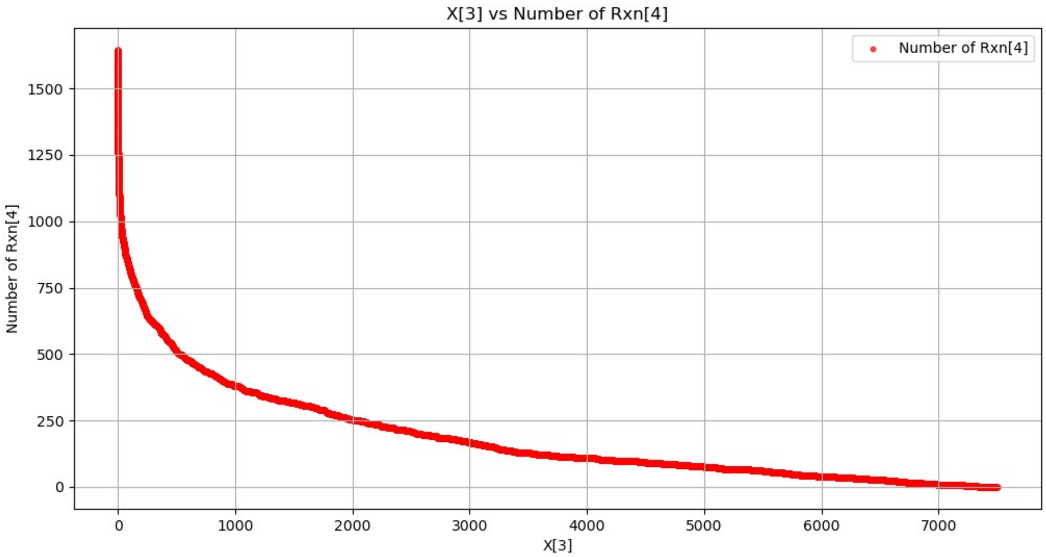

Figure S14. Graph between number of reactions **4a → 1a** (Rxn[4]) fired vs the concentration of fuel (R,R-Fuel) X [3] without incorporating water effect**.**

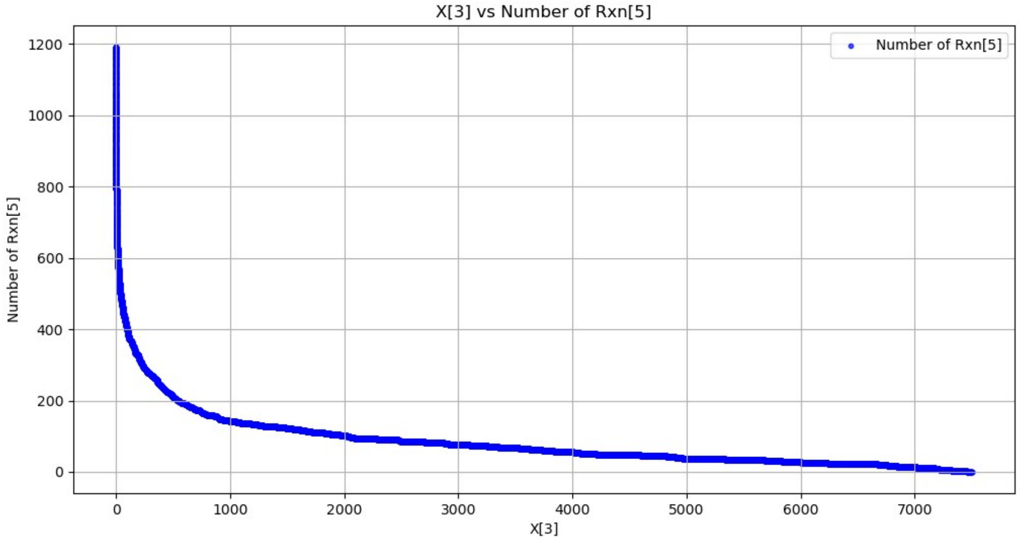

Figure S15. Graph between number of reactions **1a → 4a** (Rxn[5]) fired vs the concentration of fuel (R,R-fuel) X [3] without incorporating water effect.

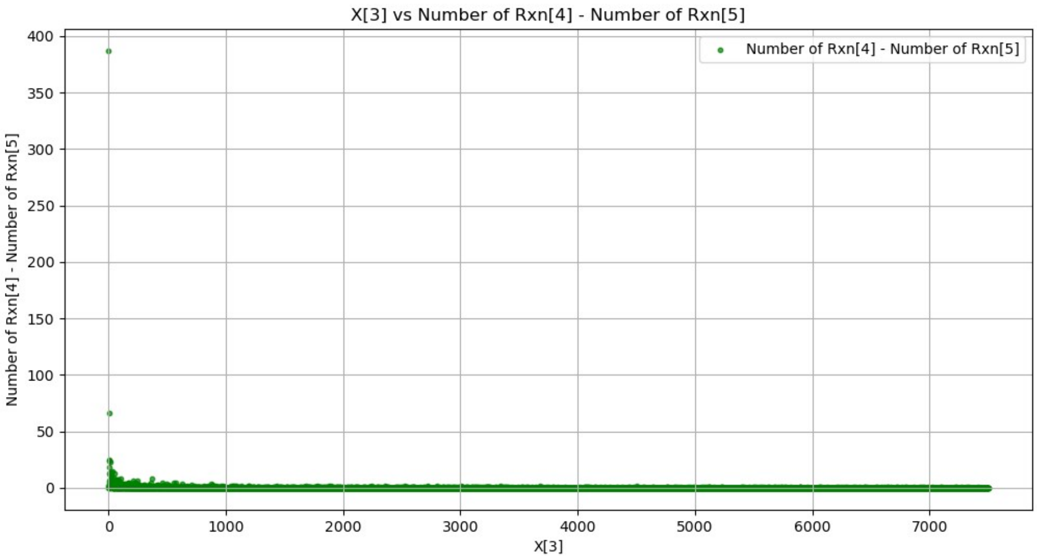

Figure S16. Graph between difference of number of reactions **4a** → **1a** (Rxn[4]) fired and number of reactions **1a** → **4a** (Rxn[5]) fired per fuel molecule vs the concentration of fuel X [3] without incorporating water effect.

We also ran the stochastic simulations (n=10 times) for the values of $\Delta E^{\ddagger}$ and $\Delta\Delta E^{\ddagger}$ determined by single point calculations at different levels of theory for the **1a** system without incorporating water effect. The values and simulation results are shown in the Tables S21-S25.

Table S21. Values of $\Delta E^{\ddagger}$ and $\Delta\Delta E^{\ddagger}$ (in kcal/mol) of different reactions calculated by DFT (level of theory: B3-LYP-D3/def2-TZVP (COSMO: ε=50.28) //PBE-D3/TZVP (COSMO: ε=50.28) , Temp. = 283.15K).

Type of reaction	$\Delta E^{\ddagger}$ (in kcal/mol)	$\Delta\Delta E^{\ddagger}$ (in kcal/mol)	$\Delta E^{\ddagger}_{rot}$ (in kcal/mol)	1a→4a rotation	4a→1a rotation	Net clockwise rotation
1a+ R-fuel ➔ 2a + R, R waste	13.8		17.3	8472	12611	4139
4a+ R-fuel ➔ 3a + R, R waste	14.7	**0.9**				
3a + water + R-catalyst ➔ 4a + R-catalyst	12.9					
2a + water + R-catalyst ➔ 1a + R-catalyst	14.7	**1.8**				

Table S22. Values of $\Delta E^{\ddagger}$ and $\Delta\Delta E^{\ddagger}$ (in kcal/mol) of different reactions calculated with DFT (level of theory: M06-2X-D3/def2-TZVP (COSMO: ε=50.28)//PBE-D3/TZVP (COSMO: ε=50.28), temperature: 283.15 K).

Type of reaction	$\Delta E^{\ddagger}$ (in kcal/mol)	$\Delta\Delta E^{\ddagger}$ (in kcal/mol)	$\Delta E^{\ddagger}_{rot}$ (in kcal/mol)	1a→4a rotation	4a→1a rotation	Net clockwise rotation
1a+R, R-fuel ➔ 2a + R, R waste	15.8		17.3	3,39,843	3,45,759	5916
4a+R, R-fuel ➔ 3a + R, R waste	16.6	**0.8**				
3a + water + R-catalyst ➔ 4a + R-catalyst	12.2					
2a + water + R-catalyst ➔ 1a + R-catalyst	15.5	**3.2**				

Table S23. Values of $\Delta E^{\ddagger}$ and $\Delta\Delta E^{\ddagger}$ (in kcal/mol) of different reactions calculated with DFT (level of theory: PBE0-D3/def2-TZVP (COSMO: ε=50.28)//PBE-D3/TZVP (COSMO: ε =50.28); temperature: 283.15 K).

Type of reaction	$\Delta E^{\ddagger}$ (in kcal/mol)	$\Delta\Delta E^{\ddagger}$ (in kcal/mol)	$\Delta\Delta E^{\ddagger}_{rot}$ (in kcal/mol)	1a→4a rotation	4a→1a rotation	Net clockwise rotation
1a+R, R-fuel ➔ 2a + R, R waste	13.6		16.6	20,996	24,919	3,923
4a+R, R-fuel ➔ 3a + R, R waste	14.4	**0.8**				
3a + water + R-catalyst ➔ 4a + R-catalyst	11.4					
2a + water + R-catalyst ➔ 1a + R-catalyst	12.3	**0.9**				

Table S24. Values of $\Delta E^{\ddagger}$ and $\Delta\Delta E^{\ddagger}$ (in kcal/mol) of different reactions calculated by DFT (level of theory – TPSSH-D3/def2-TZVP (COSMO: ε=50.28)//PBE-D3/TZVP (COSMO: ε=50.28) , Temp. =283.15K)

Type of reaction	$\Delta E^{\ddagger}$ (in kcal/mol)	$\Delta\Delta E^{\ddagger}$ (in kcal/mol)	$\Delta E^{\ddagger}_{rot}$ (in kcal/mol)	1a→4a rotation	4a→1a rotation	Net clockwise rotation
1a+R, R-fuel ➔ 2a + R, R waste	12.5		16.3	6,336	4,096	2,240
4a+R, R-fuel ➔ 3a + R, R waste	13.1	**0.6**				
3a + water + R-catalyst ➔ 4a + R-catalyst	11.0					
2a + water + R-catalyst ➔ 1a + R-catalyst	12.0	**1.0**				

Table S25. Values of ΔE‡ and ΔΔE‡ (in kcal/mol) of different reactions calculated by DFT (level of theory – ωB97X-D-D3/def2-TZVP (COSMO: ε=50.28) //PBE-D3/TZVP (COSMO: ε=50.28), temperature: 283.15K).

Type of reaction	ΔE‡ (in kcal/mol)	ΔΔE‡ (in kcal/mol)	$ΔE^‡_{rot}$ (in kcal/mol)	1a→4a rotation	4a→1a rotation	Net clockwise rotation
1a+R, R-fuel ➔ 2a + R, R waste	13.2		17.1	4,670	8,923	4,253
4a+R, R-fuel ➔ 3a + R, R waste	14.7	**1.5**				
3a + water + R-catalyst ➔ 4a + R-catalyst	13.4					
2a + water + R-catalyst ➔ 1a + R-catalyst	14.3	**0.9**				

11. Volume Conversions:

- $1000\ cm^3 = 1\ L$

- $1\ cm^3 = 0.001\ L$

- $10^{-18})\ m^3 = 10^{-12})\ cm^3$

- $1\ cm^3 = 10^{12}\ \mu m^3$

- $1\ \mu m^3 = 10^{-12}\ cm^3$

- $10^{-12}\ cm^3 = 10^{-15}\ L$

2. Concentration and Molecules:

- Concentration: 0.5 mM = 0.5 millimoles/L

- Number of molecules per liter: 0.5 millimoles/L = 3×10^{20} molecules/L

3. Volume and Molecule Count:

- 3×10^{20} molecules occupy 1 L

- 300 molecules occupy 10^{-18} L

In Simulations, we take 300 molecules of **1c**/**4c**/**1a**/**4a** and volume as 10^{-18} L . Similarly for R,R-fuel ([Fuel] =12.5 mM) , we take 7500 molecules and 600 molecules of R-catalyst were taken in simulation.when considering watter effect , 300+300 =600 molecules of hydrogen analogue were taken.

12. Direct vs machine-catalysed hydration of R, R fuel.

We also investigated how the absence of a motor molecule affected the hydration reaction of the R, R- fuel. The reaction mechanism for the hydration reaction of the fuel, which includes both thermodynamic and kinetic parameters, is shown in Figure S17. The R, R-fuel reacts with water to form adduct **F-1**, which is 5.4 kcal/mol more stable than the initial reactants. **F-1** converts into **F-2** *via* a 4-membered transition state ($\Delta E^{\ddagger}$ = 36.1 kcal/mol). A very high activation barrier compared to the machine-catalyzed pathway ($\Delta E^{\ddagger}$ = 11.2 kcal/mol in the case of **1a** reacting with R, R-fuel) verifies that the fuel almost always chooses to hydrolyze *via* the machine-catalyzed pathway. Subsequently, **F-2** transforms into waste **(W)** through a 4-membered transition state (simple keto-enol tautomerization) ($\Delta E^{\ddagger}$ = 21.4 kcal/mol).

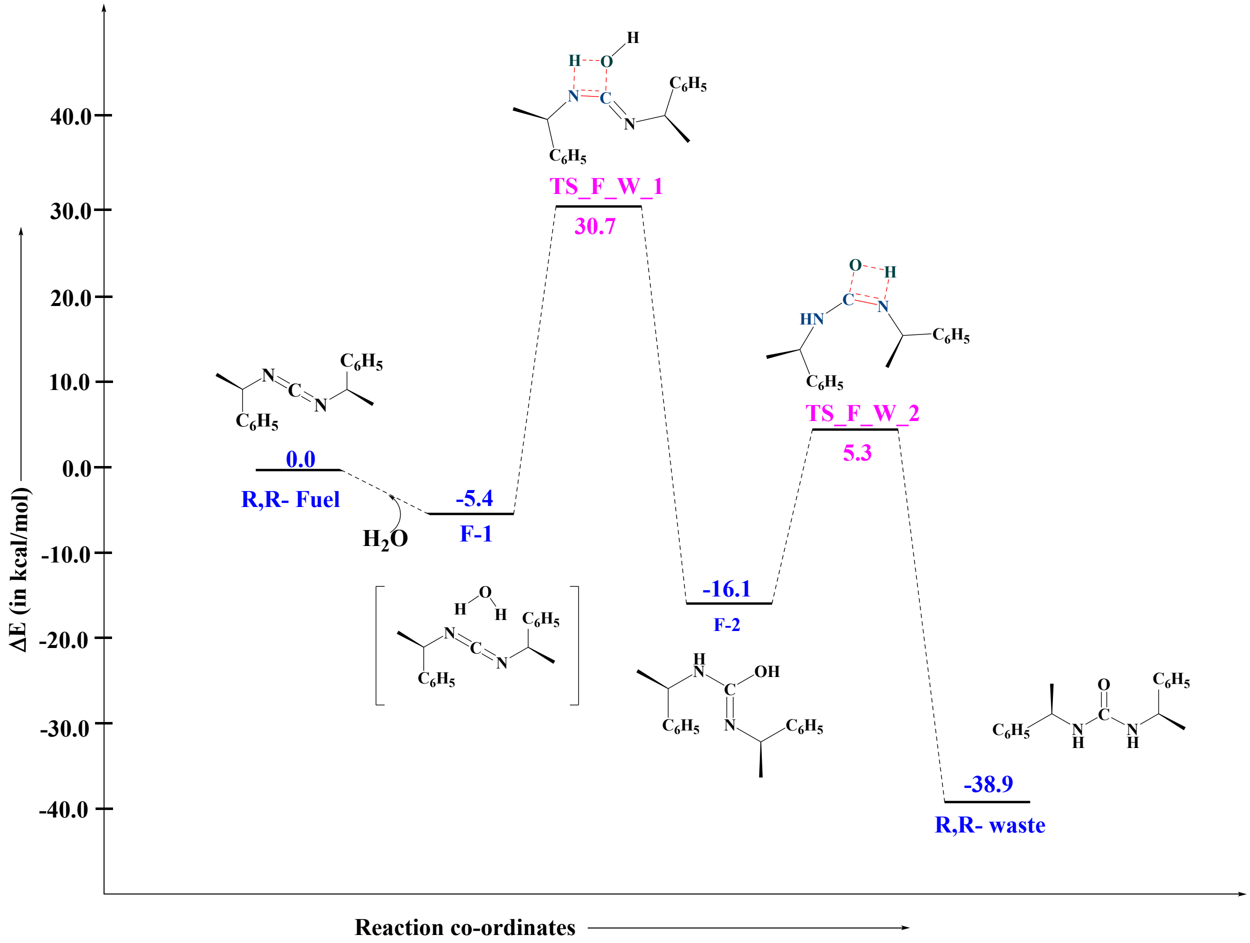

Figure S17. The reaction mechanism for the hydration reaction of the R, R-fuel.

13. Alternative mechanism to convert 1a into 4a by reacting with 2a and 3a

We explored an alternative mechanism for the conversion of **1a** to **4a** *via* a chemical reaction, in which **1a** reacts with **2a** or **3a** to form **4a** (See Figure S18), rather than simple C-N bond rotation. In this process, **1a** first reacts with **2a** and **3a** to form the adducts **Int_str_1_str_2** (ΔE = -4.8 kcal/mol) and **Int_str_1_str_3** (ΔE = -7.1 kcal/mol) respectively. Afterward, **Int_str_1_str_2** converts into **Prod_str_1_str_2** through a 6 membered transition state (**TS_str_1_str_2**, $\Delta E^{\ddagger}$ = 17.8 kcal/mol), and **Int_str_1_str_3** converts into **Prod_str_1_str_3** *via* a 6-membered transition state (**TS_str_1_str_3**, $\Delta E^{\ddagger}$ = 21.8 kcal/mol). Since both these activation barriers are much higher than for the **1a** + R,R fuel reaction ($\Delta E^{\ddagger}$ = 11.2 kcal/mol), this pathway has been discarded.

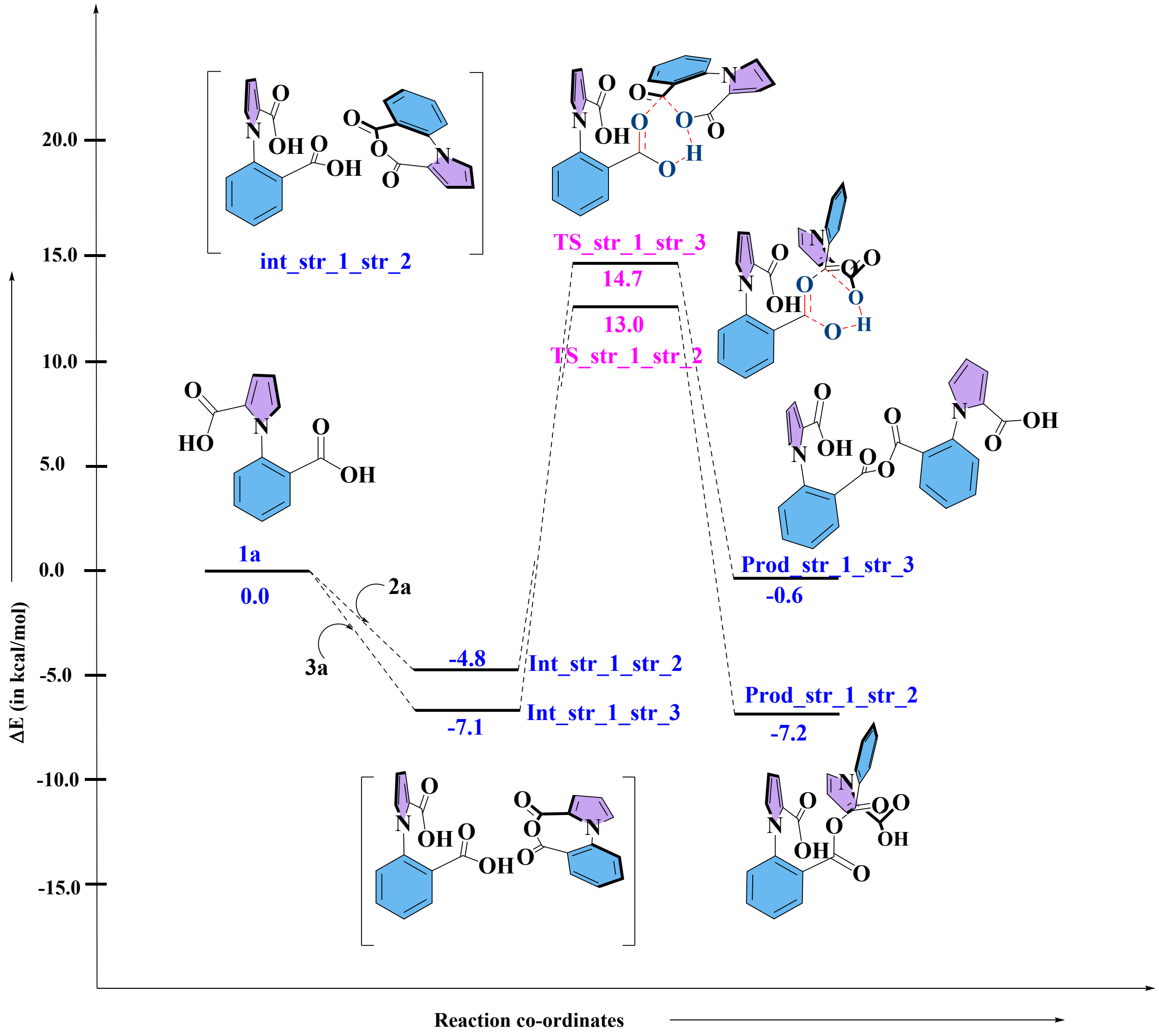

Figure S18. Energy profile diagram of the reaction of **1a** with **2a** and **3a**.

14. NCI (Non covalent interaction) and BCP (bond critical point) analysis of the rotational barrier of 1a and 1c.

MultiWFN program[13] was utilized to generate and evaluate NCI plot and AIM analysis(to obtain bond critical point) along with the Visual Molecular Dynamics (VMD) software for visualization.[14] We observed through the natural population analysis that the charge separation in the C=N bond corresponding to reactant (**1c**) (C: 0.181 and N: -0.279) is higher than that in the transition state (TS) (C: 0.161 and N: -0.269). This observation indicates an elevated polar covalent character in the case of **1c**. This is further validated by the observation of a slight increase (0.004 Å) in the C=N bond distance in the transition state, as well as a lower positive value of total electron density (ρ) in the bond critical point (BCP) analysis (Figure S19a and S19b). The higher polar covalent character suggests a stronger C=N bond in the reactant, potentially explaining the unusually high energy barrier associated with its rotation to **4**. The trend is also followed in the case of ethyl-substituted motor molecules.

a.

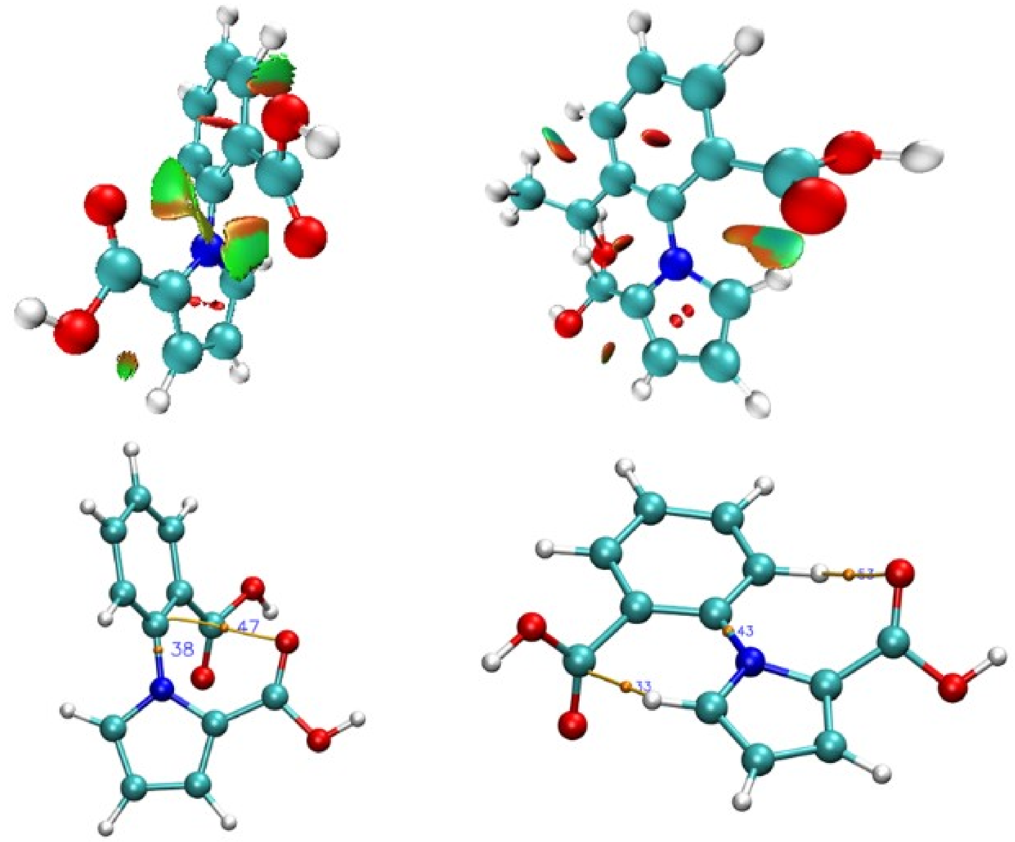

b.

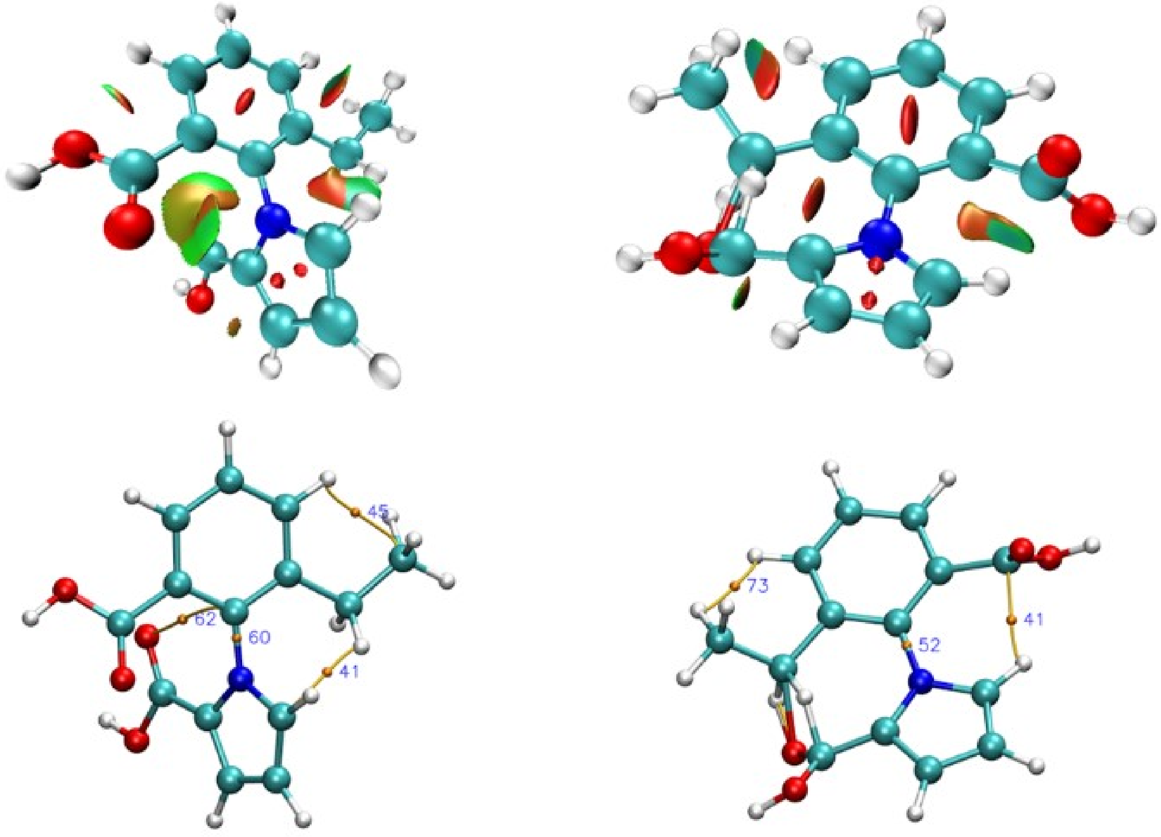

Figure S19. The NCI and BCP plots for : **(a)** Hydrogen substituted motor molecule (**1a**) and its corresponding rotational transition state. **(b)** Ethyl substituted motor molecule (**1c**) and its corresponding rotational transition state.

15. References

1. A. Uhe, S. Kozuch, S. Shaik, *J. Comput. Chem.*, 2011, **32**, 978–985.
2. S. Kozuch, S. Shaik, *J. Phys. Chem. A*, 2008, **112**, 6032–6041.
3. S. Kozuch, S. Shaik, *J. Am. Chem. Soc.* 2006, **128**, 3355–3365.
4. S. Kozuch, S. Shaik, *Acc. Chem. Res.*, 2011, **44**, 101–110.
5. (a). F. Weigend, *Phys. Chem. Chem. Phys.*, 2006,**8**, 1057-1065; (b). F. Weigend, R. Ahlrichs, *Phys. Chem. Chem. Phys.*, 2005,**7**, 3297-3305.
6. (a). A. D. Becke, *Phys. Rev. A*, 1988, **38**, 3098–3100; (b). C. Lee, W. Yang, R. G. Parr, *Phys. Rev. B*, 1988, **37**, 785–789.
7. Y. Zhao, D. G. Truhlar, *Theor. Chem. Acc*., 2008, **120**, 215–241.
8. J. P. Perdew, M. Ernzerhof, K. Burke, *J. Chem. Phys.*,1996, **105**, 9982–9985.
9. (a). J. Tao, J. P. Perdew, V. N. Staroverov, G. E. Scuseria, *Phys. Rev. Lett.*, 2003, **91**, 146401; (b). V. N. Staroverov, G. E. Scuseria, J. Tao, J. P. Perdew, J. *Chem. Phys.*, 2003, **119**, 12129–12137.
10. J.D. Chai, M. H. Gordon, *Phys. Chem. Chem. Phys.*, 2008, **10**, 6615–6620.
11. M. J. Schilstra, S. R. Martin, *Methods Enzymol.,* **2009**, *467*, 381–409.
12. S. Borsley, E. Kreidt, D. A. Leigh, B. M. W. Roberts, *Nature*, 2022, **604**, 80–85.
13. T. Lu, F. Chen, *J. Comput. Chem.,* 2012, **33**, 580– 592.
14. W. Humphrey, A. Dalke, K. Schulten, *J. Mol. Graphics,* 1996, **14**, 33– 38.

16. Cartessian co-ordinates

All the Energies are reported in hartree

Ethyl case

1c

Energy= -896.29836010071

C	-0.7030126	-0.3258085	-0.4125422
C	-3.4238026	-0.3857093	0.2202778
C	-1.4168200	0.8839679	-0.2729716
C	-1.3334544	-1.5686602	-0.2266260
C	-2.7045238	-1.5716314	0.0820717
C	-2.7793862	0.8401987	0.0577304
H	-3.2091804	-2.5313256	0.2169724
H	-3.3327536	1.7722895	0.1678238
H	-4.4888227	-0.4144396	0.4561842
N	0.6896558	-0.3096421	-0.7655662
C	1.1416200	-0.5586702	-2.0335137
H	0.4371536	-0.7785736	-2.8301703
C	1.7842151	-0.0349851	0.0545299
C	2.5284010	-0.4558547	-2.0464129
H	3.1656243	-0.6041994	-2.9136839
C	2.9330424	-0.1276852	-0.7364601
H	3.9468543	0.0266964	-0.3784893
C	1.6589863	0.2721022	1.4678486
C	-0.7612109	2.2073764	-0.4902829
O	0.6098973	0.3241999	2.1084181
O	0.1759564	2.4261744	-1.2446283

O	2.8765353	0.5062107	2.0429573
H	2.7120016	0.6982313	2.9900700
O	-1.3475679	3.1860156	0.2477555
H	-0.9088752	4.0319937	0.0136838
C	-0.5787557	-2.8770373	-0.2744935
C	-0.0406327	-3.2868710	1.1076719
H	0.2557645	-2.8171931	-0.9867047
H	-1.2599283	-3.6575159	-0.6461958
H	-0.8601827	-3.3965371	1.8333380
H	0.4908558	-4.2473667	1.0434819
H	0.6566573	-2.5330066	1.4996975

R,R- Fuel

Energy= -767.41664621258

C	0.0046601	-2.2041536	0.0010430
N	-1.0839587	-2.3022328	0.5619088
N	1.0936820	-2.3005618	-0.5590595
C	-2.3484648	-1.6005181	0.2460329
C	2.3554221	-1.5924319	-0.2470890
H	-2.9737643	-1.7182454	1.1426601
C	-3.0490033	-2.3041088	-0.9228886
H	-2.4536545	-2.2243188	-1.8440326
H	-3.1964949	-3.3690047	-0.6951114
H	-4.0306889	-1.8427814	-1.1023867
C	-2.1560473	-0.1104754	0.0106337
C	-2.6665413	0.8104294	0.9353224

C	-1.4754339	0.3723928	-1.1190818
C	-2.5046294	2.1867280	0.7398007
H	-3.1959439	0.4447254	1.8192968
C	-1.3077182	1.7444665	-1.3142533
H	-1.0613281	-0.3254389	-1.8505353
C	-1.8237940	2.6564309	-0.3864638
H	-2.9064328	2.8905910	1.4720599
H	-0.7620146	2.1041297	-2.1888059
H	-1.6844877	3.7290643	-0.5363140
C	2.1563913	-0.1033941	-0.0110308
C	1.4767841	0.3760680	1.1207021
C	2.6594571	0.8199906	-0.9372881
C	1.3026156	1.7472973	1.3163000
H	1.0685425	-0.3237672	1.8535011
C	2.4912008	2.1954485	-0.7413232
H	3.1878854	0.4569222	-1.8229345
C	1.8113101	2.6617984	0.3869456
H	0.7578226	2.1043039	2.1925263
H	2.8870092	2.9013105	-1.4749113
H	1.6669880	3.7337240	0.5371778
H	2.9786982	-1.7070852	-1.1455039
C	3.0628804	-2.2928000	0.9196201
H	3.2151839	-3.3568333	0.6910622
H	4.0427023	-1.8264552	1.0963363
H	2.4698327	-2.2163268	1.8425301

1c-A

Energy= -1663.73552843411

N	-2.9415907	-1.4003222	2.4797781
C	-2.7177326	-0.2608730	2.1087979
N	-2.4081888	0.9158636	1.8760307
O	0.4821317	-0.7090336	1.6638479
C	0.9557487	0.4218041	1.5954045
O	0.2215913	1.5395199	1.6909058
C	2.4173703	0.7090510	1.4315619
C	3.2430171	-0.1101433	0.6357425
C	4.6226954	0.1325299	0.5214867
C	5.1601975	1.2127531	1.2414106
C	4.3557680	2.0378748	2.0262365
C	2.9839338	1.7966887	2.1097520
N	2.6746097	-1.2215670	-0.0774373
C	2.7310903	-2.5122092	0.3719160
C	2.0777971	-3.3370285	-0.5380711
C	1.6025374	-2.5132272	-1.5775816
C	1.9721022	-1.1966425	-1.2835388
C	1.7364386	0.0199322	-2.0386193
O	0.9894639	-0.2228038	-3.1626533
O	2.1373926	1.1459529	-1.7548063
C	-4.7354224	1.4168387	1.0747041
H	6.2336010	1.4060364	1.1739331
H	2.3446083	2.4377635	2.7170918
H	4.7971844	2.8719656	2.5746117

H	3.2180242	-2.7398487	1.3154673
H	1.9600512	-4.4131020	-0.4466641
H	1.0486522	-2.8205580	-2.4594479
H	-5.3855389	2.2313330	0.7272119
H	-4.6962389	0.6464890	0.2887887
H	-5.1833571	0.9775669	1.9778929
H	0.9070461	0.6327166	-3.6331182
C	-2.9068581	-2.6493912	1.6733688
C	-3.3358506	1.9605662	1.3673601
H	-3.4017222	2.7174805	2.1649868
C	-3.0101431	-2.3685507	0.1838806
C	-4.2565687	-2.4764959	-0.4514259
C	-1.8969570	-1.9621477	-0.5692998
C	-4.3942664	-2.1845806	-1.8122223
H	-5.1291857	-2.7897639	0.1282235
C	-2.0322891	-1.6760984	-1.9314561
H	-0.9205530	-1.8532502	-0.0927599
C	-3.2793718	-1.7846118	-2.5564689
H	-5.3719017	-2.2724201	-2.2911404
H	-1.1593574	-1.3538528	-2.5026734
H	-3.3820500	-1.5554572	-3.6193142
C	-2.7070544	2.6164465	0.1477739
C	-2.2700942	1.8352890	-0.9315407
C	-2.5702004	4.0086365	0.0802638
C	-1.7064650	2.4350146	-2.0587303
H	-2.3565767	0.7480225	-0.8844458

C	-2.0084724	4.6131994	-1.0507879
H	-2.8999511	4.6253294	0.9207524
C	-1.5733580	3.8274131	-2.1229422
H	-1.3666851	1.8127431	-2.8896476
H	-1.9037224	5.6995417	-1.0896834
H	-1.1284965	4.2969039	-3.0027900
H	-0.7636411	1.2742712	1.7984819
H	-3.8061164	-3.2049419	1.9796849
C	-1.6747949	-3.4630925	2.0785917
H	-1.6784178	-4.4231944	1.5441192
H	-0.7492852	-2.9235116	1.8382863
H	-1.6931929	-3.6629034	3.1589318
C	5.5158116	-0.6733136	-0.3926597
C	5.6895781	-0.0049272	-1.7680032
H	5.1149841	-1.6874953	-0.5269504
H	6.5013132	-0.7805599	0.0864411
H	6.3410341	-0.6134135	-2.4117869
H	4.7203406	0.1170193	-2.2716660
H	6.1425920	0.9925602	-1.6662833

1c-B

Energy= -1663.74152321396

C	3.0899705	0.2676605	-0.0094524
C	3.5525574	2.6349923	-1.4236553
C	2.0099554	0.9622689	-0.5983320
C	4.4140016	0.7341433	-0.1471072

C	4.6144074	1.9339888	-0.8454950
C	2.2572930	2.1400833	-1.3219177
H	5.6278129	2.3189864	-0.9635390
H	1.4169620	2.6633182	-1.7795984
H	3.7418945	3.5625859	-1.9661291
N	2.8600252	-0.9470975	0.7107958
C	2.9779603	-1.0642586	2.0807560
H	3.4128040	-0.2597025	2.6654832
C	2.1831587	-2.0618896	0.2235905
C	2.4156920	-2.2684270	2.4685903
H	2.3816660	-2.6535443	3.4842618
C	1.9005393	-2.8913249	1.3012199
H	1.4064993	-3.8568060	1.2336206
C	1.9164723	-2.2623715	-1.2165985
C	0.5905749	0.5117708	-0.5658571
O	2.7678971	-2.0520642	-2.0860835
O	0.2435495	-0.0422576	0.6594174
O	0.6940321	-2.7023369	-1.5156929
O	-0.1971623	0.6453314	-1.4764863
C	-0.9673351	-0.7213737	0.7606470
N	-1.1220692	-1.8171333	0.0944249
N	-1.8005946	-0.1527511	1.6549318
C	-1.6385413	1.2045302	2.2140876
H	-2.7138854	-0.5882829	1.7665853
C	-2.4081352	-2.5333687	0.1919892
H	-0.0386934	-2.3801990	-0.7776691

C	-3.5658032	-1.6882902	-0.3327223
C	-4.7541526	-1.5741199	0.4027544
C	-3.4541723	-1.0078502	-1.5556680
C	-5.8144270	-0.7907483	-0.0706616
H	-4.8492698	-2.0970123	1.3589270
C	-4.5120047	-0.2304856	-2.0317537
H	-2.5197869	-1.0612364	-2.1175705
C	-5.6954772	-0.1178510	-1.2905027
H	-6.7297023	-0.7030550	0.5182333
H	-4.4092708	0.3017107	-2.9797735
H	-6.5175125	0.4986972	-1.6596019
C	-1.7256982	2.2782641	1.1387078
C	-0.7427632	3.2727286	1.0599958
C	-2.7788248	2.2848979	0.2132405
C	-0.8092808	4.2616033	0.0724050
H	0.0919159	3.2627564	1.7663291
C	-2.8468203	3.2688874	-0.7741905
H	-3.5358295	1.4974125	0.2408118
C	-1.8621529	4.2616315	-0.8474361
H	-0.0303750	5.0249613	0.0147522
H	-3.6663499	3.2552874	-1.4962346
H	-1.9114678	5.0266570	-1.6251162
H	-2.6102770	-2.7664260	1.2551977
C	-2.2955645	-3.8612853	-0.5640316
H	-1.4746142	-4.4680049	-0.1557476
H	-3.2315572	-4.4276839	-0.4701927

H	-2.1023306	-3.6825762	-1.6317148
C	-2.6946152	1.3926248	3.3053651
H	-2.5779784	2.3816001	3.7666555
H	-3.7090888	1.3344263	2.8810204
H	-2.5897607	0.6257414	4.0862357
H	-0.6399656	1.2548209	2.6725041
C	5.5715055	-0.0690968	0.4107708
C	6.9516752	0.2848800	-0.1440916
H	5.3613034	-1.1351897	0.2284034
H	5.5866848	0.0374775	1.5087628
H	7.7029294	-0.4050120	0.2651930
H	6.9785655	0.2017399	-1.2412434
H	7.2593009	1.3048666	0.1285706

1c-C

Energy= -1663.74344555795

C	3.0873305	0.2583579	-0.0142873
C	3.5515461	2.6230146	-1.4417482
C	2.0121091	0.9472881	-0.6202361
C	4.4095826	0.7318877	-0.1459260
C	4.6102816	1.9291196	-0.8485613
C	2.2585353	2.1219888	-1.3514856
H	5.6231304	2.3178247	-0.9597891
H	1.4203017	2.6392453	-1.8195824
H	3.7419747	3.5490567	-1.9862038
N	2.8558510	-0.9491380	0.7109641

C	2.9792520	-1.0564580	2.0853960
H	3.4018640	-0.2423421	2.6657070
C	2.2028899	-2.0781573	0.2219764
C	2.4362227	-2.2667739	2.4750025
H	2.4032699	-2.6473588	3.4927801
C	1.9371440	-2.9048666	1.3041597
H	1.4549152	-3.8767928	1.2391317
C	1.9027275	-2.2746807	-1.2329514
C	0.5997365	0.5017712	-0.5927892
O	2.7863718	-2.0061172	-2.0813422
O	0.2456413	-0.0507572	0.6593604
O	0.7174322	-2.7140879	-1.4941916
O	-0.2084586	0.6318993	-1.4800169
C	-0.9505069	-0.6965004	0.7798966
N	-1.1491559	-1.8102343	0.1184505
N	-1.7969603	-0.1464055	1.6483922
C	-1.6373889	1.2097740	2.2252925
H	-2.6862232	-0.6187929	1.8046073
C	-2.4300409	-2.5442118	0.1780401
H	-0.3425630	-2.2101514	-0.5114408
C	-3.5784744	-1.6879445	-0.3426993
C	-4.7608267	-1.5697497	0.4007969
C	-3.4663471	-1.0088884	-1.5655784
C	-5.8194686	-0.7823727	-0.0684553
H	-4.8537497	-2.0940772	1.3562106
C	-4.5225181	-0.2259587	-2.0355752

H	-2.5381360	-1.0704681	-2.1372150
C	-5.7016467	-0.1093952	-1.2882684
H	-6.7321901	-0.6919876	0.5235663
H	-4.4223027	0.3051929	-2.9841901
H	-6.5227181	0.5102526	-1.6539765
C	-1.7216618	2.2802610	1.1479572
C	-0.7308691	3.2661181	1.0626820
C	-2.7810763	2.2925044	0.2295062
C	-0.7966624	4.2534437	0.0735421
H	0.1068215	3.2525209	1.7651065
C	-2.8461110	3.2737999	-0.7606015
H	-3.5475202	1.5143962	0.2657777
C	-1.8540150	4.2583812	-0.8409428
H	-0.0135762	5.0118893	0.0108522
H	-3.6696941	3.2652205	-1.4778557
H	-1.9019989	5.0220944	-1.6198689
H	-2.6305427	-2.7923720	1.2354368
C	-2.2811964	-3.8543732	-0.5987561
H	-1.4612141	-4.4584453	-0.1863016
H	-3.2142384	-4.4266323	-0.5238915
H	-2.0739039	-3.6573149	-1.6597939
C	-2.6987633	1.3836209	3.3118234
H	-2.5825738	2.3687223	3.7807735
H	-3.7112414	1.3297005	2.8829896
H	-2.5951737	0.6114207	4.0872960
H	-0.6399596	1.2523357	2.6860409

C	5.5670060	-0.0685206	0.4154628
C	6.9473463	0.2853789	-0.1392108
H	5.3558140	-1.1342980	0.2326103
H	5.5808072	0.0379247	1.5133447
H	7.6983324	-0.4054281	0.2690066
H	6.9740691	0.2031671	-1.2364739
H	7.2560684	1.3048607	0.1341296

R,R -Waste

Energy= -843.86563046775

C	0.3405666	-2.0547848	0.4906340
N	-0.0612961	-1.5317020	-0.7188968
N	1.6614688	-1.8686695	0.8176483
C	-1.4791008	-1.4397435	-1.0691993
C	2.6605249	-1.1148205	0.0555945
C	-2.2151854	-0.3353190	-0.3157118
C	-3.5279767	-0.5463576	0.1287104
C	-1.6222229	0.9164200	-0.0960585
C	-4.2373300	0.4715639	0.7754192
H	-3.9982116	-1.5213667	-0.0279617
C	-2.3258240	1.9350694	0.5529025
H	-0.5941603	1.1019174	-0.4162127
C	-3.6376026	1.7175221	0.9896338
H	-5.2576401	0.2883512	1.1197771
H	-1.8444617	2.9013404	0.7212600
H	-4.1868577	2.5120565	1.4990735

C	2.3139172	0.3690069	-0.0513834
C	2.3520027	1.0144528	-1.2955052
C	1.9497256	1.1059203	1.0859679
C	2.0254477	2.3718859	-1.4055990
H	2.6323715	0.4483662	-2.1891156
C	1.6312359	2.4613291	0.9804059
H	1.8947598	0.6072873	2.0567102
C	1.6643648	3.0980996	-0.2669911
H	2.0473391	2.8580919	-2.3831114
H	1.3422597	3.0215904	1.8719738
H	1.4015235	4.1544123	-0.3504264
H	1.9358285	-2.2666685	1.7115738
H	0.5445003	-0.8405253	-1.1571733
O	-0.4299315	-2.6985472	1.2306393
C	4.0320504	-1.3255575	0.7036938
H	4.0360508	-0.9382344	1.7336625
H	4.2894527	-2.3942945	0.7243533
H	4.8025006	-0.7907217	0.1334432
H	2.7030377	-1.5262803	-0.9689348
C	-1.5974582	-1.2418004	-2.5854437
H	-1.1216863	-0.2965981	-2.8922701
H	-1.1139054	-2.0701458	-3.1227274
H	-2.6542144	-1.1957951	-2.8805328
H	-1.9363791	-2.4008520	-0.7933885

TS_1c_1

Energy= -1663.71707710256

C	3.9550572	-0.5358342	0.3919694
C	6.0912272	-0.0035936	-1.3119133
C	3.7595032	0.2908744	-0.7286714
C	5.2132600	-1.0826994	0.6965654
C	6.2697505	-0.8126641	-0.1892031
C	4.8413043	0.5612587	-1.5748260
H	7.2564197	-1.2313128	0.0243283
H	4.6951765	1.2129244	-2.4382168
H	6.9323443	0.1995490	-1.9778440
N	2.8634742	-0.7089577	1.3166832
C	2.5644614	0.2143464	2.2836302
H	3.1565696	1.1202821	2.3735732
C	1.9710569	-1.7725838	1.4222104
C	1.4806889	-0.2449776	3.0266865
H	1.0240254	0.2726929	3.8655988
C	1.1054259	-1.4907698	2.4852330
H	0.2915420	-2.1312093	2.8115574
C	1.9823469	-2.9106716	0.5202860
C	2.4035458	0.8734409	-1.0478305
O	2.7708970	-3.1060708	-0.3997163
O	2.3138414	2.1331082	-1.2098337
O	0.9733037	-3.7907980	0.8149633
O	1.4370452	0.0507527	-1.1521615
N	-0.3002824	2.0793142	-1.0141204
C	-0.5771082	0.8328670	-1.1771932

N	-1.2979447	-0.1170666	-1.3615694
C	-1.2559617	3.0306221	-0.3638941
C	-1.2840717	-1.4739409	-0.8032621
C	-2.0683493	2.3586321	0.7276465
C	-1.4534057	1.5546152	1.6992938
C	-3.4528778	2.5621863	0.7882188
C	-2.2144084	0.9535678	2.7035688
H	-0.3772469	1.3676616	1.6586601
C	-4.2130097	1.9744585	1.8042932
H	-3.9415325	3.1755411	0.0266246
C	-3.5960608	1.1650769	2.7620088
H	-1.7278194	0.3088098	3.4381519
H	-5.2929817	2.1328125	1.8338521
H	-4.1915401	0.6845749	3.5404327
C	-2.5585800	-1.7342446	-0.0178611
C	-3.7722926	-1.1136238	-0.3370825
C	-2.5178067	-2.6552289	1.0395850
C	-4.9289212	-1.4069409	0.3910754
H	-3.8068066	-0.3778720	-1.1431517
C	-3.6747826	-2.9517458	1.7647611
H	-1.5713780	-3.1386983	1.2957506
C	-4.8851300	-2.3269677	1.4428147
H	-5.8655304	-0.9032434	0.1429729
H	-3.6288138	-3.6658939	2.5899477
H	-5.7875197	-2.5499606	2.0159183
C	-0.4425644	4.2180164	0.1547032

H	0.2516052	3.8998837	0.9455579
H	0.1308299	4.6854728	-0.6586987
H	-1.1260871	4.9683113	0.5730627
H	-1.9438905	3.3794446	-1.1501617
H	0.7396575	2.3304042	-1.1644224
C	-1.0872960	-2.4793361	-1.9430008
H	-1.9073329	-2.4041137	-2.6712891
H	-0.1316468	-2.2935128	-2.4524534
H	-1.0757226	-3.4980009	-1.5320762
H	-0.4291519	-1.5404460	-0.1148925
H	1.0547490	-4.5358401	0.1835424
C	5.4559836	-1.9440175	1.9144694
C	5.6773169	-3.4250402	1.5630146
H	4.6136045	-1.8538755	2.6144147
H	6.3455980	-1.5614152	2.4405774
H	5.8516029	-4.0141017	2.4752430
H	4.8028054	-3.8383048	1.0418253
H	6.5511231	-3.5494348	0.9065685

TS_1c_2

Energy= -1663.74140394995

C	3.5696409	-1.1384445	0.7746089
C	4.9038220	1.2327328	0.1201590
C	2.8365220	-0.0002126	0.3703153
C	4.9792069	-1.1091542	0.8261341
C	5.6201989	0.0981442	0.5110121

C	3.5180791	1.1784707	0.0255530
H	6.7090165	0.1486624	0.5473833
H	2.9409494	2.0472658	-0.2933694
H	5.4326151	2.1554547	-0.1237356
N	2.8934590	-2.3532264	1.1085444
C	2.8111898	-2.8606582	2.3913198
H	3.3991315	-2.4258786	3.1935050
C	1.9730316	-3.0185887	0.3030467
C	1.8691808	-3.8741168	2.4011284
H	1.6006958	-4.4806391	3.2621244
C	1.3302755	-3.9652015	1.0892551
H	0.5767432	-4.6630028	0.7337747
C	1.8110858	-2.7201241	-1.1423645
C	1.3579549	0.0467978	0.2200627
O	2.7914638	-2.5787345	-1.8891337
O	0.7054531	-0.6648659	1.2266695
O	0.5644986	-2.6268221	-1.5636643
O	0.7635168	0.6622156	-0.6358109
C	-0.6488596	-0.9151701	1.0904219
N	-1.0495641	-1.6873766	0.1285859
N	-1.3843326	-0.3680163	2.0723217
C	-0.8973090	0.6634277	3.0124998
H	-2.3909229	-0.5149019	2.0300876
C	-2.4917699	-1.9517435	-0.0389515
H	-0.1755000	-2.2424666	-0.7294922
C	-3.2653761	-0.6705127	-0.3351916

C	-4.4455570	-0.3774645	0.3618317
C	-2.8018877	0.2340276	-1.3028580
C	-5.1528292	0.8025854	0.1006677
H	-4.8121261	-1.0748151	1.1208930
C	-3.5086267	1.4083880	-1.5681221
H	-1.8652233	0.0311036	-1.8262357
C	-4.6855918	1.6977419	-0.8661708
H	-6.0653577	1.0230375	0.6583491
H	-3.1335078	2.1088974	-2.3172329
H	-5.2316439	2.6214952	-1.0678711
C	-0.5207132	1.9506208	2.2922948
C	0.7173240	2.5557578	2.5405704
C	-1.3980422	2.5411977	1.3716292
C	1.0768027	3.7350193	1.8790532
H	1.4132530	2.0905439	3.2439170
C	-1.0409710	3.7162201	0.7085427
H	-2.3547068	2.0649195	1.1439906
C	0.1980621	4.3175866	0.9607393
H	2.0498598	4.1912718	2.0728784
H	-1.7281165	4.1596462	-0.0154841
H	0.4803783	5.2327891	0.4361602
H	-2.8841508	-2.3797022	0.9026517
C	-2.6829510	-2.9957420	-1.1430385
H	-2.1412708	-3.9206657	-0.8989259
H	-3.7500855	-3.2314054	-1.2493272
H	-2.3122150	-2.6150214	-2.1053306

C	-1.9807702	0.8849066	4.0698954
H	-1.6327776	1.6223899	4.8041214
H	-2.9019855	1.2734936	3.6095509
H	-2.2113865	-0.0529507	4.5952251
H	0.0027756	0.2631042	3.5017450
C	5.7606487	-2.3612351	1.1689298
C	7.2418107	-2.3418270	0.7895055
H	5.2634647	-3.2135265	0.6786389
H	5.6689128	-2.5580486	2.2503960
H	7.6920739	-3.3205508	1.0068728
H	7.3811059	-2.1363609	-0.2826801
H	7.8059947	-1.5875000	1.3570722

TS_1c_3

Energy= -1663.73409463956

C	3.1908213	-1.1291316	0.3590910
C	3.6342909	-2.5011288	2.7646758
C	2.3412249	-0.9161448	1.4673353
C	4.3196218	-1.9735712	0.4762315
C	4.4974928	-2.6690254	1.6843658
C	2.5749678	-1.6033770	2.6647917
H	5.3504761	-3.3411191	1.7872597
H	1.9128301	-1.4194579	3.5111888
H	3.7997033	-3.0524809	3.6917301
N	2.9308891	-0.4942812	-0.8951330
C	2.8665151	-1.1966166	-2.0842427

H	2.9397055	-2.2791144	-2.0876008
C	2.8278783	0.8734017	-1.1763469
C	2.7193203	-0.2968337	-3.1256075
H	2.6164614	-0.5563838	-4.1757791
C	2.7072747	1.0020054	-2.5561136
H	2.5898147	1.9496186	-3.0744658
C	2.7605318	1.9679938	-0.1744619
C	1.2216947	0.0672912	1.5061660
O	3.1073584	3.1194378	-0.5275090
O	0.4336519	-0.0173703	0.2556554
O	2.3166264	1.6972484	1.0150644
O	0.6676019	0.4416666	2.5292859
C	-0.5360292	0.8523908	-0.0142571
N	-0.9318823	1.7802554	0.8519939
N	-1.0780637	0.7366872	-1.2285594
C	-0.7761121	-0.3587530	-2.1715380
H	-1.9245682	1.2788217	-1.4049247
C	-2.0109355	2.7537324	0.5988262
H	-0.4868137	1.7218865	1.7752526
C	-3.3378495	2.0554662	0.3188604
C	-4.1037653	2.4301639	-0.7940420
C	-3.8018906	1.0307495	1.1574010
C	-5.3169086	1.7874632	-1.0686257
H	-3.7472953	3.2265844	-1.4535477
C	-5.0126874	0.3915966	0.8852975
H	-3.2047469	0.7184566	2.0176570

C	-5.7720639	0.7663489	-0.2299376
H	-5.9009817	2.0823374	-1.9425177
H	-5.3607766	-0.4100617	1.5395999
H	-6.7134328	0.2578793	-0.4468027
C	-1.4505554	-1.6560573	-1.7487574
C	-2.8209650	-1.6962869	-1.4500824
C	-0.7006010	-2.8364778	-1.6692122
C	-3.4298837	-2.8976721	-1.0789546
H	-3.4218701	-0.7845542	-1.4939174
C	-1.3090151	-4.0425443	-1.3045444
H	0.3695618	-2.8073383	-1.8877543
C	-2.6757036	-4.0755423	-1.0081503
H	-4.4960067	-2.9147723	-0.8431935
H	-0.7122394	-4.9549820	-1.2437607
H	-3.1515818	-5.0144755	-0.7180899
H	-1.7305035	3.3404949	-0.2923274
C	-2.0887670	3.6963463	1.8002623
H	-2.8616670	4.4538716	1.6207245
H	-2.3546912	3.1414499	2.7119527
H	-1.1275372	4.2062863	1.9562816
C	-1.1888276	0.0921958	-3.5725710
H	-0.9545012	-0.6996895	-4.2954737
H	-2.2713283	0.2876790	-3.6240359
H	-0.6467529	1.0039904	-3.8605943
H	0.3112732	-0.5039092	-2.1531722
C	5.3501460	-2.1128142	-0.6301578

C 6.7975790 -2.2451271 -0.1406004

H 5.2777453 -1.2431762 -1.2981065

H 5.1011535 -2.9877281 -1.2545955

H 7.4801535 -2.2412886 -1.0024627

H 7.0721577 -1.4064164 0.5161624

H 6.9725532 -3.1799988 0.4100295

2c

Energy= -819.877404777803

C -0.6490232 0.0858753 -0.0542440

C -2.5987812 2.1013779 0.0634360

C -0.2777534 1.4465719 -0.1079742

C -1.9996158 -0.2761064 0.1389742

C -2.9501247 0.7589089 0.1846180

C -1.2576828 2.4484448 -0.0768037

H -3.9974861 0.4911593 0.3426969

H -0.9502917 3.4927733 -0.1367912

H -3.3641940 2.8776762 0.1020348

N 0.3663329 -0.9038573 -0.2177172

C 1.5503835 -0.9898635 0.5258902

C 0.2279028 -2.0704723 -0.9339696

H -0.6223191 -2.2161900 -1.5907403

C 1.3064009 -2.8994970 -0.6639591

H 1.4828563 -3.8721773 -1.1138312

C 2.1290763 -2.2325843 0.2680939

H 3.0814943 -2.5663892 0.6702533

C	2.1908367	0.0895858	1.2410077
C	1.1248351	1.9227913	-0.1615146
O	2.0334746	1.4056260	0.7633537
O	1.5055151	2.8590401	-0.8305547
O	2.9895831	-0.0527519	2.1480607
C	-2.4911687	-1.6916531	0.3556777
C	-3.3072600	-2.2516354	-0.8197622
H	-3.1324977	-1.6750322	1.2513535
H	-1.6556929	-2.3642858	0.5918544
H	-3.6587135	-3.2675065	-0.5905811
H	-4.1873867	-1.6241941	-1.0221070
H	-2.7164109	-2.2967948	-1.7465448

3c

Energy= -819.87740510239

C	-0.8403112	-0.0309518	0.3231358
C	-2.9786632	1.7765743	0.2015008
C	-0.6198103	1.3487978	0.4989750
C	-2.1263503	-0.5158472	-0.0070177
C	-3.1752074	0.4176793	-0.0473193
C	-1.6965962	2.2478865	0.4632843
H	-4.1748167	0.0580026	-0.3000547
H	-1.5069748	3.3102931	0.6187645
H	-3.8203888	2.4694261	0.1668080
N	0.2618718	-0.9216465	0.5031536
C	1.4813195	-0.8445486	-0.1806342

C	0.2076975	-2.1351215	1.1471383
H	-0.6514523	-2.4017862	1.7519131
C	1.3746076	-2.8377839	0.8847929
H	1.6283686	-3.8129432	1.2901610
C	2.1693532	-2.0383481	0.0348891
H	3.1686450	-2.2524743	-0.3336588
C	2.0402898	0.3440153	-0.7832936
C	0.7224521	1.9514146	0.6847652
O	1.7323537	1.5959203	-0.2118632
O	0.9668567	2.8629187	1.4451071
O	2.8868679	0.3532138	-1.6568684
C	-2.4149513	-1.9433791	-0.4260679
C	-1.6780354	-2.3506684	-1.7131252
H	-3.4991670	-2.0222589	-0.5917067
H	-2.1827437	-2.6598585	0.3732294
H	-1.9772008	-3.3650408	-2.0122475
H	-1.9217479	-1.6633035	-2.5361419
H	-0.5868664	-2.3485352	-1.5811240

R-catalyst

Energy= -2707.35302191689

C	-0.7096827	-0.6190633	0.5695326
C	-2.2377900	-0.6747745	0.4666869
C	-2.8707192	-1.8866712	0.7779266
C	-3.0230007	0.4045834	0.0583303
C	-4.2558590	-2.0115428	0.6589349

H	-2.2702197	-2.7293076	1.1219641
C	-4.4121969	0.2713875	-0.0506908
H	-2.5635516	1.3677159	-0.1639228
C	-5.0437679	-0.9353435	0.2427231
H	-6.1245123	-1.0355300	0.1550236
C	-0.1624155	0.8178985	0.5515629
C	0.3544803	1.3695760	1.7280901
C	-0.1151596	1.5879100	-0.6178062
C	0.9106610	2.6519632	1.7289292
H	0.3244612	0.7795772	2.6436901
C	0.4466980	2.8687682	-0.6095468
H	-0.5056705	1.1977541	-1.5569008
C	0.9720268	3.4144512	0.5615298
H	1.4153718	4.4090756	0.5647453
O	-0.3903086	-1.2597558	1.8041789
H	0.5819866	-1.4848315	1.7214506
C	-0.0845325	-1.4793032	-0.6129711
C	-0.8341164	-1.4946811	-1.9593987
H	-0.0454030	-2.5032754	-0.2058541
C	0.2692138	-1.7400577	-2.9955353
H	-1.3357430	-0.5353467	-2.1536114
H	-1.6128085	-2.2672417	-1.9683837
C	1.4427116	-0.9267255	-2.4503466
H	0.5350664	-2.8072255	-3.0267793
H	-0.0190403	-1.4247826	-4.0060824
H	2.4260197	-1.2897180	-2.7710633

H	1.3612734	0.1348629	-2.7347487
N	1.2948282	-1.0819777	-0.9853478
C	2.3186649	-1.1601649	-0.1022389
O	2.1472777	-1.5326169	1.0868163
C	3.6602942	-0.6793958	-0.5422754
C	3.7558029	0.6731029	-0.8936147
C	4.8641632	-1.4421935	-0.4028705
N	4.8955237	1.3539628	-1.0850780
H	2.8310549	1.2509128	-0.9902065
C	6.0711040	-0.7032653	-0.5618010
C	6.0300023	0.6398537	-0.8970053
H	7.0391337	-1.1879702	-0.4461741
H	6.9684614	1.1850982	-1.0347247
C	3.7492474	-3.6959302	-0.3143633
C	6.1609350	-3.5211152	-0.0220997
C	4.4049544	-5.0524448	-0.5853388
H	3.0999447	-3.3669233	-1.1404753
H	3.1457501	-3.7156035	0.6068870
C	5.7103404	-4.9640858	0.2164811
H	6.7559077	-3.1146232	0.8094986
H	6.7591604	-3.4385196	-0.9469324
H	3.7604443	-5.8863967	-0.2786778
H	4.6237160	-5.1604822	-1.6588849
H	5.5124171	-5.1277444	1.2869176
H	6.4692087	-5.6881532	-0.1067546
N	4.8936643	-2.7776140	-0.1689431

C	-5.2056784	1.4656325	-0.5145874
C	-4.8917101	-3.3315418	1.0155300
C	1.5201843	3.1785346	3.0037889
C	0.5000832	3.6278922	-1.9106559
F	2.7707678	2.6593073	3.2092954
F	1.6535086	4.5321226	2.9995442
F	0.7805984	2.8500834	4.0999516
F	-0.7407690	3.7774210	-2.4614565
F	1.2557708	2.9641549	-2.8424990
F	1.0332056	4.8673846	-1.7770739
F	-4.9317728	1.7731882	-1.8201775
F	-6.5469811	1.2781895	-0.4238790
F	-4.9042920	2.5834437	0.2114332
F	-4.2679084	-4.3756511	0.3940727
F	-6.2056061	-3.3908313	0.6742565
F	-4.8174730	-3.5831029	2.3584135

3c-A

Energy = -3527.23976232188

C	-0.3951779	-0.4157087	5.9383935
C	-0.6190837	0.9355869	8.3877197
C	-0.9868480	0.8650954	5.9921460
C	-0.0111927	-1.0779217	7.1294924
C	-0.1216405	-0.3666842	8.3345400
C	-1.0704677	1.5409194	7.2209923
H	0.1747579	-0.8673324	9.2588956

H	-1.5153056	2.5358616	7.2403049
H	-0.6856502	1.4624757	9.3406526
N	-0.1640375	-1.0279614	4.6744029
C	-1.1057163	-1.1962349	3.6568898
C	0.9760580	-1.7108458	4.3209195
H	1.8551713	-1.6824496	4.9530345
C	0.7749629	-2.3196703	3.0926084
H	1.5213661	-2.8718234	2.5304600
C	-0.5408188	-2.0189848	2.6824859
H	-1.0230531	-2.2952389	1.7503048
C	-2.3493587	-0.4754427	3.5186617
C	-1.6197481	1.5773459	4.8515246
O	-2.4278397	0.8480697	3.9688121
O	-1.7004791	2.7822895	4.7623075
O	-3.3137393	-0.8824770	2.8923172
N	1.0023715	1.5363688	3.4632724
C	1.1351988	1.0974593	2.2041235
C	2.1309505	1.5058117	4.2038229
C	2.3176509	0.6326293	1.6189231
H	0.2213393	1.0931586	1.6028424
C	3.3650845	1.0871010	3.7373511
H	2.0329814	1.8357421	5.2429307
C	3.5223563	0.6470208	2.3902637
H	4.2137456	1.0939627	4.4189868
C	0.6016768	-0.4718450	-2.4769582
C	0.2652107	-0.3607825	-3.9701096

C	1.0639278	-1.0432567	-4.8942194
C	-0.7780081	0.4435288	-4.4407596
C	0.8255726	-0.9081987	-6.2653017
H	1.8674358	-1.6841426	-4.5317802
C	-1.0069365	0.5719699	-5.8127539
H	-1.4280790	0.9669496	-3.7394751
C	-0.2074710	-0.0995671	-6.7400631
H	-0.3915841	-0.0001371	-7.8092552
C	-0.6595675	-0.2777228	-1.6190688
C	-1.4681062	-1.3982287	-1.3859929
C	-1.0617960	0.9507516	-1.0873530
C	-2.6449912	-1.2862357	-0.6433926
H	-1.1589239	-2.3619178	-1.7916777
C	-2.2301417	1.0497893	-0.3204093
H	-0.4756189	1.8514614	-1.2589911
C	-3.0362546	-0.0633512	-0.0925620
H	-3.9388916	0.0141160	0.5093806
O	1.1071203	-1.7917914	-2.2829783
H	1.5598337	-1.7495726	-1.3879569
C	1.7617837	0.5614833	-2.1745415
C	1.7146151	1.9110250	-2.9163799
H	2.6730463	0.0172453	-2.4767055
C	2.5412012	2.8450389	-2.0287356
H	0.6827331	2.2827750	-3.0038848
H	2.1108691	1.8164837	-3.9343107
C	2.1384856	2.4218035	-0.6170603

H	3.6162649	2.6675977	-2.1825355
H	2.3350270	3.9060168	-2.2166676
H	2.9019663	2.6259485	0.1417117
H	1.2069749	2.9159182	-0.2948457
N	1.9261587	0.9597107	-0.7519075
C	2.1565979	0.0691471	0.2411698
O	2.1463167	-1.1723021	0.0547520
C	5.0615626	-0.1305610	0.5463824
C	5.9848234	0.5440280	2.6999053
C	6.5543403	-0.4684488	0.5984313
H	4.8623124	0.6825038	-0.1754782
H	4.4516070	-0.9984079	0.2687730
C	7.0994075	0.5024863	1.6514777
H	6.1092550	-0.2479060	3.4593067
H	5.9395842	1.5120642	3.2198959
H	6.6887691	-1.5088157	0.9320711
H	7.0324853	-0.3602331	-0.3838639
H	8.0572652	0.1834434	2.0820717
H	7.2359787	1.5027946	1.2123629
N	4.7526842	0.3178070	1.9169674
C	-2.0921163	1.4967067	-6.3017625
C	1.6773819	-1.6948850	-7.2296067
C	-3.4740400	-2.5270786	-0.4224936
C	-2.5865061	2.3957461	0.2583357
F	-4.6821212	-2.2650062	0.1360319
F	-3.7089149	-3.1908693	-1.5940803

F	-2.8387649	-3.4199575	0.4004509
F	-1.5932300	2.8693023	1.0723011
F	-3.7262038	2.3739270	0.9899615
F	-2.7538487	3.3363609	-0.7226203
F	-1.6113032	2.7612383	-6.5204585
F	-2.6375481	1.0795313	-7.4767627
F	-3.1096237	1.6198797	-5.4046240
F	1.5849782	-1.2353134	-8.5060978
F	1.3167545	-3.0152578	-7.2575227
F	2.9972785	-1.6642368	-6.8862802
C	0.3930176	-2.5384108	7.1967480
C	-0.7193693	-3.4900980	6.7256887
H	0.6350634	-2.7611191	8.2461920
H	1.3117888	-2.7395329	6.6303339
H	-0.4076923	-4.5345055	6.8687081
H	-1.6427675	-3.3257750	7.3003400
H	-0.9529235	-3.3530377	5.6602732

3c_B

Energy= -3527.24271830272

N	2.1269406	4.5697719	0.0364269
C	2.9546619	5.7692466	-0.2416522
C	1.9277447	6.8593742	-0.5515650
C	0.7242412	6.4536473	0.3074034
C	0.6967916	4.9330898	0.1498730
C	2.6682769	3.3734350	0.3034574

C	1.9010414	2.1691353	0.5243453
C	2.5387367	1.0194832	0.9275292
N	3.8888788	0.9571042	1.0763056
C	4.6520859	2.0554834	0.8057942
C	4.0861992	3.2380523	0.4214086
C	4.4852913	-0.2445850	1.7441979
O	3.9583735	-0.5979486	2.7836928
C	0.4613011	2.0508359	0.1139183
O	0.1740593	2.2299351	-1.0913699
C	5.8572363	-0.6332511	1.3094760
C	6.3159126	-0.6184223	-0.0286494
C	7.5983608	-1.1110421	-0.3500845
C	8.4231005	-1.5475792	0.7004134
C	7.9904315	-1.5394338	2.0261640
C	6.7035755	-1.1015433	2.3264415
N	5.4747246	-0.1322666	-1.0787851
C	4.3120565	-0.7369713	-1.5626138
C	3.9182860	-0.0310830	-2.6894657
C	4.8451791	1.0300435	-2.8916112
C	5.8047364	0.9338603	-1.9021073
C	3.6298462	-1.8803165	-0.8805426
O	3.5465722	-1.8176871	0.4000890
O	3.1612149	-2.8016999	-1.5954013
N	-0.4452236	1.7186039	1.0552394
C	-1.9034201	1.6679083	0.7600854
C	-2.5387000	1.4578955	2.1463180

C	-1.5310896	2.0874237	3.1135651
C	-0.1828364	1.6889390	2.5158737
C	-2.3273240	0.6756652	-0.4037481
O	-2.2820488	1.3865835	-1.6407221
C	-3.8019755	0.3071875	-0.1991111
C	-4.7786004	1.2406543	-0.5755922
C	-6.1300551	0.9715277	-0.3584268
C	-6.5411406	-0.2306489	0.2262979
C	-5.5671377	-1.1581810	0.5898639
C	-4.2079464	-0.8963301	0.3794168
C	-7.1676853	1.9634340	-0.8215750
F	-7.5607098	1.7149215	-2.1102658
C	-5.9491856	-2.4605184	1.2453324
F	-5.3391655	-3.5252771	0.6446564
C	-1.4252789	-0.5652749	-0.4755825
C	-1.0680270	-1.0687552	-1.7310337
C	-0.2299701	-2.1807436	-1.8395777
C	0.2830878	-2.8133138	-0.7055205
C	-0.0873192	-2.3207739	0.5446852
C	-0.9363547	-1.2151899	0.6624944
C	0.1375118	-2.6604905	-3.2207647
F	1.0140310	-1.8156477	-3.8429992
C	0.3961981	-2.9725335	1.8133835
F	0.9454547	-2.0606600	2.6725699
F	-5.5659701	-2.4911816	2.5595334
F	-7.2859174	-2.6953904	1.2174296

F	-6.7074169	3.2446549	-0.7963636
F	-8.2943025	1.9270345	-0.0578835
F	0.6959115	-3.8960192	-3.2248564
F	-0.9657539	-2.7217112	-4.0345051
F	1.3144054	-3.9414360	1.6058021
F	-0.6460780	-3.5530420	2.4988922
H	9.4213495	-1.9204788	0.4588315
H	6.3331674	-1.1121292	3.3520509
H	8.6505743	-1.8870625	2.8224941
H	6.6883681	1.5317265	-1.7021529
H	4.8098045	1.7935477	-3.6645447
H	3.0289224	-0.2551284	-3.2716413
H	2.0194569	0.0782178	1.1044272
H	5.7243412	1.9475061	0.9555670
H	4.7466155	4.0823555	0.2435844
H	-4.4703661	2.1745092	-1.0439932
H	-3.4693689	-1.6479563	0.6604598
H	-7.5977097	-0.4385823	0.3915634
H	-1.4462170	-0.5717792	-2.6242844
H	-1.2023005	-0.8689491	1.6601739
H	0.9865591	-3.6370506	-0.7984568
H	-1.3770263	1.8079970	-1.6473244
H	-2.1961332	2.6593856	0.3761181
H	-2.6687828	0.3892495	2.3711889
H	-3.5315771	1.9199762	2.1970167
H	-1.6332444	3.1827539	3.1161155

H	-1.6460704	1.7265427	4.1431154
H	0.6341638	2.3740594	2.7703498
H	0.1100802	0.6738239	2.8274986
H	3.6426474	5.5715118	-1.0751941
H	3.5473661	6.0192435	0.6539454
H	0.2447042	4.4219276	1.0110585
H	0.1588214	4.6330212	-0.7617148
H	2.3141031	7.8579878	-0.3129358
H	1.6614438	6.8372141	-1.6187727
H	0.8938256	6.7240334	1.3604329
H	-0.2183193	6.9088298	-0.0212804
C	8.0735635	-1.2843987	-1.7761078
C	7.4390794	-2.5124535	-2.4537414
H	9.1677736	-1.3984497	-1.7618113
H	7.8556412	-0.3914187	-2.3769140
H	7.8260439	-2.6261086	-3.4769115
H	7.6694156	-3.4318555	-1.8952147
H	6.3456755	-2.4135749	-2.5120187

3c-C

Energy= -3603.64581284458

N	1.8034543	4.6768245	-0.3218743
C	2.6662241	5.8524325	-0.6060558
C	1.6720166	6.9664481	-0.9345985
C	0.4492155	6.6024358	-0.0848041
C	0.3818514	5.0812696	-0.2214095

C	2.3033258	3.4604760	-0.0913237
C	1.4969693	2.2793460	0.1269745
C	2.0996479	1.0934445	0.4508742
N	3.4596928	0.9619806	0.5431617
C	4.2565576	2.0504272	0.2818457
C	3.7199114	3.2668806	-0.0178607
C	4.0133395	-0.3088657	1.0173264
O	3.2979587	-1.0259439	1.6926263
C	0.0236203	2.2078637	-0.1822516
O	-0.3403519	2.2954687	-1.3744158
C	5.4351545	-0.5766698	0.6937959
C	5.8914080	-0.6061554	-0.6408960
C	7.2368330	-0.8777577	-0.9335040
C	8.1003046	-1.1191032	0.1479666
C	7.6486138	-1.1293270	1.4697956
C	6.3062611	-0.8793424	1.7484889
N	4.9383985	-0.3769294	-1.6822443
C	3.7846124	-1.1400094	-1.9090833
C	2.9921664	-0.4158313	-2.7947647
C	3.6640850	0.7929272	-3.1107873
C	4.8601936	0.7952615	-2.4093058
C	3.6029348	-2.5517877	-1.4271951
O	4.6290282	-3.1178569	-0.9125767
O	2.4641697	-3.0611572	-1.6002207
N	-0.8137785	1.9873299	0.8495121
C	-2.2904635	1.9263594	0.6721821

C	-2.8184658	1.8767352	2.1174918
C	-1.7311818	2.5809839	2.9353884
C	-0.4376879	2.0971723	2.2817360
C	-2.7984314	0.8130677	-0.3368137
O	-2.8379520	1.3758293	-1.6478849
C	-4.2545344	0.4734556	0.0122150
C	-5.2716863	1.3228557	-0.4492255
C	-6.6013520	1.0710552	-0.1123755
C	-6.9507555	-0.0239278	0.6854763
C	-5.9380685	-0.8655018	1.1370264
C	-4.6003054	-0.6258969	0.7996982
C	-7.7040820	1.9459093	-0.6527716
F	-8.3467510	1.3491033	-1.7046280
C	-6.2520776	-2.0502124	2.0146737
F	-5.7459310	-3.2102563	1.4998052
C	-1.9136779	-0.4435415	-0.3300093
C	-1.6881925	-1.1135298	-1.5370639
C	-0.8910410	-2.2597509	-1.5765168
C	-0.2841446	-2.7594867	-0.4233897
C	-0.5258165	-2.1018101	0.7829462
C	-1.3366453	-0.9626010	0.8342022
C	-0.6968308	-2.9425098	-2.9069300
F	0.0825449	-2.2076149	-3.7559034
C	0.0284657	-2.6411261	2.0760591
F	0.4465058	-1.6378116	2.9064331
F	-5.6980096	-1.9103394	3.2584279

F	-7.5845110	-2.2385601	2.1916220
F	-7.2512347	3.1465196	-1.0993056
F	-8.6599102	2.1991937	0.2873111
F	-0.1357458	-4.1695056	-2.7989589
F	-1.8972247	-3.1114493	-3.5515800
F	1.0731126	-3.4821799	1.8975288
F	-0.9294417	-3.3356702	2.7738897
O	4.2645283	-3.9263204	1.6408043
H	9.1518687	-1.3308000	-0.0617503
H	5.9329824	-0.8850674	2.7732925
H	8.3444543	-1.3365012	2.2842700
H	5.6437012	1.5436816	-2.3365257
H	3.3200000	1.5841268	-3.7720596
H	2.0415987	-0.7691871	-3.1827211
H	4.4062766	-3.7127781	0.6734845
H	3.8381844	-3.1087852	1.9576033
H	1.5362498	0.1773511	0.6231907
H	5.3278221	1.8982992	0.3904744
H	4.4059562	4.0941761	-0.1777651
H	-5.0122598	2.1728664	-1.0785877
H	-3.8317844	-1.3153037	1.1506125
H	-7.9913876	-0.2153075	0.9458208
H	-2.1416842	-0.7227744	-2.4479795
H	-1.5056167	-0.4903023	1.8004682
H	0.3821844	-3.6175972	-0.4741697
H	-1.9423362	1.7955810	-1.7689767

H	-2.6073857	2.8724224	0.2025736
H	-2.9450302	0.8402816	2.4625913
H	-3.7985903	2.3620306	2.1930935
H	-1.8189273	3.6729379	2.8332326
H	-1.7704404	2.3293350	4.0022330
H	0.4045181	2.7880394	2.4047830
H	-0.1298063	1.1135223	2.6702776
H	3.3535402	5.6260092	-1.4320484
H	3.2573694	6.0903141	0.2932063
H	-0.0892300	4.5971756	0.6439433
H	-0.1538312	4.7802153	-1.1339768
H	2.0837514	7.9554505	-0.6989127
H	1.4171404	6.9431352	-2.0043273
H	0.6133046	6.8812746	0.9666635
H	-0.4773119	7.0770207	-0.4303334
C	7.7321009	-1.0365258	-2.3504073
C	7.5649205	-2.4853791	-2.8423487
H	8.7941047	-0.7495197	-2.3893099
H	7.1919064	-0.3589809	-3.0254111
H	7.8762963	-2.5745296	-3.8932692
H	8.1775114	-3.1741774	-2.2415775
H	6.5172082	-2.8056489	-2.7497168

TS_3c_1

Energy = -3527.2360025187158

N	2.7101306	5.0498661	-0.2781004

C	3.7207563	6.0608433	-0.6609611
C	2.8838881	7.2625583	-1.1038028
C	1.6330545	7.1548575	-0.2221402
C	1.3624473	5.6485757	-0.2011796
C	3.0533649	3.8064723	0.1206485
C	2.1096413	2.7761653	0.4547301
C	2.5748393	1.5539972	0.9263274
N	3.8778227	1.2593919	1.0414995
C	4.7849134	2.2021449	0.7140603
C	4.4278282	3.4515582	0.2614729
C	4.5182048	-0.3007047	1.9402289
O	4.5625149	-0.0251291	3.1365847
C	0.6562296	2.8559689	0.0964510
O	0.3358698	2.8961073	-1.1144921
C	5.7646529	-0.6507146	1.1537676
C	5.8001362	-0.8328050	-0.2426167
C	6.9650402	-1.3130766	-0.8854538
C	8.1080592	-1.5069442	-0.0941177
C	8.1034310	-1.2712156	1.2812614
C	6.9271723	-0.8626598	1.9047697
N	-0.2514318	2.8290724	1.0976741
C	-1.7053365	3.0312750	0.8441807
C	-2.2893831	3.2319117	2.2552006
C	-1.1176610	3.7999488	3.0630234
C	0.0817359	3.0176395	2.5308031
C	-2.3940821	1.9551913	-0.0911348

O	-2.2293955	2.3598494	-1.4482620
C	-3.9059155	1.9847398	0.1768566
C	-4.6922782	2.9373119	-0.4810397
C	-6.0649510	3.0136304	-0.2284807
C	-6.6809599	2.1527279	0.6816270
C	-5.8915440	1.2065034	1.3360135
C	-4.5202575	1.1145782	1.0820636
C	-6.8712410	4.0401184	-0.9834343
F	-6.8696505	3.7916068	-2.3286090
C	-6.4995518	0.2337520	2.3135368
F	-6.4883072	-1.0459085	1.8275580
C	-1.8484292	0.5321968	0.0972257
C	-1.8479284	-0.3315728	-1.0039253
C	-1.4147131	-1.6532548	-0.8691288
C	-0.9579585	-2.1433656	0.3557095
C	-0.9557885	-1.2803737	1.4495712
C	-1.3983370	0.0404809	1.3263877
C	-1.4200929	-2.5328894	-2.0932117
F	-0.5161020	-2.0965867	-3.0271958
C	-0.4599268	-1.7284339	2.7995402
F	0.6256523	-0.9983697	3.1974241
F	-5.8009047	0.2007345	3.4884947
F	-7.7876395	0.5304173	2.6211626
F	-6.3614073	5.2985077	-0.8259652
F	-8.1708249	4.0844435	-0.5903636
F	-1.1090179	-3.8249109	-1.8162190

F	-2.6368699	-2.5361214	-2.7136891
F	-0.1095824	-3.0344528	2.8360935
F	-1.4157530	-1.5426213	3.7663169
H	9.0171698	-1.8736996	-0.5763438
H	6.8813416	-0.7052408	2.9828109
H	9.0094491	-1.4296690	1.8687166
H	1.8752320	0.7590495	1.1949925
H	5.8343681	1.9237999	0.8280565
H	5.2156793	4.1624165	0.0236369
H	-4.2226776	3.6092354	-1.1993342
H	-3.9347676	0.3518644	1.5963062
H	-7.7506224	2.2165600	0.8750581
H	-2.1891659	0.0456418	-1.9681843
H	-1.3941777	0.6723599	2.2126155
H	-0.5997650	-3.1650304	0.4543491
H	-1.2615883	2.6056809	-1.5208388
H	-1.8115627	3.9709436	0.2762944
H	-2.6285001	2.2811926	2.6904047
H	-3.1588611	3.8995544	2.2285939
H	-0.9902911	4.8724335	2.8517064
H	-1.2453198	3.6745108	4.1452756
H	1.0371241	3.5455645	2.6318007
H	0.1827089	2.0401993	3.0305462
H	4.3705152	5.6726177	-1.4586483
H	4.3499408	6.3112051	0.2107990
H	0.8549357	5.3259829	0.7197257

H	0.7518632	5.3377971	-1.0630909
H	3.4271224	8.2070090	-0.9732001
H	2.6125458	7.1641830	-2.1657811
H	1.8500169	7.5150103	0.7949429
H	0.7750139	7.7161800	-0.6131875
C	7.0121947	-1.7279514	-2.3426460
C	6.0907561	-2.9176750	-2.6602769
H	8.0513867	-2.0047470	-2.5734698
H	6.7682496	-0.8908617	-3.0111242
H	6.2178957	-3.2259906	-3.7079114
H	6.3285463	-3.7780980	-2.0176214
H	5.0314344	-2.6651073	-2.5105564
N	4.6309229	-0.5360434	-1.0059179
C	4.5965744	0.2017113	-2.1681656
C	3.3618112	-1.0649228	-0.7796595
C	3.3143527	0.1461068	-2.6919826
H	5.4816903	0.7174037	-2.5231315
C	2.5408743	-0.6681186	-1.8317547
H	2.9750243	0.6578471	-3.5880418
H	1.4831025	-0.8987350	-1.9115853
C	2.9199717	-1.7141891	0.4497734
O	3.4136601	-1.2983491	1.6501767
O	2.0292840	-2.5607469	0.4597250

TS_3c_2

Energy= -3603.6323334241715

N	2.5553893	4.8174743	0.2759794
C	3.4471804	6.0013721	0.2455734
C	2.5164128	7.1499230	-0.1465256
C	1.1708080	6.7248558	0.4540668
C	1.1368784	5.2215269	0.1742963
C	3.0108757	3.5810789	0.5267924
C	2.1920004	2.3980398	0.5212637
C	2.7286315	1.1843276	0.9003945
N	4.0285480	1.0485040	1.2496886
C	4.8488598	2.1380609	1.2164080
C	4.3876153	3.3742708	0.8578226
C	4.6758472	-0.2343269	1.8328576
O	4.7632903	-0.2394606	3.0549616
C	0.8209304	2.3451675	-0.0885512
O	0.6920978	2.6075230	-1.3044948
C	5.7284131	-0.8332854	0.9303306
C	5.6535295	-0.9597766	-0.4768685
C	6.7145624	-1.5308094	-1.2065187
C	7.8410362	-1.9868736	-0.5028833
C	7.9213227	-1.8812214	0.8836357
C	6.8691600	-1.3074321	1.5944694
N	4.5172095	-0.4769014	-1.2155899
C	3.4937700	-1.2536394	-1.7739795
C	2.7920580	-0.4360455	-2.6531978
C	3.4026801	0.8427216	-2.6526667
C	4.4811242	0.7814556	-1.7868948

C	3.2786314	-2.6980376	-1.4799359
O	3.6436054	-3.1314854	-0.3088116
O	2.7679247	-3.4157913	-2.3652440
N	-0.2036877	1.9545065	0.7023605
C	-1.6131210	1.9732297	0.2188774
C	-2.4366084	1.8130506	1.5113959
C	-1.5286620	2.3851331	2.6037417
C	-0.1401217	1.9057035	2.1847203
C	-1.9463012	0.9982453	-0.9783485
O	-1.5946259	1.6359166	-2.2056553
C	-3.4733205	0.8266417	-1.0168987
C	-4.2468924	1.7982916	-1.6635081
C	-5.6402722	1.6994476	-1.6679212
C	-6.2911212	0.6386408	-1.0332139
C	-5.5152503	-0.3270102	-0.3932610
C	-4.1192411	-0.2421815	-0.3903746
C	-6.4388653	2.7310023	-2.4241124
F	-6.4329625	2.4829442	-3.7711313
C	-6.1569031	-1.4897301	0.3194112
F	-5.7061389	-2.6871939	-0.1609579
C	-1.2639711	-0.3773122	-0.8988454
C	-1.0842039	-1.0810973	-2.0958340
C	-0.5454731	-2.3697671	-2.0925516
C	-0.1509316	-2.9810891	-0.9015513
C	-0.3295264	-2.2794823	0.2914041
C	-0.8885465	-0.9964054	0.2974825

C	-0.4442916	-3.0995675	-3.4099349
F	0.3469664	-2.4408232	-4.3067444
C	-0.0198119	-2.9447943	1.6078072
F	0.3524004	-2.0486777	2.5675346
F	-5.8634227	-1.4808855	1.6566051
F	-7.5097923	-1.4973777	0.2092082
F	-5.9346965	3.9875991	-2.2618544
F	-7.7425958	2.7685545	-2.0391469
F	0.0341226	-4.3593622	-3.2888740
F	-1.6794012	-3.2028850	-4.0035307
F	0.9663759	-3.8709737	1.5111866
F	-1.1234126	-3.5964547	2.1030389
O	3.1734223	-1.4686988	1.4183562
H	8.6704576	-2.4248876	-1.0636020
H	6.9169002	-1.2085693	2.6792579
H	8.8079004	-2.2353412	1.4126553
H	5.2301537	1.5228708	-1.5268077
H	3.0786734	1.7254347	-3.1964765
H	1.9170126	-0.7499344	-3.2136152
H	3.1578850	-1.9294459	2.2772241
H	3.4418829	-2.2323858	0.6419064
H	2.1543739	0.2603036	0.8982607
H	5.8891988	1.9712300	1.4936119
H	5.0947350	4.1997510	0.8560937
H	-3.7478942	2.6250232	-2.1680897
H	-3.5374569	-1.0229990	0.1010432

H	-7.3777506	0.5664813	-1.0389323
H	-1.3758660	-0.6065342	-3.0330922
H	-1.0336537	-0.5012874	1.2545354
H	0.2923042	-3.9751720	-0.9072071
H	-0.6992179	2.0499641	-2.0396387
H	-1.7993967	2.9751040	-0.2046935
H	-2.6597667	0.7562128	1.7163733
H	-3.3966153	2.3363586	1.4316976
H	-1.5637854	3.4849189	2.5962905
H	-1.7990368	2.0375769	3.6082889
H	0.6757084	2.5387511	2.5515633
H	0.0538210	0.8757406	2.5259974
H	4.2654003	5.8446957	-0.4711498
H	3.8811345	6.1600547	1.2471597
H	0.5324633	4.6656415	0.9049884
H	0.7527713	5.0062029	-0.8341382
H	2.8774265	8.1124057	0.2363152
H	2.4413595	7.2196387	-1.2420809
H	1.1584408	6.9098803	1.5387737
H	0.3115940	7.2381622	0.0045764
C	6.6767455	-1.6866423	-2.7104179
C	6.4607418	-3.1431636	-3.1548646
H	7.6361890	-1.3265248	-3.1149262
H	5.8930042	-1.0491469	-3.1420440
H	6.4817917	-3.2139977	-4.2522792
H	7.2484319	-3.8005778	-2.7573482

H 5.4904137 -3.5237374 -2.8072211

4c

Energy= -896.29840831737

C	-0.7013116	0.7694049	-0.0907594
C	-3.4223721	0.5149698	-0.6684170
C	-1.3428584	-0.4737257	0.0907629
C	-1.4097764	1.8943788	-0.5476174
C	-2.7747469	1.7368047	-0.8412837
C	-2.7128497	-0.5848420	-0.1876090
H	-3.3347565	2.6000017	-1.2092683
H	-3.2118873	-1.5430059	-0.0439391
H	-4.4830213	0.4178641	-0.9060144
N	0.7038541	0.9051582	0.1770253
C	1.6532596	0.9245115	-0.8092934
H	1.3589807	0.8330785	-1.8506879
C	1.3409051	1.0132940	1.4140938
C	2.9087893	1.0530132	-0.2252876
H	3.8535552	1.1005142	-0.7598671
C	2.7140111	1.1090666	1.1700721
H	3.4750303	1.2135377	1.9381111
C	0.6243867	1.0249062	2.6766705
C	-0.5922102	-1.6787427	0.5508350
O	-0.5956411	0.9601485	2.8193077
O	0.5802863	-1.9252445	0.3075229

O	1.4785371	1.1204468	3.7394558
H	0.9254639	1.1281352	4.5487060
O	-1.3797890	-2.5150019	1.2756133
H	-0.8442908	-3.3081408	1.4931796
C	-0.7788817	3.2630512	-0.6529999
C	-0.9194742	4.0637312	0.6541696
H	-1.2697087	3.8112160	-1.4714742
H	0.2843108	3.1810846	-0.9173189
H	-0.4549531	5.0546635	0.5487003
H	-1.9782741	4.2056008	0.9169480
H	-0.4329875	3.5427438	1.4908451

4c-A

Energy= -1663.73516566538

C	3.1469241	-0.3884422	0.1102647
C	4.9139470	1.2082076	1.5631348
C	2.6471999	0.6562012	0.9131251
C	4.5258224	-0.6362440	0.0075448
C	5.3931029	0.1735518	0.7604457
C	3.5426452	1.4595145	1.6310985
H	6.4690450	-0.0071118	0.6957244
H	3.1565573	2.2720209	2.2477969
H	5.6103236	1.8289208	2.1298104
N	2.2392475	-1.1301252	-0.7249307
C	1.9079130	-0.7282627	-1.9931707
H	2.3390041	0.1786448	-2.4066534

C	1.5625994	-2.3127117	-0.4384857
C	1.0206363	-1.6501384	-2.5392934
H	0.5883466	-1.6000506	-3.5349775
C	0.8012658	-2.6435561	-1.5623689
H	0.1542342	-3.5123952	-1.6391800
C	1.6407132	-2.9704663	0.8534078
C	1.1734586	0.8703399	1.0629916
O	2.3729435	-2.6510838	1.7869297
O	0.8295136	2.1673057	1.1074694
O	0.7829370	-4.0324575	0.9296020
O	0.3660221	-0.0469683	1.1653754
N	-1.8909704	2.2441984	1.1586910
C	-2.4527771	1.3369786	1.7886081
N	-2.9308649	0.5055027	2.5405961
C	-2.5681268	3.1501669	0.1974898
C	-3.2048991	-0.9328599	2.2768484
C	-1.7540389	3.2204297	-1.0854699
C	-1.5567993	4.4536584	-1.7217982
C	-1.2340589	2.0591270	-1.6752449
C	-0.8590623	4.5265406	-2.9326508
H	-1.9500338	5.3658850	-1.2647516
C	-0.5366066	2.1297236	-2.8835824
H	-1.3683900	1.0948427	-1.1818311
C	-0.3470090	3.3633525	-3.5175524
H	-0.7095349	5.4946773	-3.4153100
H	-0.1361773	1.2169030	-3.3293678

H	0.2011434	3.4179540	-4.4603829
C	-3.4979487	-1.1837365	0.8068129
C	-2.4708805	-1.2768295	-0.1465085
C	-4.8310709	-1.2743359	0.3790040
C	-2.7785245	-1.4497268	-1.4993863
H	-1.4263073	-1.1982641	0.1597216
C	-5.1388105	-1.4483088	-0.9742865
H	-5.6366375	-1.2012633	1.1145818
C	-4.1106296	-1.5345192	-1.9188468
H	-1.9682149	-1.5171574	-2.2282775
H	-6.1819746	-1.5166284	-1.2900380
H	-4.3461096	-1.6683523	-2.9767679
C	-4.0174787	2.7376743	-0.0790705
H	-4.0519874	1.7336020	-0.5289712
H	-4.6087763	2.7315888	0.8481214
H	-4.4795769	3.4474336	-0.7787175
H	-2.5602305	4.1497788	0.6609133
H	-0.1924844	2.2149546	1.1773324
C	-2.0673344	-1.7803744	2.8528988
H	-1.9446787	-1.5696015	3.9244193
H	-2.3084570	-2.8457925	2.7314318
H	-1.1191146	-1.5679680	2.3431063
H	-4.1204129	-1.1492327	2.8471171
H	0.9126953	-4.4387818	1.8118908
C	5.0893958	-1.7490848	-0.8439671
C	5.4665226	-2.9875889	-0.0125919

H	5.9854845	-1.3738584	-1.3629641
H	4.3677955	-2.0349374	-1.6218796
H	4.5899989	-3.3856572	0.5172511
H	5.8709867	-3.7771762	-0.6623860
H	6.2300393	-2.7395382	0.7393641

4c-B

Energy= -1663.74363439549

C	2.9777589	-0.1355848	-0.0017592
C	4.8066651	-0.5730474	-2.0819142
C	2.5807048	0.0534664	-1.3486044
C	4.2928977	-0.5307711	0.3087622
C	5.1834823	-0.7612087	-0.7516765
C	3.5154766	-0.1480528	-2.3778327
H	6.2033468	-1.0766656	-0.5180110
H	3.2034976	0.0134073	-3.4102675
H	5.5212311	-0.7532046	-2.8865060
N	2.0590508	0.0927181	1.0722028
C	1.4864676	-0.9137743	1.8235719
H	1.8074750	-1.9428610	1.6989258
C	1.3800572	1.2805149	1.3262591
C	0.4763884	-0.3660548	2.5949524
H	-0.1489680	-0.9113744	3.2957340
C	0.3993664	1.0132732	2.2730694
H	-0.2665757	1.7554579	2.7050399
C	1.7248178	2.5536996	0.6641933

C	1.2254269	0.4830374	-1.7912203
O	0.6814217	3.2633015	0.2186462
O	0.2341284	0.0583233	-0.9320653
O	2.8843063	2.9540874	0.5328252
O	0.9871150	1.0958266	-2.8139849
C	-1.0505546	0.5768804	-1.0854248
N	-1.2757522	1.7676370	-0.6424592
N	-1.9090382	-0.2998446	-1.6458239
C	-1.5921919	-1.7099500	-1.9452626
H	-2.8984023	-0.0634335	-1.5697749
C	-2.6426692	2.3146068	-0.7410983
C	-1.2897630	-2.5207775	-0.6921704
C	-0.1911493	-3.3885754	-0.6710855
C	-2.1102947	-2.4334257	0.4408344
C	0.0810789	-4.1650541	0.4599126
H	0.4647218	-3.4481961	-1.5440532
C	-1.8374672	-3.2024721	1.5742103
H	-2.9555765	-1.7409913	0.4533989
C	-0.7432329	-4.0748348	1.5861376
H	0.9454774	-4.8325925	0.4645450
H	-2.4774373	-3.1153029	2.4550829
H	-0.5269970	-4.6724968	2.4740690
H	-2.9505294	2.3024650	-1.8040544
H	-0.1440662	2.6284100	-0.0384328
C	-3.6580765	1.4869429	0.0435326
C	-4.8873652	1.1378971	-0.5360129

C	-3.3778998	1.0576704	1.3503045
C	-5.8187326	0.3706012	0.1743905
H	-5.1151684	1.4629416	-1.5553825
C	-4.3061600	0.2940895	2.0610636
H	-2.4125259	1.2983413	1.7992348
C	-5.5298046	-0.0531950	1.4749625
H	-6.7679161	0.1001562	-0.2927638
H	-4.0699680	-0.0415365	3.0730836
H	-6.2511548	-0.6575801	2.0284059
C	4.7977617	-0.6085652	1.7308618
C	5.3058154	0.7597276	2.2199966
H	5.6156405	-1.3438661	1.7721652
H	4.0112850	-0.9676587	2.4073356
H	4.5119089	1.5177167	2.1522086
H	5.6445578	0.6932342	3.2639754
H	6.1503648	1.1051440	1.6049393
C	-2.6276725	3.7709027	-0.2663959
H	-3.6273295	4.2138579	-0.3719435
H	-2.3332661	3.8275951	0.7918317
H	-1.9165951	4.3613420	-0.8615070
C	-2.7683789	-2.2951165	-2.7316168
H	-2.9486790	-1.7196492	-3.6507853
H	-2.5508010	-3.3356876	-3.0039234
H	-3.6851015	-2.2903047	-2.1210672
H	-0.6998585	-1.7196912	-2.5885361

4c-C

Energy= -1663.7448292821441

C	3.4051146	0.6877932	-0.0287930
C	3.6048117	3.0450711	1.4833703
C	2.2652609	1.2216205	0.6180428
C	4.6563897	1.3209187	0.0996944
C	4.7247111	2.5092216	0.8440312
C	2.3818641	2.3888894	1.3917853
H	5.6915320	3.0093595	0.9394702
H	1.4949810	2.7818676	1.8898022
H	3.6887863	3.9676254	2.0597095
N	3.3049988	-0.5116701	-0.7980840
C	3.3950183	-0.5462105	-2.1793825
H	3.6912904	0.3391722	-2.7338039
C	2.8117008	-1.7312512	-0.3424665
C	2.9965194	-1.7993498	-2.6069805
H	2.9805755	-2.1400389	-3.6392481
C	2.6170219	-2.5410935	-1.4524388
H	2.2605983	-3.5671776	-1.4171575
C	2.5754770	-2.0200569	1.1095619
C	0.9150590	0.6133947	0.5898110
O	1.4536831	-2.5984616	1.3794053
O	0.6193656	0.0477813	-0.6713297
O	3.4486820	-1.6919500	1.9478920
O	0.1052150	0.6289002	1.4850502
C	-0.4929232	-0.7313192	-0.8004814

N	-0.5665657	-1.8601533	-0.1375969
N	-1.3903977	-0.2784202	-1.6731958
C	-1.3771494	1.0860209	-2.2542828
H	-2.2165619	-0.8505656	-1.8408284
C	-1.7554905	-2.7358118	-0.1892180
C	-3.0059766	-1.9961917	0.2718157
C	-4.1700283	-2.0322567	-0.5083903
C	-3.0076725	-1.2713853	1.4732625
C	-5.3218681	-1.3503595	-0.0977692
H	-4.1749300	-2.5939247	-1.4468125
C	-4.1575300	-0.5950263	1.8857545
H	-2.0964562	-1.2117193	2.0719221
C	-5.3170612	-0.6304629	1.1006586
H	-6.2195872	-1.3783927	-0.7183622
H	-4.1460931	-0.0267230	2.8179435
H	-6.2110587	-0.0912502	1.4197996
C	-1.5465357	2.1459387	-1.1765060
C	-0.6312181	3.2018122	-1.0824463
C	-2.6072327	2.0750022	-0.2622534
C	-0.7712810	4.1750043	-0.0868912
H	0.2073752	3.2534992	-1.7819675
C	-2.7468475	3.0427363	0.7337829
H	-3.3144287	1.2434430	-0.3080544
C	-1.8286460	4.0961725	0.8244077
H	-0.0453366	4.9875305	-0.0159412
H	-3.5703928	2.9703152	1.4473300

H	-1.9339986	4.8484075	1.6087682
C	-2.4708541	1.1488666	-3.3205414
H	-2.3051011	0.3880784	-4.0963424
H	-3.4648080	0.9952416	-2.8728750
H	-2.4628160	2.1387434	-3.7933942
H	-0.3973172	1.2263638	-2.7336811
C	-1.4788645	-3.9845701	0.6501964
H	-0.5899534	-4.5137259	0.2801576
H	-1.3151123	-3.7165815	1.7033518
H	-2.3425129	-4.6588081	0.5907548
H	-1.9030377	-3.0485636	-1.2381992
H	0.2981707	-2.1815794	0.4567479
C	5.9287536	0.6992712	-0.4274725
C	6.5257713	-0.2851460	0.5947827
H	6.6521152	1.5007000	-0.6403246
H	5.7454454	0.1735534	-1.3736158
H	7.4232319	-0.7701655	0.1838793
H	6.8115103	0.2389011	1.5196561
H	5.7904045	-1.0579475	0.8624222

TS_4c_1

Energy= -1663.71646600725

C	3.8850824	-0.5688775	0.3836043
C	6.0966346	0.1032630	-1.1776683
C	3.7232113	0.2898554	-0.7188823
C	5.1473405	-1.0849507	0.7342443

C	6.2410526	-0.7432788	-0.0773100
C	4.8440328	0.6345472	-1.4847768
H	7.2258748	-1.1450384	0.1735263
H	4.7182530	1.3143795	-2.3291249
H	6.9631638	0.3551887	-1.7921286
N	2.7417601	-0.9322637	1.1785899
C	2.1501783	-2.1650585	1.1231904
H	2.5591212	-2.9392055	0.4809148
C	1.9919395	-0.1152855	2.0258821
C	1.0321775	-2.1689462	1.9509194
H	0.3760767	-3.0177627	2.1225425
C	0.9276170	-0.8800264	2.5135612
H	0.1815191	-0.5282180	3.2197789
C	2.3694207	1.2480078	2.3562995
C	2.3879100	0.8818790	-1.0990158
O	3.4296273	1.8000994	2.0743025
O	2.3385737	2.1217817	-1.3471522
O	1.3798743	1.8845929	3.0583632
O	1.3928059	0.0741088	-1.1599692
N	-0.2873844	2.0877822	-1.0289600
C	-0.5207542	0.8234531	-1.1756214
N	-1.3069334	-0.0863921	-1.3266142
C	-1.2813146	3.0526318	-0.4599637
C	-1.3808813	-1.4285687	-0.7450191
C	-2.2777108	2.3564452	0.4460798
C	-1.8576869	1.7154831	1.6228696

C	-3.6360184	2.3367065	0.1067824
C	-2.7828144	1.0562377	2.4340693
H	-0.8010145	1.7121891	1.8986736
C	-4.5645475	1.6846557	0.9248977
H	-3.9672893	2.8225604	-0.8146477
C	-4.1391541	1.0400916	2.0887077
H	-2.4443687	0.5458957	3.3385392
H	-5.6184037	1.6643733	0.6408750
H	-4.8581379	0.5103715	2.7161643
C	-2.8065081	-1.7165482	-0.3066421
C	-3.9018566	-1.3764199	-1.1120186
C	-3.0340283	-2.3696419	0.9112896
C	-5.2021490	-1.6897126	-0.7083567
H	-3.7346218	-0.8449228	-2.0514314
C	-4.3340141	-2.6886075	1.3159643
H	-2.1877000	-2.6199387	1.5559057
C	-5.4223748	-2.3484340	0.5066860
H	-6.0481871	-1.4106114	-1.3403242
H	-4.4967335	-3.1910188	2.2719503
H	-6.4391947	-2.5880654	0.8249275
C	-0.4926572	4.1610035	0.2392617
H	0.0730562	3.7623927	1.0931250
H	0.2114548	4.6388917	-0.4572360
H	-1.1879680	4.9264558	0.6076950
H	-1.8287884	3.4828228	-1.3136740
H	0.7127849	2.3816997	-1.2273098

C	-0.8706212	-2.4551460	-1.7643777
H	-1.4850461	-2.4355674	-2.6757561
H	0.1738838	-2.2361089	-2.0239684
H	-0.9254709	-3.4614956	-1.3257794
H	-0.7262622	-1.4557893	0.1387980
H	1.7272369	2.7685704	3.3005891
C	5.3696437	-1.9099357	1.9814880
C	5.5412750	-1.0331407	3.2344367
H	6.2726287	-2.5220256	1.8368204
H	4.5359795	-2.6081959	2.1405649
H	5.7111621	-1.6595109	4.1221875
H	6.3989932	-0.3536109	3.1220935
H	4.6483856	-0.4172997	3.4116441

TS_4c_2

Energy= -1663.74253763693

C	2.9598515	-0.0020714	-0.0949799
C	4.6881547	-0.6056592	-2.2223625
C	2.4751845	-0.0107184	-1.4268809
C	4.3139679	-0.2815080	0.1734121
C	5.1529674	-0.5987978	-0.9064118
C	3.3582955	-0.2931314	-2.4829588
H	6.2027074	-0.8241828	-0.7028625
H	2.9764148	-0.2831286	-3.5043502
H	5.3642303	-0.8487289	-3.0434150
N	2.0962904	0.3177633	0.9997764

C	1.6477158	-0.6043984	1.9261304
H	2.0303121	-1.6197297	1.9210295
C	1.3561751	1.4884921	1.1380034
C	0.6574501	-0.0120594	2.6882369
H	0.1190541	-0.4875265	3.5030195
C	0.4647333	1.3022729	2.1851814
H	-0.2210076	2.0576624	2.5593591
C	1.5613988	2.6613028	0.2549938
C	1.0761496	0.2855271	-1.8310991
O	0.4651342	3.2096610	-0.2369899
O	0.1644635	-0.0583444	-0.8474708
O	2.6956436	3.0925742	-0.0000478
O	0.7307121	0.7289538	-2.9080778
C	-1.1480578	0.3648279	-0.9725599
N	-1.4237001	1.5948335	-0.6700250
N	-1.9937334	-0.6110885	-1.3440023
C	-1.6168793	-2.0311708	-1.5011591
H	-2.9888687	-0.4106191	-1.2402119
C	-2.8247744	2.0603122	-0.7306812
C	-1.1734413	-2.6620297	-0.1879436
C	-0.0163485	-3.4491779	-0.1489711
C	-1.9196283	-2.4872095	0.9856656
C	0.3871094	-4.0614083	1.0421260
H	0.5810814	-3.5743103	-1.0560563
C	-1.5154693	-3.0916510	2.1781064
H	-2.8106899	-1.8546367	0.9798346

C	-0.3632539	-3.8852914	2.2092707
H	1.2955108	-4.6674940	1.0600550
H	-2.0989208	-2.9376382	3.0884758
H	-0.0446530	-4.3538932	3.1426099
H	-3.1962725	1.9075272	-1.7612651
H	-0.4432439	2.4896247	-0.3613265
C	-3.7345788	1.2779906	0.2130216
C	-4.9652549	0.7814550	-0.2426511
C	-3.3535949	1.0353851	1.5420805
C	-5.7995831	0.0501424	0.6118860
H	-5.2699508	0.9617284	-1.2775439
C	-4.1854105	0.3083592	2.3962741
H	-2.3864523	1.3935839	1.8991653
C	-5.4106596	-0.1883341	1.9334641
H	-6.7503041	-0.3374213	0.2405315
H	-3.8726274	0.1184825	3.4250625
H	-6.0555600	-0.7645443	2.5997836
C	4.9067930	-0.1419287	1.5565791
C	5.3265010	1.3112727	1.8416940
H	5.7838728	-0.8023970	1.6297663
H	4.1938498	-0.4711130	2.3236516
H	4.4712179	1.9932241	1.7308918
H	5.7245914	1.4032549	2.8627017
H	6.1067283	1.6371394	1.1374057
C	-2.8607008	3.5609328	-0.4262799
H	-3.8907490	3.9328130	-0.5061378

H	-2.4989849	3.7578771	0.5934073
H	-2.2310091	4.1150479	-1.1366412
C	-2.8119961	-2.7638627	-2.1164659
H	-3.0880250	-2.3191570	-3.0830771
H	-2.5555576	-3.8190472	-2.2745111
H	-3.6815978	-2.7247031	-1.4421336
H	-0.7759176	-2.0733635	-2.2090392

TS_4c_3

Energy= -1663.73419986617

C	3.0266801	-0.1085007	-0.1374644
C	4.7105865	-0.7193626	-2.2917954
C	2.5956889	0.1147354	-1.4612350
C	4.3432144	-0.5588954	0.1222823
C	5.1524350	-0.8806664	-0.9796730
C	3.4422489	-0.1967088	-2.5312525
H	6.1656337	-1.2444703	-0.8035754
H	3.0893452	-0.0162086	-3.5471446
H	5.3637939	-0.9780199	-3.1269027
N	2.1366484	0.1153246	0.9553089
C	1.9276514	-0.8051709	1.9590172
H	2.3702346	-1.7934757	1.8979180
C	1.4368553	1.2847778	1.2748887
C	1.1089168	-0.2426989	2.9231820
H	0.7342660	-0.7503501	3.8064098
C	0.8120955	1.0723722	2.5026689

H	0.1660964	1.7936581	2.9944188
C	1.2205377	2.4370095	0.4005042
C	1.2966597	0.7558381	-1.8284818
O	1.3247319	2.2892311	-0.9206274
O	0.1542721	-0.0813255	-0.9680323
O	0.8683493	3.5326895	0.8721614
O	0.9450405	1.0375544	-2.9542382
C	-1.0825050	0.3335121	-0.9690468
N	-1.4089725	1.6102775	-1.2103872
N	-2.0400729	-0.5756084	-0.7181219
C	-1.7866771	-1.9760856	-0.3580772
H	-2.9828955	-0.2240155	-0.5606218
C	-2.7698706	2.1665832	-1.1536546
C	-1.6538211	-2.1879469	1.1460599
C	-0.8602055	-3.2447191	1.6145903
C	-2.3505536	-1.4017468	2.0723555
C	-0.7762602	-3.5213984	2.9816791
H	-0.2982871	-3.8544481	0.9017114
C	-2.2652754	-1.6736075	3.4409173
H	-2.9555680	-0.5556795	1.7397353
C	-1.4821052	-2.7366638	3.9006623
H	-0.1486912	-4.3440937	3.3311384
H	-2.8081625	-1.0444268	4.1498037
H	-1.4130299	-2.9464709	4.9700720
H	-3.4003493	1.6063370	-1.8655573
H	-0.6222390	2.2448338	-1.3733212

C	-3.3745089	2.0297771	0.2399332
C	-4.6613069	1.4964361	0.3983312
C	-2.6539419	2.4314015	1.3754252
C	-5.2221802	1.3604601	1.6744546
H	-5.2294557	1.1816238	-0.4822291
C	-3.2134441	2.2968777	2.6474615
H	-1.6446741	2.8364786	1.2639228
C	-4.4978663	1.7596511	2.8012649
H	-6.2217114	0.9357604	1.7851014
H	-2.6428596	2.6048198	3.5260673
H	-4.9285951	1.6469736	3.7979837
C	-2.7035865	3.6255177	-1.6101782
H	-2.2974468	3.6930187	-2.6291527
H	-3.7104739	4.0613059	-1.6038874
H	-2.0685625	4.2138513	-0.9308966
C	-2.9161221	-2.8352890	-0.9406535
H	-2.9672596	-2.7210160	-2.0324063
H	-2.7408119	-3.8926496	-0.7024303
H	-3.8860386	-2.5487486	-0.5045857
H	-0.8371914	-2.2540508	-0.8347041
C	4.9074085	-0.6549212	1.5285043
C	6.3936468	-0.2960766	1.6456835
H	4.7533853	-1.6754465	1.9193426
H	4.3278523	0.0041572	2.1903254
H	6.6882178	-0.2856280	2.7046427
H	7.0430688	-1.0193412	1.1329445

H	6.5959460	0.7006966	1.2258272

2c-A

Energy= -3527.24160726672

C	2.2125841	1.0565148	-0.6900742
C	4.2395607	2.0280082	-1.0572154
C	1.5500512	2.2880761	-0.6358909
H	1.6250143	0.1374191	-0.5955409
C	3.7053725	3.3039836	-1.0123059
H	5.3128846	1.8973659	-1.2284483
C	2.3153796	3.4909715	-0.7655383
H	4.3661188	4.1586274	-1.1470572
C	-2.7116844	0.6347432	-0.5872884
C	-4.1562773	0.3145715	-0.1814251
C	-4.6610197	-0.9861675	-0.0934471
C	-5.0276012	1.3894540	0.0397210
C	-5.9989665	-1.2033249	0.2477097
C	-6.3602037	1.1631935	0.3884717
H	-4.6669216	2.4129628	-0.0664812
C	-6.8608854	-0.1349131	0.4971685
H	-7.9016318	-0.3083152	0.7713246
C	-1.8101647	-0.6076876	-0.6879223
C	-1.6378936	-1.4989259	0.3813858
C	-1.0879212	-0.8404779	-1.8613487
C	-0.7622809	-2.5828218	0.2753029

H	-2.2030250	-1.3749852	1.3049140
C	-0.2013371	-1.9188193	-1.9537090
H	-1.2240678	-0.1678698	-2.7083342
C	-0.0215908	-2.7994408	-0.8877675
H	0.6752249	-3.6329789	-0.9566240
O	-2.8313638	1.2849270	-1.8550694
H	-2.0382644	1.8929219	-1.9312967
C	-2.0706311	1.6534705	0.4425151
C	-2.3727974	1.4218530	1.9384752
C	-1.0789220	1.8116782	2.6664983
H	-2.6194126	0.3708918	2.1372268
H	-3.2366113	2.0159237	2.2599299
C	0.0113830	1.3775305	1.6892054
H	-1.0320193	2.9007307	2.8165522
H	-0.9816029	1.3251773	3.6454812
H	0.9633343	1.9073451	1.8044782
H	0.2110532	0.2957582	1.7622413
N	-0.5885900	1.6852997	0.3773491
C	0.0590807	2.2455693	-0.6705351
O	-0.5554156	2.6502136	-1.6909092
C	2.6226685	5.9517532	-0.7946876
C	0.4732416	5.0611792	-0.0746530
C	1.6269384	7.0960531	-0.5925283
H	3.1041192	5.9713463	-1.7834462
H	3.4154688	5.9730755	-0.0247565
C	0.6262717	6.5058105	0.4095220

H	0.2295892	4.3668483	0.7437064
H	-0.3121491	4.9739631	-0.8424469
H	2.1189692	8.0073723	-0.2295209
H	1.1224491	7.3314610	-1.5423598
H	1.0504194	6.5219577	1.4251989
H	-0.3355858	7.0341318	0.4279058
N	1.7960387	4.7384149	-0.6407849
C	-7.2752736	2.3430805	0.5956193
C	-6.5524636	-2.6021894	0.3412460
C	-0.6377457	-3.5122493	1.4557541
C	0.5497146	-2.1088931	-3.2479582
F	-0.0048236	-2.9052588	2.5108802
F	-1.8650376	-3.8952301	1.9225860
F	0.0512999	-4.6418699	1.1719063
F	-0.3092853	-2.2779950	-4.3021520
F	1.3697583	-3.1894321	-3.2288288
F	1.3171006	-1.0219338	-3.5501551
F	-7.7355667	2.8397722	-0.5940085
F	-8.3701505	2.0274511	1.3378300
F	-6.6444766	3.3738477	1.2267920
F	-7.1446138	-2.8292878	1.5523113
F	-5.6014984	-3.5578463	0.1777332
F	-7.5141936	-2.8285962	-0.6042744
C	8.4896949	-0.3831592	0.5388639
C	7.3221169	-0.3776667	1.3174969
C	6.0876615	-0.5820654	0.6526523

C	6.0600831	-0.9110063	-0.7201847
C	7.2555589	-0.8966480	-1.4584640
C	8.4654306	-0.6122295	-0.8368843
H	9.4461269	-0.2230125	1.0411555
H	7.2134893	-1.1341070	-2.5214051
H	9.3906482	-0.6021184	-1.4148705
N	4.8643932	-0.4424008	1.3666347
C	4.5944392	0.5440498	2.2846690
C	3.8112135	-1.3571777	1.3854662
C	3.3900626	0.2606543	2.9110040
H	5.2677962	1.3832976	2.4099504
C	2.9053949	-0.9462259	2.3631215
H	2.9128740	0.8775062	3.6669988
H	1.9766353	-1.4599257	2.5914877
C	3.5595931	-2.4111948	0.4267753
O	2.8602377	-3.3845931	0.6623990
O	3.9843220	-2.2723575	-0.8919497
C	4.8658835	-1.3522594	-1.4917561
O	4.7556334	-1.2355977	-2.6925294
H	-4.0216313	-1.8422944	-0.3003454
H	-2.4319408	2.6468943	0.1316517
N	3.5253408	0.8963185	-0.8895373
C	7.4745691	-0.2721585	2.8240839
C	6.9957934	-1.5284040	3.5703725
H	6.9663044	0.6118860	3.2313667
H	8.5436660	-0.1194719	3.0307713

H	7.2049327	-1.4301895	4.6452276
H	5.9147574	-1.6895011	3.4530999
H	7.5154069	-2.4240246	3.1997241

2c_B

Energy= -3527.24294699283

C	2.2241487	1.0525508	-0.8992390
C	4.3490035	2.0662480	-0.8684382
C	1.5904477	2.2522547	-0.6885793
H	1.6856344	0.1136307	-1.0182954
C	3.7858826	3.2981636	-0.6853397
H	5.4237834	1.9246279	-0.9645694
C	2.3698113	3.4575888	-0.5481303
H	4.4517851	4.1542767	-0.6190390
C	-2.6328660	0.5462515	-0.7377069
C	-4.0611204	0.1543834	-0.3395096
C	-4.5028186	-1.1663479	-0.2575781
C	-4.9844648	1.1859022	-0.1118364
C	-5.8338169	-1.4505891	0.0773356
C	-6.3024389	0.8945292	0.2358595
H	-4.6724569	2.2259085	-0.2120879
C	-6.7439420	-0.4289706	0.3350355
H	-7.7749494	-0.6540640	0.6047692
C	-1.6792605	-0.6551825	-0.8565865
C	-1.4322530	-1.5228273	0.2196250
C	-0.9945302	-0.8758028	-2.0534599

C	-0.5078598	-2.5606867	0.1032433
H	-1.9648241	-1.4062503	1.1623295
C	-0.0631404	-1.9162673	-2.1603487
H	-1.1912417	-0.2257981	-2.9057106
C	0.1964543	-2.7634524	-1.0854152
H	0.9305115	-3.5630948	-1.1682893
O	-2.7856115	1.2099884	-1.9960738
H	-1.9991953	1.8178368	-2.0880247
C	-2.0454458	1.5803611	0.3099339
C	-2.3631516	1.3310523	1.8003422
C	-1.0924536	1.7507733	2.5525057
H	-2.5859077	0.2726808	1.9870302
H	-3.2474077	1.8997481	2.1119983
C	0.0288158	1.3568155	1.5935334
H	-1.0840146	2.8385270	2.7189345
H	-0.9959234	1.2544006	3.5263423
H	0.9552064	1.9258571	1.7322628
H	0.2785158	0.2861306	1.6662026
N	-0.5640243	1.6580430	0.2715185
C	0.0883272	2.1974783	-0.7738199
O	-0.4802968	2.5754413	-1.8250905
C	2.6799239	5.8915952	-0.2146314
C	0.4388360	4.9593611	0.0494390
C	1.6550480	7.0197913	-0.0920902
H	3.3088480	5.9790094	-1.1110335
H	3.3338285	5.8397698	0.6720856

C	0.4974681	6.3588859	0.6653950
H	0.0495860	4.2104857	0.7513532
H	-0.1750669	4.9429094	-0.8637765
H	2.0725914	7.8902594	0.4286521
H	1.3253887	7.3397296	-1.0917204
H	0.7300382	6.2896916	1.7385690
H	-0.4561102	6.8893687	0.5515816
N	1.8500491	4.6645685	-0.2858575
C	-7.2670251	2.0327841	0.4539472
C	-6.2501220	-2.8965081	0.1721795
C	-0.3572782	-3.5426166	1.2380177
C	0.6084100	-2.1352240	-3.4922707
F	-0.6221908	-2.9931723	2.4505816
F	-1.2571058	-4.5805054	1.0868440
F	0.8698625	-4.1108669	1.2928528
F	-0.2776906	-2.6465591	-4.4103455
F	1.6461871	-3.0048424	-3.4291179
F	1.0758601	-0.9722989	-4.0276876
F	-7.7510741	2.5189152	-0.7305810
F	-8.3454332	1.6678660	1.1972226
F	-6.6766486	3.0845355	1.0905510
F	-5.6085986	-3.5373973	1.1967589
F	-5.9379266	-3.5879679	-0.9644955
F	-7.5833211	-3.0502852	0.3800616
C	7.8989409	-1.7397691	0.2462232
C	7.0746193	-1.0126977	1.1221456

C	5.8754800	-0.4675348	0.6133749
C	5.4820259	-0.7333259	-0.7188577
C	6.3242402	-1.4826628	-1.5547250
C	7.5427381	-1.9625857	-1.0832592
H	8.8330175	-2.1531420	0.6334547
H	5.9993545	-1.6851923	-2.5756211
H	8.2018488	-2.5287373	-1.7434393
N	5.0787422	0.3588600	1.4715994
C	5.5820527	1.5142126	2.0461591
C	3.8023143	0.1104711	1.9967251
C	4.6422580	2.0163271	2.9262034
H	6.5637421	1.8827480	1.7649572
C	3.5343965	1.1288933	2.9044650
H	4.7417955	2.9315353	3.5038206
H	2.6115931	1.2107564	3.4716377
C	2.8751091	-0.9743313	1.5510023
O	1.9325258	-1.2854276	2.3233745
O	3.0719412	-1.4737030	0.3840992
C	4.1562488	-0.3890757	-1.3038823
O	3.6434829	-0.9786770	-2.2369851
H	-3.8191626	-1.9887073	-0.4678138
H	-2.4313767	2.5639874	-0.0024812
N	3.5774836	0.9458890	-0.9609560
C	7.4524158	-0.9453000	2.5865201
C	6.6035341	-1.8976705	3.4474774
H	7.3544827	0.0758197	2.9787272

H	8.5143513	-1.2178506	2.6780846
H	6.9282268	-1.8558840	4.4971853
H	5.5395182	-1.6244120	3.4096245
H	6.7026376	-2.9360826	3.0978388

2c_C

Energy= -3603.6453906233883

N	-12.5858038	3.7599067	-0.2549534
C	-13.7702996	2.9225899	-0.5814390
C	-14.8486148	3.9407037	-0.9504030
C	-14.4948525	5.1509532	-0.0798966
C	-12.9681828	5.1892519	-0.1494862
C	-11.3818273	3.2302731	-0.0156540
C	-10.1770519	3.9976122	0.2130617
C	-8.9942519	3.3536806	0.4568617
N	-8.8878473	1.9889512	0.4669272
C	-10.0063903	1.2293654	0.2284860
C	-11.2201070	1.8072029	0.0058541
C	-7.6078385	1.3860386	0.8512126
O	-6.8442052	2.0528723	1.5242890
C	-10.1016409	5.5052241	0.2497183
O	-10.1645614	6.0932362	1.3529001
C	-7.3883457	-0.0208149	0.4429852
C	-7.4494114	-0.4160359	-0.9102301
C	-7.2222355	-1.7518014	-1.2781278
C	-6.9353995	-2.6681637	-0.2528099

C	-6.8363439	-2.2775978	1.0847187
C	-7.0404868	-0.9449881	1.4373906
N	-7.7149529	0.5833546	-1.8980691
C	-6.9280472	1.7150301	-2.1487379
C	-7.6572143	2.5263176	-3.0120822
C	-8.8947025	1.8889329	-3.2888203
C	-8.9080126	0.6941309	-2.5853623
C	-5.5007691	1.8692320	-1.7036240
O	-4.9281748	0.8226274	-1.2420569
O	-4.9901179	3.0111256	-1.8509034
N	-9.9181942	6.1364625	-0.9224561
C	-9.9552614	7.6183870	-1.0362343
C	-10.0638941	7.8649936	-2.5570601
C	-10.5302929	6.5295049	-3.1531249
C	-9.8648685	5.4921654	-2.2526714
C	-8.7604774	8.3423430	-0.2799904
O	-9.1388256	8.5531865	1.0813921
C	-8.5771723	9.7556765	-0.8492031
C	-7.3519644	10.2507724	-1.2962288
C	-7.2539315	11.5535254	-1.8021227
C	-8.3674129	12.3864764	-1.8632183
C	-9.5893085	11.8968413	-1.3908163
C	-9.6944395	10.6045149	-0.8773477
C	-5.9120049	12.0178138	-2.3071652
F	-5.9080009	13.3248088	-2.6733185
C	-10.8061835	12.7869798	-1.3900619

F	-11.9437454	12.0957597	-1.6865988
C	-7.4984387	7.4687207	-0.3795959
C	-6.9765855	6.8652920	0.7677525
C	-5.8801480	5.9988199	0.6815898
C	-5.2589448	5.7351001	-0.5394794
C	-5.7544059	6.3722798	-1.6775736
C	-6.8668741	7.2135447	-1.6058586
C	-5.3418548	5.3940153	1.9545048
F	-4.5269731	4.3346321	1.7314549
C	-5.0983049	6.1844043	-3.0223978
F	-4.9199746	7.3933793	-3.6490675
F	-11.0095026	13.3625446	-0.1650817
F	-10.7078034	13.8026824	-2.2893297
F	-5.5117148	11.2942464	-3.3976966
F	-4.9358826	11.8602682	-1.3643944
F	-4.6170424	6.3094305	2.6756747
F	-6.3457706	4.9702235	2.7786485
F	-5.8665076	5.4352519	-3.8708338
F	-3.8825579	5.5994351	-2.9459835
O	-3.9519710	1.1391038	1.2569897
H	-6.7591542	-3.7126312	-0.5217022
H	-6.9658038	-0.6189407	2.4755034
H	-6.5958932	-3.0136858	1.8533557
H	-9.6747189	-0.0687834	-2.4894086
H	-9.6940819	2.2496668	-3.9316264
H	-7.2821554	3.4601059	-3.4198127

H	-4.7587831	1.5317592	1.6384667
H	-4.2234270	1.0199395	0.3007988
H	-8.0652100	3.8895973	0.6460509
H	-9.8773675	0.1510676	0.2841716
H	-12.0704226	1.1474940	-0.1424970
H	-6.4561159	9.6323923	-1.2476319
H	-10.6530853	10.2533532	-0.4942153
H	-8.2893123	13.3948968	-2.2670291
H	-7.4344886	7.0712223	1.7347388
H	-7.2182132	7.6890242	-2.5211076
H	-4.4292047	5.0356833	-0.6154827
H	-9.5934555	7.7134248	1.3701789
H	-10.8704817	7.9583802	-0.5255254
H	-9.0935618	8.1530895	-2.9806594
H	-10.7587370	8.6855437	-2.7707618
H	-11.6248107	6.4377877	-3.0907889
H	-10.2394624	6.4118238	-4.2043034
H	-10.3703983	4.5204645	-2.2395765
H	-8.8122026	5.3235870	-2.5304581
H	-14.0534948	2.3364108	0.3077601
H	-13.5298383	2.2312689	-1.3999284
H	-12.6324545	5.7293037	-1.0488439
H	-12.5118819	5.6443835	0.7367071
H	-14.7763878	4.1991411	-2.0175377
H	-15.8546801	3.5482237	-0.7586987
H	-14.9356563	6.0892085	-0.4380364

H	-14.8184660	4.9877893	0.9587358
C	-7.1574090	-2.1867270	-2.7220713
C	-5.7418628	-2.0082389	-3.2995832
H	-7.8715897	-1.6152012	-3.3298359
H	-7.4552832	-3.2444739	-2.7864218
H	-5.7245897	-2.2769095	-4.3657257
H	-5.4062789	-0.9672773	-3.1871450
H	-5.0207942	-2.6490832	-2.7706106

TS_2c_1

Energy= -3527.23696421353

C	2.1524366	1.1908373	-0.4545711
C	4.1358703	2.3127775	-0.8567136
C	1.4212397	2.3722167	-0.4223563
H	1.6526144	0.2239042	-0.3634431
C	3.5045067	3.5345321	-0.8425918
H	5.2094307	2.2351061	-1.0375530
C	2.1033706	3.6265887	-0.5862279
H	4.1014723	4.4282161	-1.0090926
C	-2.7971612	0.5877860	-0.1376660
C	-4.2160663	0.2458439	0.3320119
C	-4.6794167	-1.0648694	0.4531821
C	-5.1146054	1.2985985	0.5593620
C	-6.0061897	-1.3182439	0.8249037
C	-6.4291928	1.0376973	0.9440810
H	-4.7889664	2.3311540	0.4296584

C	-6.8915510	-0.2748615	1.0820420
H	-7.9199824	-0.4750855	1.3796525
C	-1.8540123	-0.6264209	-0.1617357
C	-1.6205506	-1.4124324	0.9763443
C	-1.1508892	-0.9304351	-1.3301151
C	-0.6916117	-2.4556160	0.9458805
H	-2.1748308	-1.2334880	1.8976007
C	-0.2173966	-1.9723572	-1.3494400
H	-1.3351686	-0.3423062	-2.2290234
C	0.0301108	-2.7419328	-0.2135802
H	0.7734526	-3.5357096	-0.2233726
O	-2.9805835	1.1255616	-1.4506457
H	-2.2010445	1.7300115	-1.6150561
C	-2.1601017	1.7085851	0.7830571
C	-2.4164097	1.5991450	2.3015568
C	-1.1125353	2.0723347	2.9596614
H	-2.6368223	0.5644515	2.5937517
H	-3.2823926	2.2032718	2.5966445
C	-0.0387851	1.5803308	1.9923102
H	-1.0847319	3.1704756	3.0200744
H	-0.9790060	1.6695177	3.9716445
H	0.9061184	2.1322933	2.0419946
H	0.1796796	0.5104125	2.1429517
N	-0.6793133	1.7706664	0.6764794
C	-0.0717779	2.2422686	-0.4323835
O	-0.7035629	2.5214338	-1.4804025

C	2.2421554	6.0953948	-0.7345152
C	0.1470932	5.0981917	0.0152273
C	1.1632808	7.1732572	-0.6157433
H	2.7252617	6.0787787	-1.7219390
H	3.0236056	6.2237824	0.0343757
C	0.1901993	6.5767728	0.4087656
H	-0.0722643	4.4424663	0.8694658
H	-0.6023728	4.9124735	-0.7694805
H	1.5840446	8.1374160	-0.3040508
H	0.6599770	7.3131597	-1.5844753
H	0.5925087	6.6859008	1.4274168
H	-0.8070576	7.0340022	0.3797715
N	1.5071061	4.8331958	-0.4962455
C	-7.3684118	2.1972598	1.1592250
C	-6.4458088	-2.7539667	0.9580746
C	-0.4821246	-3.2546555	2.2073700
C	0.4953894	-2.2621274	-2.6459460
F	0.1046791	-2.5036532	3.1904861
F	-1.6751263	-3.6874003	2.7239950
F	0.2891251	-4.3521272	2.0218136
F	-0.3721256	-2.7598741	-3.5869524
F	1.4906374	-3.1716920	-2.5111124
F	1.0380914	-1.1350425	-3.1874393
F	-7.8423507	2.6903998	-0.0265552
F	-8.4537999	1.8584131	1.9043575
F	-6.7547794	3.2381251	1.7920812

F	-5.8115311	-3.3805482	1.9960054
F	-6.1495413	-3.4780289	-0.1622070
F	-7.7808360	-2.8797839	1.1743819
C	8.3603574	-0.1322377	0.4283288
C	7.3085891	0.0780669	1.3335976
C	5.9908212	0.0624843	0.8175056
C	5.7456600	-0.2484104	-0.5334244
C	6.8258707	-0.4597805	-1.3986180
C	8.1327037	-0.3853383	-0.9246113
H	9.3834772	-0.1182347	0.8106558
H	6.6127808	-0.6864243	-2.4438270
H	8.9727995	-0.5450217	-1.6027361
N	4.8796036	0.3321658	1.6742304
C	4.7986157	1.3614131	2.5848708
C	3.7951255	-0.5216644	1.8730739
C	3.6765419	1.1692763	3.3763408
H	5.5394808	2.1529289	2.5930780
C	3.0573673	-0.0284328	2.9458333
H	3.3397755	1.8344342	4.1666305
H	2.1439548	-0.4796041	3.3227099
C	3.3757474	-1.5959878	0.9773493
O	2.7372398	-2.5660441	1.3828864
O	3.5786111	-1.4829809	-0.3617590
C	4.3670254	-0.4210004	-1.1376398
O	4.2049347	-0.5593112	-2.3467838
H	-4.0157192	-1.9037263	0.2440183

H -2.5520039 2.6634916 0.3980300

N 3.4804163 1.1527255 -0.6406589

C 7.6391100 0.1960400 2.8084950

C 7.1159230 -0.9880758 3.6386723

H 7.2628046 1.1350218 3.2364467

H 8.7344006 0.2422488 2.8974827

H 7.4423815 -0.8914851 4.6840641

H 6.0178984 -1.0374453 3.6329292

H 7.5010519 -1.9402926 3.2452633

TS_2c_2

Energy =-3603.6314161622704

N -12.7680402 3.8307234 -0.1204398

C -14.0002921 3.0595459 -0.4164483

C -15.0107901 4.1304923 -0.8279963

C -14.5818734 5.3463151 0.0010406

C -13.0548883 5.2825095 -0.0641142

C -11.6036626 3.2320637 0.1814201

C -10.3586375 3.9198771 0.3993197

C -9.2253628 3.2068202 0.7273326

N -9.2194341 1.8566218 0.8251978

C -10.3766208 1.1689652 0.6190569

C -11.5432706 1.8066550 0.2976151

C -8.0537349 1.0518887 1.4557633

O -8.0987168 0.9890744 2.6807078

C -10.1659522 5.4145896 0.3522188

O	-10.1547019	6.0650831	1.4242755
C	-7.5690804	-0.0935608	0.5950958
C	-7.3775151	-0.0720655	-0.8062178
C	-6.8780816	-1.2032281	-1.4831512
C	-6.5654878	-2.3482260	-0.7330762
C	-6.7458746	-2.3813739	0.6475320
C	-7.2435732	-1.2585219	1.3051484
N	-7.7391231	1.0722808	-1.6057984
C	-6.8611074	1.9853127	-2.2062705
C	-7.5730636	2.6493850	-3.1991573
C	-8.8893682	2.1255262	-3.2270326
C	-8.9570573	1.1403472	-2.2565766
C	-5.4212262	2.1394307	-1.8630417
O	-5.0383357	1.7740499	-0.6722909
O	-4.6507029	2.5988822	-2.7302166
N	-9.9598404	5.9776088	-0.8523821
C	-9.9323447	7.4509064	-1.0452614
C	-10.0915414	7.6262874	-2.5723208
C	-10.6124355	6.2770683	-3.0876223
C	-9.9626226	5.2664202	-2.1472418
C	-8.6800897	8.1581584	-0.3814334
O	-8.9645649	8.4013124	0.9970015
C	-8.5019894	9.5606571	-0.9836604
C	-7.2749154	10.0506887	-1.4322204
C	-7.1726212	11.3481646	-1.9503244
C	-8.2843907	12.1823167	-2.0240850

C	-9.5079211	11.6993811	-1.5495758
C	-9.6162247	10.4129386	-1.0219909
C	-5.8284324	11.8053975	-2.4554734
F	-5.8185426	13.1120240	-2.8235577
C	-10.7237006	12.5906256	-1.5607631
F	-11.8604527	11.8986208	-1.8592928
C	-7.4322107	7.2692408	-0.5411537
C	-6.8529558	6.6802001	0.5870479
C	-5.7226547	5.8648863	0.4665693
C	-5.1276080	5.6319959	-0.7744157
C	-5.6904959	6.2390916	-1.8973763
C	-6.8375057	7.0313089	-1.7886860
C	-5.0861035	5.3334065	1.7267487
F	-4.2212908	4.3137845	1.4943528
C	-5.0425029	6.1198411	-3.2557138
F	-4.8596221	7.3594309	-3.8147407
F	-10.9334840	13.1737736	-0.3403165
F	-10.6196379	13.6007331	-2.4655726
F	-5.4303972	11.0788157	-3.5447713
F	-4.8533889	11.6452622	-1.5119206
F	-4.3756671	6.3150830	2.3697350
F	-6.0112096	4.8861729	2.6247117
F	-5.8169203	5.4222271	-4.1403547
F	-3.8300940	5.5233526	-3.2139886
O	-6.6530664	2.3437796	1.0541075
H	-6.1835904	-3.2302822	-1.2531622

H	-7.3921139	-1.2644002	2.3851972
H	-6.5065803	-3.2835985	1.2131420
H	-9.7587613	0.4585196	-1.9918935
H	-9.7056863	2.4282060	-3.8779963
H	-7.1599516	3.4382075	-3.8199236
H	-6.1830736	2.2807579	1.9060766
H	-5.9166869	2.0203043	0.2506811
H	-8.2636774	3.6841571	0.9034823
H	-10.3251600	0.0864591	0.7318718
H	-12.4300817	1.1964507	0.1488174
H	-6.3798989	9.4319634	-1.3761699
H	-10.5769871	10.0719483	-0.6353645
H	-8.2036368	13.1862042	-2.4384878
H	-7.2801911	6.8786749	1.5696380
H	-7.2297059	7.5017821	-2.6897923
H	-4.2412896	5.0058768	-0.8656528
H	-9.4611386	7.5952729	1.3227835
H	-10.8096214	7.8577591	-0.5169404
H	-9.1306932	7.8685757	-3.0433716
H	-10.7727600	8.4543296	-2.8009119
H	-11.7078190	6.2252133	-3.0012705
H	-10.3471945	6.0962777	-4.1366984
H	-10.4986689	4.3150634	-2.0700098
H	-8.9243112	5.0413253	-2.4378615
H	-14.3277061	2.5251093	0.4910278
H	-13.8073780	2.3224856	-1.2081807

H	-12.6847373	5.7771366	-0.9759333
H	-12.5738482	5.7370030	0.8103294
H	-14.9173944	4.3468474	-1.9030679
H	-16.0415099	3.8117722	-0.6295799
H	-14.9627826	6.2978344	-0.3901180
H	-14.9176392	5.2393559	1.0434194
C	-6.6479700	-1.2242571	-2.9782621
C	-5.1580360	-1.1462384	-3.3523321
H	-7.1933826	-0.4038470	-3.4641211
H	-7.0686091	-2.1618558	-3.3753753
H	-5.0355650	-1.1976569	-4.4441202
H	-4.7100285	-0.2054156	-3.0037491
H	-4.5930930	-1.9784740	-2.9070506

TS_COOH_H_rotation_et

Energy= -896.24338438598

C	-2.1429310	-2.5787058	0.7402318
C	-1.9157801	-0.2330450	0.1972253
C	0.0545486	-1.6665115	0.0994976
C	-0.7946827	-2.7365287	0.4386738
C	-2.7166952	-1.3272862	0.5450688
H	-0.3707756	-3.7409907	0.4452983
H	-3.7976415	-1.1882910	0.5911890
H	-2.7540752	-3.4345265	1.0290312
C	-0.3584111	2.0924766	0.4982176
H	-1.3594449	2.1395528	0.9077744

C	1.6012971	1.1758217	-0.1486825
C	0.4829173	3.1303533	0.1817622
H	0.2546011	4.1862887	0.2930740
C	1.7096647	2.5592261	-0.2354104
H	2.6403453	3.0823515	-0.4397286
C	2.8387216	0.4350550	0.1672487
C	-2.7039569	0.9193265	-0.3533575
O	3.8497986	0.4612588	-0.5163471
O	-3.7139663	1.3753404	0.1570083
O	2.8785769	-0.1826791	1.3841749
H	2.0484780	-0.0313235	1.8806586
O	-2.3167154	1.3875552	-1.5723657
H	-1.5550033	0.8774441	-1.9160918
C	1.4326665	-2.0241603	-0.4320135
H	1.7951102	-1.1983276	-1.0578943
N	0.2728646	0.8681559	0.2632231
H	2.1529948	-2.1423366	0.3899029
C	1.4616228	-3.2944320	-1.2942716
H	0.7025713	-3.2604404	-2.0895873
H	1.3025611	-4.2073388	-0.7044799
H	2.4501799	-3.3820719	-1.7671477
C	-0.4893123	-0.3533172	0.1679650

TS_anhydride_rotation_Et

Energy= -819.85900192840

C	-0.7154421	-0.7766873	-0.0127124

C	-3.3308402	-0.4160689	1.0009687
C	-1.1998171	0.5293454	0.3023016
C	-1.6755064	-1.8293464	-0.1362331
C	-2.9411290	-1.6262905	0.4291302
C	-2.4933116	0.6731394	0.8434619
H	-3.6566785	-2.4478821	0.4005559
H	-2.8298942	1.6767395	1.1005441
H	-4.3082544	-0.3085043	1.4721042
N	0.6805235	-1.0470022	-0.1386787
C	1.7670045	-0.1516559	0.0068751
C	1.2211727	-2.3235275	-0.0768778
H	0.6003340	-3.2041028	-0.0641678
C	2.5942604	-2.2589575	0.0523704
H	3.2594051	-3.1155697	0.1088584
C	2.9388793	-0.9005385	0.1176799
H	3.9288258	-0.4668490	0.2220171
C	1.8086668	1.2844437	0.0174056
C	-0.6314744	1.8329377	-0.1309251
O	0.7040496	2.0392286	-0.3891058
O	-1.3431372	2.8019304	-0.3388050
O	2.8123254	1.9300436	0.2743260
C	-1.4640814	-3.1227555	-0.9187119
C	-2.7231022	-3.6407449	-1.6281458
H	-1.0963757	-3.9266911	-0.2580763
H	-0.6855786	-2.9586184	-1.6770853
H	-2.4448430	-4.4739115	-2.2889288

H	-3.4759007	-4.0229561	-0.9252028
H	-3.1913162	-2.8573605	-2.2416670

Hydrogen

1a

Energy= -817.75230818730

C	-0.7047660	-0.3086446	-0.4272777
C	-3.4425073	-0.4062507	0.1542762
C	-1.4060860	0.8911204	-0.1842638
C	-1.3718598	-1.5345281	-0.3792961
C	-2.7395893	-1.5862759	-0.0983855
C	-2.7752172	0.8189399	0.1199525
H	-3.2486004	-2.5511204	-0.0697635
H	-3.3220229	1.7420154	0.3114467
H	-4.5097296	-0.4369257	0.3783285
N	0.6888436	-0.3342943	-0.7605474
C	1.1399000	-0.7485346	-1.9879567
H	0.4324878	-1.0535860	-2.7532382
C	1.7879223	0.0219817	0.0224636
C	2.5269120	-0.6636223	-2.0114177
H	3.1624365	-0.9244629	-2.8530049
C	2.9345099	-0.1831384	-0.7500829
H	3.9507891	-0.0090140	-0.4086609
C	1.6796444	0.4686372	1.4000940

C	-0.7464768	2.2240698	-0.2950593
O	0.6391063	0.5836691	2.0468996
O	0.2170113	2.4793574	-1.0038539
O	2.9031789	0.7616828	1.9320580
H	2.7509937	1.0433859	2.8586691
O	-1.3617009	3.1614397	0.4692272
H	-0.9160633	4.0190629	0.2996851
H	-0.8050055	-2.4504631	-0.5526527

1a-A

Energy= -1585.1884065763043

N	-2.5373014	-1.1587515	2.6965416
C	-2.1956413	-0.0579150	2.3005634
N	-1.7442363	1.0687071	2.0529715
O	0.9449214	-0.8370782	1.4312184
C	1.5306444	0.2301263	1.5943965
O	0.9293543	1.3608884	1.9847907
C	3.0080202	0.3970890	1.4132553
C	3.7180885	-0.3097963	0.4226613
C	5.1053499	-0.1759379	0.3195497
C	5.8034682	0.6500008	1.2045839
C	5.1086489	1.3640728	2.1838525
C	3.7203044	1.2446523	2.2747167
N	3.0717953	-1.2014706	-0.4950325
C	3.3460328	-2.5455520	-0.5236931
C	2.5583410	-3.1478943	-1.4968381

C	1.7748217	-2.1322309	-2.0816846
C	2.0951395	-0.9253218	-1.4541382
C	1.5818717	0.4076714	-1.7207316
O	0.6072061	0.3896684	-2.6789606
O	1.9428437	1.4496939	-1.1786862
C	-4.0451307	2.0404369	1.8015153
H	6.8874634	0.7412353	1.1174734
H	3.1716412	1.8016926	3.0348474
H	5.6446893	2.0170502	2.8744562
H	4.0659867	-2.9685925	0.1705374
H	2.5572173	-4.2053155	-1.7461727
H	1.0560998	-2.2404146	-2.8884236
H	-4.5831485	2.9529783	1.5105483
H	-4.4150292	1.2118290	1.1786647
H	-4.2789463	1.8189373	2.8525759
H	0.2388226	1.3002082	-2.7224036
C	-2.6937123	-2.4168893	1.9239055
C	-2.5421857	2.2459240	1.6160679
H	-2.2109006	3.0721841	2.2635141
C	-2.8997653	-2.1623015	0.4401604
C	-4.2009001	-2.1900614	-0.0849918
C	-1.8261437	-1.8678467	-0.4165289
C	-4.4311918	-1.9384008	-1.4414995
H	-5.0419486	-2.4110691	0.5775966
C	-2.0560225	-1.6226828	-1.7732082
H	-0.8081732	-1.8063807	-0.0257381

C	-3.3553703	-1.6594604	-2.2908463
H	-5.4499909	-1.9625173	-1.8340467
H	-1.2154981	-1.3718569	-2.4213753
H	-3.5297459	-1.4618804	-3.3507976
C	-2.1608799	2.5995905	0.1848727
C	-1.4669366	3.7863440	-0.0879554
C	-2.4967151	1.7496080	-0.8776558
C	-1.1192119	4.1243614	-1.3998197
H	-1.1940452	4.4512315	0.7357198
C	-2.1594519	2.0869847	-2.1905227
H	-3.0117489	0.8065244	-0.6847620
C	-1.4691893	3.2772888	-2.4571800
H	-0.5775180	5.0516681	-1.5969278
H	-2.4332146	1.4163087	-3.0078252
H	-1.2102200	3.5445868	-3.4841797
H	-0.0845074	1.1998555	2.0372269
H	-3.6099375	-2.8790534	2.3211586
C	-1.5109264	-3.3354835	2.2458014
H	-1.6450897	-4.2996067	1.7356141
H	-0.5647106	-2.8851052	1.9177413
H	-1.4565006	-3.5158098	3.3282620
H	5.6313366	-0.7146680	-0.4701455

1a-B

Energy= -1585.19769018617

C	3.3129523	1.0465141	-0.0852172

C	3.1806824	3.5187486	-1.4072206
C	2.0959382	1.4703819	-0.6650914
C	4.4410391	1.8693069	-0.1575377
C	4.3722120	3.1061815	-0.8007105
C	2.0570815	2.6973325	-1.3491720
H	5.2603208	3.7385379	-0.8463953
H	1.1163875	3.0099823	-1.8047079
H	3.1277651	4.4782650	-1.9235132
N	3.4322780	-0.2132252	0.5703944
C	3.7643816	-0.3563599	1.9042292
H	4.0873198	0.5035521	2.4832274
C	2.9578909	-1.4320922	0.0926275
C	3.5313641	-1.6699477	2.2737204
H	3.7160349	-2.0933512	3.2573893
C	3.0093580	-2.3446300	1.1384030
H	2.7403923	-3.3948151	1.0633689
C	2.6264365	-1.6626398	-1.3310961
C	0.8188119	0.7075314	-0.6113879
O	3.3308021	-1.2355924	-2.2518875
O	0.6366573	0.1150098	0.6324150
O	1.5314699	-2.3861944	-1.5552610
O	0.0026995	0.6444589	-1.5032001
C	-0.3981427	-0.8017411	0.7937532
N	-0.3297668	-1.9299512	0.1682600
N	-1.3116390	-0.3926965	1.6972846
C	-1.4274589	0.9842297	2.2182753

H	-2.1150682	-1.0024667	1.8359827
C	-1.4178322	-2.9097480	0.3457789
H	0.7923282	-2.2441619	-0.7654468
C	-2.7582947	-2.3624526	-0.1382241
C	-3.9102161	-2.5043661	0.6489503
C	-2.8538176	-1.6995453	-1.3718406
C	-5.1382470	-1.9896151	0.2150505
H	-3.8451931	-3.0159356	1.6137681
C	-4.0785890	-1.1903297	-1.8083551
H	-1.9551664	-1.5530134	-1.9745394
C	-5.2246587	-1.3312740	-1.0152718
H	-6.0250509	-2.0986406	0.8428118
H	-4.1374603	-0.6677770	-2.7654388
H	-6.1790429	-0.9221803	-1.3527205
C	-1.7681857	1.9856349	1.1222675
C	-1.0275109	3.1683561	0.9996485
C	-2.8161599	1.7405133	0.2225609
C	-1.3262751	4.0945418	-0.0062158
H	-0.1977430	3.3573815	1.6859520
C	-3.1146443	2.6613559	-0.7837705
H	-3.3845511	0.8098629	0.2847332
C	-2.3708334	3.8424294	-0.9010630
H	-0.7335661	5.0071138	-0.0973854
H	-3.9254071	2.4531239	-1.4853121
H	-2.5997534	4.5586720	-1.6927705
H	-1.5147978	-3.1439321	1.4232377

C	-1.0441540	-4.2040606	-0.3836765
H	-0.0919926	-4.5988600	-0.0016363
H	-1.8248773	-4.9612576	-0.2302393
H	-0.9422199	-4.0234949	-1.4637871
C	-2.4711040	0.9767513	3.3377544
H	-2.5577485	1.9822570	3.7688610
H	-3.4598620	0.6891410	2.9476468
H	-2.1840752	0.2752893	4.1340157
H	-0.4507767	1.2580737	2.6440458
H	5.3796976	1.5186567	0.2737914

1a-C

Energy= -1585.1998463854368

C	3.0452969	0.2826332	0.0293218
C	3.4821553	2.6765804	-1.3795730
C	1.9748270	0.9132412	-0.6463713
C	4.3128984	0.8734640	0.0115246
C	4.5277345	2.0697964	-0.6742008
C	2.2186890	2.0925107	-1.3731061
H	5.5232012	2.5170539	-0.6758816
H	1.3872050	2.5618620	-1.9006278
H	3.6509571	3.6034421	-1.9291530
N	2.8781012	-0.9475112	0.7206830
C	3.1264662	-1.1048066	2.0753895
H	3.5744788	-0.2985685	2.6482857
C	2.2159223	-2.0767019	0.2421631

C	2.6427769	-2.3411025	2.4604888
H	2.7013000	-2.7594241	3.4621621
C	2.0602402	-2.9473798	1.3107460
H	1.6063709	-3.9327535	1.2459118
C	1.8706679	-2.2744111	-1.2041828
C	0.5730510	0.4401056	-0.6415730
O	2.7104241	-1.9503506	-2.0782946
O	0.2198411	-0.0913197	0.6235011
O	0.7072794	-2.7815144	-1.4321858
O	-0.2300760	0.5446731	-1.5356144
C	-0.9808267	-0.7241935	0.7633660
N	-1.1904163	-1.8486773	0.1234556
N	-1.8182578	-0.1490549	1.6234495
C	-1.6362759	1.2105368	2.1862157
H	-2.7088017	-0.6128330	1.7979019
C	-2.4824017	-2.5645347	0.1783715
C	-3.6189098	-1.6839332	-0.3277642
C	-4.7872527	-1.5364552	0.4324878
C	-3.5081368	-1.0088951	-1.5530795
C	-5.8327374	-0.7228938	-0.0217784
H	-4.8790826	-2.0577467	1.3896738
C	-4.5515587	-0.2006513	-2.0084979
H	-2.5900610	-1.0929787	-2.1379709
C	-5.7163445	-0.0540023	-1.2440214
H	-6.7336756	-0.6090724	0.5840393
H	-4.4519559	0.3275839	-2.9587730

H	-6.5268968	0.5859478	-1.5981101
C	-1.7046530	2.2714323	1.0985730
C	-0.6862534	3.2261198	0.9880933
C	-2.7771312	2.3058652	0.1959055
C	-0.7375947	4.2050155	-0.0101947
H	0.1616408	3.1938015	1.6774198
C	-2.8279755	3.2789865	-0.8030878
H	-3.5663506	1.5520728	0.2525754
C	-1.8083567	4.2327183	-0.9084417
H	0.0671339	4.9384518	-0.0927936
H	-3.6628045	3.2888834	-1.5072189
H	-1.8458412	4.9900500	-1.6941166
H	-2.6836469	-2.8240551	1.2329282
C	-2.3545990	-3.8638735	-0.6197959
H	-1.5455169	-4.4892768	-0.2177600
H	-3.2975142	-4.4211586	-0.5562893
H	-2.1431288	-3.6511199	-1.6770968
C	-2.6924630	1.4116329	3.2729274
H	-2.5588625	2.3989666	3.7325074
H	-3.7066472	1.3700497	2.8469833
H	-2.5995736	0.6452266	4.0555403
H	-0.6375126	1.2414846	2.6449855
H	5.1372460	0.3688134	0.5175447
H	-0.3783380	-2.2675341	-0.4830668

TS_1a_1

Energy= -1585.17050443746

C	14.1898907	-16.8570212	-1.8963605
C	13.9342340	-16.4281293	0.8458292
C	13.0365955	-16.2245473	-1.4046842
C	15.2001985	-17.2826095	-1.0319528
C	15.0800886	-17.0533715	0.3400963
C	12.9141185	-16.0298669	-0.0208782
H	15.8745031	-17.3765260	1.0149813
H	12.0141836	-15.5507466	0.3697643
H	13.8328037	-16.2563466	1.9190431
N	14.2843895	-17.1779520	-3.2956273
C	13.6005151	-18.2224317	-3.8606631
H	12.9611107	-18.8508278	-3.2474125
C	15.0316055	-16.5435674	-4.2855362
C	13.9072543	-18.2807605	-5.2168718
H	13.5242067	-19.0142755	-5.9209370
C	14.8017729	-17.2256452	-5.4857323
H	15.2426000	-16.9650973	-6.4430963
C	15.8346555	-15.3580133	-4.0388142
C	11.9645953	-15.7172646	-2.3386301
O	15.9980125	-14.7975261	-2.9592225
O	10.7636654	-16.0862642	-2.1484385
O	16.4157945	-14.9031014	-5.1936214
O	12.3561980	-14.9272586	-3.2593511
H	10.1022980	-15.6574414	-3.5617008
H	16.9535140	-14.1203148	-4.9518084

H	16.0734994	-17.7929419	-1.4412670
C	11.0513177	-14.5627849	-4.9273824
N	10.0763036	-15.3304386	-4.5844528
N	11.6223569	-13.7583867	-5.6234559
C	13.0310825	-13.6586995	-6.0243042
H	13.5527338	-14.5453882	-5.6345440
C	9.1553847	-15.9694344	-5.5801273
H	8.3148496	-15.2730730	-5.7254373
C	9.8370804	-16.1934920	-6.9158713
C	9.2290003	-15.7458512	-8.0953698
C	11.0663021	-16.8661001	-6.9943816
C	9.8378576	-15.9669641	-9.3352623
H	8.2771686	-15.2110592	-8.0421598
C	11.6825358	-17.0722223	-8.2295563
H	11.5650352	-17.2057623	-6.0830412
C	11.0678150	-16.6266875	-9.4047820
H	9.3580738	-15.6045585	-10.2466969
H	12.6515887	-17.5734436	-8.2742491
H	11.5559981	-16.7768123	-10.3693873
C	13.1497581	-13.6537289	-7.5386369
C	14.3268290	-14.1486154	-8.1197199
C	12.1428483	-13.1407419	-8.3649498
C	14.4946085	-14.1282465	-9.5067216
H	15.1133191	-14.5547707	-7.4780414
C	12.3088457	-13.1236096	-9.7529656
H	11.2144223	-12.7762542	-7.9212880

C	13.4841791	-13.6158142	-10.3285843
H	15.4133575	-14.5217480	-9.9472745
H	11.5099330	-12.7347097	-10.3878415
H	13.6098972	-13.6081361	-11.4133512
C	8.6348502	-17.2662047	-4.9569312
H	8.1454207	-17.0694365	-3.9922202
H	7.8996017	-17.7225207	-5.6326165
H	9.4553976	-17.9812219	-4.8007942
C	13.6417401	-12.4038581	-5.3891299
H	13.1147471	-11.5013950	-5.7300341
H	13.5843919	-12.4688794	-4.2941485
H	14.6973590	-12.3253987	-5.6834532

TS_1a_2

Energy= -1585.1937731871237

C	2.5686337	-0.7460639	1.0698298
C	0.2393274	-1.2519709	2.5581110
C	1.3206171	-0.8715400	0.4118043
C	2.6269785	-0.8690880	2.4593997
C	1.4716647	-1.1326929	3.2014459
C	0.1673012	-1.1063382	1.1729546
H	1.5403026	-1.2312498	4.2859955
H	-0.7951068	-1.2161411	0.6740051
H	-0.6691324	-1.4529758	3.1270878
N	3.7906101	-0.5063084	0.3708327
C	4.8818499	-1.3428763	0.4895009

H	4.8444463	-2.2007441	1.1545222
C	4.0786623	0.4696396	-0.5928136
C	5.8717345	-0.9096087	-0.3786273
H	6.8491386	-1.3684320	-0.5018660
C	5.3641225	0.2212438	-1.0626966
H	5.8756099	0.8305502	-1.8031036
C	3.1565425	1.5475411	-0.9769851
C	1.2424989	-0.9142569	-1.0681884
O	3.2514446	2.1299491	-2.0669691
O	0.1105711	-0.3783423	-1.7096111
O	2.2328302	1.8129058	-0.0606159
O	2.0312567	-1.4800433	-1.7902913
H	3.5862710	-0.7352671	2.9613191
C	-0.6819401	0.6766250	-1.3060295
N	-0.1688744	1.7451482	-0.7777765
N	-1.9700155	0.4902401	-1.6601419
C	-2.5806496	-0.7893969	-2.0715225
H	-2.6165935	1.2299943	-1.3939477
C	-1.0586977	2.8700169	-0.4187839
H	1.1846621	1.8770030	-0.4646876
C	-2.0571212	2.4827592	0.6677058
C	-3.4254974	2.7398686	0.5012037
C	-1.6210432	1.8572393	1.8457444
C	-4.3464827	2.3685365	1.4889587
H	-3.7763455	3.2316713	-0.4112109
C	-2.5372185	1.4862686	2.8318561

H	-0.5589857	1.6420343	1.9804943
C	-3.9035841	1.7386083	2.6559327
H	-5.4100277	2.5661404	1.3408766
H	-2.1859226	0.9912710	3.7396262
H	-4.6200743	1.4404135	3.4238137
C	-2.7316811	-1.7398899	-0.8913113
C	-3.3614130	-1.3199834	0.2917580
C	-2.2239784	-3.0434080	-0.9670545
C	-3.4713067	-2.1869188	1.3816014
H	-3.7434535	-0.2997561	0.3790566
C	-2.3365391	-3.9149216	0.1226032
H	-1.7205523	-3.3731999	-1.8797465
C	-2.9583893	-3.4876217	1.3000780
H	-3.9494132	-1.8422975	2.3007960
H	-1.9265655	-4.9245757	0.0542917
H	-3.0362257	-4.1615409	2.1553592
H	-1.6299455	3.1662422	-1.3189344
C	-0.2031669	4.0682496	0.0037152
H	0.3742651	3.8361919	0.9100433
H	0.4973108	4.3425229	-0.7974571
H	-0.8519427	4.9285965	0.2158492
C	-3.9203844	-0.4799427	-2.7424441
H	-3.7773085	0.1777156	-3.6112023
H	-4.3888538	-1.4136657	-3.0791387
H	-4.6099189	0.0081405	-2.0359073
H	-1.9086634	-1.2509115	-2.8078950

TS_1a_fuel_3

Energy= -1585.19327085733

C	3.1438652	-1.1598434	0.3129909
C	3.6194301	-2.5770759	2.6948923
C	2.3459347	-0.9129121	1.4490954
C	4.1774088	-2.1044035	0.3902652
C	4.4099089	-2.8152723	1.5677152
C	2.6026567	-1.6256455	2.6299027
H	5.2234579	-3.5416843	1.6046692
H	1.9786926	-1.4204497	3.5006933
H	3.7974844	-3.1230367	3.6224888
N	2.9485235	-0.4986494	-0.9332052
C	3.0313793	-1.1680509	-2.1414696
H	3.1353285	-2.2481970	-2.1647988
C	2.8176072	0.8728880	-1.1899055
C	2.9377455	-0.2458801	-3.1689191
H	2.9357067	-0.4801792	-4.2298937
C	2.8172991	1.0339617	-2.5719406
H	2.7203197	1.9914822	-3.0758667
C	2.7037871	1.9537741	-0.1819080
C	1.2488252	0.0953681	1.5130021
O	3.0162100	3.1166476	-0.5206763
O	0.4334271	-0.0231428	0.2609646
O	2.2708342	1.6734000	1.0163547
O	0.6863712	0.4415169	2.5437782

H	4.8188872	-2.2610730	-0.4777451
C	-0.5404615	0.8356189	-0.0116647
N	-0.9916635	1.7175873	0.8763459
N	-1.0349927	0.7560905	-1.2501341
C	-0.6653643	-0.2960272	-2.2182355
H	-1.8889436	1.2812947	-1.4401281
C	-2.0911400	2.6674788	0.6227693
H	-0.5657449	1.6412634	1.8081337
C	-3.3861181	1.9375380	0.2796502
C	-4.1283181	2.3197865	-0.8465569
C	-3.8408175	0.8722291	1.0713123
C	-5.3082701	1.6443612	-1.1808009
H	-3.7786258	3.1475197	-1.4702046
C	-5.0185325	0.2003333	0.7398550
H	-3.2611320	0.5540787	1.9412682
C	-5.7538107	0.5825349	-0.3888557
H	-5.8734686	1.9455051	-2.0649323
H	-5.3591035	-0.6326640	1.3580279
H	-6.6687721	0.0485307	-0.6524870
C	-1.2950142	-1.6326615	-1.8516397
C	-2.6679707	-1.7350682	-1.5811246
C	-0.5011910	-2.7852079	-1.7931134
C	-3.2361283	-2.9697371	-1.2575371
H	-3.3028069	-0.8459299	-1.6082025
C	-1.0683748	-4.0243224	-1.4760946
H	0.5708695	-2.7077369	-1.9892733

C	-2.4378063	-4.1192213	-1.2068667
H	-4.3048782	-3.0349176	-1.0426155
H	-0.4373988	-4.9143405	-1.4303134
H	-2.8816143	-5.0840329	-0.9531148
H	-1.8033933	3.2919601	-0.2399700
C	-2.2351377	3.5684080	1.8499072
H	-3.0249301	4.3079598	1.6688332
H	-2.5120292	2.9773703	2.7351675
H	-1.2955566	4.1019121	2.0523417
C	-1.0640088	0.1825453	-3.6140173
H	-0.7760859	-0.5727178	-4.3562514
H	-2.1527300	0.3305257	-3.6862504
H	-0.5578856	1.1274166	-3.8575372
H	0.4264572	-0.3977298	-2.1772159

3a

Energy= -741.33697064310

C	-2.8992126	-1.6835889	-0.5741498
C	-1.5326106	-1.8562073	-0.3674346
C	-0.7225641	-0.7699143	-0.0056028
C	-1.2978239	0.5101874	0.1267743
C	-2.6841824	0.6604953	-0.0688637
C	-3.4820561	-0.4209066	-0.4252756
H	-3.5076472	-2.5411034	-0.8650026
H	-1.0847351	-2.8398902	-0.5043973
H	-3.1196406	1.6527337	0.0532688

H	-4.5512549	-0.2803285	-0.5881248
N	0.6514094	-1.0046064	0.2611097
C	1.1565606	-2.2164859	0.6793518
C	1.7295134	-0.1961583	-0.1261108
C	2.5384928	-2.1894209	0.5814420
H	0.4944814	-3.0022122	1.0274130
C	2.9005807	-0.9307654	0.0582921
H	3.2084687	-2.9908023	0.8790871
H	3.9016911	-0.5515567	-0.1256350
C	1.6874854	1.1873914	-0.5346042
O	2.5706728	1.7421160	-1.1632619
O	0.6616592	2.0283546	-0.0656510
C	-0.5728043	1.7442227	0.5065319
O	-1.0558097	2.6206466	1.1940474

3a-A

Energy= -3448.6999610798175

C	-0.5244149	-0.7707463	6.3230506
C	-0.3666415	0.7532565	8.6844125
C	-0.9709855	0.5677650	6.3237014
C	-0.0327475	-1.3349413	7.5110900
C	0.0506997	-0.5814491	8.6791721
C	-0.8680796	1.3132233	7.5143851
H	0.4320248	-1.0461751	9.5897769
H	-1.2026266	2.3506827	7.4982503
H	-0.3074020	1.3513252	9.5945370

N	-0.5054732	-1.5638631	5.1511901
C	-1.4462518	-1.5796605	4.1170226
C	0.3949611	-2.5822175	4.9269046
H	1.2290604	-2.7442219	5.6006834
C	0.0452393	-3.2467618	3.7622962
H	0.5934703	-4.0715726	3.3170107
C	-1.1216334	-2.6337134	3.2629852
H	-1.6651060	-2.8770282	2.3556007
C	-2.5164100	-0.6394951	3.8678810
C	-1.5317869	1.3144842	5.1684196
O	-2.4326783	0.6786206	4.3046785
O	-1.4729419	2.5211009	5.0617111
O	-3.4835418	-0.9034627	3.1683652
N	0.7516176	0.7551761	3.7567577
C	0.9251824	0.2610523	2.5250293
C	1.8625193	1.2137835	4.3667464
C	2.1450583	0.1953255	1.8462293
H	0.0267901	-0.1051194	2.0196236
C	3.1301290	1.1846935	3.8077179
H	1.7247142	1.6226313	5.3729324
C	3.3282480	0.6467900	2.5047838
H	3.9701885	1.5692141	4.3837588
C	0.6146304	-0.6157563	-2.3378148
C	0.2961530	-0.4030114	-3.8225714
C	1.0673903	-1.0734985	-4.7768425
C	-0.7091081	0.4704614	-4.2537416

C	0.8414115	-0.8592513	-6.1405558
H	1.8408954	-1.7669251	-4.4467948
C	-0.9278291	0.6747790	-5.6166958
H	-1.3325632	0.9924083	-3.5285148
C	-0.1560730	0.0121609	-6.5754686
H	-0.3294035	0.1732350	-7.6395385
C	-0.6208789	-0.3359490	-1.4661592
C	-1.5371764	-1.3789062	-1.2734930
C	-0.8920427	0.8999859	-0.8736030
C	-2.6828361	-1.1859676	-0.5003773
H	-1.3377875	-2.3463495	-1.7349224
C	-2.0257421	1.0746359	-0.0699888
H	-0.2258317	1.7474665	-1.0200356
C	-2.9327358	0.0370671	0.1279343
H	-3.8073976	0.1720394	0.7610079
O	0.9832202	-1.9884634	-2.2121632
H	1.3585201	-2.0423261	-1.2831485
C	1.8740110	0.2808675	-1.9838847
C	1.9545328	1.6609607	-2.6652589
H	2.7289825	-0.3365559	-2.3068361
C	2.8335718	2.4891225	-1.7252960
H	0.9573667	2.1161446	-2.7569948
H	2.3646039	1.5779847	-3.6787332
C	2.3881434	2.0330803	-0.3364632
H	3.8951476	2.2432859	-1.8796796
H	2.7076737	3.5700281	-1.8635426

H	3.1721943	2.1447237	0.4208187
H	1.4996038	2.5834748	0.0140196
N	2.0557618	0.6023780	-0.5437024
C	2.0392541	-0.3223767	0.4426203
O	1.8217384	-1.5407664	0.2339229
C	4.9540406	-0.2001760	0.7720979
C	5.7587662	1.1333769	2.6473702
C	6.4664505	-0.3832035	0.9334863
H	4.7280507	0.3391225	-0.1640301
H	4.4103814	-1.1548008	0.7608528
C	6.8938021	0.9056194	1.6471465
H	5.9349867	0.5743484	3.5841674
H	5.6177832	2.1942290	2.9017637
H	6.6733122	-1.2572780	1.5695424
H	6.9656067	-0.5332501	-0.0324917
H	7.8684606	0.8238896	2.1448611
H	6.9416879	1.7412594	0.9318582
N	4.5704911	0.6036417	1.9505611
C	-2.0189549	1.6014863	-6.0895741
C	1.6758001	-1.6237958	-7.1368363
C	-3.6429320	-2.3359737	-0.3306646
C	-2.2372767	2.4216287	0.5708493
F	-4.8426182	-1.9478691	0.1711347
F	-3.8877723	-2.9683397	-1.5161224
F	-3.1483183	-3.2941164	0.5161796
F	-1.1594493	2.7915127	1.3250132

F	-3.3261677	2.4600157	1.3756206
F	-2.3983191	3.4039055	-0.3718001
F	-1.5426864	2.5317908	-6.9696495
F	-3.0100397	0.9251032	-6.7453449
F	-2.6109674	2.2805303	-5.0719711
F	1.5549958	-1.1427405	-8.4022640
F	1.3235457	-2.9456437	-7.1786053
F	3.0030912	-1.5904462	-6.8194332
H	0.2733992	-2.3805489	7.5224495

3a_B

Energy= -3448.6989712374398

N	2.1235148	4.4781437	-0.0381957
C	2.9734788	5.6588074	-0.3305298
C	1.9665173	6.7735738	-0.6173688
C	0.7703626	6.3930631	0.2630758
C	0.7044974	4.8742031	0.1018723
C	2.6427161	3.2710430	0.2241018
C	1.8544918	2.0847260	0.4664737
C	2.4746166	0.9258738	0.8727508
N	3.8234291	0.8358687	1.0089046
C	4.6038693	1.9161549	0.7187592
C	4.0590756	3.1042543	0.3198559
C	4.3961513	-0.3895483	1.6816785
O	3.8586402	-0.7209272	2.7255206
C	0.4050130	1.9973800	0.0847667

O	0.0962241	2.1889190	-1.1134402
C	5.7993311	-0.7280321	1.3029223
C	6.3303649	-0.6532950	-0.0046904
C	7.6755283	-0.9750807	-0.2255229
C	8.4972408	-1.3701684	0.8312616
C	7.9740268	-1.4672866	2.1236481
C	6.6355325	-1.1511536	2.3493513
N	5.5518764	-0.2411767	-1.1240121
C	4.3713629	-0.8191052	-1.6066378
C	4.0853450	-0.2135846	-2.8216112
C	5.0946800	0.7523582	-3.0858135
C	5.9986103	0.6984434	-2.0424432
C	3.6045010	-1.8971748	-0.9169514
O	3.5358353	-1.8578010	0.3707922
O	3.0497732	-2.7683917	-1.6291641
N	-0.4892360	1.6791454	1.0425973
C	-1.9530702	1.6618175	0.7749438
C	-2.5666468	1.4559456	2.1716642
C	-1.5273232	2.0564429	3.1235537
C	-0.1999907	1.6324359	2.4976814
C	-2.4203926	0.6860167	-0.3875466
O	-2.3913921	1.4067969	-1.6192963
C	-3.8964262	0.3459824	-0.1507788
C	-4.8561792	1.3236331	-0.4509422
C	-6.2067730	1.0870542	-0.1937923
C	-6.6342138	-0.1273207	0.3522213

C	-5.6777052	-1.0999959	0.6360761
C	-4.3189315	-0.8696113	0.3889130
C	-7.2244404	2.1377084	-0.5632266
F	-7.6509617	1.9954368	-1.8570680
C	-6.0794371	-2.4129025	1.2577432
F	-5.4702231	-3.4700569	0.6448160
C	-1.5362220	-0.5659465	-0.4856829
C	-1.1759783	-1.0438950	-1.7501603
C	-0.3398795	-2.1552919	-1.8794849
C	0.1664463	-2.8140483	-0.7576621
C	-0.2081877	-2.3481591	0.5015567
C	-1.0541751	-1.2425165	0.6400031
C	0.0405162	-2.5999866	-3.2687758
F	0.9233057	-1.7388745	-3.8586387
C	0.2750826	-3.0250440	1.7571584
F	0.8262346	-2.1286395	2.6317207
F	-5.7164972	-2.4730843	2.5771960
F	-7.4180074	-2.6346680	1.2060929
F	-6.7226545	3.3993783	-0.4562551
F	-8.3358484	2.0806441	0.2207419
F	0.5999310	-3.8348241	-3.2993251
F	-1.0541313	-2.6405776	-4.0947555
F	1.1948076	-3.9887200	1.5296271
F	-0.7648003	-3.6187863	2.4327076
H	9.5421170	-1.6184628	0.6372214
H	6.2125617	-1.2139165	3.3528789

H	8.6060687	-1.7863975	2.9536852
H	6.9109701	1.2565653	-1.8556755
H	5.1523496	1.4253214	-3.9374931
H	3.2167778	-0.4505333	-3.4294963
H	1.9352084	0.0000281	1.0698675
H	5.6752309	1.7928667	0.8602783
H	4.7355608	3.9319345	0.1245309
H	-4.5357492	2.2684074	-0.8887052
H	-3.5947040	-1.6541082	0.6109498
H	-7.6900078	-0.3097544	0.5494506
H	-1.5475929	-0.5257698	-2.6340513
H	-1.3214328	-0.9153050	1.6439606
H	0.8670180	-3.6381987	-0.8655798
H	-1.4797774	1.8126763	-1.6427596
H	-2.2315513	2.6619223	0.4030539
H	-2.7162720	0.3887693	2.3916338
H	-3.5478021	1.9394385	2.2445727
H	-1.6049919	3.1537455	3.1347064
H	-1.6310357	1.6914857	4.1528348
H	0.6369291	2.2969731	2.7415094
H	0.0750484	0.6083623	2.7960519
H	3.6400786	5.4471360	-1.1779052
H	3.5892842	5.8926073	0.5536475
H	0.2560421	4.3710685	0.9697772
H	0.1432466	4.5890309	-0.8003105
H	2.3807154	7.7620235	-0.3829025

H	1.6794974	6.7617740	-1.6793219
H	0.9659415	6.6557524	1.3135303
H	-0.1672766	6.8713550	-0.0463776
H	8.0670534	-0.9341820	-1.2429983

3a-C

Energy= -3525.09936598633

N	1.7971114	4.6475003	-0.3327487
C	2.6602031	5.8205054	-0.6270047
C	1.6657700	6.9356867	-0.9504892
C	0.4498137	6.5771394	-0.0886613
C	0.3774970	5.0556708	-0.2198512
C	2.2963623	3.4310188	-0.1017081
C	1.4902163	2.2515305	0.1260832
C	2.0934339	1.0655824	0.4487963
N	3.4543115	0.9325309	0.5330931
C	4.2501872	2.0209982	0.2675823
C	3.7128701	3.2358209	-0.0363861
C	4.0068355	-0.3424381	1.0039158
O	3.2899082	-1.0605721	1.6756989
C	0.0156426	2.1834126	-0.1781195
O	-0.3499711	2.2694950	-1.3700461
C	5.4309901	-0.5999289	0.6849264
C	5.9160873	-0.5597192	-0.6395703
C	7.2736805	-0.7526065	-0.8908168
C	8.1425898	-1.0235825	0.1697345

C	7.6561791	-1.1290539	1.4779536
C	6.3001622	-0.9291418	1.7352883
N	5.0019581	-0.3349920	-1.7075236
C	3.8268110	-1.0665321	-1.9304865
C	3.0822639	-0.3516398	-2.8632618
C	3.8067382	0.8174840	-3.2137859
C	4.9866628	0.8073035	-2.4856903
C	3.6014177	-2.4648184	-1.4231614
O	4.6202085	-3.0642020	-0.9291101
O	2.4407062	-2.9302227	-1.5577313
N	-0.8209274	1.9705425	0.8555044
C	-2.2982800	1.9191348	0.6802690
C	-2.8252094	1.8800731	2.1264076
C	-1.7299936	2.5756449	2.9413921
C	-0.4417100	2.0793237	2.2867795
C	-2.8134695	0.8033925	-0.3226930
O	-2.8500595	1.3593645	-1.6366695
C	-4.2717929	0.4738984	0.0270455
C	-5.2830165	1.3287797	-0.4377655
C	-6.6149054	1.0856175	-0.1034897
C	-6.9725820	-0.0058762	0.6955138
C	-5.9659355	-0.8525169	1.1507955
C	-4.6259423	-0.6217860	0.8158154
C	-7.7117339	1.9651478	-0.6483583
F	-8.3557602	1.3684590	-1.6994848
C	-6.2890230	-2.0336045	2.0299541

F	-5.7870716	-3.1972529	1.5192012
C	-1.9326103	-0.4561225	-0.3076751
C	-1.6894527	-1.1222813	-1.5134508
C	-0.8905957	-2.2674295	-1.5454086
C	-0.3027333	-2.7722262	-0.3849908
C	-0.5606422	-2.1180652	0.8195197
C	-1.3708664	-0.9781297	0.8627627
C	-0.6717255	-2.9411852	-2.8763609
F	0.1049246	-2.1912068	-3.7128854
C	-0.0215954	-2.6579980	2.1185485
F	0.4154296	-1.6566330	2.9419826
F	-5.7384932	-1.8939689	3.2752968
F	-7.6230083	-2.2147525	2.2027686
F	-7.2517069	3.1618546	-1.0978247
F	-8.6678900	2.2265876	0.2892449
F	-0.0944279	-4.1609148	-2.7677735
F	-1.8632718	-3.1253200	-3.5346731
F	1.0062968	-3.5218891	1.9504753
F	-0.9952419	-3.3265515	2.8191838
O	4.1986833	-3.9783517	1.5774686
H	9.2034759	-1.1810015	-0.0322727
H	5.9104979	-0.9930049	2.7522136
H	8.3352660	-1.3638122	2.2987258
H	5.7927551	1.5318459	-2.4168932
H	3.5067601	1.5908665	-3.9163047
H	2.1312194	-0.6893777	-3.2643816

H	4.3554170	-3.7274007	0.6206792
H	3.7672697	-3.1738225	1.9191633
H	1.5301827	0.1499841	0.6245325
H	5.3218097	1.8719742	0.3745633
H	4.3987826	4.0622209	-0.2013717
H	-5.0171959	2.1758367	-1.0684665
H	-3.8626977	-1.3158422	1.1690277
H	-8.0149251	-0.1905123	0.9538625
H	-2.1292696	-0.7284426	-2.4297264
H	-1.5505618	-0.5067184	1.8276869
H	0.3621745	-3.6316105	-0.4280031
H	-1.9526738	1.7748544	-1.7598617
H	-2.6090553	2.8648807	0.2057829
H	-2.9628921	0.8462935	2.4752331
H	-3.8000062	2.3760516	2.2012158
H	-1.8078507	3.6682019	2.8377239
H	-1.7700252	2.3262424	4.0087207
H	0.4066493	2.7630660	2.4075446
H	-0.1418981	1.0935187	2.6760934
H	3.3403795	5.5903814	-1.4578651
H	3.2590186	6.0595788	0.2668493
H	-0.0857645	4.5756103	0.6519243
H	-0.1679002	4.7528610	-1.1260506
H	2.0818387	7.9244718	-0.7215595
H	1.4015723	6.9093552	-2.0178967
H	0.6243576	6.8585628	0.9604377

H	-0.4786646	7.0530009	-0.4270917
H	7.6354557	-0.7307004	-1.9198251

TS_3a_1

Energy= -3448.69460385132

N	2.6959237	5.0344407	-0.2800157
C	3.7023883	6.0472991	-0.6716602
C	2.8607101	7.2493722	-1.1037295
C	1.6176715	7.1381892	-0.2116804
C	1.3478319	5.6318150	-0.1935534
C	3.0445202	3.7926862	0.1133706
C	2.1063543	2.7578998	0.4549750
C	2.5770960	1.5401338	0.9278384
N	3.8844197	1.2521993	1.0352084
C	4.7873089	2.1979224	0.6986540
C	4.4213324	3.4416394	0.2420130
C	4.5100977	-0.2443739	1.9175736
O	4.5535822	0.0172154	3.1212440
C	0.6513627	2.8367094	0.0986183
O	0.3339984	2.8757352	-1.1130061
C	5.7659760	-0.5920860	1.1425155
C	5.8275461	-0.7441416	-0.2557441
C	7.0472361	-1.0466290	-0.8784957
C	8.2088936	-1.1864674	-0.1201035
C	8.1588949	-1.0401230	1.2697479
C	6.9435482	-0.7454132	1.8869998

N	-0.2563525	2.8163469	1.0987595
C	-1.7095784	3.0270971	0.8449895
C	-2.2933274	3.2210847	2.2568931
C	-1.1203240	3.7814018	3.0681692
C	0.0769423	2.9979409	2.5329545
C	-2.4035518	1.9614513	-0.0984565
O	-2.2400339	2.3771362	-1.4524328
C	-3.9151061	1.9923724	0.1713051
C	-4.7010885	2.9496690	-0.4801313
C	-6.0739495	3.0239991	-0.2277746
C	-6.6903784	2.1566647	0.6759143
C	-5.9011997	1.2061295	1.3242645
C	-4.5298432	1.1160873	1.0703110
C	-6.8803706	4.0545819	-0.9770392
F	-6.8813819	3.8115665	-2.3232633
C	-6.5094538	0.2260792	2.2943273
F	-6.4951819	-1.0503151	1.7998737
C	-1.8622050	0.5355184	0.0774275
C	-1.8662488	-0.3197859	-1.0301070
C	-1.4379743	-1.6440152	-0.9053749
C	-0.9809912	-2.1446013	0.3150818
C	-0.9742701	-1.2897919	1.4152806
C	-1.4124979	0.0332316	1.3024099
C	-1.4480791	-2.5142929	-2.1359137
F	-0.5384825	-2.0785173	-3.0652687
C	-0.4778465	-1.7481460	2.7614567

F	0.6068882	-1.0202846	3.1655232
F	-5.8129828	0.1863807	3.4703779
F	-7.7985565	0.5187501	2.6014375
F	-6.3689354	5.3117059	-0.8153338
F	-8.1792493	4.0986048	-0.5815878
F	-1.1476904	-3.8107837	-1.8681053
F	-2.6632263	-2.5028202	-2.7590996
F	-0.1270790	-3.0541694	2.7883263
F	-1.4341652	-1.5700990	3.7296107
H	9.1502187	-1.4241781	-0.6182925
H	6.8812110	-0.6134688	2.9680150
H	9.0627126	-1.1543129	1.8701996
H	1.8841385	0.7428310	1.2050774
H	5.8379896	1.9264984	0.8110548
H	5.2054229	4.1537208	-0.0036830
H	-4.2314072	3.6265593	-1.1936712
H	-3.9447248	0.3494434	1.5790774
H	-7.7602171	2.2187768	0.8689307
H	-2.2077315	0.0655771	-1.9910296
H	-1.4052875	0.6584987	2.1932583
H	-0.6264057	-3.1682924	0.4057679
H	-1.2700280	2.6113384	-1.5266906
H	-1.8099688	3.9713755	0.2837762
H	-2.6349629	2.2686833	2.6863504
H	-3.1609342	3.8912716	2.2338321
H	-0.9894889	4.8545287	2.8624204

H	-1.2488523	3.6507963	4.1496733
H	1.0338115	3.5226812	2.6368798
H	0.1744373	2.0178630	3.0281346
H	4.3440604	5.6601301	-1.4762072
H	4.3392720	6.2955309	0.1947909
H	0.8464812	5.3058612	0.7292489
H	0.7337260	5.3229272	-1.0535178
H	3.4046339	8.1935747	-0.9745809
H	2.5805512	7.1541560	-2.1636409
H	1.8431626	7.4946991	0.8047849
H	0.7559952	7.7001634	-0.5934891
N	4.6742345	-0.5680635	-1.0664938
C	4.6882916	-0.0215535	-2.3332723
C	3.3960471	-1.0714111	-0.8161567
C	3.4257913	-0.1543114	-2.8888267
H	5.5924411	0.4283067	-2.7305136
C	2.6183434	-0.8313618	-1.9454887
H	3.1227797	0.2142334	-3.8647655
H	1.5678943	-1.0900529	-2.0351897
C	2.9201639	-1.6679419	0.4285191
O	3.4108801	-1.2596155	1.6272221
O	2.0061212	-2.4916951	0.4345434
H	7.0777971	-1.1905957	-1.9590874

TS_3a_2

Energy= -3525.08569731210

N	2.5377065	4.8184157	0.3237555
C	3.4283611	6.0033810	0.2998291
C	2.4952107	7.1542234	-0.0796497
C	1.1519480	6.7229265	0.5217307
C	1.1184687	5.2219500	0.2296778
C	2.9950387	3.5807020	0.5626868
C	2.1769773	2.3969749	0.5505237
C	2.7156564	1.1812082	0.9190570
N	4.0169363	1.0438865	1.2644881
C	4.8365900	2.1343322	1.2359085
C	4.3732882	3.3724089	0.8870528
C	4.6666871	-0.2428052	1.8344316
O	4.7626989	-0.2617096	3.0553829
C	0.8037676	2.3481997	-0.0556374
O	0.6720586	2.6170071	-1.2697611
C	5.7080327	-0.8370830	0.9181208
C	5.6150687	-0.9582889	-0.4851059
C	6.6505842	-1.5611847	-1.2055451
C	7.7804015	-2.0579035	-0.5544240
C	7.8817431	-1.9454434	0.8347315
C	6.8534239	-1.3421209	1.5566605
N	4.4944833	-0.4696765	-1.2406507
C	3.4844639	-1.2501223	-1.8166897
C	2.7818393	-0.4288991	-2.6917153
C	3.3780616	0.8570191	-2.6670403
C	4.4494592	0.7966799	-1.7925481

C	3.2777823	-2.6968574	-1.5270689
O	3.6093091	-3.1249281	-0.3433637
O	2.8022232	-3.4225570	-2.4254962
N	-0.2190766	1.9548322	0.7361738
C	-1.6301038	1.9788839	0.2572848
C	-2.4496026	1.8109263	1.5512922
C	-1.5375863	2.3745436	2.6446287
C	-0.1509065	1.8963984	2.2180505
C	-1.9690378	1.0126579	-0.9457119
O	-1.6220740	1.6589304	-2.1698273
C	-3.4963400	0.8426138	-0.9788917
C	-4.2718537	1.8210472	-1.6129669
C	-5.6653925	1.7244725	-1.6107670
C	-6.3144929	0.6591335	-0.9818706
C	-5.5368133	-0.3134932	-0.3548769
C	-4.1406222	-0.2307981	-0.3585268
C	-6.4667691	2.7639069	-2.3532442
F	-6.4719372	2.5257879	-3.7020859
C	-6.1770495	-1.4801870	0.3526454
F	-5.7222472	-2.6749663	-0.1302247
C	-1.2860683	-0.3632499	-0.8785045
C	-1.1088295	-1.0586463	-2.0807648
C	-0.5672508	-2.3462444	-2.0876857
C	-0.1662574	-2.9641492	-0.9021776
C	-0.3428013	-2.2709396	0.2959381
C	-0.9057877	-0.9897935	0.3124407

C	-0.4714370	-3.0690999	-3.4093694
F	0.3106990	-2.4019081	-4.3081237
C	-0.0262894	-2.9420937	1.6077139
F	0.3654433	-2.0509557	2.5646161
F	-5.8866299	-1.4745223	1.6906155
F	-7.5296803	-1.4908239	0.2394988
F	-5.9578500	4.0177900	-2.1856142
F	-7.7674338	2.8022234	-1.9579995
F	0.0152572	-4.3265639	-3.2967112
F	-1.7094155	-3.1765075	-3.9951085
F	0.9495588	-3.8775200	1.4990859
F	-1.1309745	-3.5826824	2.1139055
O	3.1706339	-1.4724009	1.4021394
H	8.5780844	-2.5272223	-1.1327091
H	6.9193855	-1.2506361	2.6415175
H	8.7614386	-2.3250543	1.3570561
H	5.1884904	1.5424581	-1.5168534
H	3.0501760	1.7429597	-3.2032148
H	1.9142007	-0.7432959	-3.2633899
H	3.1521104	-1.9474880	2.2531364
H	3.4223827	-2.2303638	0.6083854
H	2.1420404	0.2567954	0.9122463
H	5.8778050	1.9666113	1.5091979
H	5.0798740	4.1983252	0.8885290
H	-3.7743515	2.6515410	-2.1128471
H	-3.5575581	-1.0168137	0.1230185

H	-7.4012624	0.5887948	-0.9822677
H	-1.4047857	-0.5786719	-3.0139442
H	-1.0496684	-0.5017250	1.2733349
H	0.2781355	-3.9577638	-0.9151837
H	-0.7247748	2.0692138	-2.0052176
H	-1.8161929	2.9839073	-0.1588042
H	-2.6733803	0.7529546	1.7497779
H	-3.4092209	2.3358479	1.4781676
H	-1.5715391	3.4743956	2.6448648
H	-1.8052110	2.0203598	3.6476067
H	0.6667557	2.5261792	2.5864807
H	0.0428880	0.8640419	2.5519937
H	4.2441871	5.8531489	-0.4209752
H	3.8655867	6.1540494	1.3011890
H	0.5168854	4.6596033	0.9577013
H	0.7316498	5.0145455	-0.7794007
H	2.8566284	8.1137816	0.3100632
H	2.4165670	7.2329984	-1.1743351
H	1.1428722	6.8989326	1.6079624
H	0.2908179	7.2390439	0.0792215
H	6.5527779	-1.6404158	-2.2896259

4a

Energy= -817.75230993799

C	-0.6951468	0.7496041	-0.0998936
C	-3.4130431	0.5164294	-0.7314990

C	-1.3701497	-0.4581417	0.1737056
C	-1.3767899	1.8189683	-0.6843228
C	-2.7306765	1.7026527	-1.0093565
C	-2.7366060	-0.5493378	-0.1363895
H	-3.2496557	2.5444939	-1.4706005
H	-3.2650168	-1.4786417	0.0763851
H	-4.4716579	0.4184259	-0.9767439
N	0.6998195	0.9310268	0.1729374
C	1.6240124	1.1132330	-0.8243594
H	1.3106181	1.1093011	-1.8640747
C	1.3600616	0.9513665	1.4027762
C	2.8834132	1.2520047	-0.2530792
H	3.8109900	1.4093962	-0.7962751
C	2.7184958	1.1526885	1.1436420
H	3.4894743	1.2314340	1.9046975
C	0.6800414	0.8332623	2.6804665
C	-0.6600291	-1.6466176	0.7280887
O	-0.5327040	0.7153767	2.8507952
O	0.5233789	-1.9044223	0.5589042
O	1.5611340	0.8684972	3.7238711
H	1.0321891	0.7925359	4.5456599
O	-1.4924046	-2.4532294	1.4335495
H	-0.9751493	-3.2385263	1.7142083
H	-0.8379918	2.7493678	-0.8696813

4a-A

Energy= -1585.18861616822

C	3.2315034	-0.9685381	-4.2289934
C	4.6223296	1.1767792	-5.3673417
C	2.5954628	0.2848942	-4.3441107
C	4.5401953	-1.1412509	-4.6870091
C	5.2348730	-0.0748666	-5.2625025
C	3.3191349	1.3500429	-4.9020201
H	6.2555721	-0.2230280	-5.6193629
H	2.8302476	2.3227969	-4.9748309
H	5.1586192	2.0175118	-5.8103733
N	2.5738356	-2.1158028	-3.6722259
C	2.1086217	-3.1504097	-4.4401040
H	2.2451130	-3.1331182	-5.5173775
C	2.2520316	-2.3688172	-2.3380838
C	1.4804949	-4.0771031	-3.6143971
H	1.0062212	-4.9979176	-3.9425042
C	1.5736240	-3.5893109	-2.2958296
H	1.1796582	-4.0454507	-1.3931004
C	2.6246510	-1.4882534	-1.2477537
C	1.1966517	0.5793089	-3.8889885
O	3.1687910	-0.3885057	-1.3511379
O	0.3871407	-0.4795568	-3.8988778
O	2.3040969	-2.0263263	-0.0330866
O	0.8387843	1.7129951	-3.5687526
N	-2.0382930	-0.0032623	-2.8195597

C	-2.3173840	1.1478207	-2.4556778
N	-2.5500356	2.3274666	-2.2575910
C	-2.8800498	-1.1967880	-2.5602131
C	-2.1933825	3.1441095	-1.0714304
C	-2.1999087	-2.1378659	-1.5767734
C	-2.3274423	-3.5228779	-1.7567430
C	-1.5177076	-1.6577122	-0.4509570
C	-1.7995567	-4.4135978	-0.8177084
H	-2.8454290	-3.9070649	-2.6397792
C	-0.9849112	-2.5471203	0.4857162
H	-1.3858476	-0.5836663	-0.3069536
C	-1.1283193	-3.9270066	0.3096986
H	-1.9031116	-5.4898055	-0.9724093
H	-0.4433919	-2.1568386	1.3490152
H	-0.7066970	-4.6211096	1.0398196
C	-1.3779095	2.3686134	-0.0515852
C	-0.0561351	1.9810690	-0.3282032
C	-1.9456821	2.0131728	1.1803814
C	0.6837876	1.2645264	0.6153305
H	0.3921115	2.2179420	-1.2954698
C	-1.2060522	1.2924208	2.1252476
H	-2.9763710	2.3025801	1.4024535
C	0.1132424	0.9217188	1.8468349
H	1.7100430	0.9758458	0.3780270
H	-1.6620550	1.0212264	3.0796263
H	0.6919038	0.3618919	2.5848139

C	-4.2888201	-0.8251190	-2.0864850
H	-4.2473216	-0.3100401	-1.1142325
H	-4.7893333	-0.1720895	-2.8163469
H	-4.8913096	-1.7352161	-1.9637048
H	-2.9534460	-1.7157550	-3.5286883
H	-0.5249705	-0.2286326	-3.5005270
C	-1.5074236	4.4234759	-1.5572592
H	-2.1585638	4.9604469	-2.2606008
H	-1.2957073	5.0784614	-0.7007581
H	-0.5605365	4.1916221	-2.0644038
H	-3.1551179	3.4200453	-0.6106781
H	2.5749323	-1.3727334	0.6439316
H	5.0130501	-2.1183996	-4.5748786

4a-B

Energy= -1585.1982958747979

C	3.1868035	-0.0994625	0.4614291
C	5.2597666	-0.5016183	-1.3895919
C	2.9726405	0.1856679	-0.9084618
C	4.4211616	-0.6040265	0.8802362
C	5.4479363	-0.8194669	-0.0403765
C	4.0338927	0.0053289	-1.8133875
H	6.4051666	-1.2146275	0.3035498
H	3.8674725	0.2430391	-2.8650295
H	6.0652065	-0.6514800	-2.1096696
N	2.1864780	0.1281878	1.4506098

C	1.6137796	-0.8712033	2.2138486
H	1.9853109	-1.8888665	2.1516753
C	1.4474627	1.2966889	1.6128565
C	0.5316587	-0.3373273	2.8907534
H	-0.1163714	-0.8795145	3.5732177
C	0.4188856	1.0241989	2.5051645
H	-0.2972201	1.7552343	2.8708315
C	1.8339381	2.5788192	0.9872328
C	1.6843018	0.6405905	-1.4962026
O	0.8326892	3.2818645	0.4534712
O	0.6060417	0.1311084	-0.8004770
O	2.9985403	2.9883852	0.9700104
O	1.5640345	1.3155166	-2.4990755
C	-0.6728456	0.6115085	-1.0682748
N	-0.9870037	1.7796631	-0.6178687
N	-1.4382871	-0.2798388	-1.7308482
C	-1.0401996	-1.6722806	-2.0207688
H	-2.4387852	-0.0816593	-1.7469510
C	-2.3597851	2.2801125	-0.8224564
C	-0.8173680	-2.4915532	-0.7563928
C	0.3112081	-3.3132845	-0.6474700
C	-1.7389610	-2.4574469	0.2990263
C	0.5131178	-4.0976294	0.4926609
H	1.0451423	-3.3305108	-1.4581124
C	-1.5366165	-3.2340671	1.4419028
H	-2.6109708	-1.8010105	0.2445346

C	-0.4123189	-4.0611565	1.5405084
H	1.4014261	-4.7287736	0.5667245
H	-2.2562191	-3.1882866	2.2623932
H	-0.2514126	-4.6644227	2.4364258
H	-2.5720568	2.2924416	-1.9081432
H	0.0401051	2.6379872	0.0987076
C	-3.4048768	1.3891016	-0.1552528
C	-4.5608698	1.0084195	-0.8530301
C	-3.2247613	0.9328022	1.1599127
C	-5.5184414	0.1832147	-0.2506427
H	-4.7101836	1.3549165	-1.8798638
C	-4.1801125	0.1125034	1.7633592
H	-2.3142823	1.1972570	1.7010087
C	-5.3299565	-0.2664648	1.0594909
H	-6.4089960	-0.1114082	-0.8096210
H	-4.0219919	-0.2431590	2.7837795
H	-6.0715758	-0.9156154	1.5290743
C	-2.4429149	3.7187064	-0.3032425
H	-3.4464878	4.1276156	-0.4826588
H	-2.2442225	3.7500737	0.7782359
H	-1.7068915	4.3551860	-0.8146518
C	-2.1188964	-2.2875531	-2.9164602
H	-2.2411124	-1.7022041	-3.8387401
H	-1.8365106	-3.3135226	-3.1845015
H	-3.0850034	-2.3299004	-2.3889526
H	-0.0948590	-1.6365953	-2.5823196

H	4.5770235	-0.7999638	1.9420984

4a-C

Energy= -1585.20000032599

C	3.3785290	0.6344617	0.0293941
C	3.5541757	3.0471792	1.4631284
C	2.2394985	1.1515975	0.6892797
C	4.5817581	1.3461598	0.0746042
C	4.6665563	2.5513720	0.7733118
C	2.3539852	2.3427274	1.4284936
H	5.6137166	3.0927966	0.7976968
H	1.4712097	2.7241147	1.9431740
H	3.6219298	3.9806145	2.0232176
N	3.3449340	-0.6005475	-0.6728499
C	3.6331501	-0.7228165	-2.0232960
H	4.0107218	0.1275958	-2.5830067
C	2.7881510	-1.7927436	-0.2133801
C	3.2805797	-1.9977480	-2.4243463
H	3.3989052	-2.4007408	-3.4269900
C	2.7384824	-2.6664287	-1.2896896
H	2.3815766	-3.6916903	-1.2390134
C	2.4385749	-2.0348911	1.2251261
C	0.8915463	0.5424251	0.6568425
O	1.3279232	-2.6576925	1.4286676
O	0.6135173	-0.0091304	-0.6179299

O	3.2258992	-1.6350516	2.1163351
O	0.0684462	0.5575217	1.5386823
H	5.4601547	0.9284345	-0.4193411
C	-0.5163003	-0.7561853	-0.7840309
N	-0.6240537	-1.9018243	-0.1560150
N	-1.3920643	-0.2588151	-1.6543694
C	-1.3332366	1.1146792	-2.2097311
H	-2.2310310	-0.8052716	-1.8443058
C	-1.8317713	-2.7482137	-0.2458864
C	-3.0698196	-1.9936688	0.2245735
C	-4.2301064	-1.9891337	-0.5620355
C	-3.0633588	-1.2954719	1.4417618
C	-5.3704975	-1.2941010	-0.1414307
H	-4.2410922	-2.5293849	-1.5128381
C	-4.2014993	-0.6052018	1.8635042
H	-2.1543959	-1.2673398	2.0461073
C	-5.3576503	-0.6009009	1.0726048
H	-6.2654377	-1.2909404	-0.7665828
H	-4.1835228	-0.0574533	2.8077952
H	-6.2429463	-0.0523299	1.3999553
C	-1.5060279	2.1604223	-1.1188094
C	-0.5808731	3.2050640	-0.9987083
C	-2.5828346	2.0922424	-0.2230802
C	-0.7281444	4.1709910	0.0028857
H	0.2700669	3.2542533	-1.6831924
C	-2.7291964	3.0524165	0.7794248

H	-3.2982351	1.2691257	-0.2887136
C	-1.8019962	4.0955536	0.8949571
H	0.0050014	4.9749981	0.0935128
H	-3.5654478	2.9825319	1.4782754
H	-1.9133330	4.8423894	1.6835750
C	-2.3975703	1.2222432	-3.3020539
H	-2.2291690	0.4719891	-4.0875425
H	-3.4059185	1.0838839	-2.8823380
H	-2.3545255	2.2203130	-3.7556216
H	-0.3388570	1.2405399	-2.6621152
C	-1.5912399	-4.0275695	0.5583173
H	-0.7095967	-4.5641580	0.1812252
H	-1.4341697	-3.7953142	1.6209955
H	-2.4679875	-4.6810296	0.4683913
H	-1.9742959	-3.0260255	-1.3053220
H	0.2147405	-2.2434748	0.4624226

TS_4a_1

Energy= -1585.17053311700

C	3.8610967	-0.6457924	0.3415617
C	6.0971919	0.0434896	-1.1825799
C	3.7207931	0.2840544	-0.7048092
C	5.1006852	-1.2281732	0.6178672
C	6.2175362	-0.8947316	-0.1527606
C	4.8589709	0.6350231	-1.4437266
H	7.1819056	-1.3578831	0.0630016

H	4.7572559	1.3706087	-2.2438241
H	6.9678779	0.3165448	-1.7815291
N	2.7401604	-1.0246414	1.1532179
C	2.1870605	-2.2774663	1.1340319
H	2.6199070	-3.0555838	0.5127551
C	1.9646552	-0.2046292	1.9734891
C	1.0629650	-2.2864087	1.9533565
H	0.4277605	-3.1468072	2.1449620
C	0.9181063	-0.9840044	2.4758289
H	0.1629370	-0.6346709	3.1736757
C	2.3368113	1.1586036	2.3151219
C	2.3921862	0.8960342	-1.0770859
O	3.4100173	1.7040379	2.0721031
O	2.3387307	2.1422158	-1.2921081
O	1.3281128	1.7992739	2.9838648
O	1.4044076	0.0850954	-1.1728424
N	-0.3005549	2.0886985	-1.0573487
C	-0.5365935	0.8257140	-1.1970569
N	-1.2996148	-0.1010414	-1.3505299
C	-1.3084973	3.0566180	-0.5152323
C	-1.3629163	-1.4318546	-0.7396238
C	-2.3006028	2.3696670	0.4026092
C	-1.8717593	1.7321427	1.5780553
C	-3.6625266	2.3569903	0.0778205
C	-2.7917675	1.0826590	2.4027228
H	-0.8118691	1.7223705	1.8419605

C	-4.5858572	1.7146824	0.9096024
H	-4.0008223	2.8400568	-0.8425272
C	-4.1518201	1.0732821	2.0720655
H	-2.4463390	0.5748301	3.3058653
H	-5.6429238	1.6993145	0.6371992
H	-4.8671937	0.5509912	2.7099040
C	-2.7847384	-1.7218531	-0.2904170
C	-3.8887330	-1.3784803	-1.0823870
C	-2.9984738	-2.3830007	0.9260917
C	-5.1843541	-1.6962018	-0.6666699
H	-3.7321576	-0.8412265	-2.0203095
C	-4.2935784	-2.7066601	1.3422645
H	-2.1447297	-2.6368588	1.5596386
C	-5.3909591	-2.3630802	0.5462779
H	-6.0374027	-1.4141444	-1.2877715
H	-4.4456143	-3.2159850	2.2963482
H	-6.4040297	-2.6063769	0.8736174
C	-0.5365535	4.1897003	0.1625848
H	0.0255338	3.8181231	1.0310872
H	0.1678647	4.6577247	-0.5400645
H	-1.2431464	4.9563224	0.5066608
H	-1.8559169	3.4602164	-1.3817444
H	0.7094749	2.3776074	-1.2260688
C	-0.8463428	-2.4753557	-1.7380891
H	-1.4611852	-2.4792482	-2.6495236
H	0.1970740	-2.2556298	-2.0012260

H -0.8950224 -3.4725740 -1.2783567

H -0.7053298 -1.4350478 0.1423986

H 1.6729430 2.6794032 3.2433162

H 5.1855243 -1.9298397 1.4495200

TS_4a_2

Energy= -1585.19783542568

C 2.9362214 -0.0579474 -0.0578387

C 4.6813202 -0.7341806 -2.1545420

C 2.4844507 -0.0019879 -1.3987575

C 4.2416417 -0.4781958 0.2138034

C 5.1052536 -0.8291018 -0.8246261

C 3.3838177 -0.3146715 -2.4343106

H 6.1207170 -1.1548790 -0.5930298

H 3.0328994 -0.2522654 -3.4653784

H 5.3589280 -0.9902475 -2.9700103

N 2.1136429 0.3199683 1.0404631

C 1.7430376 -0.5393883 2.0596133

H 2.1460150 -1.5459373 2.1012603

C 1.3638914 1.4889414 1.1400121

C 0.7804842 0.0882754 2.8280613

H 0.2942105 -0.3361473 3.7017791

C 0.5341004 1.3608774 2.2450005

H -0.1367562 2.1338327 2.6103903

C 1.5563512 2.6480931 0.2306480

C 1.1011294 0.3320446 -1.8214136

O	0.4513954	3.1985329	-0.2306997
O	0.1730028	-0.0701738	-0.8699043
O	2.6906692	3.0633644	-0.0547304
O	0.7762742	0.8213673	-2.8830534
C	-1.1413963	0.3461592	-0.9780759
N	-1.4184290	1.5791294	-0.6859327
N	-1.9892740	-0.6378910	-1.3191041
C	-1.6092704	-2.0609447	-1.4471164
H	-2.9827077	-0.4395435	-1.1952338
C	-2.8178864	2.0484817	-0.7142689
C	-1.1316088	-2.6525801	-0.1274000
C	0.0304467	-3.4328358	-0.0937184
C	-1.8505613	-2.4476565	1.0583286
C	0.4647141	-4.0094699	1.1042243
H	0.6068061	-3.5809676	-1.0108602
C	-1.4148629	-3.0156445	2.2575105
H	-2.7452587	-1.8201845	1.0559891
C	-0.2586665	-3.8034465	2.2833926
H	1.3763693	-4.6107364	1.1177876
H	-1.9766679	-2.8372169	3.1769134
H	0.0846586	-4.2435190	3.2218809
H	-3.2170295	1.8821080	-1.7320186
H	-0.4370023	2.4236448	-0.3692595
C	-3.6984032	1.2772109	0.2654328
C	-4.9324147	0.7562103	-0.1518024
C	-3.2839367	1.0671223	1.5901181

C	-5.7378378	0.0327791	0.7366760
H	-5.2628777	0.9113012	-1.1829134
C	-4.0872527	0.3486856	2.4780326
H	-2.3132264	1.4438049	1.9178115
C	-5.3161957	-0.1726392	2.0536502
H	-6.6915008	-0.3744449	0.3951429
H	-3.7491191	0.1843614	3.5031922
H	-5.9384261	-0.7425003	2.7464312
C	-2.8423622	3.5527720	-0.4289595
H	-3.8736788	3.9248362	-0.4884000
H	-2.4550095	3.7626350	0.5784947
H	-2.2288351	4.0954216	-1.1616587
C	-2.8155317	-2.8146932	-2.0129601
H	-3.1192081	-2.3953991	-2.9825156
H	-2.5569132	-3.8720418	-2.1517665
H	-3.6676196	-2.7637372	-1.3172960
H	-0.7848250	-2.1181270	-2.1730987
H	4.5828287	-0.4987646	1.2498464

TS_4a_3

Energy= -1585.19150829123

C	3.8675394	-0.8550646	0.3745304
C	2.8136838	-3.4258512	0.6875249
C	2.4725953	-1.0453135	0.3473532
C	4.7248024	-1.9509109	0.5383963
C	4.1976966	-3.2327953	0.7011534

C	1.9580776	-2.3367665	0.5038654
H	4.8737519	-4.0804782	0.8237171
H	0.8795479	-2.4844780	0.4859803
H	2.3965609	-4.4272727	0.8091315
N	4.4227522	0.4519139	0.2536484
C	5.5456305	0.8667865	0.9355334
H	6.0007075	0.2270164	1.6849925
C	4.0437257	1.4711036	-0.6325163
C	5.8880637	2.1401719	0.5074360
H	6.7119707	2.7355309	0.8904735
C	4.9560734	2.5134389	-0.4863172
H	4.9076512	3.4509687	-1.0330868
C	2.8921022	1.4792523	-1.5309567
C	1.6355303	0.1990039	0.1845250
O	2.8571091	2.1892350	-2.5373272
O	0.0834881	-0.4816018	-0.1676978
O	1.8220320	0.6926118	-1.2595310
O	1.5858950	1.0755922	1.0565878
H	5.8044572	-1.7982129	0.5186541
N	-0.8769415	1.6195207	0.0015093
C	-0.9250754	0.3164874	-0.3153417
N	-2.0536631	-0.2258452	-0.8200192
C	-1.9685713	2.5830960	-0.1992769
C	-2.1831122	-1.6690635	-1.0649483
C	-3.2179894	2.1945801	0.5846038
C	-4.4631064	2.1520799	-0.0586952

C	-3.1379473	1.8533571	1.9437639
C	-5.6127671	1.7675394	0.6418604
H	-4.5312971	2.4119381	-1.1189933
C	-4.2847091	1.4731024	2.6442672
H	-2.1699569	1.8664237	2.4505435
C	-5.5245794	1.4252349	1.9940126
H	-6.5740184	1.7279438	0.1258078
H	-4.2106583	1.2035136	3.6997379
H	-6.4174517	1.1159421	2.5405190
C	-2.1299996	-2.4926292	0.2180669
C	-2.6876654	-2.0244550	1.4157149
C	-1.5339767	-3.7616719	0.1977173
C	-2.6456302	-2.8086472	2.5727891
H	-3.1435433	-1.0320137	1.4566866
C	-1.4926768	-4.5496419	1.3526142
H	-1.0864343	-4.1324955	-0.7287977
C	-2.0476408	-4.0737883	2.5455679
H	-3.0770192	-2.4279655	3.5012907
H	-1.0170962	-5.5322785	1.3225083
H	-2.0102113	-4.6830679	3.4508332
H	-0.0018157	1.9145261	0.4518378
H	-2.9142541	0.3137758	-0.7305484
C	-1.4577791	3.9744978	0.1815663
H	-1.1817692	4.0099144	1.2459833
H	-0.5809545	4.2448296	-0.4243425
H	-2.2453258	4.7182071	0.0057200

H	-2.2254563	2.5865810	-1.2725132
C	-3.4842874	-1.9022433	-1.8374715
H	-4.3537185	-1.5922149	-1.2368774
H	-3.4803893	-1.3383225	-2.7808142
H	-3.5946992	-2.9698860	-2.0655922
H	-1.3321188	-1.9674971	-1.6947800

2a

Energy = -741.33694540424

C	-3.3012641	0.4129825	0.2475763
C	-1.9617505	0.3265155	-0.1248034
C	-1.0530573	1.3316344	0.2387117
C	-1.5002298	2.4245948	1.0089674
C	-2.8606650	2.5001120	1.3647585
C	-3.7571855	1.5030355	0.9966088
H	-3.9877858	-0.3842272	-0.0411118
H	-3.1956908	3.3569936	1.9497832
H	-4.8040600	1.5736528	1.2937811
N	0.2853931	1.2393887	-0.2236076
C	0.6582663	0.5578142	-1.3616909
C	1.4476664	1.5430632	0.4999511
C	2.0382417	0.4353235	-1.3851059
H	-0.0867460	0.2243641	-2.0763081
C	2.5357053	1.0352021	-0.2097415
H	2.6173400	-0.0225224	-2.1817500
H	3.5736564	1.1461784	0.0907852

C	1.5557679	2.3318726	1.7040371
O	2.5101220	2.2977844	2.4599013
O	0.6023839	3.3273470	1.9818876
C	-0.6625734	3.5612667	1.4555178
O	-1.0673091	4.7034705	1.5288991
H	-1.6123230	-0.5377298	-0.6886407

2a-A

Energy= -3448.70242287637

C	2.1977604	1.0260947	-0.6814828
C	4.2066520	1.9464524	-1.2269973
C	1.5507989	2.2674184	-0.6573622
H	1.6068920	0.1223632	-0.5037608
C	3.6916794	3.2313057	-1.2144086
H	5.2639454	1.7913853	-1.4648026
C	2.3239344	3.4511055	-0.8849762
H	4.3517519	4.0673845	-1.4391282
C	-2.7146621	0.6419423	-0.5469142
C	-4.1602575	0.3306532	-0.1355801
C	-4.6857584	-0.9643265	-0.0910115
C	-5.0147573	1.4097388	0.1279862
C	-6.0231989	-1.1738594	0.2575756
C	-6.3489926	1.1915964	0.4759678
H	-4.6406794	2.4312417	0.0541247
C	-6.8676723	-0.1021009	0.5483368
H	-7.9086176	-0.2694898	0.8258521

C	-1.8201013	-0.6028982	-0.6667921
C	-1.6912950	-1.5392613	0.3692299
C	-1.0423819	-0.7802863	-1.8146658
C	-0.8104428	-2.6178906	0.2527504
H	-2.2941111	-1.4548330	1.2731476
C	-0.1442051	-1.8480068	-1.9126538
H	-1.1413857	-0.0704958	-2.6362642
C	-0.0150753	-2.7789314	-0.8826164
H	0.6880581	-3.6066758	-0.9568590
O	-2.8374984	1.3089092	-1.8058751
H	-2.0501311	1.9258732	-1.8738729
C	-2.0523106	1.6389900	0.4921981
C	-2.3267263	1.3684496	1.9867985
C	-1.0187319	1.7358712	2.7010099
H	-2.5682252	0.3117653	2.1604420
H	-3.1841432	1.9530617	2.3409257
C	0.0512993	1.3228026	1.6929275
H	-0.9644983	2.8204533	2.8777259
H	-0.9053293	1.2237585	3.6651205
H	1.0077528	1.8452822	1.8052770
H	0.2466073	0.2384978	1.7350308
N	-0.5709720	1.6690958	0.4012612
C	0.0604111	2.2482851	-0.6469300
O	-0.5751492	2.6868521	-1.6412338
C	2.6660306	5.9029897	-1.0152624
C	0.5573661	5.0723054	-0.1240285

C	1.7136568	7.0725131	-0.7545747
H	3.0671635	5.8956919	-2.0394539
H	3.5174069	5.9187182	-0.3112878
C	0.7785925	6.5180168	0.3282818
H	0.3530821	4.3917603	0.7169608
H	-0.2798308	4.9951203	-0.8358440
H	2.2521903	7.9765994	-0.4426360
H	1.1439433	7.3082561	-1.6666525
H	1.2782830	6.5355383	1.3089958
H	-0.1664890	7.0699060	0.4115622
N	1.8304905	4.7097811	-0.7734167
C	-7.2494093	2.3763029	0.7173171
C	-6.5942792	-2.5679063	0.3139036
C	-0.7350158	-3.5988959	1.3946382
C	0.6992416	-1.9559223	-3.1590630
F	-0.1398958	-3.0426698	2.4997805
F	-1.9793265	-3.9964451	1.7978178
F	-0.0370221	-4.7171534	1.0876535
F	-0.0749740	-1.9735159	-4.2884036
F	1.4622022	-3.0773367	-3.1825827
F	1.5420737	-0.8905831	-3.2886138
F	-7.7232493	2.8954139	-0.4576020
F	-8.3361822	2.0579875	1.4704738
F	-6.6010386	3.3915738	1.3550768
F	-7.2067924	-2.8122918	1.5113128
F	-5.6512019	-3.5306023	0.1451984

F	-7.5442401	-2.7655678	-0.6497720
C	8.3272667	0.0616063	0.6292319
C	7.1175953	-0.0408402	1.3108657
C	5.9641049	-0.5132842	0.6647362
C	6.0418212	-0.9205981	-0.6837011
C	7.2740087	-0.7995716	-1.3552049
C	8.4099810	-0.3181572	-0.7138975
H	9.2095655	0.4268293	1.1570552
H	7.3178277	-1.1035982	-2.4012897
H	9.3537723	-0.2454971	-1.2557578
N	4.7494977	-0.5219747	1.3926051
C	4.4720237	0.3334980	2.4359146
C	3.7252012	-1.4700619	1.3224393
C	3.2841079	-0.0546899	3.0349158
H	5.1307409	1.1641910	2.6638316
C	2.8237176	-1.1993580	2.3517599
H	2.8029189	0.4511583	3.8669266
H	1.9197959	-1.7702375	2.5399283
C	3.5314115	-2.5046319	0.3307362
O	2.8283451	-3.4856675	0.5245277
O	4.0275338	-2.3664954	-0.9603382
C	4.9313802	-1.4515824	-1.5151648
O	4.8943792	-1.3510794	-2.7225099
H	-4.0629364	-1.8224209	-0.3366876
H	-2.4174242	2.6399247	0.2111707
N	3.4940492	0.8349692	-0.9491844

H 7.0691708 0.2337303 2.3640713

2a_B

Energy= -3448.7000492431262

C	2.1912331	1.0086397	-0.8968459
C	4.3281123	1.9902158	-0.8680696
C	1.5745560	2.2189689	-0.6925399
H	1.6375352	0.0783803	-1.0150956
C	3.7841306	3.2312111	-0.6866232
H	5.4011549	1.8347983	-0.9616812
C	2.3704620	3.4128308	-0.5520297
H	4.4633073	4.0771610	-0.6234016
C	-2.6549919	0.5547707	-0.7477710
C	-4.0824347	0.1604003	-0.3500721
C	-4.5215336	-1.1612822	-0.2706423
C	-5.0072785	1.1897749	-0.1193744
C	-5.8516905	-1.4487568	0.0647458
C	-6.3241503	0.8952116	0.2297072
H	-4.6971521	2.2305378	-0.2172049
C	-6.7632616	-0.4292793	0.3261533
H	-7.7934068	-0.6567845	0.5970967
C	-1.7012609	-0.6471293	-0.8657574
C	-1.4471602	-1.5074755	0.2148240
C	-1.0273757	-0.8780490	-2.0668810
C	-0.5268510	-2.5489736	0.0977872

H	-1.9711517	-1.3821015	1.1612377
C	-0.1006508	-1.9228049	-2.1746419
H	-1.2288513	-0.2328287	-2.9216666
C	0.1651534	-2.7634204	-1.0962499
H	0.8948691	-3.5669145	-1.1807187
O	-2.8084589	1.2170123	-2.0068021
H	-2.0173828	1.8181604	-2.1022389
C	-2.0699704	1.5914244	0.2992635
C	-2.3963957	1.3509127	1.7890955
C	-1.1312263	1.7792272	2.5452453
H	-2.6175193	0.2931760	1.9816520
H	-3.2836260	1.9195998	2.0920714
C	-0.0032624	1.3763624	1.5978822
H	-1.1250749	2.8687272	2.7001452
H	-1.0406179	1.2935895	3.5250280
H	0.9210535	1.9489295	1.7371401
H	0.2480358	0.3071240	1.6825305
N	-0.5883384	1.6631927	0.2687431
C	0.0721967	2.1843565	-0.7807021
O	-0.4893691	2.5582546	-1.8371398
C	2.7181152	5.8409349	-0.2153820
C	0.4607004	4.9449593	0.0354568
C	1.7118601	6.9857176	-0.0924943
H	3.3515636	5.9214914	-1.1093442
H	3.3679341	5.7745831	0.6733345
C	0.5394650	6.3413269	0.6563748

H	0.0557595	4.2010046	0.7335108
H	-0.1493100	4.9408503	-0.8806368
H	2.1413712	7.8466858	0.4343770
H	1.3928257	7.3157739	-1.0922355
H	0.7654045	6.2640731	1.7304579
H	-0.4043952	6.8882605	0.5398509
N	1.8683898	4.6281393	-0.2941139
C	-7.2896675	2.0316897	0.4529618
C	-6.2650905	-2.8956992	0.1561549
C	-0.3644965	-3.5220077	1.2388387
C	0.5614003	-2.1516742	-3.5098187
F	-0.6258047	-2.9647574	2.4486755
F	-1.2574251	-4.5669538	1.1005701
F	0.8673306	-4.0808042	1.2910668
F	-0.3367611	-2.6464617	-4.4247139
F	1.5842991	-3.0390599	-3.4517957
F	1.0465158	-0.9958761	-4.0452289
F	-7.7760700	2.5209939	-0.7292503
F	-8.3663825	1.6634173	1.1969435
F	-6.6991916	3.0820039	1.0919290
F	-5.6220788	-3.5377351	1.1791096
F	-5.9515070	-3.5837830	-0.9822069
F	-7.5979045	-3.0526620	0.3637532
C	8.0240019	-1.5121687	0.1713186
C	7.1751626	-0.7968266	1.0177253
C	5.8934175	-0.4156972	0.5981677

C	5.4514186	-0.7749139	-0.6957026
C	6.3123586	-1.5118399	-1.5260017
C	7.5916654	-1.8733927	-1.1075332
H	9.0168398	-1.7988186	0.5217053
H	5.9539415	-1.7916172	-2.5173861
H	8.2447132	-2.4380150	-1.7746571
N	5.1102580	0.3587202	1.5052364
C	5.6516310	1.4431235	2.1795822
C	3.8148945	0.1244665	1.9948245
C	4.7145556	1.9208477	3.0749918
H	6.6504659	1.7931274	1.9382920
C	3.5716451	1.0873052	2.9676071
H	4.8406474	2.7855631	3.7209238
H	2.6451289	1.1599704	3.5298618
C	2.8752841	-0.9416060	1.5392697
O	1.9150360	-1.2302950	2.2951474
O	3.0791437	-1.4743627	0.3840784
C	4.1038483	-0.4818996	-1.2591208
O	3.5993781	-1.0776046	-2.1950936
H	-3.8363293	-1.9817414	-0.4830300
H	-2.4511388	2.5748142	-0.0196951
N	3.5417835	0.8804227	-0.9551896
H	7.4951662	-0.5435173	2.0290947

2a-C

Energy= -3525.0990705426693

N	2.7703470	-4.2893364	4.1122840
C	3.5678008	-4.9724832	5.1643560
C	2.6160658	-6.0354801	5.7137477
C	1.2372724	-5.3882355	5.5444374
C	1.3471889	-4.6995170	4.1838290
C	3.3027065	-3.3839158	3.2834149
C	2.6024711	-2.7686770	2.1817869
C	3.2528451	-1.8742671	1.3747452
N	4.5673405	-1.5393411	1.5651814
C	5.2598627	-2.0873146	2.6161551
C	4.6674377	-2.9774895	3.4577279
C	5.1627689	-0.5521869	0.6674846
O	4.4186954	0.2727573	0.1681658
C	1.1547303	-3.0081496	1.8246429
O	0.2845429	-2.2172145	2.2604361
C	6.6283239	-0.5839827	0.4620317
C	7.4163275	-1.7485369	0.2815690
C	8.7925752	-1.6134655	0.0637014
C	9.3908682	-0.3544461	0.0190358
C	8.6140097	0.7999373	0.1648885
C	7.2431774	0.6797749	0.3670464
N	6.8821730	-3.0649597	0.3256288
C	5.7364981	-3.5626660	-0.3071517
C	5.5710193	-4.8697052	0.1305661
C	6.6269592	-5.1820463	1.0302101
C	7.4185647	-4.0557866	1.1404458

C	4.9374775	-2.8168749	-1.3416205
O	5.5786035	-1.9680715	-2.0524284
O	3.7117996	-3.0918295	-1.4049251
N	0.8901262	-4.0413373	1.0099846
C	-0.4953878	-4.4511261	0.6639462
C	-0.3089454	-5.8079080	-0.0469841
C	1.0725833	-6.3025560	0.3983494
C	1.8901225	-5.0186044	0.5186917
C	-1.3328931	-3.3438638	-0.1100308
O	-1.9513624	-2.4910182	0.8531150
C	-2.5011467	-4.0391348	-0.8218031
C	-3.4965132	-4.6168342	-0.0156503
C	-4.5639710	-5.3010483	-0.5928097
C	-4.6775032	-5.4181898	-1.9835357
C	-3.6998373	-4.8290223	-2.7784173
C	-2.6229155	-4.1367198	-2.2069741
C	-5.6509045	-5.8826895	0.2760060
F	-6.0913606	-7.0847186	-0.1931108
C	-3.7723324	-4.9109450	-4.2817610
F	-4.7832838	-5.7045385	-4.7198141
C	-0.4209757	-2.5363691	-1.0515651
C	0.3534609	-3.1351068	-2.0567699
C	1.2041181	-2.3707210	-2.8563783
C	1.3232937	-0.9941004	-2.6700677
C	0.5425064	-0.3972154	-1.6800506
C	-0.3236882	-1.1524656	-0.8826347

C	1.9313617	-3.0385903	-3.9948215
F	1.1229119	-3.0704048	-5.1174032
C	0.6827954	1.0776412	-1.4072151
F	-0.4784225	1.6315477	-0.9509883
F	-3.9554824	-3.6792300	-4.8470785
F	-2.6132131	-5.4059121	-4.8121172
F	-6.7465906	-5.0631123	0.3270764
F	-5.2460105	-6.0768856	1.5598664
F	3.0618419	-2.3963466	-4.3636229
F	2.2486089	-4.3346511	-3.7327089
F	1.0527606	1.7825394	-2.5113295
F	1.6305886	1.3248012	-0.4497662
O	4.3388553	0.2949330	-2.8419979
H	10.4656496	-0.2764893	-0.1530806
H	6.6221223	1.5691615	0.4813540
H	9.0744312	1.7875222	0.1170240
H	8.2792265	-3.8443290	1.7675420
H	6.7881557	-6.1208367	1.5537604
H	4.7827046	-5.5323545	-0.2141177
H	4.2186060	0.6693784	-1.9506605
H	4.7084727	-0.6166517	-2.6340018
H	2.7765828	-1.4091089	0.5144958
H	6.2812668	-1.7418776	2.7569202
H	5.2580290	-3.3564737	4.2872875
H	-3.4323257	-4.5184883	1.0676173
H	-1.8869014	-3.6691282	-2.8605029

H	-5.5103275	-5.9588634	-2.4321587
H	0.3168219	-4.2096627	-2.2265427
H	-0.9290907	-0.6669562	-0.1190814
H	2.0442960	-0.4123890	-3.2459785
H	-1.0405310	-4.5958802	1.6119060
H	-0.3412953	-5.6925610	-1.1382220
H	-1.1158889	-6.5003478	0.2201671
H	1.0031887	-6.8044628	1.3753789
H	1.5174135	-7.0092757	-0.3136254
H	2.7253410	-5.0985324	1.2239555
H	2.3018807	-4.6633093	-0.4386554
H	3.8481723	-4.2413944	5.9395980
H	4.4827444	-5.3964554	4.7288036
H	1.1235782	-5.4060886	3.3691455
H	0.6909187	-3.8263480	4.0937753
H	2.6858855	-6.9536653	5.1115669
H	2.8522170	-6.2875079	6.7548489
H	0.4113472	-6.1100921	5.5633515
H	1.0626410	-4.6392457	6.3306450
H	9.3881165	-2.5118973	-0.1029832
H	-1.2342667	-2.2842379	1.5183288

TS_2a_1

Energy= -3448.6957812026199

C	2.1621983	1.1693898	-0.4877911
C	4.1381868	2.3187398	-0.8725000

C	1.4231287	2.3424354	-0.4293317
H	1.6725472	0.1961767	-0.4171544
C	3.4943500	3.5321264	-0.8347871
H	5.2111991	2.2543503	-1.0578030
C	2.0929651	3.6069803	-0.5719625
H	4.0828327	4.4336662	-0.9875441
C	-2.7919294	0.5390547	-0.1209175
C	-4.2091264	0.1994046	0.3557802
C	-4.6750753	-1.1107895	0.4729083
C	-5.1042801	1.2532468	0.5911629
C	-6.0011846	-1.3628075	0.8477611
C	-6.4184553	0.9935706	0.9782808
H	-4.7767867	2.2856458	0.4648958
C	-6.8834119	-0.3185140	1.1118579
H	-7.9115635	-0.5176776	1.4111666
C	-1.8523418	-0.6771650	-0.1532478
C	-1.6145457	-1.4644601	0.9828189
C	-1.1534048	-0.9792873	-1.3245314
C	-0.6846547	-2.5061372	0.9486050
H	-2.1637797	-1.2853889	1.9069766
C	-0.2210107	-2.0223018	-1.3485477
H	-1.3395957	-0.3887746	-2.2215352
C	0.0306470	-2.7930367	-0.2143702
H	0.7737379	-3.5867092	-0.2279025
O	-2.9819245	1.0807017	-1.4313369
H	-2.2044034	1.6866908	-1.5984429

C	-2.1456604	1.6547626	0.7995891
C	-2.3884179	1.5376788	2.3198296
C	-1.0761600	1.9993582	2.9696999
H	-2.6126806	0.5030057	2.6082735
H	-3.2479771	2.1455799	2.6258387
C	-0.0125298	1.5124694	1.9884775
H	-1.0435739	3.0968259	3.0403686
H	-0.9347732	1.5861714	3.9763251
H	0.9335886	2.0625873	2.0357717
H	0.2041003	0.4406048	2.1277113
N	-0.6653007	1.7165548	0.6798467
C	-0.0695595	2.1996042	-0.4299455
O	-0.7090897	2.4832948	-1.4716235
C	2.2100383	6.0782097	-0.6821132
C	0.1234763	5.0514694	0.0528457
C	1.1203699	7.1436012	-0.5520829
H	2.6937864	6.0771781	-1.6692500
H	2.9891921	6.2045911	0.0891824
C	0.1519165	6.5255514	0.4638820
H	-0.0912152	4.3837279	0.8985678
H	-0.6215563	4.8682953	-0.7365152
H	1.5313339	8.1079553	-0.2283538
H	0.6170935	7.2899931	-1.5198425
H	0.5514851	6.6264549	1.4844173
H	-0.8498046	6.9730311	0.4385590
N	1.4874039	4.8054082	-0.4589995

C	-7.3553433	2.1539683	1.1989406
C	-6.4436304	-2.7980286	0.9768530
C	-0.4668367	-3.2982397	2.2127119
C	0.4904061	-2.3083464	-2.6464227
F	0.0814542	-2.5283360	3.2027934
F	-1.6535361	-3.7673478	2.7135626
F	0.3400948	-4.3715983	2.0395449
F	-0.3761880	-2.8052264	-3.5883374
F	1.4879344	-3.2163132	-2.5143565
F	1.0315420	-1.1789383	-3.1855945
F	-7.8315327	2.6505385	0.0154377
F	-8.4394324	1.8148174	1.9458447
F	-6.7387848	3.1924397	1.8327389
F	-5.8115857	-3.4282432	2.0139608
F	-6.1476488	-3.5199767	-0.1448249
F	-7.7791220	-2.9218260	1.1915689
C	8.3725209	0.1745162	0.4006573
C	7.2956505	0.3343969	1.2717772
C	5.9816772	0.1609248	0.8120906
C	5.7426623	-0.1883600	-0.5297462
C	6.8397193	-0.3538091	-1.3860808
C	8.1466967	-0.1769388	-0.9336612
H	9.3892272	0.3101273	0.7731033
H	6.6364223	-0.6134880	-2.4259999
H	8.9848995	-0.3114761	-1.6191541
N	4.9094609	0.3553439	1.7250258

C	4.9274483	1.2632335	2.7634262
C	3.7964882	-0.4747609	1.8821774
C	3.8299676	1.0321021	3.5771131
H	5.7167888	2.0047158	2.8343871
C	3.1312841	-0.0733259	3.0375078
H	3.5622058	1.6106344	4.4568436
H	2.2198458	-0.5319014	3.4102719
C	3.3468335	-1.5250317	0.9709344
O	2.6726283	-2.4713300	1.3797558
O	3.5785932	-1.4404902	-0.3608453
C	4.3679397	-0.3687614	-1.1434594
O	4.2276260	-0.5377291	-2.3551952
H	-4.0139799	-1.9501522	0.2576293
H	-2.5395144	2.6123091	0.4230115
N	3.4925526	1.1482957	-0.6741905
H	7.4713218	0.5738183	2.3210942

TS_2a_2

Energy= -3525.08462686574

N	-12.7605340	3.8544828	-0.0788395
C	-13.9899186	3.0935011	-0.4109363
C	-14.9950570	4.1776521	-0.8006494
C	-14.5754552	5.3660605	0.0718377
C	-13.0479231	5.3040623	0.0206673
C	-11.6008616	3.2468420	0.2226639
C	-10.3577973	3.9286580	0.4685185

C	-9.2290661	3.2079178	0.7941067
N	-9.2249323	1.8556866	0.8618138
C	-10.3803668	1.1734466	0.6285291
C	-11.5430691	1.8191594	0.3083891
C	-8.0589666	1.0371531	1.4730835
O	-8.1045704	0.9349894	2.6953538
C	-10.1601548	5.4231746	0.4429217
O	-10.1434665	6.0593127	1.5233705
C	-7.5665927	-0.0769177	0.5776958
C	-7.3784375	-0.0104011	-0.8189219
C	-6.8382720	-1.1021783	-1.5066337
C	-6.4716138	-2.2648843	-0.8290528
C	-6.6590506	-2.3446593	0.5534053
C	-7.1993436	-1.2599928	1.2415666
N	-7.7519681	1.1291428	-1.6168584
C	-6.8737457	2.0445586	-2.2122866
C	-7.5862633	2.7146520	-3.2006354
C	-8.9036096	2.1918022	-3.2285135
C	-8.9709472	1.2005214	-2.2642261
C	-5.4360097	2.1957734	-1.8602284
O	-5.0613723	1.8445163	-0.6614923
O	-4.6565802	2.6424859	-2.7256838
N	-9.9555142	6.0015606	-0.7548724
C	-9.9291972	7.4770527	-0.9287532
C	-10.0980782	7.6726563	-2.4525184
C	-10.6130960	6.3277279	-2.9850711

C	-9.9578173	5.3074599	-2.0589565
C	-8.6723391	8.1755258	-0.2646387
O	-8.9451433	8.3966632	1.1198682
C	-8.5011655	9.5873272	-0.8468753
C	-7.2769552	10.0869764	-1.2925125
C	-7.1800877	11.3913864	-1.7939821
C	-8.2944453	12.2232564	-1.8531020
C	-9.5150632	11.7306337	-1.3808686
C	-9.6179195	10.4369793	-0.8699338
C	-5.8384031	11.8582984	-2.2969356
F	-5.8357694	13.1661111	-2.6606818
C	-10.7336114	12.6183860	-1.3772163
F	-11.8697833	11.9257343	-1.6763353
C	-7.4248405	7.2904210	-0.4488760
C	-6.8386245	6.6801078	0.6645206
C	-5.7093243	5.8671286	0.5215821
C	-5.1222106	5.6574687	-0.7274815
C	-5.6921441	6.2851838	-1.8354197
C	-6.8380621	7.0758625	-1.7044164
C	-5.0624252	5.3111506	1.7660371
F	-4.2240000	4.2756200	1.5087342
C	-5.0546282	6.1877797	-3.2006484
F	-4.8745928	7.4354557	-3.7415522
F	-10.9387313	13.1895215	-0.1503755
F	-10.6374754	13.6374410	-2.2729724
F	-5.4355600	11.1374086	-3.3881965

F	-4.8629466	11.7006995	-1.3532132
F	-4.3188413	6.2717668	2.4043720
F	-5.9806575	4.8757236	2.6760972
F	-5.8368426	5.5047360	-4.0901499
F	-3.8428172	5.5886849	-3.1781133
O	-6.6650467	2.3333418	1.0971748
H	-6.0467790	-3.1045324	-1.3816996
H	-7.3425632	-1.3064344	2.3217590
H	-6.3841279	-3.2501362	1.0968272
H	-9.7721149	0.5165958	-2.0035084
H	-9.7205411	2.4987604	-3.8767313
H	-7.1754773	3.5088818	-3.8161116
H	-6.1791109	2.2397678	1.9373723
H	-5.9316721	2.0500522	0.2605358
H	-8.2687811	3.6802423	0.9901720
H	-10.3310546	0.0886424	0.7178374
H	-12.4287789	1.2133382	0.1363511
H	-6.3800756	9.4700141	-1.2478068
H	-10.5765771	10.0881865	-0.4851139
H	-8.2179927	13.2325758	-2.2549422
H	-7.2597742	6.8596729	1.6532951
H	-7.2359718	7.5629627	-2.5941190
H	-4.2368155	5.0327257	-0.8357066
H	-9.4416795	7.5872514	1.4373310
H	-10.8027328	7.8768257	-0.3889256
H	-9.1415075	7.9282956	-2.9250256

H -10.7859441 8.4993385 -2.6656444

H -11.7081428 6.2694104 -2.8988520

H -10.3479841 6.1621916 -4.0367191

H -10.4899219 4.3530777 -1.9940671

H -8.9191115 5.0902802 -2.3541733

H -14.3277894 2.5326964 0.4765340

H -13.7886670 2.3801804 -1.2221130

H -12.6687732 5.8278518 -0.8708376

H -12.5750945 5.7296825 0.9140063

H -14.8885755 4.4281411 -1.8671170

H -16.0282466 3.8535716 -0.6251152

H -14.9522947 6.3297174 -0.2925231

H -14.9223633 5.2254165 1.1065889

H -6.7053361 -1.0220044 -2.5870058

TS_anhydride_rotation_H

Energy= -741.3296557343024

C -0.7146654 -0.7294691 -0.0006005

C -3.5283129 -0.4043733 0.2657971

C -1.2717540 0.5687476 0.1582407

C -1.6010200 -1.8254412 -0.0449785

C -2.9752845 -1.6741384 0.0818965

C -2.6766289 0.6855409 0.3023827

H -3.6106376 -2.5601155 0.0415496

H -3.0810194 1.6882942 0.4308102

H -4.6043453 -0.2679072 0.3768742

N	0.6786778	-1.0173207	-0.0791300
C	1.7850225	-0.1555930	0.0621092
C	1.1825975	-2.3054793	-0.1581110
H	0.5422999	-3.1698575	-0.2661297
C	2.5604490	-2.2796334	-0.0683782
H	3.2081477	-3.1502705	-0.1109887
C	2.9421689	-0.9351317	0.0748183
H	3.9471807	-0.5314432	0.1493073
C	1.8542831	1.2806553	0.0153900
C	-0.6015806	1.8926884	0.1524206
O	0.7228730	2.0623785	-0.2223698
O	-1.1928645	2.9332831	0.3774051
O	2.9014869	1.9024096	0.1019653
H	-1.2234656	-2.8355648	-0.1669306

TS_COOH_H_crossing_H

Energy= -817.72744355965

C	-0.7839491	0.8340130	0.0006254
C	-3.5152451	0.4011240	-0.6688639
C	-1.3777488	-0.4456060	0.1589026
C	-1.5702307	1.8413329	-0.5817200
C	-2.9067153	1.6307017	-0.9168088
C	-2.7418793	-0.6245324	-0.1332394
H	-3.4704176	2.4473213	-1.3708354
H	-3.1797716	-1.6122689	0.0222269
H	-4.5663651	0.2317013	-0.9056102

N	0.5634136	1.1141392	0.4140545
C	1.4061387	0.1705359	0.9803973
H	1.0375797	-0.7668424	1.3687614
C	1.3695885	2.2303918	0.0768040
C	2.7051355	0.6292488	0.9840568
H	3.5625875	0.0961845	1.3841532
C	2.6874705	1.9155308	0.4034521
H	3.5209923	2.6054734	0.3101911
C	0.9627548	3.6316624	-0.0823356
C	-0.6720287	-1.7435360	0.4180982
O	-0.0301264	4.1746630	0.3968809
O	0.1023858	-2.2691787	-0.3671775
O	1.9043224	4.3300978	-0.7730941
H	1.6629864	5.2790166	-0.7101325
O	-1.0750237	-2.3216432	1.5715150
H	-0.6719882	-3.2165428	1.6292934
H	-1.1547010	2.8259222	-0.7544364

Water

Energy= -76.38704064037

O	7.3401120	-7.1266454	0.2992336
H	8.3117943	-7.0763270	0.2992336
H	7.0631981	-6.1939072	0.2992336

1b

Energy= -1277.1766243995542

C	-0.6890168	-0.3155472	-0.4357674
C	-3.4163183	-0.3834369	0.2322917
C	-1.4114677	0.8861706	-0.2830932
C	-1.3544889	-1.5347338	-0.2408829
C	-2.7120381	-1.5777031	0.0834526
C	-2.7688651	0.8404204	0.0640216
H	-3.2043203	-2.5398829	0.2258294
H	-3.3190764	1.7725881	0.1864755
H	-4.4761387	-0.4112657	0.4882973
N	0.6952259	-0.3153214	-0.7820196
C	1.1568318	-0.5633960	-2.0481709
H	0.4603814	-0.7914500	-2.8492919
C	1.7810795	-0.0271586	0.0474077
C	2.5406020	-0.4435529	-2.0497814
H	3.1863920	-0.5867379	-2.9115334
C	2.9334033	-0.1075276	-0.7368451
H	3.9439650	0.0531097	-0.3725875
C	1.6357636	0.2473540	1.4661772
C	-0.7552015	2.2112280	-0.5119151
O	0.5809742	0.2317967	2.0996289
O	0.1395364	2.4284689	-1.3145761
O	2.8351762	0.5363728	2.0496194
H	2.6593570	0.6987232	3.0003830
O	-1.2943229	3.1746741	0.2734603

H	-0.8678400	4.0260319	0.0348749
Cl	-0.4679007	-3.0463534	-0.3641143

4b

Energy= -1277.1766336562284

C	-0.6783809	0.7574163	-0.1015952
C	-3.4210188	0.5165492	-0.6520315
C	-1.3331863	-0.4769422	0.0878134
C	-1.4212815	1.8554657	-0.5587582
C	-2.7833415	1.7413547	-0.8434545
C	-2.7057516	-0.5818027	-0.1756532
H	-3.3359392	2.6097003	-1.2026332
H	-3.2075651	-1.5362783	-0.0195915
H	-4.4853166	0.4233211	-0.8714090
N	0.7154956	0.9091845	0.1619226
C	1.6724309	0.9298342	-0.8191853
H	1.3873052	0.8483258	-1.8638184
C	1.3415986	1.0019767	1.4068875
C	2.9212525	1.0420437	-0.2216382
H	3.8714236	1.0872843	-0.7463948
C	2.7153736	1.0869981	1.1737421
H	3.4703535	1.1846635	1.9485066
C	0.6043347	1.0447619	2.6575220
C	-0.5761692	-1.6837661	0.5441428
O	-0.6204665	1.0455554	2.7733470
O	0.5883999	-1.9324647	0.2737945

O	1.4418103	1.0846875	3.7345203
H	0.8782755	1.1244342	4.5356293
O	-1.3540706	-2.5037022	1.2916989
H	-0.8216873	-3.2977957	1.5145389
Cl	-0.6473944	3.4214203	-0.7465342

TS_COOH_Cl_crossing

Energy= -1277.1305871288025

C	-0.8377102	0.9676626	-0.0332613
C	-3.6070393	0.3498414	-0.4370123
C	-1.3605911	-0.3499292	0.1570378
C	-1.7277439	1.8734755	-0.6624264
C	-3.0864980	1.5774493	-0.8247379
C	-2.7255777	-0.6274185	0.0157250
H	-3.7270180	2.3294405	-1.2859863
H	-3.0762914	-1.6430477	0.2007723
H	-4.6696234	0.1371146	-0.5554140
N	0.4850059	1.2503276	0.4330490
C	1.1825827	0.3088835	1.1938151
H	0.6634806	-0.4687784	1.7391100
C	1.4478067	2.1837068	-0.0386288
C	2.5290681	0.5681041	1.1503918
H	3.3004903	0.0050522	1.6675113
C	2.7011796	1.7349534	0.3631437
H	3.6219301	2.2947085	0.2197179
C	1.2665021	3.6497351	-0.0975003

C	-0.5041903	-1.5821392	0.2154699
O	0.6206761	4.3013354	0.7136378
O	0.3334270	-1.8669153	-0.6264522
O	2.0458713	4.2113272	-1.0539301
H	2.0307238	5.1832236	-0.9166918
O	-0.8492520	-2.4000814	1.2364299
H	-0.3379626	-3.2343455	1.1481211
Cl	-1.2343326	3.3761610	-1.4316626

Hydration of Fuel

F-1

Energy=-843.81228011455

C	0.7413866	-1.3464241	0.5960580
N	0.0141623	-1.4573637	1.5733714
N	1.5586050	-1.3509125	-0.3291827
C	-1.4730292	-1.5099689	1.5567359
C	2.1019318	-0.1316752	-0.9967898
H	-1.7852885	-1.0893556	2.5232861
C	-1.9206927	-2.9735499	1.4986292
H	-1.6089078	-3.4472273	0.5564903
H	-1.4831961	-3.5400530	2.3320972
H	-3.0160010	-3.0300243	1.5702900
C	-2.0597284	-0.6449706	0.4535313
C	-1.9452994	-1.0039979	-0.9001137
C	-2.6966888	0.5599164	0.7799066

C	-2.4498884	-0.1701104	-1.9015671
H	-1.4528166	-1.9373232	-1.1829395
C	-3.2094653	1.3931089	-0.2195950
H	-2.7838248	0.8525342	1.8294513
C	-3.0846249	1.0313910	-1.5641333
H	-2.3485861	-0.4600812	-2.9495591
H	-3.6992044	2.3302057	0.0533078
H	-3.4775466	1.6829165	-2.3472727
C	1.7291403	1.1338446	-0.2464976
C	2.4052914	1.5210866	0.9213603
C	0.6428656	1.9036858	-0.6858223
C	2.0056715	2.6582316	1.6281703
H	3.2480303	0.9308887	1.2881879
C	0.2403930	3.0407685	0.0198359
H	0.0948394	1.5981566	-1.5808812
C	0.9229588	3.4228816	1.1782718
H	2.5419981	2.9495337	2.5339957
H	-0.6129879	3.6234493	-0.3330404
H	0.6112032	4.3111792	1.7318826
H	1.6066009	-0.1018630	-1.9809201
C	3.6004833	-0.3261814	-1.2229680
H	3.7769473	-1.2470652	-1.7964379
H	4.0056217	0.5234730	-1.7897462
H	4.1445731	-0.4033212	-0.2715760
H	1.2990378	-3.7095268	-2.4893673
O	1.8652087	-3.8302159	-1.7081071

H	1.7830747	-2.9746341	-1.2140513

TS_F_W_1

Energy=-843.75480033082

C	0.2566458	-2.8307066	0.3690604
N	-0.7616223	-2.6887396	0.9996608
N	1.3685420	-2.5040548	-0.1978553
C	-2.1779650	-3.0076946	0.8027628
C	1.6698477	-1.1692317	-0.7990421
H	-2.6735991	-2.7793927	1.7566550
C	-2.3562269	-4.4986612	0.4957039
H	-1.8954224	-4.7594800	-0.4670614
H	-1.8918693	-5.1120245	1.2796034
H	-3.4286014	-4.7311918	0.4482575
C	-2.7650444	-2.0931947	-0.2646053
C	-2.3249934	-2.1610634	-1.5952021
C	-3.7477549	-1.1575427	0.0802577
C	-2.8594340	-1.3077120	-2.5626735
H	-1.5518118	-2.8787082	-1.8823514
C	-4.2878477	-0.3032046	-0.8875636
H	-4.0900351	-1.0927177	1.1163182
C	-3.8431847	-0.3750067	-2.2107715
H	-2.5050551	-1.3668507	-3.5938610
H	-5.0523380	0.4234323	-0.6044826
H	-4.2587595	0.2942852	-2.9668001
C	0.9241030	-0.0661385	-0.0752896

C	1.2229614	0.2584702	1.2578133
C	-0.1043392	0.6253997	-0.7291792
C	0.5054253	1.2572906	1.9199156
H	2.0120517	-0.2799941	1.7877380
C	-0.8244176	1.6257543	-0.0684097
H	-0.3556863	0.3662948	-1.7601386
C	-0.5207567	1.9441249	1.2582016
H	0.7454005	1.4999373	2.9573378
H	-1.6301985	2.1467521	-0.5889038
H	-1.0831099	2.7219260	1.7789164
H	1.3095595	-1.2046328	-1.8400206
C	3.1881720	-0.9957781	-0.8095204
H	3.6657077	-1.8272035	-1.3471109
H	3.4453784	-0.0581673	-1.3206168
H	3.5927730	-0.9613052	0.2115388
H	0.6740685	-5.2667954	-0.5661665
O	1.2196624	-4.8515180	0.1251967
H	1.8182218	-3.4890409	-0.3676072

F-2

Energy=-843.82932218110

C	-0.0241934	-2.1904347	-0.0235951
N	-0.5496890	-1.7625973	1.0693576
N	0.4458442	-1.5895426	-1.1608860
C	-0.7777015	-0.3581891	1.3899253
C	0.7280875	-0.1733957	-1.3826215

H	0.1312899	0.2409970	1.2001731
C	-1.0665385	-0.2625782	2.8979754
H	-1.9697783	-0.8402381	3.1461414
H	-0.2209484	-0.6745028	3.4676073
H	-1.2225055	0.7816538	3.2043210
C	-1.9133771	0.2858780	0.5973895
C	-2.8905283	-0.4750288	-0.0588197
C	-1.9932501	1.6876727	0.5275288
C	-3.9219165	0.1488376	-0.7707424
H	-2.8336899	-1.5645364	-0.0175074
C	-3.0184115	2.3135173	-0.1858611
H	-1.2328809	2.2948365	1.0270518
C	-3.9895052	1.5440576	-0.8390215
H	-4.6733191	-0.4595376	-1.2796296
H	-3.0569673	3.4039438	-0.2374387
H	-4.7890312	2.0302217	-1.4021101
C	1.8865118	0.3729132	-0.5539437
C	2.9004225	-0.4532576	-0.0536984
C	1.9485722	1.7530082	-0.3011202
C	3.9575105	0.0888469	0.6863850
H	2.8585079	-1.5296893	-0.2331748
C	3.0021512	2.2968862	0.4372588
H	1.1558916	2.4061857	-0.6776823
C	4.0124368	1.4641196	0.9343645
H	4.7391768	-0.5680408	1.0741788
H	3.0322472	3.3713224	0.6308458

H	4.8343032	1.8853225	1.5170890
H	-0.1746475	0.3994298	-1.1215999
C	0.9854735	0.0231573	-2.8832007
H	0.1137517	-0.3015710	-3.4692703
H	1.1793195	1.0817775	-3.0975833
H	1.8661802	-0.5562586	-3.2019379
O	0.0970574	-3.5571749	-0.1725224
H	-0.2967978	-3.9393396	0.6373793
H	0.8776358	-2.2334008	-1.8203557

TS_F_W_2

Energy=-843.79527993115

C	0.4656374	-2.8530532	0.2679210
N	-0.4857549	-2.0726317	0.7650497
N	1.6315667	-2.6138330	-0.3615113
C	-0.6588474	-0.6276296	0.7391326
C	2.2735985	-1.3100082	-0.5477271
H	0.2474741	-0.1411931	1.1429798
C	-1.8283496	-0.2767669	1.6717810
H	-2.7528666	-0.7527985	1.3127697
H	-1.6205335	-0.6334846	2.6908208
H	-1.9849173	0.8101658	1.7026437
C	-0.8982987	-0.0607747	-0.6582355
C	-1.4476278	-0.8376333	-1.6874476
C	-0.5762579	1.2806505	-0.9221632
C	-1.6701258	-0.2866566	-2.9541680

H	-1.6913810	-1.8845998	-1.4943335
C	-0.7944293	1.8331339	-2.1870941
H	-0.1387765	1.8953411	-0.1300581
C	-1.3434678	1.0494358	-3.2092294
H	-2.0957502	-0.9060970	-3.7468994
H	-0.5305500	2.8758561	-2.3777236
H	-1.5100187	1.4769814	-4.2001669
C	2.7744299	-0.6842433	0.7495287
C	3.0897624	-1.4536518	1.8763563
C	2.9458180	0.7082377	0.8075364
C	3.5696093	-0.8431237	3.0407583
H	2.9505493	-2.5364241	1.8491758
C	3.4248374	1.3200769	1.9683746
H	2.6919921	1.3199735	-0.0628790
C	3.7389896	0.5441869	3.0909255
H	3.8079229	-1.4552232	3.9133070
H	3.5471888	2.4048111	1.9997170
H	4.1083648	1.0202868	4.0015386
H	1.5273841	-0.6292888	-0.9870253
C	3.4129569	-1.4810582	-1.5599682
H	3.0273086	-1.8652861	-2.5148619
H	3.9015831	-0.5152233	-1.7415775
H	4.1705840	-2.1793694	-1.1719276
O	0.0613983	-4.1005851	0.4833559
H	-0.8788043	-3.3672756	0.9489703
H	2.1838914	-3.4401281	-0.5778217

Int_str_1_str_2

Energy= -1559.09684802155

C	0.6419110	0.1280021	3.6679300
C	3.1469245	-0.5688689	4.6927444
C	1.7358818	-0.0082162	2.7926928
C	0.8034448	-0.0705074	5.0393779
C	2.0511213	-0.4328434	5.5513238
C	2.9918017	-0.3451545	3.3245156
H	2.1688632	-0.5956593	6.6239644
H	3.8433584	-0.4476619	2.6511370
H	4.1252427	-0.8438751	5.0899257
N	-0.6312621	0.5870799	3.1863998
C	-0.9076988	1.9087608	2.9510891
H	-0.1430358	2.6638017	3.1089548
C	-1.7735815	-0.1657573	2.9247485
C	-2.2320892	2.0228447	2.5398340
H	-2.7366279	2.9499505	2.2828360
C	-2.7763416	0.7218178	2.5245043
H	-3.7845562	0.4324125	2.2427540
C	-1.7899813	-1.6164008	2.9922347
C	1.5581603	0.1231676	1.3172109
O	-0.8420821	-2.3369633	3.2972578
O	0.5672525	-0.2483018	0.7057386
O	-3.0165461	-2.1204188	2.6632727

H	-2.9505820	-3.0962338	2.7281048
O	2.6359751	0.6826844	0.7140583
H	2.4411700	0.7381377	-0.2545883
H	-0.0546960	0.0649767	5.6991551
C	-3.3167073	-0.7201843	-2.5576933
C	-2.3381707	-0.6101603	-3.5436673
C	-1.1669058	0.1226337	-3.3041157
C	-0.9804521	0.7396057	-2.0519147
C	-1.9831126	0.6276412	-1.0713247
C	-3.1396561	-0.1038468	-1.3142907
H	-4.2147139	-1.3056676	-2.7603973
H	-1.8161632	1.0977643	-0.1020032
H	-3.8980417	-0.1999050	-0.5360789
N	-0.2140006	0.2539560	-4.3490524
C	-0.5289439	0.1879721	-5.6879285
C	1.1798025	0.1551426	-4.2267298
C	0.6395128	0.0674416	-6.4239193
H	-1.5616671	0.2434204	-6.0159316
C	1.7123676	0.0270439	-5.5099360
H	0.7021706	0.0342651	-7.5076904
H	2.7734967	-0.0342126	-5.7341179
C	1.9655016	0.2775923	-3.0241492
O	3.1019086	-0.1377368	-2.8898920
O	1.4842385	1.0601753	-1.9537070
C	0.1961468	1.5420141	-1.6622336
O	0.1502549	2.5367495	-0.9752695

H -2.4724830 -1.1151622 -4.4998587

Prod_str_1_str_2

Energy= -1559.10061239891

C	0.6280545	0.6372625	3.2885403
C	2.4140189	-0.3676107	5.1954442
C	1.7441465	-0.1175118	2.8705061
C	0.4143696	0.8847646	4.6447035
C	1.2958954	0.3717786	5.5979913
C	2.6404884	-0.5997953	3.8410530
H	1.1169572	0.5641710	6.6570887
H	3.5061270	-1.1772979	3.5134933
H	3.1091708	-0.7613934	5.9381748
N	-0.2382746	1.2643899	2.3323277
C	0.1152554	2.3866137	1.6260906
H	1.0809058	2.8555027	1.7921892
C	-1.5216410	0.8767097	1.9502641
C	-0.9363481	2.7343480	0.7853438
H	-0.9465280	3.5822806	0.1064701
C	-1.9625100	1.7883410	0.9871061
H	-2.9247822	1.7407575	0.4855928
C	-2.1446041	-0.3485638	2.4208901
C	2.0311774	-0.4638162	1.4545578
O	-1.6300203	-1.1797188	3.1660960
O	0.8554087	-0.6464189	0.7337484
O	-3.4042567	-0.4956747	1.9176981

H	-3.7523105	-1.3446990	2.2617717
O	3.1320128	-0.6688192	0.9904954
H	1.5790987	-2.3450834	-4.3501268
H	-0.4420572	1.4905235	4.9437524
C	-2.7770554	-2.2364753	-2.2030402
C	-2.2034467	-1.1560870	-2.8784450
C	-1.0442400	-0.5546882	-2.3903893
C	-0.4539391	-1.0130810	-1.1924010
C	-1.0491505	-2.0965632	-0.5211292
C	-2.1933961	-2.7112567	-1.0256225
H	-3.6802080	-2.7034072	-2.5990835
H	-0.6033821	-2.4540956	0.4060829
H	-2.6346892	-3.5551092	-0.4935660
N	-0.5356676	0.5831344	-3.1018438
C	-1.0400166	1.8462753	-2.9250256
C	0.4713772	0.6320172	-4.0689026
C	-0.3646585	2.7214945	-3.7682755
H	-1.8385081	2.0182424	-2.2099034
C	0.5810599	1.9605056	-4.4851874
H	-0.5476941	3.7892812	-3.8491216
H	1.2837807	2.3134526	-5.2347839
C	1.2819068	-0.4806836	-4.5374853
O	2.1744315	-0.3635977	-5.3780948
O	0.9717343	-1.6762605	-3.9686410
C	0.7694093	-0.3785571	-0.6421815
O	1.5680287	0.3179862	-1.2232650

H -2.6478892 -0.7718792 -3.7974115

TS_str_1_str_2

Energy= -1559.06844061065

C	-6.9304261	7.5083411	9.0140827
C	-4.1370481	7.2750867	8.9867624
C	-6.2976236	6.8613009	7.9300163
C	-6.1659266	8.0343622	10.0572384
C	-4.7733879	7.9259541	10.0455449
C	-4.8957793	6.7435689	7.9458346
H	-4.1919692	8.3442832	10.8686629
H	-4.4027663	6.2394424	7.1153483
H	-3.0506647	7.1792758	8.9691210
N	-8.3503697	7.6854825	9.1088518
C	-8.9462808	8.9171080	9.0199697
H	-8.3530319	9.7930707	8.7749082
C	-9.3240087	6.7380681	9.4326081
C	-10.3064108	8.7790024	9.2732781
H	-11.0370540	9.5827000	9.2618960
C	-10.5431643	7.4152241	9.5384619
H	-11.4932034	6.9460042	9.7780068
C	-9.0400462	5.3213578	9.5881293
C	-7.0178101	6.3093799	6.7535673
O	-7.9429421	4.7834719	9.4486558
O	-8.2167125	6.6681812	6.5133246
O	-10.1657053	4.6185488	9.9025088

H	-9.9053904	3.6762911	9.9758776
O	-6.3469916	5.4891507	6.0088206
H	-6.9922792	5.1266665	5.1960852
H	-6.6783358	8.5187165	10.8895603
C	-12.8193983	4.8101280	5.2798652
C	-12.1773264	4.8138533	4.0396402
C	-10.9134229	5.3956621	3.8943236
C	-10.2910837	5.9668892	5.0274970
C	-10.9296670	5.9415002	6.2707213
C	-12.1982711	5.3757529	6.3954593
H	-13.8028521	4.3469465	5.3714639
H	-10.4328054	6.3790873	7.1333843
H	-12.6922094	5.3665309	7.3680593
N	-10.3125673	5.4220599	2.6028843
C	-11.0449428	5.7183651	1.4692601
C	-9.0180635	5.0369031	2.2028148
C	-10.2439531	5.5428972	0.3545180
H	-12.0742156	6.0522193	1.5558558
C	-8.9796184	5.1091173	0.8126634
H	-10.5426033	5.7274360	-0.6735375
H	-8.1015223	4.8824788	0.2145293
C	-7.8970953	4.6592775	3.0683648
O	-6.8726277	4.1385363	2.6123249
O	-8.0371380	4.9097055	4.3737580
C	-8.9815606	6.5940610	4.8267968
O	-8.5602884	7.4834195	4.1762851

H -12.6465967 4.3406589 3.1766216

Int_str_1_str_3

Energy=-1559.10062006303

C -3.0087023 -0.2092484 -0.1272940

C -5.0322167 1.6787478 -0.5098851

C -2.7146133 1.0057002 -0.7704398

C -4.3041654 -0.4792162 0.3166181

C -5.3107495 0.4712334 0.1406507

C -3.7433003 1.9381442 -0.9768561

H -6.3205330 0.2600023 0.4964290

H -3.5192563 2.8769710 -1.4853426

H -5.8219718 2.4167684 -0.6590752

N -2.0012344 -1.2241611 -0.0373117

C -1.4880889 -1.8595504 -1.1405269

H -1.8410937 -1.5988421 -2.1341456

C -1.4093623 -1.7614734 1.1047409

C -0.5646145 -2.8101725 -0.7235446

H 0.0091359 -3.4634438 -1.3743298

C -0.5153625 -2.7515106 0.6838632

H 0.1111589 -3.3439928 1.3440430

C -1.6360582 -1.2368088 2.4402009

C -1.3090927 1.3339202 -1.1557207

O -2.3252812 -0.2639280 2.7347658

O -0.3500266 1.1863307 -0.4113273

O	-0.9599055	-1.9635548	3.3826782
H	-1.1462349	-1.5468976	4.2499259
O	-1.2188828	1.8303166	-2.4100720
H	-4.5169854	-1.4392737	0.7885951
C	3.3197235	-2.8163874	-0.6607087
C	3.0142986	-1.9029798	0.3439661
C	2.5844595	-0.6046729	0.0302206
C	2.5026763	-0.2142780	-1.3236995
C	2.7957391	-1.1611801	-2.3253335
C	3.2063028	-2.4493335	-2.0055095
H	3.6564649	-3.8175944	-0.3876285
H	3.1209089	-2.1953159	1.3878018
H	2.7077455	-0.8516054	-3.3669925
H	3.4430034	-3.1603570	-2.7977950
N	2.1959424	0.2470265	1.0922563
C	1.7212940	-0.2076244	2.3014474
C	2.3936855	1.6292961	1.1976729
C	1.6026507	0.8614275	3.1749614
H	1.4779172	-1.2535069	2.4464270
C	2.0433802	2.0129007	2.4926792
H	1.2185594	0.8077606	4.1893763
H	2.0693551	3.0345206	2.8605863
C	2.7881400	2.5498961	0.1637216
O	3.2502460	3.6580916	0.3717406
O	2.5156084	2.2655437	-1.1883076
C	2.0917733	1.1146076	-1.8187640

O 1.5115687 1.2800705 -2.8823326

H -0.2582142 1.9642689 -2.6180781

TS_str_1_str_3

Energy=-1559.06590855637

C -2.4505356 1.1071547 0.8723463

C -2.6560342 3.5069683 2.3099154

C -1.4163562 2.0722404 0.7853975

C -3.5801461 1.3673495 1.6440899

C -3.6825826 2.5586720 2.3664434

C -1.5360828 3.2684958 1.5191155

H -4.5700833 2.7457097 2.9730994

H -0.7364718 4.0071038 1.4616642

H -2.7321689 4.4350976 2.8774935

N -2.4129970 -0.1122720 0.1184343

C -2.8244609 -0.2118275 -1.1863347

H -3.2731553 0.6420646 -1.6852155

C -1.8604354 -1.3317215 0.5115211

C -2.5482211 -1.4955902 -1.6431674

H -2.7669869 -1.8720776 -2.6383380

C -1.9445442 -2.1983659 -0.5797123

H -1.5972674 -3.2272667 -0.5831356

C -1.2913373 -1.5356914 1.8307417

C -0.2344785 1.8822450 -0.0617170

O -1.2291183 -0.6958950 2.7259658

O 0.0738868 0.7069724 -0.4652297

O	-0.8111114	-2.8063773	1.9849087
H	-0.4809209	-2.8742608	2.9052077
O	0.4663453	2.9299540	-0.3830171
H	-4.3758229	0.6223696	1.6798133
C	2.2001549	-3.4764725	-2.1689969
C	2.4849810	-2.7452398	-1.0164645
C	2.0894886	-1.4054677	-0.8974598
C	1.4063192	-0.8012503	-1.9713919
C	1.1520643	-1.5395987	-3.1412326
C	1.5395451	-2.8718280	-3.2419886
H	2.5155909	-4.5190570	-2.2324978
H	3.0404728	-3.2083585	-0.2007678
H	0.6198818	-1.0534462	-3.9593316
H	1.3237878	-3.4349861	-4.1507140
N	2.3924808	-0.7286652	0.3168363
C	2.3321435	-1.3695599	1.5406994
C	3.1057583	0.4711747	0.4839210
C	2.9646820	-0.5905615	2.4899581
H	1.8286004	-2.3264326	1.6284907
C	3.4678907	0.5538488	1.8241946
H	3.0357710	-0.8160662	3.5502803
H	4.0037391	1.3917733	2.2616335
C	3.3202598	1.5368285	-0.5066840
O	4.2830066	2.3079121	-0.3992712
O	2.3975450	1.7204251	-1.4554322
C	0.8542863	0.5560806	-1.9616375

O 0.3306411 1.1874316 -2.8271445

H 1.3310430 2.5988730 -0.8937361

Prod_str_1_str_3

Energy= -1559.0903084726317

C -3.0361646 -0.2365850 -0.2533685

C -4.5624995 2.1018982 0.0033334

C -2.4225256 1.0253896 -0.4480317

C -4.3970753 -0.3141035 0.0382111

C -5.1598893 0.8476641 0.1705005

C -3.2099688 2.1863633 -0.3091524

H -6.2232197 0.7711431 0.4031346

H -2.7361314 3.1574328 -0.4560031

H -5.1536756 3.0121417 0.1099041

N -2.3142341 -1.4674230 -0.4012683

C -2.0896849 -2.0793348 -1.6076890

H -2.5322654 -1.6792920 -2.5150995

C -1.6200581 -2.1589193 0.5924178

C -1.2552492 -3.1728193 -1.4035221

H -0.9095048 -3.8559296 -2.1741123

C -0.9594728 -3.2228298 -0.0248916

H -0.3404369 -3.9517235 0.4897933

C -1.6371408 -1.7469445 1.9848455

C -1.0139603 1.2295722 -0.8294854

O -2.2726057 -0.8053262 2.4519279

O -0.2036496 0.1247173 -0.6910802

O	-0.8369433	-2.5440690	2.7561899
H	-0.9380815	-2.2376835	3.6818808
O	-0.5373883	2.2999130	-1.1929257
H	-4.8502014	-1.2974334	0.1689474
C	4.2344434	-2.1338021	-0.1345653
C	3.6230468	-1.1505487	0.6461953
C	2.4898865	-0.4786941	0.1796318
C	1.9646379	-0.7954431	-1.0906787
C	2.6112298	-1.7617367	-1.8816576
C	3.7301676	-2.4373815	-1.4035277
H	5.1142555	-2.6541146	0.2471257
H	4.0276008	-0.8840057	1.6237334
H	2.2023585	-1.9911634	-2.8665503
H	4.2094889	-3.1998959	-2.0187148
N	1.9152405	0.5276639	1.0259878
C	1.3373843	0.2363476	2.2461817
C	2.1607035	1.9034463	0.9684375
C	1.1677305	1.4157871	2.9467903
H	1.1056450	-0.7928895	2.5020119
C	1.7065631	2.4639719	2.1535547
H	0.6992380	1.5130919	3.9221301
H	1.7362142	3.5237621	2.3925738
C	2.8241424	2.5947707	-0.1664637
O	3.8978596	3.1696868	-0.0554658
O	2.1872752	2.5868921	-1.3611912
C	0.7670190	-0.1557120	-1.6680831

O 0.5593012 0.0513765 -2.8417906

H 1.2055395 2.4118845 -1.2577068

1c_A_pbe0_TZVP

Energy = -1663.8408136795499

N -2.9278681 -1.3800055 2.4624764

C -2.6967907 -0.2530983 2.0986911

N -2.3913034 0.9095068 1.8562016

O 0.4728047 -0.6920746 1.5865008

C 0.9539508 0.4212218 1.5608929

O 0.2381180 1.5262416 1.6898672

C 2.4120418 0.6968442 1.4141148

C 3.2331811 -0.1186429 0.6291550

C 4.6015946 0.1249375 0.5186453

C 5.1330839 1.1997312 1.2321774

C 4.3319082 2.0183003 2.0099017

C 2.9691425 1.7768584 2.0896263

N 2.6718871 -1.2224355 -0.0800978

C 2.7224123 -2.5007113 0.3675305

C 2.0679955 -3.3190079 -0.5299644

C 1.5947021 -2.4972816 -1.5632336

C 1.9712880 -1.1966061 -1.2713421

C 1.7349256 0.0143479 -2.0268251

O 0.9958245 -0.2204282 -3.1288795

O 2.1372611 1.1241618 -1.7449287

C -4.7033609 1.3959069 1.0674975

H	6.1986341	1.3954925	1.1651340
H	2.3314039	2.4148639	2.6885834
H	4.7677141	2.8477559	2.5549410
H	3.2111741	-2.7296263	1.3018721
H	1.9466966	-4.3873236	-0.4375230
H	1.0394579	-2.7986273	-2.4378426
H	-5.3554363	2.2033594	0.7304477
H	-4.6583598	0.6373073	0.2805618
H	-5.1433792	0.9489605	1.9621848
H	0.9088036	0.6175072	-3.6057393
C	-2.9022382	-2.6185502	1.6668354
C	-3.3157842	1.9426980	1.3615222
H	-3.3862326	2.6909751	2.1558986
C	-3.0013013	-2.3432695	0.1843399
C	-4.2358519	-2.4547222	-0.4506606
C	-1.8919295	-1.9450715	-0.5602028
C	-4.3657445	-2.1767280	-1.8055817
H	-5.1061148	-2.7598410	0.1227525
C	-2.0191643	-1.6726602	-1.9161991
H	-0.9250563	-1.8302635	-0.0824587
C	-3.2543917	-1.7872509	-2.5425040
H	-5.3347200	-2.2667056	-2.2849064
H	-1.1491624	-1.3568094	-2.4813068
H	-3.3508708	-1.5709147	-3.6010686
C	-2.6972084	2.6023897	0.1500062
C	-2.2612531	1.8305190	-0.9237380

C -2.5699875 3.9851801 0.0850558

C -1.7092522 2.4311416 -2.0444892

H -2.3404899 0.7499764 -0.8767383

C -2.0186889 4.5909198 -1.0389983

H -2.8971043 4.5953668 0.9216188

C -1.5863879 3.8151550 -2.1066158

H -1.3717248 1.8173749 -2.8729081

H -1.9207549 5.6705757 -1.0761325

H -1.1519165 4.2856492 -2.9820107

H -0.7323937 1.2773019 1.7829428

H -3.7964435 -3.1682678 1.9699187

C -1.6790656 -3.4300550 2.0656678

H -1.6820197 -4.3819088 1.5311837

H -0.7603753 -2.8922277 1.8276389

H -1.6973710 -3.6313461 3.1384147

C 5.4925644 -0.6757297 -0.3893865

C 5.6622646 -0.0046578 -1.7520866

H 5.0953279 -1.6828695 -0.5258066

H 6.4708778 -0.7816221 0.0874571

H 6.3087044 -0.6039171 -2.3973588

H 4.6975894 0.1182732 -2.2488166

H 6.1112015 0.9862401 -1.6448019

1c_A_tpssh_TZVP

Energy= -1665.8803342499268

N -3.1625021 -1.0638958 2.3387139

C	-2.8419667	0.0194675	1.8962934
N	-2.4768007	1.1543911	1.5861597
O	0.2779534	-0.4801665	1.4060154
C	0.8519223	0.5883371	1.5390207
O	0.1995887	1.7238610	1.8145282
C	2.3296985	0.7727471	1.4393892
C	3.1505161	-0.1842867	0.8176770
C	4.5390510	-0.0287597	0.7612378
C	5.0947116	1.1178232	1.3400314
C	4.2985282	2.0823252	1.9448357
C	2.9197427	1.9142018	1.9880496
N	2.5761721	-1.3662610	0.2376581
C	2.4959707	-2.5608072	0.8890138
C	1.8205798	-3.4663209	0.0890185
C	1.4665543	-2.7914020	-1.0916165
C	1.9345225	-1.4859390	-0.9899155
C	1.8352891	-0.3960064	-1.9330397
O	1.1151895	-0.7486391	-3.0347614
O	2.3177206	0.7198582	-1.8071189
C	-4.4964668	1.4131731	0.1256842
H	6.1714690	1.2491350	1.3092866
H	2.2916958	2.6580472	2.4598028
H	4.7506161	2.9635849	2.3851350
H	2.9132933	-2.6660694	1.8778607
H	1.6076774	-4.4933738	0.3423589
H	0.9311255	-3.1892921	-1.9391007

H	-5.0791498	2.1475127	-0.4335784
H	-4.1434095	0.6441170	-0.5657518
H	-5.1470513	0.9469216	0.8698909
H	1.1290562	0.0076605	-3.6461220
C	-3.0572459	-2.3841781	1.6499309
C	-3.3076555	2.1045403	0.7975182
H	-3.6731077	2.8532006	1.5051659
C	-3.1523526	-2.2254752	0.1453345
C	-4.3698573	-2.4676258	-0.4956558
C	-2.0597850	-1.7921376	-0.6125216
C	-4.5005605	-2.2749627	-1.8696631
H	-5.2233445	-2.8015914	0.0864229
C	-2.1885740	-1.6024600	-1.9864882
H	-1.1097156	-1.5923863	-0.1311866
C	-3.4081339	-1.8408993	-2.6186367
H	-5.4528798	-2.4638778	-2.3533904
H	-1.3310390	-1.2712682	-2.5613930
H	-3.5060937	-1.6904758	-3.6883620
C	-2.4193961	2.7952643	-0.2186870
C	-1.5063367	2.0638452	-0.9821219
C	-2.5323876	4.1706798	-0.4269194
C	-0.7150314	2.6982119	-1.9345146
H	-1.4063780	0.9970700	-0.8147403
C	-1.7469757	4.8075761	-1.3872898
H	-3.2338018	4.7481809	0.1676355
C	-0.8345523	4.0722383	-2.1420546

H 0.0081248 2.1249753 -2.5024317

H -1.8404028 5.8777809 -1.5371510

H -0.2155159 4.5674063 -2.8821306

H -0.7885345 1.5219465 1.7941023

H -3.9304384 -2.9402060 1.9942369

C -1.7958245 -3.1019201 2.1305692

H -1.7662319 -4.1037338 1.6962774

H -0.8987330 -2.5592054 1.8330961

H -1.8104792 -3.1919398 3.2186924

C 5.4342083 -1.0144652 0.0485825

C 5.6947192 -0.6028278 -1.4098027

H 4.9934050 -2.0121752 0.0697908

H 6.3855439 -1.0691658 0.5844845

H 6.3445967 -1.3323393 -1.8994857

H 4.7583662 -0.5411273 -1.9672515

H 6.1797776 0.3756926 -1.4546764

1c_A_pbe_def2TZVP

Energy= -1663.79998065688

N -2.8694559 -1.4970250 2.4802517

C -2.6537117 -0.3596587 2.1005162

N -2.3559373 0.8177409 1.8589521

O 0.5506069 -0.7642127 1.6525715

C 1.0140461 0.3710761 1.6564029

O 0.2746009 1.4725505 1.8387138

C 2.4677170 0.6821660 1.4784983

C	3.2748294	-0.0693752	0.6029939
C	4.6422309	0.2143589	0.4452963
C	5.1881332	1.2543804	1.2143140
C	4.4028641	2.0066455	2.0857231
C	3.0411243	1.7329545	2.2051642
N	2.7020993	-1.1442963	-0.1587615
C	2.8549320	-2.4560023	0.1926423
C	2.2073483	-3.2571557	-0.7408569
C	1.6377812	-2.3960965	-1.6965716
C	1.9424733	-1.0824319	-1.3300697
C	1.5186287	0.1099113	-2.0439066
O	1.9151229	1.2799923	-1.4787261
O	0.8478674	0.0797287	-3.0762387
C	-4.6475919	1.2474212	0.9407485
H	6.2530336	1.4764922	1.1134694
H	2.4144812	2.3205186	2.8759636
H	4.8513920	2.8105350	2.6714199
H	3.4055708	-2.7175975	1.0913518
H	2.1544247	-4.3415070	-0.7169736
H	1.0547765	-2.6693787	-2.5705860
H	-5.3118812	2.0437355	0.5787276
H	-4.5231808	0.5064079	0.1364613
H	-5.1283051	0.7602250	1.8013992
H	1.5578938	2.0012498	-2.0388921
C	-2.8156034	-2.7497261	1.6847119
C	-3.2926205	1.8398192	1.3347823

H	-3.4403727	2.5638037	2.1523533
C	-2.9422408	-2.4767885	0.1970717
C	-4.1804753	-2.6579057	-0.4342512
C	-1.8607697	-1.9953561	-0.5565440
C	-4.3416018	-2.3638439	-1.7910245
H	-5.0288361	-3.0300156	0.1461818
C	-2.0199278	-1.7011421	-1.9132629
H	-0.8903165	-1.8356636	-0.0824029
C	-3.2598190	-1.8823420	-2.5337056
H	-5.3134027	-2.5095406	-2.2669771
H	-1.1721119	-1.3161549	-2.4840009
H	-3.3824770	-1.6481063	-3.5930317
C	-2.6448777	2.5736136	0.1719864
C	-1.9863370	1.8718363	-0.8459583
C	-2.7471376	3.9673833	0.0781692
C	-1.4458115	2.5482436	-1.9412389
H	-1.8921165	0.7863751	-0.7801073
C	-2.2060874	4.6486481	-1.0166098
H	-3.2523230	4.5255573	0.8708047
C	-1.5542161	3.9401922	-2.0304298
H	-0.9394350	1.9816145	-2.7260994
H	-2.2902827	5.7356580	-1.0742661
H	-1.1296771	4.4703150	-2.8851730
H	-0.7101921	1.1946223	1.9001890
H	-3.6994954	-3.3227574	2.0039950
C	-1.5631669	-3.5353177	2.0800389

H	-1.5589322	-4.5027200	1.5594155
H	-0.6518083	-2.9830873	1.8165473
H	-1.5595216	-3.7193440	3.1632368
C	5.5112594	-0.5076593	-0.5565596
C	5.5120333	0.1838659	-1.9304620
H	5.1830934	-1.5498408	-0.6733935
H	6.5395019	-0.5395058	-0.1653976
H	6.1684458	-0.3528278	-2.6301169
H	4.5012033	0.2101132	-2.3613780
H	5.8697692	1.2205872	-1.8480989

1c_A_pbe0_def2TZVP

Energy= -1663.9108616474082

N	-2.9274700	-1.3947941	2.4747371
C	-2.7017502	-0.2640272	2.1197783
N	-2.4088053	0.9028017	1.8864782
O	0.4562374	-0.6797114	1.5972168
C	0.9426695	0.4296413	1.5829034
O	0.2346040	1.5333878	1.7538019
C	2.3968857	0.7003055	1.4041579
C	3.2026696	-0.1287327	0.6183893
C	4.5694382	0.1081580	0.4847169
C	5.1160008	1.1879201	1.1771002
C	4.3305654	2.0208788	1.9540012
C	2.9690013	1.7861636	2.0560679
N	2.6315640	-1.2395105	-0.0687633

C	2.6663168	-2.5062196	0.4053737
C	2.0441640	-3.3439865	-0.4968482
C	1.6115975	-2.5443346	-1.5635569
C	1.9791631	-1.2388532	-1.2845759
C	1.7640572	-0.0436305	-2.0710377
O	1.0996614	-0.3098335	-3.2099896
O	2.1233800	1.0777358	-1.7812968
C	-4.7012131	1.3360853	1.0232489
H	6.1815651	1.3753323	1.0932481
H	2.3431346	2.4329716	2.6573335
H	4.7780925	2.8544595	2.4825236
H	3.1241374	-2.7165870	1.3593721
H	1.9167427	-4.4093529	-0.3844815
H	1.0863876	-2.8625514	-2.4501695
H	-5.3621199	2.1293947	0.6704567
H	-4.6053808	0.5876376	0.2316984
H	-5.1601481	0.8679390	1.8972560
H	1.0242040	0.5190313	-3.7043742
C	-2.8742413	-2.6210712	1.6630196
C	-3.3389179	1.9147456	1.3700741
H	-3.4563297	2.6547784	2.1671567
C	-2.9579218	-2.3183030	0.1853274
C	-4.2008998	-2.3349695	-0.4417646
C	-1.8330000	-1.9737115	-0.5600309
C	-4.3240859	-2.0137354	-1.7864776
H	-5.0836468	-2.6005812	0.1318435

C	-1.9537017	-1.6574409	-1.9064647
H	-0.8563739	-1.9372097	-0.0916896
C	-3.1974960	-1.6733914	-2.5229691
H	-5.3002345	-2.0308779	-2.2586303
H	-1.0699032	-1.3875131	-2.4731838
H	-3.2884928	-1.4216901	-3.5737914
C	-2.7107116	2.6046481	0.1812820
C	-2.0665957	1.8683736	-0.8074901
C	-2.8045129	3.9847858	0.0430342
C	-1.5240387	2.5007348	-1.9151036
H	-1.9818733	0.7922721	-0.7061105
C	-2.2647138	4.6209322	-1.0677359
H	-3.2987949	4.5690256	0.8132180
C	-1.6209965	3.8800903	-2.0499134
H	-1.0186459	1.9145239	-2.6743266
H	-2.3407351	5.6986408	-1.1614135
H	-1.1921863	4.3757253	-2.9137528
H	-0.7325820	1.2816024	1.8480693
H	-3.7691332	-3.1829122	1.9422205
C	-1.6545640	-3.4290618	2.0743040
H	-1.6384600	-4.3729841	1.5264572
H	-0.7342164	-2.8818784	1.8671715
H	-1.6957271	-3.6459487	3.1432141
C	5.4472301	-0.7148743	-0.4144769
C	5.6269896	-0.0673763	-1.7859482
H	5.0370546	-1.7188293	-0.5357295

H	6.4242017	-0.8275686	0.0635253
H	6.2749701	-0.6795245	-2.4169958
H	4.6663828	0.0511471	-2.2910447
H	6.0785895	0.9234927	-1.6925376

1c_A_b3lyp_TZVP

Energy = -1664.8230737522135

N	-2.9521153	-1.4274482	2.4834534
C	-2.7175317	-0.2937528	2.1323602
N	-2.4427508	0.8775677	1.8889204
O	0.3622703	-0.5827940	1.4251312
C	0.9059896	0.4886840	1.6136241
O	0.2363201	1.5613684	2.0470555
C	2.3634444	0.7459561	1.4050416
C	3.1540877	-0.1004781	0.6073764
C	4.5194273	0.1390357	0.4266388
C	5.0847822	1.2428154	1.0732137
C	4.3186616	2.0909317	1.8601236
C	2.9602285	1.8492444	2.0178109
N	2.5745334	-1.2475958	-0.0328882
C	2.5936939	-2.4964726	0.5086344
C	1.9072225	-3.3613270	-0.3241926
C	1.4435489	-2.6038772	-1.4134570
C	1.8578194	-1.2916502	-1.2233992
C	1.6316421	-0.1313601	-2.0555438
O	0.8720689	-0.4272614	-3.1435844

O	2.0473108	0.9957598	-1.8494289
C	-4.5400272	1.1229737	0.5353911
H	6.1444372	1.4351885	0.9490449
H	2.3574599	2.5083356	2.6261433
H	4.7774922	2.9401340	2.3511996
H	3.0841031	-2.6716043	1.4520693
H	1.7570824	-4.4150864	-0.1530290
H	0.8665918	-2.9519755	-2.2541416
H	-5.1819763	1.8619633	0.0541049
H	-4.1889751	0.4197712	-0.2227260
H	-5.1349199	0.5772666	1.2707140
H	0.7940026	0.3764167	-3.6826408
C	-2.8196878	-2.6728006	1.6756286
C	-3.3538034	1.8269529	1.2017652
H	-3.7283254	2.5048710	1.9722909
C	-3.0190393	-2.3897595	0.1976532
C	-4.2660588	-2.6259593	-0.3843829
C	-2.0017040	-1.8358093	-0.5844459
C	-4.5001165	-2.3080782	-1.7200230
H	-5.0637087	-3.0519682	0.2144811
C	-2.2330786	-1.5197195	-1.9200868
H	-1.0311053	-1.6365370	-0.1511495
C	-3.4825721	-1.7513215	-2.4911928
H	-5.4751060	-2.4929970	-2.1555359
H	-1.4330647	-1.0937329	-2.5126742
H	-3.6618948	-1.5000822	-3.5300934

C	-2.5600636	2.6419491	0.1966364
C	-1.5886900	2.0470555	-0.6103175
C	-2.8309985	4.0017685	0.0377222
C	-0.8933787	2.7992064	-1.5513405
H	-1.3660946	0.9946528	-0.4895540
C	-2.1450713	4.7555984	-0.9125005
H	-3.5796090	4.4770376	0.6626446
C	-1.1711776	4.1558592	-1.7079785
H	-0.1178169	2.3310600	-2.1439026
H	-2.3638137	5.8113280	-1.0228295
H	-0.6276735	4.7426609	-2.4391333
H	-0.7373934	1.3287943	2.0747456
H	-3.6398086	-3.3062343	2.0145398
C	-1.5018838	-3.3680750	2.0176111
H	-1.4499912	-4.3217614	1.4892424
H	-0.6473718	-2.7584422	1.7295550
H	-1.4491422	-3.5613668	3.0905477
C	5.3777631	-0.7052443	-0.4863807
C	5.4896009	-0.0987440	-1.8950575
H	4.9804796	-1.7173281	-0.5588445
H	6.3758563	-0.7869042	-0.0491309
H	6.1149074	-0.7274063	-2.5326189
H	4.5060848	-0.0052222	-2.3574185
H	5.9356651	0.8977544	-1.8543883

1c_A_blyp_TZVP

Energy= -1665.2122478054955

N	-2.9430683	-1.4385000	2.5210110
C	-2.7175476	-0.2986681	2.1444550
N	-2.4477023	0.8811118	1.8825825
O	0.3748587	-0.5998282	1.5030806
C	0.9131246	0.4982417	1.6295536
O	0.2244146	1.6063755	1.9944378
C	2.3778808	0.7569592	1.4186568
C	3.1723051	-0.0992321	0.6175072
C	4.5510983	0.1280939	0.4435469
C	5.1255756	1.2334496	1.0997688
C	4.3567660	2.0925093	1.8895841
C	2.9865630	1.8618957	2.0405475
N	2.5764593	-1.2426036	-0.0397784
C	2.5825954	-2.5093074	0.4914854
C	1.8622979	-3.3570365	-0.3477801
C	1.3919861	-2.5766887	-1.4267378
C	1.8323304	-1.2606600	-1.2307704
C	1.6244474	-0.0825018	-2.0504707
O	0.8290197	-0.3579055	-3.1484535
O	2.0756450	1.0452953	-1.8434162
C	-4.5468540	1.0876010	0.4843800
H	6.1931463	1.4187355	0.9808361
H	2.3832948	2.5277159	2.6518448
H	4.8235607	2.9408017	2.3881061
H	3.0836416	-2.7014466	1.4320978

H	1.6956756	-4.4154033	-0.1843779
H	0.7915247	-2.9081537	-2.2644934
H	-5.2019749	1.8185816	-0.0052768
H	-4.1686289	0.3917049	-0.2752547
H	-5.1419663	0.5228577	1.2138712
H	0.7676769	0.4658631	-3.6768780
C	-2.8091202	-2.6992116	1.7031856
C	-3.3774164	1.8221778	1.1725925
H	-3.7775076	2.4946679	1.9439384
C	-3.0115945	-2.4104451	0.2181059
C	-4.2658355	-2.6523514	-0.3695971
C	-1.9922983	-1.8377671	-0.5653303
C	-4.5041151	-2.3225254	-1.7112416
H	-5.0649215	-3.0902814	0.2296041
C	-2.2278133	-1.5097938	-1.9067441
H	-1.0184132	-1.6334000	-0.1282193
C	-3.4842610	-1.7473378	-2.4829674
H	-5.4834413	-2.5112429	-2.1505061
H	-1.4268036	-1.0692146	-2.4977922
H	-3.6674583	-1.4862279	-3.5250738
C	-2.5760947	2.6548890	0.1741209
C	-1.6106669	2.0600173	-0.6569255
C	-2.8320520	4.0303013	0.0441643
C	-0.9071340	2.8271988	-1.5926606
H	-1.3990497	0.9974458	-0.5570706
C	-2.1376466	4.7996981	-0.9014565

H	-3.5745868	4.5045149	0.6872966
C	-1.1699524	4.1996777	-1.7204939
H	-0.1373386	2.3593142	-2.2032046
H	-2.3440831	5.8662537	-0.9886779
H	-0.6200442	4.7973610	-2.4470519
H	-0.7589657	1.3544540	2.0379315
H	-3.6364520	-3.3306786	2.0485845
C	-1.4844214	-3.4044545	2.0489063
H	-1.4403673	-4.3649261	1.5194864
H	-0.6213338	-2.7991127	1.7560931
H	-1.4308833	-3.5972334	3.1280930
C	5.4086246	-0.7281392	-0.4737257
C	5.4865149	-0.1477998	-1.9086796
H	5.0215192	-1.7523732	-0.5179791
H	6.4218596	-0.7879104	-0.0526985
H	6.1109684	-0.7873512	-2.5467220
H	4.4881716	-0.0763202	-2.3561386
H	5.9233054	0.8600535	-1.8977081

1c_B_b3lyp_TZVP

Energy= -1664.8257562350877

C	3.0724533	0.2715773	-0.0126367
C	3.5172109	2.6428373	-1.3982958
C	1.9928275	0.9646380	-0.5862476
C	4.3872776	0.7414589	-0.1501852
C	4.5800307	1.9434971	-0.8347799

C	2.2292110	2.1453267	-1.2942655
H	5.5811044	2.3351657	-0.9518520
H	1.3924205	2.6678906	-1.7377111
H	3.6992257	3.5694153	-1.9281146
N	2.8482260	-0.9465673	0.7029699
C	2.9271891	-1.0529033	2.0705133
H	3.3431246	-0.2505812	2.6564978
C	2.1950844	-2.0678159	0.2126731
C	2.3666526	-2.2501765	2.4530122
H	2.3058202	-2.6239816	3.4628097
C	1.8880991	-2.8847005	1.2802583
H	1.3979559	-3.8424081	1.2094301
C	1.9434830	-2.2738953	-1.2232057
C	0.5768352	0.5042824	-0.5475899
O	2.7778734	-2.0485878	-2.0860893
O	0.2419812	-0.0659124	0.6549185
O	0.7327759	-2.7358162	-1.5302564
O	-0.2073940	0.6419304	-1.4476448
C	-0.9652746	-0.7420631	0.7725152
N	-1.1324637	-1.8178561	0.1044496
N	-1.7737910	-0.1679000	1.6825490
C	-1.5976981	1.1950298	2.2188147
H	-2.6817005	-0.5880674	1.8091362
C	-2.4229339	-2.5233408	0.1963425
H	0.0246431	-2.4444738	-0.8309327
C	-3.5723090	-1.6677474	-0.3258006

C	-4.7639146	-1.5641535	0.3928513
C	-3.4440767	-0.9661684	-1.5277583
C	-5.8115183	-0.7717673	-0.0768770
H	-4.8732689	-2.0976878	1.3314151
C	-4.4885141	-0.1799768	-2.0007478
H	-2.5090820	-1.0070853	-2.0718139
C	-5.6759099	-0.0780841	-1.2760878
H	-6.7267737	-0.6931119	0.4977508
H	-4.3715980	0.3681723	-2.9280002
H	-6.4845296	0.5443380	-1.6401857
C	-1.6965040	2.2509579	1.1297059
C	-0.7313500	3.2524145	1.0366054
C	-2.7402973	2.2256287	0.2036167
C	-0.8064663	4.2175181	0.0341842
H	0.0949668	3.2687257	1.7391791
C	-2.8170938	3.1847736	-0.7988452
H	-3.4786105	1.4336439	0.2412309
C	-1.8498490	4.1852159	-0.8866943
H	-0.0434501	4.9839435	-0.0344355
H	-3.6245758	3.1441428	-1.5204210
H	-1.9045968	4.9279256	-1.6738767
H	-2.6271414	-2.7652361	1.2471561
C	-2.3144053	-3.8425613	-0.5718429
H	-1.5040337	-4.4525835	-0.1679839
H	-3.2469995	-4.4023225	-0.4913800
H	-2.1139872	-3.6526550	-1.6283077

C	-2.6366108	1.4042981	3.3209370
H	-2.5055681	2.3901446	3.7669101
H	-3.6493837	1.3500359	2.9128388
H	-2.5285864	0.6491791	4.1021006
H	-0.6021144	1.2459547	2.6601864
C	5.5469803	-0.0605892	0.4065774
C	6.9294399	0.3141288	-0.1276960
H	5.3537681	-1.1169928	0.2036698
H	5.5494678	0.0307482	1.4972303
H	7.6757677	-0.3739655	0.2732845
H	6.9659995	0.2529357	-1.2182672
H	7.2227748	1.3240805	0.1658788

1c_B_pbe0_TZVP

Energy= -1663.85289637679

C	3.0570461	0.2625002	-0.0010954
C	3.5457847	2.5760393	-1.4526522
C	1.9944842	0.9551524	-0.5951285
C	4.3766342	0.7007328	-0.1521573
C	4.5924121	1.8742058	-0.8707555
C	2.2516248	2.1067416	-1.3355602
H	5.6041301	2.2381588	-1.0015069
H	1.4230392	2.6288256	-1.7984002
H	3.7457712	3.4829036	-2.0113641
N	2.8116034	-0.9261670	0.7380608
C	2.8937469	-1.0107658	2.0997904

H	3.3207556	-0.2028579	2.6730512
C	2.1521945	-2.0393225	0.2646869
C	2.3231045	-2.1942341	2.4997799
H	2.2608262	-2.5529122	3.5161720
C	1.8386324	-2.8390903	1.3392199
H	1.3424625	-3.7961493	1.2808308
C	1.9247737	-2.2662997	-1.1723704
C	0.5767818	0.5166873	-0.5571436
O	2.7889827	-2.0811613	-2.0101686
O	0.2204923	-0.0199190	0.6445988
O	0.7215234	-2.6971775	-1.4944081
O	-0.1920836	0.6469040	-1.4667613
C	-0.9752306	-0.6949073	0.7396034
N	-1.1246201	-1.7760305	0.0774736
N	-1.8096874	-0.1303744	1.6200202
C	-1.6394505	1.2059320	2.1910353
H	-2.7149259	-0.5623579	1.7254084
C	-2.3957391	-2.4874174	0.1721096
H	0.0002813	-2.3815936	-0.8010398
C	-3.5559925	-1.6664065	-0.3612929
C	-4.7950255	-1.7132316	0.2716620
C	-3.4055411	-0.8718326	-1.4962445
C	-5.8699835	-0.9817276	-0.2213749
H	-4.9199016	-2.3225901	1.1622339
C	-4.4782524	-0.1444992	-1.9917294
H	-2.4322495	-0.7938488	-1.9677259

C	-5.7143373	-0.1967902	-1.3566180
H	-6.8275800	-1.0216217	0.2862874
H	-4.3455883	0.4783334	-2.8697920
H	-6.5498511	0.3786729	-1.7400951
C	-1.6813886	2.2849637	1.1309332
C	-0.7069503	3.2760825	1.1046279
C	-2.6881051	2.2977990	0.1698392
C	-0.7361580	4.2685455	0.1320516
H	0.0911330	3.2636434	1.8412137
C	-2.7185297	3.2842542	-0.8033788
H	-3.4365957	1.5120598	0.1585399
C	-1.7420407	4.2739819	-0.8249954
H	0.0354113	5.0307944	0.1158724
H	-3.5007230	3.2751797	-1.5551210
H	-1.7609386	5.0413514	-1.5913045
H	-2.6024448	-2.7215346	1.2262959
C	-2.2759212	-3.8056539	-0.5814516
H	-1.4618079	-4.4074937	-0.1713294
H	-3.2047793	-4.3721001	-0.5006402
H	-2.0746859	-3.6195390	-1.6392263
C	-2.7191237	1.4038135	3.2436029
H	-2.6014606	2.3806809	3.7133276
H	-3.7126804	1.3663664	2.7872769
H	-2.6540406	0.6336340	4.0154961
H	-0.6615387	1.2383053	2.6766567
C	5.5141627	-0.1075566	0.4167279

C	6.8822375	0.1891363	-0.1743069
H	5.2765463	-1.1659827	0.2761627
H	5.5494309	0.0409078	1.5016357
H	7.6192726	-0.5028220	0.2380406
H	6.8802693	0.0705464	-1.2612368
H	7.2196397	1.2022533	0.0559144

1c_B_tpssh_TZVP

Energy= -1665.88811724830

C	3.0688210	0.2779923	-0.0186402
C	3.5373091	2.6343273	-1.4213540
C	1.9986686	0.9620187	-0.6200589
C	4.3846195	0.7500148	-0.1284225
C	4.5895457	1.9456625	-0.8224061
C	2.2471439	2.1341582	-1.3400306
H	5.5944551	2.3373737	-0.9192950
H	1.4160843	2.6482786	-1.8065731
H	3.7288999	3.5553069	-1.9592455
N	2.8313773	-0.9415817	0.6889104
C	2.8971878	-1.0622321	2.0566306
H	3.3234097	-0.2710634	2.6519044
C	2.1548103	-2.0418491	0.1803411
C	2.3061328	-2.2522180	2.4213667
H	2.2309147	-2.6359366	3.4274927
C	1.8227047	-2.8637003	1.2396302
H	1.3167642	-3.8131731	1.1553712

C	1.9299623	-2.2272590	-1.2614642
C	0.5846145	0.5048053	-0.5886298
O	2.7819623	-1.9933846	-2.1105606
O	0.2430197	-0.0345928	0.6328468
O	0.7223409	-2.6863544	-1.5920219
O	-0.1981502	0.6270535	-1.4963158
C	-0.9531917	-0.7282176	0.7503062
N	-1.1140068	-1.8052963	0.0747871
N	-1.7556417	-0.1777812	1.6804116
C	-1.5832872	1.1861207	2.2205542
H	-2.6679084	-0.5991480	1.7932636
C	-2.3991594	-2.5174619	0.2058069
H	0.0123255	-2.4021768	-0.8775139
C	-3.5525584	-1.6678945	-0.3113441
C	-4.7332512	-1.5487992	0.4247309
C	-3.4365392	-0.9840798	-1.5258441
C	-5.7843477	-0.7577183	-0.0415742
H	-4.8294541	-2.0702673	1.3727314
C	-4.4858849	-0.2009034	-1.9957907
H	-2.5078787	-1.0388357	-2.0817733
C	-5.6630102	-0.0838443	-1.2547422
H	-6.6913730	-0.6653970	0.5456865
H	-4.3808832	0.3328966	-2.9339762
H	-6.4747672	0.5373788	-1.6165990
C	-1.6868420	2.2394020	1.1313741
C	-0.7236973	3.2439383	1.0366707

C	-2.7366154	2.2114999	0.2110992
C	-0.8083986	4.2126305	0.0377735
H	0.1061388	3.2588569	1.7366718
C	-2.8229386	3.1759018	-0.7869645
H	-3.4715412	1.4146634	0.2517850
C	-1.8593493	4.1807069	-0.8759735
H	-0.0476998	4.9823593	-0.0333927
H	-3.6363218	3.1367121	-1.5032472
H	-1.9226606	4.9275779	-1.6596489
H	-2.5831588	-2.7405189	1.2655545
C	-2.2959084	-3.8463761	-0.5449151
H	-1.4728825	-4.4428871	-0.1446180
H	-3.2240594	-4.4101942	-0.4375618
H	-2.1159450	-3.6687016	-1.6077159
C	-2.6308711	1.3854808	3.3161448
H	-2.5108225	2.3739033	3.7611362
H	-3.6394808	1.3202391	2.8972486
H	-2.5192520	0.6298667	4.0972542
H	-0.5871833	1.2383772	2.6632629
C	5.5312699	-0.0456157	0.4602114
C	6.9134381	0.2981630	-0.0948469
H	5.3228583	-1.1064558	0.2943642
H	5.5369450	0.0890763	1.5474484
H	7.6558870	-0.3827559	0.3269813
H	6.9389778	0.1976773	-1.1834544
H	7.2146279	1.3164705	0.1621319

1c_B_pbe_def2TZVP

Energy= -1663.8067665445899

C	3.0882742	0.2651179	-0.0086790
C	3.5497912	2.6317253	-1.4217129
C	2.0090156	0.9595797	-0.5967073
C	4.4115775	0.7315075	-0.1466856
C	4.6111281	1.9304852	-0.8449079
C	2.2557312	2.1368053	-1.3196028
H	5.6239374	2.3163765	-0.9627770
H	1.4140546	2.6590283	-1.7751036
H	3.7385390	3.5597735	-1.9627910
N	2.8575729	-0.9483458	0.7102102
C	2.9793211	-1.0664775	2.0775848
H	3.4126427	-0.2614037	2.6622671
C	2.1854409	-2.0631479	0.2235903
C	2.4184418	-2.2702208	2.4667261
H	2.3841581	-2.6536516	3.4826323
C	1.9040064	-2.8932151	1.3001733
H	1.4108954	-3.8588359	1.2326153
C	1.9218776	-2.2628056	-1.2169580
C	0.5886254	0.5139122	-0.5608944
O	2.7749276	-2.0549425	-2.0827221
O	0.2435350	-0.0455028	0.6591037
O	0.7007356	-2.6991834	-1.5189922
O	-0.2010627	0.6555085	-1.4670333

C	-0.9669990	-0.7203110	0.7575652
N	-1.1214845	-1.8164772	0.0939277
N	-1.8024387	-0.1494291	1.6465141
C	-1.6394606	1.2031368	2.2087086
H	-2.7121827	-0.5893153	1.7624570
C	-2.4059090	-2.5293607	0.1935205
H	-0.0275192	-2.3823385	-0.7813068
C	-3.5639607	-1.6866803	-0.3318876
C	-4.7514434	-1.5717937	0.4030328
C	-3.4540956	-1.0069913	-1.5543257
C	-5.8123281	-0.7910484	-0.0703026
H	-4.8453022	-2.0936396	1.3595985
C	-4.5118861	-0.2312254	-2.0303687
H	-2.5213850	-1.0617101	-2.1184095
C	-5.6944296	-0.1190268	-1.2895070
H	-6.7270262	-0.7037272	0.5190131
H	-4.4097142	0.3003187	-2.9785234
H	-6.5164381	0.4971174	-1.6583176
C	-1.7267996	2.2798049	1.1370334
C	-0.7446704	3.2735820	1.0586421
C	-2.7791367	2.2880794	0.2119748
C	-0.8105148	4.2624336	0.0723314
H	0.0895458	3.2631533	1.7651263
C	-2.8471224	3.2721309	-0.7739943
H	-3.5376375	1.5023498	0.2392513
C	-1.8623772	4.2633692	-0.8471746

H	-0.0310221	5.0246519	0.0148339
H	-3.6663731	3.2589194	-1.4959260
H	-1.9105981	5.0278193	-1.6249248
H	-2.6089426	-2.7602266	1.2572804
C	-2.2963921	-3.8585503	-0.5595183
H	-1.4754677	-4.4651618	-0.1515734
H	-3.2326975	-4.4236918	-0.4643777
H	-2.1038796	-3.6817038	-1.6274389
C	-2.6931152	1.3901997	3.3013107
H	-2.5757909	2.3784407	3.7635508
H	-3.7080511	1.3323983	2.8787106
H	-2.5873854	0.6221172	4.0806050
H	-0.6404362	1.2532850	2.6668989
C	5.5686349	-0.0705553	0.4107137
C	6.9474767	0.2855681	-0.1428869
H	5.3594509	-1.1367488	0.2275004
H	5.5832900	0.0339014	1.5089361
H	7.7000655	-0.4033254	0.2648237
H	6.9740866	0.2048230	-1.2399773
H	7.2529921	1.3055715	0.1309149

1c_B_pbe0_def2TZVP

Energy= -1663.92374053728

C	3.0629579	0.2622778	-0.0079263
C	3.5215498	2.6119956	-1.4101020
C	1.9936917	0.9526915	-0.5898437

C	4.3759196	0.7240396	-0.1449757
C	4.5755928	1.9144356	-0.8380437
C	2.2353417	2.1213030	-1.3067751
H	5.5815823	2.2975234	-0.9550841
H	1.3992256	2.6397713	-1.7592815
H	3.7101067	3.5329521	-1.9487303
N	2.8322918	-0.9432887	0.7048556
C	2.9389506	-1.0592659	2.0609215
H	3.3612016	-0.2584728	2.6470028
C	2.1808015	-2.0524952	0.2172938
C	2.3896570	-2.2576636	2.4436670
H	2.3471914	-2.6385399	3.4526283
C	1.8950652	-2.8809740	1.2767446
H	1.4112030	-3.8427906	1.2022670
C	1.9275045	-2.2539514	-1.2187551
C	0.5792765	0.5052967	-0.5483132
O	2.7725638	-2.0477177	-2.0688958
O	0.2394558	-0.0489800	0.6471327
O	0.7226558	-2.6892639	-1.5234829
O	-0.1991100	0.6452846	-1.4465304
C	-0.9558666	-0.7177220	0.7509764
N	-1.1124440	-1.8012397	0.0957363
N	-1.7823103	-0.1478206	1.6342521
C	-1.6139518	1.1932351	2.1866583
H	-2.6856330	-0.5789686	1.7498140
C	-2.3872878	-2.5023641	0.2063033

H	0.0159975	-2.3970694	-0.8113070
C	-3.5398684	-1.6663743	-0.3162972
C	-4.7231844	-1.5606757	0.4069699
C	-3.4268322	-0.9885180	-1.5281056
C	-5.7791608	-0.7912607	-0.0687029
H	-4.8188558	-2.0797197	1.3564317
C	-4.4798222	-0.2256696	-2.0075941
H	-2.4958936	-1.0371519	-2.0813931
C	-5.6596026	-0.1230098	-1.2787418
H	-6.6919925	-0.7109296	0.5109452
H	-4.3768565	0.3025645	-2.9490751
H	-6.4785652	0.4824021	-1.6502845
C	-1.7039814	2.2628121	1.1209709
C	-0.7398782	3.2603716	1.0500431
C	-2.7482915	2.2627212	0.2016606
C	-0.8170776	4.2490384	0.0771683
H	0.0874602	3.2568078	1.7532572
C	-2.8288856	3.2473047	-0.7694469
H	-3.4913829	1.4719281	0.2241488
C	-1.8630261	4.2447181	-0.8342858
H	-0.0529804	5.0168226	0.0258706
H	-3.6434161	3.2317808	-1.4855134
H	-1.9222549	5.0110060	-1.5991474
H	-2.5864486	-2.7290734	1.2636891
C	-2.2888964	-3.8243924	-0.5407164
H	-1.4730666	-4.4295862	-0.1393595

H	-3.2207530	-4.3830874	-0.4430323
H	-2.1017956	-3.6483236	-1.6026369
C	-2.6630821	1.3872375	3.2690840
H	-2.5431847	2.3688997	3.7277849
H	-3.6693670	1.3341431	2.8434908
H	-2.5652818	0.6232144	4.0432891
H	-0.6228917	1.2458191	2.6430280
C	5.5237652	-0.0769616	0.4109356
C	6.8953674	0.2864507	-0.1303368
H	5.3200327	-1.1342370	0.2161267
H	5.5306986	0.0179947	1.5021011
H	7.6429657	-0.4011908	0.2694719
H	6.9247952	0.2184535	-1.2210679
H	7.1946060	1.2976725	0.1544339

1c_B_blyp_TZVP

Energy= -1665.20959758674

C	3.0965750	0.2775944	0.0112754
C	3.5105609	2.7049166	-1.3237279
C	1.9967200	0.9805244	-0.5386840
C	4.4160239	0.7657838	-0.1264728
C	4.5919415	1.9965095	-0.7840220
C	2.2196332	2.1895462	-1.2231453
H	5.5935331	2.4035971	-0.8994609
H	1.3696156	2.7194966	-1.6465805
H	3.6803938	3.6526066	-1.8321919

N	2.8863188	-0.9714333	0.6989559
C	2.9930791	-1.1217778	2.0731546
H	3.4240763	-0.3338936	2.6774570
C	2.2111983	-2.0856768	0.1830438
C	2.4301564	-2.3379603	2.4306172
H	2.3900670	-2.7457258	3.4345653
C	1.9219620	-2.9375743	1.2440242
H	1.4240267	-3.8959122	1.1535840
C	1.9199074	-2.2443043	-1.2564703
C	0.5800059	0.5027512	-0.5009554
O	2.7346998	-1.9798204	-2.1476539
O	0.2568402	-0.0818123	0.7262536
O	0.6877263	-2.7109552	-1.5461239
O	-0.2208726	0.6333688	-1.4032759
C	-0.9662506	-0.7652580	0.8436798
N	-1.1359017	-1.8453494	0.1590593
N	-1.7745877	-0.1904913	1.7699933
C	-1.6252228	1.2077480	2.2691210
H	-2.6876916	-0.6162708	1.9007758
C	-2.4517268	-2.5410072	0.2118689
H	-0.0128192	-2.4273255	-0.8024131
C	-3.5751004	-1.6479029	-0.3307123
C	-4.7662868	-1.4741088	0.3935137
C	-3.4179672	-0.9718633	-1.5548570
C	-5.7842984	-0.6373643	-0.0918486
H	-4.8978205	-1.9870428	1.3475234

C	-4.4338990	-0.1436784	-2.0445609
H	-2.4814411	-1.0633295	-2.1016442
C	-5.6205296	0.0287280	-1.3140022
H	-6.6966661	-0.5025903	0.4883004
H	-4.2932323	0.3848180	-2.9867130
H	-6.4048014	0.6852001	-1.6892943
C	-1.7408983	2.2275472	1.1349741
C	-0.7585633	3.2168581	0.9716487
C	-2.8098148	2.1694585	0.2251559
C	-0.8415050	4.1359906	-0.0847475
H	0.0886715	3.2550485	1.6571135
C	-2.8937481	3.0816116	-0.8316675
H	-3.5602863	1.3863597	0.3174940
C	-1.9094524	4.0697433	-0.9899651
H	-0.0649135	4.8901377	-0.2090633
H	-3.7186014	3.0105960	-1.5398310
H	-1.9697947	4.7747776	-1.8186120
H	-2.6854601	-2.7942593	1.2600001
C	-2.3419287	-3.8623950	-0.5748849
H	-1.5471307	-4.4927026	-0.1553688
H	-3.2909303	-4.4094302	-0.5217074
H	-2.1115468	-3.6655948	-1.6300001
C	-2.6740223	1.4334477	3.3735314
H	-2.5591066	2.4416363	3.7876984
H	-3.6925136	1.3472496	2.9694731
H	-2.5492317	0.7035726	4.1840794

H	-0.6246743	1.2878385	2.7093788
C	5.5963297	-0.0428817	0.4030626
C	6.9778484	0.3285136	-0.1675620
H	5.3951252	-1.1051893	0.2068489
H	5.6264163	0.0501606	1.4996679
H	7.7333896	-0.3688614	0.2167471
H	6.9880483	0.2689032	-1.2647906
H	7.2866440	1.3413225	0.1227403

TS_1c_1_pbe_def2TZVP

Energy= -1663.78143916969

C	3.9441246	-0.5269434	0.3844879
C	6.0775307	-0.0021350	-1.3237640
C	3.7450150	0.2856154	-0.7447032
C	5.2040154	-1.0663966	0.6926017
C	6.2591877	-0.7980770	-0.1933226
C	4.8258269	0.5536814	-1.5914789
H	7.2471915	-1.2105020	0.0249313
H	4.6755518	1.1970655	-2.4599901
H	6.9177850	0.1989706	-1.9908906
N	2.8535631	-0.7031468	1.3068983
C	2.5484322	0.2190799	2.2698605
H	3.1316269	1.1310483	2.3554250
C	1.9722347	-1.7723518	1.4171119
C	1.4703100	-0.2460236	3.0166436
H	1.0103853	0.2713935	3.8534717

C	1.1059827	-1.4960764	2.4800614
H	0.2976292	-2.1422558	2.8078107
C	1.9906213	-2.9120364	0.5168395
C	2.3905525	0.8659773	-1.0708332
O	2.7759743	-3.1004041	-0.4059175
O	2.3096981	2.1205794	-1.2589659
O	0.9941168	-3.7998253	0.8160104
O	1.4213743	0.0456494	-1.1544803
N	-0.2939540	2.0760575	-1.0169493
C	-0.5769598	0.8305922	-1.1719919
N	-1.3093222	-0.1128604	-1.3446092
C	-1.2254739	3.0343448	-0.3499511
C	-1.2948519	-1.4690299	-0.7923014
C	-2.0520604	2.3626772	0.7292089
C	-1.4517289	1.5411350	1.6937162
C	-3.4341619	2.5767509	0.7836669
C	-2.2229563	0.9359417	2.6861234
H	-0.3773009	1.3452866	1.6569049
C	-4.2055726	1.9835454	1.7868223
H	-3.9120433	3.2041254	0.0270493
C	-3.6023676	1.1575799	2.7377678
H	-1.7463572	0.2781260	3.4152394
H	-5.2840144	2.1508035	1.8111849
H	-4.2068615	0.6722258	3.5056964
C	-2.5699130	-1.7366015	-0.0117467
C	-3.7847429	-1.1191290	-0.3287520

C	-2.5283802	-2.6608762	1.0414396
C	-4.9406747	-1.4195281	0.3960674
H	-3.8215625	-0.3804939	-1.1317384
C	-3.6838853	-2.9643739	1.7640917
H	-1.5810187	-3.1423283	1.2968239
C	-4.8951878	-2.3429403	1.4435182
H	-5.8784030	-0.9181223	0.1488944
H	-3.6358022	-3.6811047	2.5864548
H	-5.7971055	-2.5707401	2.0148482
C	-0.3844606	4.1917881	0.1906512
H	0.3031075	3.8417231	0.9737416
H	0.2002291	4.6596772	-0.6141596
H	-1.0483716	4.9504768	0.6244566
H	-1.9064732	3.4152147	-1.1279038
H	0.7437522	2.3186357	-1.1890762
C	-1.0944581	-2.4723259	-1.9329495
H	-1.9138370	-2.3983812	-2.6618506
H	-0.1390200	-2.2824005	-2.4408992
H	-1.0800327	-3.4911260	-1.5230508
H	-0.4420266	-1.5390666	-0.1013384
H	1.0812647	-4.5417387	0.1816338
C	5.4517497	-1.9134896	1.9182680
C	5.6700212	-3.3975387	1.5827916
H	4.6139192	-1.8148866	2.6223580
H	6.3447734	-1.5263132	2.4349447
H	5.8538134	-3.9755062	2.4999296

H	4.7910094	-3.8174465	1.0751216
H	6.5370875	-3.5286364	0.9190524

TS_1c_1_pbe0_TZVP

Energy= -1663.81872197804

C	3.9248715	-0.5220454	0.3820969
C	6.0494028	-0.0110709	-1.3129973
C	3.7350704	0.2910198	-0.7357018
C	5.1715531	-1.0681262	0.6855689
C	6.2229418	-0.8087906	-0.1934269
C	4.8095271	0.5521843	-1.5766911
H	7.2009671	-1.2276698	0.0217691
H	4.6652100	1.1971124	-2.4359829
H	6.8858421	0.1847214	-1.9749205
N	2.8423531	-0.6924099	1.3003383
C	2.5418972	0.2229799	2.2564066
H	3.1272170	1.1250651	2.3480043
C	1.9603668	-1.7463651	1.4025560
C	1.4648482	-0.2314598	2.9910861
H	1.0064786	0.2832591	3.8216302
C	1.0962032	-1.4725396	2.4510022
H	0.2881612	-2.1117116	2.7711113
C	1.9720860	-2.8820385	0.5045696
C	2.3951163	0.8888676	-1.0632884
O	2.7553647	-3.0710141	-0.4014634
O	2.3440523	2.1128284	-1.3260501

O	0.9803785	-3.7488060	0.7899121
O	1.4077737	0.1019502	-1.0736834
N	-0.2756077	2.0892497	-1.0029072
C	-0.5212797	0.8396343	-1.1513259
N	-1.2410732	-0.0968791	-1.3111506
C	-1.2378342	3.0189261	-0.3709464
C	-1.2324789	-1.4521508	-0.7865722
C	-2.0448620	2.3430349	0.7107460
C	-1.4323104	1.5404859	1.6703594
C	-3.4190084	2.5453251	0.7735844
C	-2.1861487	0.9383275	2.6660872
H	-0.3629455	1.3576904	1.6288142
C	-4.1723955	1.9567550	1.7813707
H	-3.9059866	3.1591606	0.0219838
C	-3.5583310	1.1478361	2.7270933
H	-1.7016658	0.2945376	3.3918085
H	-5.2448330	2.1150493	1.8141851
H	-4.1485155	0.6672353	3.4991758
C	-2.5038547	-1.7222239	-0.0167350
C	-3.7052907	-1.0970276	-0.3290957
C	-2.4709000	-2.6559869	1.0160411
C	-4.8590723	-1.4005234	0.3813445
H	-3.7341712	-0.3519045	-1.1163640
C	-3.6241450	-2.9610403	1.7251228
H	-1.5329356	-3.1419449	1.2666396
C	-4.8233218	-2.3329967	1.4093264

H	-5.7875516	-0.8954302	0.1378842
H	-3.5844891	-3.6849258	2.5322346
H	-5.7234840	-2.5632130	1.9689888
C	-0.4425675	4.2019355	0.1584795
H	0.2448377	3.8807276	0.9443228
H	0.1295628	4.6757896	-0.6425122
H	-1.1274591	4.9399074	0.5773303
H	-1.9205136	3.3646551	-1.1520374
H	0.7172498	2.3697784	-1.1972362
C	-1.0400144	-2.4303867	-1.9368850
H	-1.8553040	-2.3395238	-2.6579039
H	-0.0907577	-2.2391564	-2.4408930
H	-1.0321553	-3.4496793	-1.5470295
H	-0.3866775	-1.5344292	-0.1014960
H	1.0534168	-4.4936444	0.1755881
C	5.4093685	-1.9202227	1.9012800
C	5.6365542	-3.3898163	1.5517445
H	4.5677565	-1.8329495	2.5906563
H	6.2881231	-1.5350431	2.4278627
H	5.8059767	-3.9765289	2.4576970
H	4.7724604	-3.8017212	1.0272427
H	6.5092432	-3.5082713	0.9047704

TS_1c_1_b3lyp_TZVP

Energy= -1664.8009254832275

C	3.9286200	-0.5275367	0.3845725

C	6.0661665	-0.0805176	-1.3243187
C	3.7509448	0.2735695	-0.7489252
C	5.1697510	-1.0910988	0.6967685
C	6.2280759	-0.8629594	-0.1880140
C	4.8330804	0.5010497	-1.5966678
H	7.1977650	-1.2948910	0.0327036
H	4.7003249	1.1327768	-2.4658383
H	6.9036749	0.0887421	-1.9904728
N	2.8351286	-0.6839588	1.3048868
C	2.5350518	0.2361833	2.2651441
H	3.1228719	1.1344107	2.3599149
C	1.9409964	-1.7391379	1.4063888
C	1.4505278	-0.2145725	2.9974315
H	0.9905859	0.3028564	3.8236369
C	1.0747031	-1.4564011	2.4559845
H	0.2602785	-2.0864772	2.7723699
C	1.9441868	-2.8720869	0.5033900
C	2.4218752	0.9034407	-1.0882886
O	2.7223212	-3.0631563	-0.4123852
O	2.3995203	2.1160402	-1.4027344
O	0.9418926	-3.7446581	0.7914847
O	1.4058678	0.1352857	-1.0518265
N	-0.2791587	2.1400161	-0.9885036
C	-0.4822465	0.8748736	-1.1454440
N	-1.2322306	-0.0473285	-1.2858435
C	-1.2847548	3.0441642	-0.3559470

C	-1.2056806	-1.4195284	-0.7742577
C	-2.0642321	2.3417506	0.7385447
C	-1.4184721	1.5622939	1.7006315
C	-3.4485658	2.4917710	0.8055036
C	-2.1480355	0.9301353	2.7005099
H	-0.3456684	1.4172225	1.6545486
C	-4.1783074	1.8743746	1.8182468
H	-3.9619221	3.0823266	0.0546376
C	-3.5304573	1.0876708	2.7651746
H	-1.6380778	0.3041600	3.4223864
H	-5.2551060	1.9893487	1.8515482
H	-4.1000817	0.5835795	3.5360624
C	-2.4741836	-1.7145555	0.0052511
C	-3.6900727	-1.1021112	-0.2925252
C	-2.4221319	-2.6597749	1.0322744
C	-4.8374246	-1.4280889	0.4251414
H	-3.7360579	-0.3494581	-1.0693954
C	-3.5690632	-2.9877412	1.7485827
H	-1.4787243	-3.1369002	1.2710067
C	-4.7821684	-2.3716641	1.4468270
H	-5.7727365	-0.9321797	0.1941875
H	-3.5136502	-3.7182445	2.5474264
H	-5.6742186	-2.6186555	2.0103224
C	-0.5324159	4.2720330	0.1580352
H	0.1694556	3.9904686	0.9451617
H	0.0159221	4.7589513	-0.6512017

H	-1.2464626	4.9858907	0.5688517
H	-1.9797680	3.3519883	-1.1395620
H	0.6779807	2.4669304	-1.2081980
C	-1.0039392	-2.3847392	-1.9450908
H	-1.8222625	-2.2937336	-2.6617248
H	-0.0595101	-2.1728164	-2.4483992
H	-0.9806870	-3.4085151	-1.5698902
H	-0.3558157	-1.4992990	-0.0988026
H	1.0042888	-4.4887701	0.1708105
C	5.3957302	-1.9365953	1.9287700
C	5.5465019	-3.4312091	1.6028697
H	4.5772613	-1.7996086	2.6355911
H	6.3037353	-1.5865404	2.4277102
H	5.7092715	-4.0053824	2.5176664
H	4.6534494	-3.8124500	1.1065063
H	6.3975210	-3.6016603	0.9396168

TS_1c_1_pbe0_def2TZVP

Energy= -1663.8884408708675

C	3.9368000	-0.5515620	0.3508183
C	6.0752203	0.0790410	-1.2850415
C	3.7474424	0.3054697	-0.7325264
C	5.1905800	-1.0820530	0.6503794
C	6.2492970	-0.7595535	-0.1967322
C	4.8280153	0.6258045	-1.5431480
H	7.2330918	-1.1638615	0.0186455

H	4.6802257	1.3040051	-2.3753843
H	6.9166230	0.3214421	-1.9242612
N	2.8434040	-0.7974971	1.2360731
C	2.5056162	0.0531660	2.2354007
H	3.0619703	0.9660108	2.3825145
C	1.9942124	-1.8797658	1.2700602
C	1.4351399	-0.4710656	2.9315396
H	0.9525343	-0.0152522	3.7819672
C	1.1109807	-1.6904960	2.3207139
H	0.3182869	-2.3688782	2.5938452
C	2.0464824	-2.9619168	0.3089875
C	2.4036993	0.8946409	-1.0560218
O	2.8422556	-3.0758770	-0.5972784
O	2.3420718	2.1291443	-1.2624017
O	1.0792637	-3.8692218	0.5330196
O	1.4325479	0.0959813	-1.1214930
N	-0.2693899	2.0674079	-0.9818476
C	-0.5232858	0.8263769	-1.1623839
N	-1.2267764	-0.1155502	-1.3455776
C	-1.2120866	2.9758701	-0.2972130
C	-1.2070866	-1.4856240	-0.8693523
C	-2.0197639	2.2581238	0.7549164
C	-1.4096852	1.4019228	1.6672416
C	-3.3905175	2.4691135	0.8374479
C	-2.1614542	0.7584145	2.6368945
H	-0.3425297	1.2121245	1.6088345

C	-4.1423383	1.8383934	1.8195739
H	-3.8758104	3.1250562	0.1215891
C	-3.5302634	0.9772157	2.7181993
H	-1.6779597	0.0742493	3.3247299
H	-5.2124181	2.0058849	1.8684738
H	-4.1194654	0.4642721	3.4694135
C	-2.4747258	-1.8011511	-0.1121370
C	-3.6757125	-1.1524273	-0.3682362
C	-2.4373789	-2.8041937	0.8525014
C	-4.8239710	-1.5012788	0.3291739
H	-3.7101503	-0.3545634	-1.1011485
C	-3.5841899	-3.1543102	1.5490050
H	-1.5001101	-3.3109748	1.0598047
C	-4.7831644	-2.5023537	1.2888279
H	-5.7523759	-0.9780575	0.1291642
H	-3.5396499	-3.9325947	2.3030096
H	-5.6790091	-2.7679461	1.8390223
C	-0.3961374	4.1181401	0.2853422
H	0.2951851	3.7454774	1.0445828
H	0.1751119	4.6248659	-0.4956642
H	-1.0655402	4.8419346	0.7507180
H	-1.8957897	3.3704632	-1.0543217
H	0.7308775	2.3430064	-1.1626756
C	-1.0039261	-2.4192948	-2.0538843
H	-1.8198881	-2.3097059	-2.7711921
H	-0.0567468	-2.1988390	-2.5494399

H	-0.9852332	-3.4515330	-1.7012900
H	-0.3608558	-1.5855974	-0.1864600
H	1.1781949	-4.5708346	-0.1268501
C	5.4312044	-1.9759165	1.8340740
C	5.6769475	-3.4283486	1.4340925
H	4.5864704	-1.9231281	2.5231947
H	6.3036153	-1.6007571	2.3780902
H	5.8557847	-4.0435908	2.3187974
H	4.8182859	-3.8340650	0.8963130
H	6.5499733	-3.5118248	0.7823310

TS_1c_1_tpssh_TZVP

Energy= -1665.8601916343237

C	3.9269940	-0.5356615	0.3754199
C	6.0543656	-0.0147203	-1.3240439
C	3.7309652	0.2828658	-0.7430212
C	5.1787014	-1.0799364	0.6806356
C	6.2318734	-0.8169755	-0.2023758
C	4.8094641	0.5485878	-1.5864052
H	7.2109750	-1.2337176	0.0107709
H	4.6628998	1.1944704	-2.4442150
H	6.8894146	0.1836710	-1.9866785
N	2.8408016	-0.7111656	1.3028913
C	2.5419725	0.2020558	2.2719476
H	3.1261253	1.1041001	2.3626854
C	1.9576180	-1.7754363	1.4071218

C	1.4680276	-0.2636296	3.0128138
H	1.0124610	0.2451296	3.8481918
C	1.0985375	-1.5056676	2.4682036
H	0.2945001	-2.1477258	2.7908069
C	1.9700553	-2.9025038	0.4989830
C	2.3872847	0.8811841	-1.0700402
O	2.7422600	-3.0815672	-0.4289189
O	2.3378707	2.1077682	-1.3605013
O	0.9812310	-3.7905980	0.8006089
O	1.3901176	0.0910547	-1.0555912
N	-0.2926413	2.1084630	-1.0158301
C	-0.5422962	0.8528739	-1.1486497
N	-1.2833837	-0.0793797	-1.3014761
C	-1.2383202	3.0528905	-0.3404725
C	-1.2403286	-1.4417483	-0.7531954
C	-2.0555454	2.3515759	0.7235595
C	-1.4423843	1.5386865	1.6809965
C	-3.4373179	2.5356970	0.7705370
C	-2.2032427	0.9085870	2.6596207
H	-0.3708706	1.3704054	1.6465911
C	-4.1979291	1.9187781	1.7620090
H	-3.9218623	3.1553930	0.0226099
C	-3.5831627	1.1005815	2.7056938
H	-1.7205252	0.2585975	3.3804106
H	-5.2727081	2.0612235	1.7826433
H	-4.1769891	0.5987654	3.4609869

C	-2.5224526	-1.7356511	0.0006436
C	-3.7328423	-1.1235399	-0.3221771
C	-2.4868898	-2.6777506	1.0320545
C	-4.8930544	-1.4472546	0.3774553
H	-3.7630257	-0.3741939	-1.1048792
C	-3.6466469	-3.0031605	1.7299329
H	-1.5456780	-3.1526721	1.2893033
C	-4.8548210	-2.3873637	1.4043935
H	-5.8253195	-0.9523473	0.1282144
H	-3.6051529	-3.7308552	2.5333809
H	-5.7570493	-2.6321459	1.9542119
C	-0.3994033	4.1990285	0.2251198
H	0.2786181	3.8310778	0.9983868
H	0.1847537	4.6773811	-0.5645427
H	-1.0630528	4.9430343	0.6673223
H	-1.9104116	3.4378054	-1.1107378
H	0.7022674	2.3815154	-1.2218903
C	-1.0033302	-2.4255296	-1.9019607
H	-1.8081916	-2.3559242	-2.6367848
H	-0.0490028	-2.2116923	-2.3868857
H	-0.9786166	-3.4413333	-1.5033058
H	-0.4013623	-1.4914188	-0.0580936
H	1.0483604	-4.5281466	0.1700471
C	5.4186612	-1.9336568	1.9035842
C	5.6354605	-3.4145156	1.5536283
H	4.5793281	-1.8407720	2.5947271

H	6.3044216	-1.5548880	2.4228295
H	5.8100547	-3.9964825	2.4621508
H	4.7626640	-3.8210572	1.0397775
H	6.5018816	-3.5362804	0.8986194

TS_1c_1_blyp_TZVP

Energy= -1665.1931824243215

C	3.9928563	-0.4889588	0.4175557
C	6.1124688	-0.1127444	-1.3575713
C	3.7937498	0.2753489	-0.7493630
C	5.2470043	-1.0475003	0.7328623
C	6.2953780	-0.8563604	-0.1870191
C	4.8685422	0.4668244	-1.6313640
H	7.2734681	-1.2850265	0.0326237
H	4.7215674	1.0686404	-2.5267097
H	6.9422517	0.0280598	-2.0494066
N	2.9066702	-0.6074574	1.3694722
C	2.6397048	0.3458734	2.3248590
H	3.2418884	1.2442416	2.3802952
C	1.9993826	-1.6629287	1.5240057
C	1.5660950	-0.0843082	3.1043642
H	1.1288048	0.4610338	3.9324046
C	1.1616825	-1.3410619	2.6016797
H	0.3457504	-1.9569915	2.9580394
C	1.9720562	-2.8199422	0.6444397
C	2.4528592	0.9024353	-1.0821620

O	2.7221775	-3.0390827	-0.3054263
O	2.4140242	2.1317475	-1.3798930
O	0.9631464	-3.6969969	0.9989163
O	1.4381323	0.1043370	-1.0559379
N	-0.2901833	2.1316483	-0.9792632
C	-0.4678692	0.8500117	-1.1162267
N	-1.2463575	-0.0763728	-1.2145130
C	-1.2807360	3.0214410	-0.2599582
C	-1.1794296	-1.4315396	-0.6235153
C	-2.0445086	2.2549028	0.8136483
C	-1.3651095	1.4788713	1.7701984
C	-3.4454846	2.3208259	0.8604626
C	-2.0761954	0.7648421	2.7397370
H	-0.2797847	1.3942408	1.7352479
C	-4.1580542	1.6214289	1.8449768
H	-3.9840449	2.9055033	0.1141193
C	-3.4758986	0.8370188	2.7828534
H	-1.5386730	0.1395836	3.4507138
H	-5.2463061	1.6666584	1.8588921
H	-4.0306636	0.2635347	3.5236375
C	-2.4764071	-1.7465553	0.1180457
C	-3.7170904	-1.2341097	-0.2901411
C	-2.4242255	-2.6097239	1.2268650
C	-4.8877881	-1.5758286	0.3999359
H	-3.7646964	-0.5450918	-1.1315839
C	-3.5940134	-2.9534274	1.9163021

H	-1.4634591	-3.0113324	1.5479902
C	-4.8312276	-2.4357020	1.5049372
H	-5.8423080	-1.1560516	0.0833148
H	-3.5381429	-3.6166720	2.7794579
H	-5.7406450	-2.6924652	2.0476091
C	-0.4952295	4.2200799	0.3036555
H	0.2299377	3.8900812	1.0582732
H	0.0376016	4.7508344	-0.4964375
H	-1.1956211	4.9185917	0.7760240
H	-1.9916246	3.3780307	-1.0166771
H	0.6697074	2.4729305	-1.2044974
C	-0.8740198	-2.4542025	-1.7376060
H	-1.6588640	-2.4352538	-2.5048290
H	0.0941511	-2.2266475	-2.1995530
H	-0.8321805	-3.4601671	-1.3032131
H	-0.3559083	-1.4374556	0.0976857
H	1.0033129	-4.4530717	0.3756560
C	5.4920939	-1.8519096	1.9991768
C	5.5722187	-3.3766459	1.7351006
H	4.7049157	-1.6513419	2.7352664
H	6.4379579	-1.5163671	2.4486064
H	5.7510323	-3.9176553	2.6741317
H	4.6407438	-3.7455843	1.2902661
H	6.3925251	-3.6117547	1.0434877

4c_A_pbe_def2TZVP

Energy= -1663.79948945878

C	3.1461015	-0.3882925	0.1085215
C	4.9127198	1.2072004	1.5619002
C	2.6475617	0.6553111	0.9119230
C	4.5242166	-0.6376272	0.0087443
C	5.3908822	0.1730311	0.7595360
C	3.5424415	1.4584405	1.6289530
H	6.4665121	-0.0082413	0.6950757
H	3.1556463	2.2697137	2.2463253
H	5.6088812	1.8275165	2.1285929
N	2.2385771	-1.1301375	-0.7238009
C	1.9087001	-0.7297518	-1.9905686
H	2.3388756	0.1775456	-2.4041724
C	1.5618274	-2.3101529	-0.4378413
C	1.0216223	-1.6501938	-2.5377995
H	0.5898566	-1.5992175	-3.5333069
C	0.8018398	-2.6422976	-1.5613183
H	0.1550293	-3.5111149	-1.6379192
C	1.6409781	-2.9705425	0.8526851
C	1.1754264	0.8713703	1.0626175
O	2.3719503	-2.6530611	1.7863932
O	0.8318535	2.1666324	1.1080128
O	0.7853591	-4.0307162	0.9269244
O	0.3683784	-0.0442262	1.1644772
N	-1.8940879	2.2438058	1.1572284
C	-2.4548857	1.3355572	1.7848740

N	-2.9309817	0.5040802	2.5373786
C	-2.5676221	3.1488979	0.1972363
C	-3.2042360	-0.9317305	2.2754839
C	-1.7543171	3.2199330	-1.0853605
C	-1.5573905	4.4520828	-1.7218884
C	-1.2341083	2.0599897	-1.6754248
C	-0.8596823	4.5252428	-2.9315646
H	-1.9502610	5.3641241	-1.2646477
C	-0.5368677	2.1304651	-2.8827628
H	-1.3669984	1.0957608	-1.1819994
C	-0.3479152	3.3631281	-3.5166192
H	-0.7100878	5.4933100	-3.4136898
H	-0.1367702	1.2177001	-3.3282885
H	0.2004584	3.4179553	-4.4589353
C	-3.4980637	-1.1832509	0.8067276
C	-2.4724239	-1.2761988	-0.1465388
C	-4.8300690	-1.2734008	0.3786363
C	-2.7793807	-1.4499458	-1.4984013
H	-1.4281679	-1.1992426	0.1596778
C	-5.1376838	-1.4486205	-0.9735450
H	-5.6353322	-1.2007584	1.1142745
C	-4.1104939	-1.5347774	-1.9178459
H	-1.9688294	-1.5165959	-2.2267452
H	-6.1806030	-1.5166679	-1.2890987
H	-4.3459982	-1.6685033	-2.9754423
C	-4.0164518	2.7381301	-0.0793466

H -4.0515339 1.7339424 -0.5287758

H -4.6062420 2.7317100 0.8486085

H -4.4786043 3.4478837 -0.7787718

H -2.5591374 4.1489898 0.6605516

H -0.1891249 2.2098480 1.1778920

C -2.0678908 -1.7796474 2.8520759

H -1.9460383 -1.5678913 3.9233554

H -2.3094732 -2.8448705 2.7311952

H -1.1186975 -1.5684087 2.3437410

H -4.1199998 -1.1487672 2.8459063

H 0.9155353 -4.4338002 1.8106270

C 5.0884832 -1.7487730 -0.8427285

C 5.4660142 -2.9867580 -0.0127392

H 5.9846046 -1.3728117 -1.3610129

H 4.3677765 -2.0348352 -1.6213078

H 4.5897726 -3.3853630 0.5169224

H 5.8708180 -3.7755623 -0.6628612

H 6.2292264 -2.7384523 0.7392535

4c_A_b3lyp_TZVP

Energy= -1664.8231473472144

C 3.1218633 -0.3539179 0.0798031

C 4.8951755 1.2177243 1.5307112

C 2.6337975 0.6884725 0.8820447

C 4.4910652 -0.6156976 -0.0156360

C 5.3629015 0.1828831 0.7323977

C	3.5318431	1.4790994	1.5982906
H	6.4283520	-0.0092176	0.6729431
H	3.1571607	2.2867851	2.2125680
H	5.5912249	1.8267661	2.0942317
N	2.2126631	-1.1005871	-0.7448451
C	1.8972621	-0.7293394	-2.0202196
H	2.3483868	0.1461851	-2.4569317
C	1.5054506	-2.2520024	-0.4307438
C	0.9930269	-1.6361873	-2.5412972
H	0.5659711	-1.6026036	-3.5307997
C	0.7445472	-2.5931479	-1.5391639
H	0.0782158	-3.4382021	-1.5926336
C	1.5472321	-2.8687018	0.8794881
C	1.1662924	0.9209540	1.0320509
O	2.2733938	-2.5425363	1.8011306
O	0.8454463	2.2099640	1.1814051
O	0.6647923	-3.8934706	0.9834489
O	0.3404608	0.0308554	1.0460242
N	-1.9120782	2.2441711	1.2108187
C	-2.4409071	1.3255229	1.8302572
N	-2.9135561	0.4755909	2.5493708
C	-2.5985155	3.0899347	0.2040669
C	-3.1183162	-0.9701890	2.2556343
C	-1.7457835	3.1655359	-1.0505059
C	-1.5979184	4.3811802	-1.7194046
C	-1.1448319	2.0225229	-1.5817998

C	-0.8681297	4.4551821	-2.9045847
H	-2.0534912	5.2773108	-1.3117032
C	-0.4174034	2.0937585	-2.7652198
H	-1.2361615	1.0767870	-1.0642294
C	-0.2766675	3.3097245	-3.4321268
H	-0.7582777	5.4070157	-3.4109187
H	0.0425661	1.1978770	-3.1632666
H	0.2930504	3.3643591	-4.3522723
C	-3.4297002	-1.1909553	0.7853948
C	-2.4220552	-1.2848466	-0.1781563
C	-4.7636853	-1.2408788	0.3731153
C	-2.7473718	-1.4164328	-1.5254544
H	-1.3816884	-1.2362420	0.1109160
C	-5.0900015	-1.3735485	-0.9741052
H	-5.5542718	-1.1661548	1.1118886
C	-4.0796172	-1.4593440	-1.9292275
H	-1.9531414	-1.4806324	-2.2589639
H	-6.1300839	-1.4082840	-1.2762613
H	-4.3286098	-1.5590920	-2.9791413
C	-4.0136201	2.5971471	-0.1121232
H	-3.9821866	1.5888516	-0.5307324
H	-4.6326651	2.5848022	0.7872613
H	-4.4794789	3.2612425	-0.8413608
H	-2.6583252	4.0902933	0.6379933
H	-0.1539130	2.2742267	1.2473852
C	-1.9332745	-1.7735504	2.7919916

H	-1.8187113	-1.5968769	3.8628214
H	-2.1139507	-2.8381232	2.6324538
H	-1.0082822	-1.4918916	2.2926091
H	-4.0034278	-1.2417218	2.8312996
H	0.7558762	-4.2786864	1.8700489
C	5.0437812	-1.7451914	-0.8521112
C	5.3067858	-3.0093034	-0.0175075
H	5.9792678	-1.4151711	-1.3105975
H	4.3595757	-1.9849347	-1.6662897
H	4.3883285	-3.3621044	0.4534822
H	5.7032581	-3.8069654	-0.6494954
H	6.0325594	-2.8079013	0.7737424

4c_A_pbe0_TZVP

Energy= -1663.8408302501894

C	3.1202539	-0.3828581	0.0988963
C	4.8879672	1.1841858	1.5457215
C	2.6343934	0.6536927	0.9012216
C	4.4857062	-0.6414342	-0.0010012
C	5.3546971	0.1537866	0.7470760
C	3.5273687	1.4438857	1.6152716
H	6.4216144	-0.0365268	0.6837309
H	3.1503534	2.2520064	2.2303700
H	5.5851636	1.7937430	2.1092385
N	2.2136458	-1.1144472	-0.7270910
C	1.8870104	-0.7196732	-1.9854233

H	2.3188147	0.1774744	-2.4017078
C	1.5398413	-2.2818310	-0.4391055
C	1.0030147	-1.6312031	-2.5249406
H	0.5725330	-1.5835024	-3.5137539
C	0.7818851	-2.6150159	-1.5475836
H	0.1362032	-3.4766375	-1.6179271
C	1.6138077	-2.9351212	0.8507828
C	1.1698764	0.8785331	1.0510602
O	2.3359179	-2.6116264	1.7710284
O	0.8396968	2.1589479	1.1284615
O	0.7746405	-3.9824493	0.9285092
O	0.3627994	-0.0220055	1.1252171
N	-1.8850528	2.2371230	1.1658566
C	-2.4374936	1.3391370	1.7928699
N	-2.9215602	0.5116297	2.5249720
C	-2.5640254	3.1173095	0.2031516
C	-3.1821990	-0.9138829	2.2581367
C	-1.7464473	3.1915483	-1.0664913
C	-1.5827066	4.4101353	-1.7179976
C	-1.1930110	2.0440894	-1.6284099
C	-0.8843345	4.4820292	-2.9176512
H	-2.0022991	5.3122447	-1.2825948
C	-0.4963272	2.1132142	-2.8263361
H	-1.3020608	1.0919032	-1.1216022
C	-0.3399512	3.3323116	-3.4761262
H	-0.7607347	5.4392112	-3.4127913

H	-0.0708430	1.2108343	-3.2515521
H	0.2074639	3.3864973	-4.4109460
C	-3.4670918	-1.1604102	0.7939495
C	-2.4423430	-1.2419348	-0.1478272
C	-4.7878905	-1.2626022	0.3640975
C	-2.7399821	-1.4133410	-1.4932646
H	-1.4069097	-1.1554191	0.1608212
C	-5.0860666	-1.4357464	-0.9817282
H	-5.5923118	-1.2001420	1.0907904
C	-4.0602244	-1.5091245	-1.9155991
H	-1.9311343	-1.4708151	-2.2134206
H	-6.1200316	-1.5133123	-1.3001383
H	-4.2884061	-1.6411218	-2.9677643
C	-3.9919418	2.6750498	-0.0859535
H	-3.9980505	1.6712901	-0.5203694
H	-4.5918845	2.6667499	0.8271671
H	-4.4557696	3.3619223	-0.7957894
H	-2.5816329	4.1136193	0.6542294
H	-0.1616135	2.2192224	1.1928985
C	-2.0443876	-1.7492765	2.8244833
H	-1.9269002	-1.5483142	3.8911900
H	-2.2700535	-2.8092722	2.6918849
H	-1.1048621	-1.5201279	2.3213810
H	-4.0901595	-1.1421743	2.8200159
H	0.8909073	-4.3905489	1.7987703
C	5.0360809	-1.7538766	-0.8481791

C 5.4183839 -2.9765083 -0.0151169

H 5.9198769 -1.3847041 -1.3764860

H 4.3099017 -2.0425640 -1.6099993

H 4.5517670 -3.3677440 0.5214650

H 5.8164290 -3.7662146 -0.6566503

H 6.1821532 -2.7217257 0.7238619

4c_A_tpssh_TZVP

Energy= -1665.8805637749797

C 3.0703615 0.6073616 0.1267744

C 4.3211271 2.0901185 2.1141838

C 2.3195412 1.0995199 1.2041441

C 4.4415517 0.8546483 0.0198592

C 5.0512633 1.5928774 1.0413661

C 2.9523928 1.8542120 2.1923459

H 6.1161931 1.7913915 0.9780558

H 2.3713217 2.2414660 3.0201424

H 4.8153321 2.6688747 2.8859826

N 2.3949444 -0.0509267 -0.9581645

C 1.8831144 0.6290787 -2.0268820

H 1.9794135 1.7019609 -2.0792626

C 2.1480380 -1.4051650 -1.1330731

C 1.3072472 -0.2771305 -2.9002792

H 0.8249893 -0.0321192 -3.8341906

C 1.4712842 -1.5559377 -2.3368555

H 1.1316877 -2.4971127 -2.7393103

C	2.5072781	-2.3977769	-0.1432524
C	0.8715612	0.7670999	1.3391399
O	3.1337029	-2.1967601	0.8860016
O	0.1433626	1.7754366	1.8302359
O	2.0642993	-3.6345484	-0.4931936
O	0.4025816	-0.3224478	1.0599980
N	-2.4653317	0.9348689	1.7777269
C	-2.6609119	-0.2444934	2.0759993
N	-2.8049295	-1.3705674	2.5034676
C	-3.4877991	1.7956711	1.1239311
C	-2.6217761	-2.6489139	1.7545178
C	-2.8274027	2.5893162	0.0138140
C	-3.1579833	3.9328448	-0.1722472
C	-1.9317217	1.9804145	-0.8681301
C	-2.6076984	4.6577602	-1.2284223
H	-3.8482307	4.4160233	0.5126436
C	-1.3808986	2.7024262	-1.9228536
H	-1.6602260	0.9410630	-0.7222186
C	-1.7172992	4.0434554	-2.1074972
H	-2.8701469	5.7019255	-1.3602263
H	-0.6869991	2.2177389	-2.5999800
H	-1.2856733	4.6058819	-2.9281946
C	-2.9113987	-2.4655038	0.2771762
C	-1.9769284	-1.8809962	-0.5840480
C	-4.1598420	-2.8373654	-0.2286665
C	-2.2934066	-1.6673155	-1.9234932

H	-1.0088558	-1.5721943	-0.2098525
C	-4.4764515	-2.6242013	-1.5691916
H	-4.8922216	-3.2878819	0.4340082
C	-3.5425815	-2.0357952	-2.4204868
H	-1.5593236	-1.2098006	-2.5768832
H	-5.4506303	-2.9151358	-1.9466738
H	-3.7864661	-1.8651684	-3.4633273
C	-4.6722763	0.9824848	0.5945919
H	-4.3300940	0.2644317	-0.1549862
H	-5.1657014	0.4401704	1.4049748
H	-5.3995942	1.6519134	0.1318585
H	-3.8368501	2.4925239	1.8901039
H	-0.8221230	1.4773425	1.8606511
C	-1.2346547	-3.2116980	2.0643176
H	-1.1161385	-3.3375242	3.1424564
H	-1.1254505	-4.1861104	1.5830392
H	-0.4539062	-2.5432613	1.7008726
H	-3.3739788	-3.3190753	2.1729542
H	2.3587303	-4.2570064	0.1938199
C	5.2714797	0.3254783	-1.1247996
C	6.0520238	-0.9399998	-0.7323912
H	5.9758522	1.1023628	-1.4345407
H	4.6326845	0.1079742	-1.9825315
H	5.3699278	-1.7320806	-0.4190585
H	6.6413224	-1.2992248	-1.5796516
H	6.7337036	-0.7330220	0.0965390

4c_A_pbe0_def2TZVP

Energy= -1663.9104522999935

C	3.1372856	-0.3920005	0.1009687
C	4.8874617	1.1882318	1.5539203
C	2.6418313	0.6392459	0.9023258
C	4.5044061	-0.6391636	0.0056421
C	5.3639607	0.1635515	0.7547744
C	3.5254481	1.4362607	1.6188346
H	6.4321789	-0.0167063	0.6919522
H	3.1391188	2.2412729	2.2317266
H	5.5777531	1.8023173	2.1206349
N	2.2375094	-1.1289149	-0.7246393
C	1.9059186	-0.7303612	-1.9782463
H	2.3338445	0.1696899	-2.3921668
C	1.5658593	-2.2952742	-0.4382069
C	1.0205668	-1.6393265	-2.5187890
H	0.5868847	-1.5863622	-3.5056139
C	0.8043613	-2.6259385	-1.5444038
H	0.1587859	-3.4873184	-1.6150371
C	1.6466412	-2.9549800	0.8481717
C	1.1759496	0.8571316	1.0443217
O	2.3711297	-2.6345108	1.7663676
O	0.8397333	2.1350920	1.1057981
O	0.8108659	-4.0021880	0.9240540
O	0.3740623	-0.0457915	1.1244126

N	-1.8821507	2.2219320	1.1435994
C	-2.4409240	1.3317721	1.7746144
N	-2.9256602	0.5122452	2.5145414
C	-2.5511979	3.1245982	0.1994435
C	-3.2023841	-0.9090164	2.2574785
C	-1.7477232	3.1999770	-1.0784154
C	-1.5650242	4.4245526	-1.7114601
C	-1.2224420	2.0518057	-1.6631950
C	-0.8746717	4.5026246	-2.9141658
H	-1.9628230	5.3269317	-1.2569380
C	-0.5331431	2.1268268	-2.8641851
H	-1.3463071	1.0935915	-1.1713642
C	-0.3575205	3.3524066	-3.4948546
H	-0.7356351	5.4648180	-3.3945503
H	-0.1290802	1.2238748	-3.3078705
H	0.1855138	3.4113321	-4.4314740
C	-3.4928674	-1.1622690	0.7969621
C	-2.4714093	-1.2552201	-0.1458162
C	-4.8141430	-1.2581060	0.3707062
C	-2.7722050	-1.4406096	-1.4877301
H	-1.4353264	-1.1700343	0.1603524
C	-5.1158794	-1.4453466	-0.9715411
H	-5.6159276	-1.1815048	1.0987346
C	-4.0930809	-1.5366065	-1.9057208
H	-1.9655671	-1.5088145	-2.2089324
H	-6.1506213	-1.5195759	-1.2870819

H	-4.3239735	-1.6831662	-2.9550293
C	-3.9913322	2.7181591	-0.0738151
H	-4.0259270	1.7229893	-0.5259494
H	-4.5739637	2.7046206	0.8501656
H	-4.4534133	3.4274561	-0.7622247
H	-2.5406785	4.1161134	0.6619524
H	-0.1617319	2.1888025	1.1678669
C	-2.0739023	-1.7536179	2.8279146
H	-1.9499077	-1.5416386	3.8915200
H	-2.3143829	-2.8118022	2.7090931
H	-1.1321241	-1.5439176	2.3206742
H	-4.1114241	-1.1261629	2.8227140
H	0.9307707	-4.4093699	1.7941758
C	5.0660877	-1.7446429	-0.8412055
C	5.4452257	-2.9681546	-0.0101858
H	5.9528859	-1.3706784	-1.3607624
H	4.3475060	-2.0324355	-1.6104565
H	4.5745777	-3.3660237	0.5142553
H	5.8563410	-3.7528788	-0.6490390
H	6.1974850	-2.7117023	0.7396563

4c_A_blyp_TZVP

Energy= -1665.21218057202

C	3.0491805	0.6666531	0.0597647
C	4.3410293	2.1947909	2.0118947
C	2.3107012	1.1766121	1.1520842

C	4.4254424	0.9225828	-0.0793851
C	5.0551615	1.6853880	0.9237148
C	2.9678939	1.9517307	2.1221785
H	6.1225082	1.8889483	0.8364662
H	2.4025379	2.3491655	2.9619754
H	4.8504351	2.7875826	2.7703411
N	2.3539912	-0.0411887	-0.9946385
C	1.7498399	0.6007236	-2.0514433
H	1.8176068	1.6774985	-2.1417212
C	2.1185223	-1.4172991	-1.1119487
C	1.1293435	-0.3492641	-2.8606211
H	0.5764421	-0.1412172	-3.7692153
C	1.3582641	-1.6141083	-2.2711280
H	1.0038228	-2.5753010	-2.6223693
C	2.5457660	-2.3736766	-0.1054380
C	0.8578661	0.8400979	1.3228483
O	3.2161602	-2.1243650	0.8971734
O	0.1391072	1.8433031	1.8839378
O	2.1086176	-3.6494973	-0.3967141
O	0.3655698	-0.2400559	1.0117595
N	-2.4590014	0.9077750	1.8684560
C	-2.5844631	-0.2943231	2.1381291
N	-2.6637128	-1.4470993	2.5342221
C	-3.4957796	1.7400717	1.1762412
C	-2.4115847	-2.7011107	1.7329524
C	-2.8356848	2.5269209	0.0450831

C	-3.1866066	3.8709482	-0.1673064
C	-1.9262830	1.9121119	-0.8337536
C	-2.6452793	4.5897906	-1.2435326
H	-3.8864295	4.3598658	0.5117157
C	-1.3866516	2.6275118	-1.9096961
H	-1.6322698	0.8775165	-0.6706275
C	-1.7437132	3.9683803	-2.1197926
H	-2.9239984	5.6326481	-1.3934253
H	-0.6864593	2.1369431	-2.5834562
H	-1.3204010	4.5241132	-2.9560620
C	-2.8175829	-2.4950433	0.2744887
C	-1.9430530	-1.9321553	-0.6733656
C	-4.1355962	-2.7994336	-0.1145536
C	-2.3855718	-1.6722815	-1.9778597
H	-0.9248320	-1.6717312	-0.3974732
C	-4.5788217	-2.5384230	-1.4182159
H	-4.8244093	-3.2284852	0.6139165
C	-3.7030983	-1.9692702	-2.3545440
H	-1.6970547	-1.2294426	-2.6958734
H	-5.6050425	-2.7732654	-1.6995712
H	-4.0450340	-1.7583100	-3.3673247
C	-4.6795212	0.8987269	0.6552437
H	-4.3339090	0.1686150	-0.0876983
H	-5.1721661	0.3612469	1.4759241
H	-5.4170613	1.5568733	0.1798368
H	-3.8618805	2.4527400	1.9276312

H -0.8279069 1.5294146 1.9398466

C -0.9657016 -3.1797061 1.9569593

H -0.7909572 -3.3546965 3.0261518

H -0.8085006 -4.1234394 1.4187057

H -0.2410556 -2.4418349 1.6013032

H -3.0904687 -3.4398875 2.1751743

H 2.4460311 -4.2403576 0.3098105

C 5.2458393 0.3756896 -1.2350946

C 6.0679076 -0.8743079 -0.8314179

H 5.9314293 1.1613963 -1.5829954

H 4.5938897 0.1251838 -2.0797604

H 5.4082395 -1.6758807 -0.4782107

H 6.6451160 -1.2451203 -1.6890518

H 6.7717516 -0.6347711 -0.0228806

4c_B_b3lyp_TZVP

Energy= -1664.8287280321842

C 2.9663146 -0.1592600 -0.0208007

C 4.7749864 -0.6224590 -2.0957447

C 2.5617646 0.0070557 -1.3617111

C 4.2766310 -0.5469172 0.2852408

C 5.1596033 -0.7873030 -0.7712674

C 3.4848175 -0.2116199 -2.3887811

H 6.1739098 -1.0941878 -0.5432264

H 3.1707971 -0.0705709 -3.4141631

H 5.4797481 -0.8094314 -2.8960904

N	2.0562087	0.0775467	1.0584623
C	1.4499948	-0.9234358	1.7791940
H	1.7484950	-1.9490733	1.6467983
C	1.4037882	1.2705985	1.3314299
C	0.4510799	-0.3718722	2.5471699
H	-0.1912994	-0.9116635	3.2230804
C	0.4111628	1.0117991	2.2534564
H	-0.2450358	1.7527817	2.6808987
C	1.7569174	2.5373029	0.6766120
C	1.2038746	0.4291468	-1.8023143
O	0.7185492	3.2777759	0.2764813
O	0.2312884	0.1003970	-0.9082809
O	2.9050531	2.9109792	0.5003690
O	0.9599379	0.9742458	-2.8516805
C	-1.0547897	0.6010867	-1.0816550
N	-1.2932348	1.7708514	-0.6304078
N	-1.8808052	-0.2824608	-1.6707319
C	-1.5345693	-1.6874473	-1.9533975
H	-2.8649123	-0.0581993	-1.6487733
C	-2.6613108	2.3115447	-0.7197451
C	-1.2488491	-2.4821020	-0.6885428
C	-0.1593774	-3.3500548	-0.6424532
C	-2.0762007	-2.3731801	0.4294979
C	0.0967391	-4.1061606	0.4990237
H	0.5016496	-3.4276643	-1.4991860
C	-1.8202784	-3.1221149	1.5728986

H	-2.9102909	-1.6818319	0.4221747
C	-0.7347514	-3.9949224	1.6103614
H	0.9519750	-4.7713574	0.5236115
H	-2.4635596	-3.0184963	2.4389586
H	-0.5314208	-4.5740078	2.5033099
H	-2.9712592	2.3133096	-1.7721678
H	-0.0923517	2.6915806	0.0417891
C	-3.6688477	1.4758687	0.0632007
C	-4.9010859	1.1429128	-0.5021055
C	-3.3753612	1.0272959	1.3536050
C	-5.8235151	0.3732004	0.2061194
H	-5.1400043	1.4786792	-1.5058426
C	-4.2934409	0.2607480	2.0626195
H	-2.4122019	1.2559393	1.7909535
C	-5.5213797	-0.0702330	1.4904708
H	-6.7726947	0.1166641	-0.2492809
H	-4.0472617	-0.0889247	3.0582307
H	-6.2330845	-0.6744100	2.0401498
C	4.7815409	-0.6200506	1.7078922
C	5.2078051	0.7628051	2.2277429
H	5.6338090	-1.3020814	1.7400994
H	4.0211380	-1.0347028	2.3685575
H	4.3786837	1.4697747	2.1725330
H	5.5447340	0.6932478	3.2642274
H	6.0271725	1.1647416	1.6269072
C	-2.6424283	3.7605376	-0.2287156

H	-3.6346237	4.2046725	-0.3213370
H	-2.3423708	3.8013908	0.8206471
H	-1.9372969	4.3512988	-0.8165751
C	-2.6816612	-2.2968804	-2.7614617
H	-2.8502169	-1.7312625	-3.6798427
H	-2.4400663	-3.3267832	-3.0230951
H	-3.6041563	-2.3049533	-2.1744331
H	-0.6375999	-1.6878278	-2.5737157

4c_B_pbe0_def2TZVP

Energy= -1663.92572030855

C	2.9504067	-0.1453964	-0.0013311
C	4.7435091	-0.6050731	-2.0804306
C	2.5456754	0.0285043	-1.3343161
C	4.2557729	-0.5409975	0.2966540
C	5.1289305	-0.7813644	-0.7617178
C	3.4586735	-0.1846483	-2.3642051
H	6.1420658	-1.0981127	-0.5366666
H	3.1352218	-0.0349620	-3.3866429
H	5.4450962	-0.7914822	-2.8846667
N	2.0490043	0.0967225	1.0694493
C	1.4750765	-0.8900708	1.8196122
H	1.7864893	-1.9158148	1.7048365
C	1.3924055	1.2789511	1.3219614
C	0.4824550	-0.3371638	2.5884906
H	-0.1410895	-0.8703217	3.2885630

C	0.4205984	1.0347669	2.2635002
H	-0.2348869	1.7809284	2.6859156
C	1.7382821	2.5403186	0.6515203
C	1.1900370	0.4469247	-1.7668666
O	0.7092988	3.2480167	0.2223544
O	0.2235453	0.0811348	-0.8956251
O	2.8825530	2.9227892	0.5040157
O	0.9474675	1.0145344	-2.7975629
C	-1.0456388	0.5844348	-1.0656548
N	-1.2812453	1.7544682	-0.6193914
N	-1.8792128	-0.2847695	-1.6475457
C	-1.5451155	-1.6741681	-1.9450417
H	-2.8591534	-0.0462632	-1.6257694
C	-2.6363923	2.2839678	-0.7292633
C	-1.2529912	-2.4813316	-0.6985838
C	-0.1783513	-3.3616708	-0.6766232
C	-2.0613534	-2.3732643	0.4277332
C	0.0818530	-4.1305977	0.4498007
H	0.4688480	-3.4390556	-1.5451437
C	-1.8021785	-3.1364343	1.5556641
H	-2.8882415	-1.6706111	0.4375409
C	-0.7311505	-4.0206043	1.5694981
H	0.9283539	-4.8084224	0.4559997
H	-2.4340913	-3.0344757	2.4312076
H	-0.5245573	-4.6130900	2.4537541
H	-2.9416358	2.2678775	-1.7851491

H	-0.1037447	2.6460772	0.0004997
C	-3.6432428	1.4617304	0.0529604
C	-4.8640243	1.1125441	-0.5163379
C	-3.3609482	1.0405528	1.3501698
C	-5.7871801	0.3546596	0.1949609
H	-5.0930779	1.4298878	-1.5296637
C	-4.2805045	0.2874978	2.0631932
H	-2.4020080	1.2846364	1.7924652
C	-5.4970185	-0.0598538	1.4867957
H	-6.7308202	0.0838218	-0.2653474
H	-4.0437734	-0.0402435	3.0694284
H	-6.2119278	-0.6571611	2.0412585
C	4.7627057	-0.6252179	1.7086411
C	5.2011770	0.7428559	2.2271220
H	5.6079997	-1.3173468	1.7322967
H	3.9997230	-1.0364653	2.3704868
H	4.3776235	1.4584041	2.1792084
H	5.5433949	0.6689494	3.2616323
H	6.0204714	1.1407887	1.6229551
C	-2.6348872	3.7313003	-0.2604822
H	-3.6302970	4.1658521	-0.3658282
H	-2.3431061	3.7884759	0.7911524
H	-1.9299968	4.3209304	-0.8503817
C	-2.6976030	-2.2704936	-2.7376754
H	-2.8757916	-1.6992112	-3.6511041
H	-2.4657075	-3.3009100	-3.0073675

H -3.6126916 -2.2766766 -2.1381500

H -0.6525031 -1.6776829 -2.5755138

4c_B_pbe0_TZVP

Energy= -1663.8546711417887

C 2.9526920 -0.1401487 -0.0038318

C 4.7598760 -0.5707430 -2.0770083

C 2.5515668 0.0392478 -1.3384718

C 4.2598192 -0.5268032 0.2990100

C 5.1413186 -0.7524984 -0.7573293

C 3.4719014 -0.1597363 -2.3656476

H 6.1570250 -1.0605353 -0.5303115

H 3.1553208 -0.0046891 -3.3899184

H 5.4674252 -0.7444754 -2.8792828

N 2.0434676 0.0873537 1.0657140

C 1.4590960 -0.9107561 1.7956336

H 1.7725700 -1.9348414 1.6708770

C 1.3820352 1.2677414 1.3255532

C 0.4587604 -0.3664390 2.5614788

H -0.1731627 -0.9083494 3.2477328

C 0.3999339 1.0102508 2.2538360

H -0.2596771 1.7508261 2.6804744

C 1.7332481 2.5357690 0.6701190

C 1.1944715 0.4529488 -1.7711905

O 0.7063133 3.2468796 0.2349712

O 0.2273384 0.0701251 -0.9042014

O	2.8792299	2.9220128	0.5364181
O	0.9502817	1.0280863	-2.7985924
C	-1.0447843	0.5763920	-1.0593016
N	-1.2731077	1.7519874	-0.6220686
N	-1.8889792	-0.2959019	-1.6254291
C	-1.5574370	-1.6880347	-1.9261183
H	-2.8712179	-0.0703590	-1.5556560
C	-2.6273625	2.2902240	-0.7341286
C	-1.2628413	-2.4979237	-0.6815198
C	-0.1791676	-3.3681804	-0.6598337
C	-2.0793475	-2.4050016	0.4413579
C	0.0825749	-4.1424551	0.4636228
H	0.4734811	-3.4328221	-1.5255469
C	-1.8184303	-3.1733166	1.5663872
H	-2.9129508	-1.7098859	0.4512757
C	-0.7383630	-4.0479580	1.5799543
H	0.9354163	-4.8128909	0.4700051
H	-2.4565037	-3.0843998	2.4393119
H	-0.5310957	-4.6448039	2.4615051
H	-2.9277394	2.2745983	-1.7912728
H	-0.1049843	2.6450333	-0.0008018
C	-3.6416132	1.4725852	0.0450559
C	-4.8661754	1.1382510	-0.5276058
C	-3.3623934	1.0400409	1.3401339
C	-5.7955453	0.3816260	0.1784957
H	-5.0934308	1.4654168	-1.5385115

C	-4.2887172	0.2885332	2.0476317
H	-2.4006178	1.2716858	1.7833066
C	-5.5084371	-0.0448875	1.4680107
H	-6.7414909	0.1215431	-0.2838236
H	-4.0551056	-0.0487313	3.0517400
H	-6.2284580	-0.6403589	2.0183792
C	4.7631237	-0.6067407	1.7134209
C	5.2550247	0.7552217	2.2030408
H	5.5806618	-1.3312087	1.7514115
H	3.9837311	-0.9722612	2.3825761
H	4.4579089	1.4992637	2.1389218
H	5.5945261	0.6905866	3.2393586
H	6.0888759	1.1086930	1.5907536
C	-2.6175748	3.7380895	-0.2661037
H	-3.6092497	4.1794845	-0.3789261
H	-2.3326321	3.7939333	0.7875689
H	-1.9048594	4.3223741	-0.8520987
C	-2.7143530	-2.2788314	-2.7177346
H	-2.8932064	-1.7035303	-3.6287022
H	-2.4860752	-3.3090565	-2.9917735
H	-3.6283635	-2.2852729	-2.1160722
H	-0.6669894	-1.6901009	-2.5589805

4c_B_pbe_def2TZVP

Energy= -1663.8090374133167

C	2.9750807	-0.1363760	0.0014753

C	4.8000093	-0.5702142	-2.0820190
C	2.5762624	0.0519384	-1.3436375
C	4.2910936	-0.5284816	0.3086956
C	5.1788375	-0.7577152	-0.7530582
C	3.5083915	-0.1471357	-2.3742252
H	6.1991458	-1.0720218	-0.5208386
H	3.1908866	0.0140247	-3.4046428
H	5.5130116	-0.7492708	-2.8878824
N	2.0579811	0.0935519	1.0743633
C	1.4900032	-0.9097111	1.8300664
H	1.8103650	-1.9389614	1.7067142
C	1.3814830	1.2801668	1.3280558
C	0.4806371	-0.3621074	2.6013468
H	-0.1423975	-0.9063954	3.3044540
C	0.4030436	1.0158928	2.2767599
H	-0.2620384	1.7591400	2.7074920
C	1.7275241	2.5521347	0.6636574
C	1.2203901	0.4783745	-1.7857477
O	0.6887502	3.2571836	0.2084625
O	0.2306198	0.0569424	-0.9270298
O	2.8860809	2.9530201	0.5405625
O	0.9809247	1.0886617	-2.8082040
C	-1.0505523	0.5753027	-1.0861238
N	-1.2763745	1.7645620	-0.6422386
N	-1.9067483	-0.3000563	-1.6484818
C	-1.5923515	-1.7083393	-1.9459157

H	-2.8941717	-0.0547044	-1.5947822
C	-2.6404273	2.3104903	-0.7409599
C	-1.2913145	-2.5194941	-0.6932879
C	-0.1939244	-3.3874660	-0.6722433
C	-2.1089796	-2.4310845	0.4405655
C	0.0791838	-4.1633706	0.4577350
H	0.4606695	-3.4477521	-1.5458398
C	-1.8365390	-3.2007339	1.5726124
H	-2.9537405	-1.7384258	0.4547684
C	-0.7434292	-4.0729274	1.5839871
H	0.9436650	-4.8302030	0.4619205
H	-2.4759745	-3.1131577	2.4534045
H	-0.5266402	-4.6701633	2.4717345
H	-2.9501792	2.2988208	-1.8036356
H	-0.1370818	2.6231122	-0.0414026
C	-3.6562579	1.4849487	0.0432982
C	-4.8840281	1.1340841	-0.5357426
C	-3.3777280	1.0566329	1.3495907
C	-5.8157920	0.3688507	0.1743876
H	-5.1106406	1.4573780	-1.5556885
C	-4.3060008	0.2951422	2.0607682
H	-2.4136028	1.2984136	1.8000916
C	-5.5285279	-0.0527722	1.4749365
H	-6.7638230	0.0975260	-0.2937811
H	-4.0698956	-0.0394861	3.0728131
H	-6.2502097	-0.6562571	2.0283960

C	4.7977513	-0.6087912	1.7288733
C	5.3043641	0.7577384	2.2205080
H	5.6159500	-1.3436537	1.7676545
H	4.0122690	-0.9701190	2.4052999
H	4.5090425	1.5143871	2.1572303
H	5.6462833	0.6891227	3.2629487
H	6.1460137	1.1066365	1.6038501
C	-2.6271259	3.7667079	-0.2673766
H	-3.6271056	4.2082757	-0.3731609
H	-2.3320576	3.8253397	0.7904154
H	-1.9169389	4.3571116	-0.8631842
C	-2.7673062	-2.2929974	-2.7328023
H	-2.9461317	-1.7166030	-3.6514608
H	-2.5495296	-3.3331322	-3.0056860
H	-3.6847510	-2.2880510	-2.1237040
H	-0.6988217	-1.7216075	-2.5878637

4c_B_tpssh_TZVP

Energy= -1665.89017685547

C	2.9566337	-0.1920665	-0.0235469
C	4.7798916	-0.6588525	-2.0832535
C	2.5779361	0.0239197	-1.3634853
C	4.2454875	-0.6389360	0.2938316
C	5.1355079	-0.8812255	-0.7569678
C	3.5093689	-0.1926202	-2.3846518
H	6.1349595	-1.2318532	-0.5216376

H	3.2132335	-0.0106514	-3.4103487
H	5.4927156	-0.8458630	-2.8775722
N	2.0433879	0.0763204	1.0441013
C	1.4154970	-0.8935053	1.7898337
H	1.6971730	-1.9280399	1.6856127
C	1.4074302	1.2886537	1.2727213
C	0.4200200	-0.3001689	2.5337773
H	-0.2347999	-0.8078072	3.2243496
C	0.4048188	1.0735177	2.1976430
H	-0.2348338	1.8412349	2.6046365
C	1.8051845	2.5290262	0.5952879
C	1.2382882	0.4919266	-1.8036274
O	0.7879747	3.2894903	0.1731702
O	0.2422610	0.0893603	-0.9568614
O	2.9683821	2.8690284	0.4215672
O	1.0193898	1.1162824	-2.8166649
C	-1.0344198	0.6205168	-1.1106242
N	-1.2516747	1.7950400	-0.6491758
N	-1.8805199	-0.2428087	-1.7030617
C	-1.5599656	-1.6576385	-1.9802758
C	-2.6248646	2.3337585	-0.7278133
C	-1.3098040	-2.4521777	-0.7093756
C	-0.2313619	-3.3335848	-0.6377061
C	-2.1632893	-2.3306535	0.3883189
C	-0.0117989	-4.0907880	0.5116327
H	0.4482271	-3.4195791	-1.4802604

C	-1.9431948	-3.0803936	1.5395103
H	-2.9905202	-1.6292257	0.3573502
C	-0.8685492	-3.9665411	1.6035595
H	0.8350282	-4.7671095	0.5581072
H	-2.6063618	-2.9670543	2.3904806
H	-0.6930059	-4.5465344	2.5029613
H	-2.9449451	2.3368430	-1.7780534
H	-0.0426399	2.7051852	-0.0350281
C	-3.6148481	1.4874715	0.0636943
C	-4.8284495	1.0948800	-0.5068542
C	-3.3198098	1.0847745	1.3695311
C	-5.7305127	0.3098155	0.2127067
H	-5.0675749	1.3982958	-1.5221424
C	-4.2192543	0.3067469	2.0908668
H	-2.3689680	1.3605523	1.8085615
C	-5.4278847	-0.0851058	1.5137116
H	-6.6643754	0.0043904	-0.2460853
H	-3.9722275	-0.0066498	3.0992404
H	-6.1241822	-0.7000741	2.0727639
C	4.7173444	-0.7719986	1.7230985
H	5.5820830	-1.4396324	1.7409138
H	3.9457019	-1.2306043	2.3429295
C	-2.6022695	3.7780596	-0.2242912
H	-3.5980844	4.2189468	-0.2995019
H	-2.2871843	3.8075181	0.8217959
H	-1.9061013	4.3747576	-0.8180903

C	-2.7173381	-2.2388223	-2.7941748
H	-2.8640087	-1.6697018	-3.7148158
H	-2.4994766	-3.2763443	-3.0493781
H	-3.6425109	-2.2193184	-2.2101963
H	-0.6552435	-1.6751525	-2.5897937
C	5.1029606	0.5915270	2.3197701
H	4.2577956	1.2814463	2.2827394
H	5.4184130	0.4738767	3.3594867
H	5.9261580	1.0383595	1.7566505
H	-2.8643988	-0.0116857	-1.6359341

4c_B_blyp_TZVP

Energy= -1665.2129405361466

C	3.0003267	-0.1741180	-0.0126030
C	4.8706527	-0.6399669	-2.0558939
C	2.6247317	0.0000333	-1.3725253
C	4.3111181	-0.5679509	0.3283971
C	5.2252217	-0.8097879	-0.7137434
C	3.5818689	-0.2202611	-2.3828653
H	6.2390419	-1.1192252	-0.4599258
H	3.2943516	-0.0740428	-3.4216291
H	5.5985331	-0.8286257	-2.8432741
N	2.0598567	0.0683099	1.0532908
C	1.4214130	-0.9380379	1.7618384
H	1.7159142	-1.9704094	1.6313912
C	1.4013974	1.2773111	1.3104318

C	0.3966029	-0.3741345	2.5059185
H	-0.2732459	-0.9140331	3.1637603
C	0.3745793	1.0166619	2.2124908
H	-0.2879299	1.7666689	2.6277127
C	1.7765653	2.5419144	0.6528947
C	1.2752538	0.4397883	-1.8457805
O	0.7355292	3.2930923	0.2186582
O	0.2653581	0.0832811	-0.9688724
O	2.9424341	2.9161867	0.4940564
O	1.0559030	1.0163059	-2.8981238
C	-1.0307698	0.6006458	-1.1646700
N	-1.2690455	1.7804587	-0.7051277
N	-1.8562051	-0.2836145	-1.7804127
C	-1.5360687	-1.7254713	-1.9834354
H	-2.8463718	-0.0508973	-1.7681397
C	-2.6548278	2.3254924	-0.7732043
C	-1.3365489	-2.4660703	-0.6589783
C	-0.2586029	-3.3505541	-0.5020233
C	-2.2330222	-2.2874854	0.4074042
C	-0.0820875	-4.0533448	0.6985163
H	0.4550586	-3.4825569	-1.3164340
C	-2.0538351	-2.9789834	1.6108250
H	-3.0615319	-1.5877105	0.3125858
C	-0.9794626	-3.8686426	1.7593885
H	0.7647367	-4.7302439	0.8099233
H	-2.7481497	-2.8159147	2.4348959

H	-0.8351229	-4.4020844	2.6984110
H	-2.9827014	2.3281148	-1.8263576
H	-0.0814302	2.6893193	-0.0346146
C	-3.6456999	1.4732089	0.0319085
C	-4.8512449	1.0391729	-0.5460972
C	-3.3566517	1.1004734	1.3572658
C	-5.7485198	0.2421435	0.1826540
H	-5.0892378	1.3186912	-1.5734607
C	-4.2503806	0.3096704	2.0878156
H	-2.4140246	1.4054961	1.8050208
C	-5.4496839	-0.1250537	1.5016768
H	-6.6747511	-0.0936388	-0.2826075
H	-4.0053558	0.0187112	3.1086513
H	-6.1407869	-0.7494310	2.0667111
C	4.7854452	-0.6354869	1.7711985
C	5.2204276	0.7583572	2.2916296
H	5.6335076	-1.3308102	1.8304410
H	4.0011081	-1.0355679	2.4227171
H	4.3965454	1.4781583	2.2106685
H	5.5355163	0.6930479	3.3416627
H	6.0623889	1.1459696	1.7016902
C	-2.6295638	3.7850207	-0.2793829
H	-3.6315843	4.2254896	-0.3531781
H	-2.3063219	3.8307991	0.7687868
H	-1.9362341	4.3817984	-0.8859385
C	-2.6656022	-2.3469197	-2.8272065

H	-2.7722351	-1.8197178	-3.7839566
H	-2.4362809	-3.3997212	-3.0273367
H	-3.6224971	-2.3055470	-2.2874453
H	-0.6040337	-1.7742177	-2.5581153

TS_4c_1_pbe_def2TZVP

Energy= -1663.78093191067

C	3.8766381	-0.5598174	0.3961798
C	6.0958227	0.1078050	-1.1535289
C	3.7207331	0.2928841	-0.7108917
C	5.1365760	-1.0724404	0.7563073
C	6.2337344	-0.7346018	-0.0504367
C	4.8451780	0.6360861	-1.4701729
H	7.2167415	-1.1358218	0.2074531
H	4.7230555	1.3128704	-2.3171517
H	6.9656934	0.3576858	-1.7635748
N	2.7306480	-0.9184769	1.1863096
C	2.1386601	-2.1485975	1.1306323
H	2.5428126	-2.9205146	0.4828979
C	1.9909714	-0.1062707	2.0430184
C	1.0256075	-2.1554011	1.9641761
H	0.3687974	-3.0034534	2.1344200
C	0.9278812	-0.8699722	2.5332667
H	0.1869053	-0.5193861	3.2449761
C	2.3714360	1.2561187	2.3749870
C	2.3879100	0.8818790	-1.0990158

O	3.4218517	1.8137847	2.0729421
O	2.3385737	2.1217817	-1.3471522
O	1.3987728	1.8827197	3.1031831
O	1.3928059	0.0741088	-1.1599692
N	-0.2873844	2.0877822	-1.0289600
C	-0.5207542	0.8234531	-1.1756214
N	-1.3062485	-0.0857984	-1.3270456
C	-1.2879441	3.0475660	-0.4724966
C	-1.3821206	-1.4273750	-0.7514318
C	-2.2735754	2.3540699	0.4462019
C	-1.8369258	1.7023493	1.6094858
C	-3.6369510	2.3433337	0.1321726
C	-2.7494477	1.0437002	2.4335409
H	-0.7756328	1.6910515	1.8653266
C	-4.5533084	1.6906793	0.9619425
H	-3.9821455	2.8373461	-0.7794488
C	-4.1107361	1.0353818	2.1122391
H	-2.3966986	0.5245225	3.3270483
H	-5.6117728	1.6771160	0.6964289
H	-4.8206732	0.5043456	2.7483113
C	-2.8070023	-1.7166311	-0.3139672
C	-3.9042373	-1.3517375	-1.1039443
C	-3.0322173	-2.3934738	0.8903565
C	-5.2034888	-1.6641002	-0.6992014
H	-3.7394359	-0.8018745	-2.0328469
C	-4.3309287	-2.7107530	1.2967198

H	-2.1835662	-2.6639879	1.5232245
C	-5.4210579	-2.3464613	0.5019693
H	-6.0508911	-1.3653364	-1.3196688
H	-4.4911908	-3.2318671	2.2426825
H	-6.4373367	-2.5845481	0.8219348
C	-0.5121002	4.1741701	0.2105213
H	0.0550289	3.7955655	1.0724814
H	0.1892969	4.6464606	-0.4922971
H	-1.2155671	4.9390602	0.5636883
H	-1.8435912	3.4614599	-1.3294585
H	0.7127849	2.3816997	-1.2273098
C	-0.8731321	-2.4521779	-1.7725970
H	-1.4875383	-2.4301190	-2.6837174
H	0.1711009	-2.2324769	-2.0322551
H	-0.9280281	-3.4591520	-1.3361586
H	-0.7280344	-1.4584739	0.1327976
H	1.7470984	2.7696202	3.3329304
C	5.3525299	-1.8944667	2.0052059
C	5.5679647	-1.0145662	3.2480597
H	6.2351889	-2.5334805	1.8524094
H	4.5016641	-2.5671744	2.1813444
H	5.7306812	-1.6387608	4.1384086
H	6.4443949	-0.3628082	3.1185573
H	4.6970717	-0.3699392	3.4320872

TS_4c_1_b3lyp_TZVP

Energy= -1664.7986120994315

C	3.8585426	-0.5255776	0.4228011
C	6.0678980	-0.0265165	-1.1851562
C	3.7152337	0.2521183	-0.7316546
C	5.1042897	-1.0427597	0.8029392
C	6.1966953	-0.7914128	-0.0319671
C	4.8328270	0.5106639	-1.5229132
H	7.1658994	-1.1934893	0.2398920
H	4.7211538	1.1295264	-2.4040425
H	6.9306485	0.1584946	-1.8136076
N	2.7169157	-0.7906432	1.2553576
C	2.1140927	-2.0120076	1.3120732
H	2.5056057	-2.8376127	0.7411982
C	1.9829036	0.0982795	2.0403795
C	1.0151435	-1.9396983	2.1471779
H	0.3587927	-2.7574990	2.3955703
C	0.9292587	-0.6111933	2.5991531
H	0.2005328	-0.1945324	3.2739577
C	2.2635306	1.5039586	2.2697997
C	2.4006546	0.8666603	-1.1391754
O	1.4854935	2.2584978	2.8359860
O	2.3910879	2.0621051	-1.5005040
O	3.4624823	1.9183302	1.8167778
O	1.3771091	0.1013305	-1.0950290
N	-0.2662481	2.1028831	-0.9778384
C	-0.4576447	0.8349427	-1.1479189

N	-1.2436435	-0.0623450	-1.2874100
C	-1.2950584	3.0209094	-0.3981512
C	-1.2547856	-1.4368828	-0.7814007
C	-2.2343897	2.2743846	0.5258993
C	-1.7583064	1.6646215	1.6899731
C	-3.5849074	2.1595475	0.2027587
C	-2.6208129	0.9406102	2.5042769
H	-0.7093413	1.7360174	1.9488336
C	-4.4515452	1.4419929	1.0240351
H	-3.9589405	2.6154119	-0.7071902
C	-3.9700843	0.8268473	2.1741174
H	-2.2402470	0.4538197	3.3944053
H	-5.4953738	1.3452760	0.7518769
H	-4.6359908	0.2457677	2.7998862
C	-2.6495419	-1.7882723	-0.3005667
C	-3.7781619	-1.4810361	-1.0618035
C	-2.8128717	-2.4615476	0.9087246
C	-5.0464687	-1.8456024	-0.6229103
H	-3.6642080	-0.9370725	-1.9917487
C	-4.0811512	-2.8320765	1.3496940
H	-1.9457678	-2.6873313	1.5182413
C	-5.2022036	-2.5240900	0.5846612
H	-5.9157713	-1.5918328	-1.2184012
H	-4.1924264	-3.3482159	2.2961465
H	-6.1910721	-2.8025774	0.9295591
C	-0.5471163	4.1666293	0.2824870

H	0.0538929	3.7990442	1.1157999
H	0.1098687	4.6727373	-0.4284660
H	-1.2664591	4.8931754	0.6610213
H	-1.8724607	3.4173390	-1.2358979
H	0.6703840	2.4517386	-1.2300205
C	-0.7599269	-2.3837789	-1.8790971
H	-1.4121269	-2.3339301	-2.7531025
H	0.2551700	-2.1116544	-2.1693161
H	-0.7606561	-3.4078142	-1.5014783
H	-0.5650853	-1.4894920	0.0611813
H	3.5456910	2.8699890	1.9936029
C	5.3131595	-1.7846503	2.1040269
C	5.4120107	-0.8356129	3.3092048
H	6.2343082	-2.3658860	2.0257233
H	4.5072030	-2.4982648	2.2766051
H	5.5835570	-1.4022853	4.2270039
H	6.2370385	-0.1313415	3.1809072
H	4.4945636	-0.2582741	3.4307116

TS_4c_1_pbe0_TZVP

Energy= -1663.81778036884

C	3.8500370	-0.5547878	0.3887773
C	6.0570287	0.1231240	-1.1419065
C	3.6994614	0.3115452	-0.6947995
C	5.0983819	-1.0792493	0.7332784
C	6.1904626	-0.7359527	-0.0622289

C	4.8163084	0.6603709	-1.4451602
H	7.1651490	-1.1448475	0.1843917
H	4.6979097	1.3488316	-2.2738062
H	6.9223038	0.3792140	-1.7432459
N	2.7131295	-0.9148702	1.1751482
C	2.1236451	-2.1343884	1.1156891
H	2.5277732	-2.9035653	0.4759457
C	1.9748741	-0.1084766	2.0204868
C	1.0177534	-2.1426732	1.9403988
H	0.3647651	-2.9853014	2.1087460
C	0.9192967	-0.8618555	2.5060244
H	0.1806968	-0.5106837	3.2095527
C	2.3469659	1.2506357	2.3551677
C	2.3739394	0.9042870	-1.0828866
O	3.3894671	1.7988145	2.0636820
O	2.3384189	2.1198261	-1.3698036
O	1.3819111	1.8697497	3.0613273
O	1.3792921	0.1198820	-1.1147026
N	-0.2840109	2.0951711	-1.0489111
C	-0.5076925	0.8383668	-1.1946608
N	-1.2566970	-0.0785183	-1.3521265
C	-1.3017139	3.0355923	-0.5256060
C	-1.3221255	-1.4188519	-0.7956821
C	-2.2548096	2.3425818	0.4161866
C	-1.7871150	1.7171767	1.5709664
C	-3.6144383	2.3114618	0.1315624

C	-2.6680931	1.0615544	2.4167303
H	-0.7276120	1.7218120	1.8029971
C	-4.4993794	1.6623115	0.9841099
H	-3.9833645	2.7846146	-0.7731838
C	-4.0274038	1.0326904	2.1261344
H	-2.2933379	0.5636630	3.3046277
H	-5.5561726	1.6325802	0.7434255
H	-4.7127827	0.5064441	2.7808059
C	-2.7310926	-1.7089241	-0.3365731
C	-3.8324257	-1.3512030	-1.1083595
C	-2.9353629	-2.3803579	0.8637323
C	-5.1168946	-1.6652225	-0.6881213
H	-3.6825335	-0.8072897	-2.0349095
C	-4.2197511	-2.6999411	1.2850750
H	-2.0835278	-2.6460658	1.4815599
C	-5.3145819	-2.3422950	0.5096156
H	-5.9683311	-1.3730049	-1.2935980
H	-4.3647091	-3.2180386	2.2270561
H	-6.3192096	-2.5825800	0.8406425
C	-0.5606088	4.1942008	0.1209284
H	0.0165154	3.8533613	0.9831737
H	0.1196024	4.6656053	-0.5922791
H	-1.2791719	4.9421708	0.4576907
H	-1.8700548	3.4074607	-1.3827830
H	0.6964167	2.3979816	-1.2434532
C	-0.8385389	-2.4189003	-1.8374002

H	-1.4726693	-2.3857649	-2.7261053
H	0.1910419	-2.1918308	-2.1186400
H	-0.8778992	-3.4261926	-1.4180158
H	-0.6531140	-1.4591861	0.0666012
H	1.7099950	2.7474588	3.3058676
C	5.3060446	-1.9236428	1.9605868
C	5.4296952	-1.0748574	3.2250536
H	6.2169585	-2.5119839	1.8249566
H	4.4877070	-2.6354780	2.0831297
H	5.5845331	-1.7103138	4.1001006
H	6.2736636	-0.3847855	3.1484841
H	4.5279820	-0.4809851	3.3874773

TS_4c_1_pbe0_def2TZVP

Energy= -1663.88758703056

C	3.8454297	-0.5408357	0.3903840
C	6.0490056	0.1322563	-1.1451333
C	3.6923446	0.3158378	-0.6992614
C	5.0951523	-1.0571059	0.7392251
C	6.1850451	-0.7172340	-0.0592126
C	4.8070377	0.6627058	-1.4519019
H	7.1605987	-1.1214813	0.1905731
H	4.6849954	1.3436196	-2.2858856
H	6.9129917	0.3854407	-1.7490311
N	2.7098899	-0.8973651	1.1776020
C	2.1229280	-2.1161677	1.1245287

H	2.5246123	-2.8859270	0.4842267
C	1.9777953	-0.0922162	2.0256473
C	1.0206216	-2.1251185	1.9535045
H	0.3686333	-2.9674495	2.1246227
C	0.9247398	-0.8443321	2.5172770
H	0.1904169	-0.4923754	3.2244337
C	2.3501434	1.2682075	2.3562628
C	2.3643765	0.8980817	-1.0926601
O	3.3840741	1.8207998	2.0475889
O	2.3245161	2.1141867	-1.3753071
O	1.3971343	1.8806044	3.0798669
O	1.3788974	0.1069134	-1.1321010
N	-0.2864911	2.0773333	-1.0528153
C	-0.5211079	0.8257559	-1.2049988
N	-1.2638310	-0.0940054	-1.3671771
C	-1.2888288	3.0200143	-0.5116242
C	-1.3285911	-1.4315974	-0.8087980
C	-2.2534824	2.3232910	0.4140325
C	-1.7947185	1.6532610	1.5458680
C	-3.6141274	2.3303993	0.1371377
C	-2.6845677	0.9942076	2.3779681
H	-0.7340026	1.6276352	1.7699083
C	-4.5082968	1.6771120	0.9754456
H	-3.9768627	2.8385764	-0.7506563
C	-4.0448025	1.0039332	2.0949393
H	-2.3160437	0.4606649	3.2471162

H	-5.5665170	1.6784234	0.7405213
H	-4.7380097	0.4742253	2.7379189
C	-2.7360594	-1.7234214	-0.3481603
C	-3.8402292	-1.3464593	-1.1047925
C	-2.9357798	-2.4157869	0.8400993
C	-5.1226797	-1.6620951	-0.6819707
H	-3.6947232	-0.7869033	-2.0223520
C	-4.2178564	-2.7362555	1.2644796
H	-2.0809574	-2.6971498	1.4463783
C	-5.3156175	-2.3598957	0.5034208
H	-5.9762490	-1.3547417	-1.2762496
H	-4.3587989	-3.2703979	2.1976976
H	-6.3188770	-2.6007626	0.8370525
C	-0.5324680	4.1529257	0.1610850
H	0.0360167	3.7853432	1.0178811
H	0.1579996	4.6271217	-0.5400897
H	-1.2401961	4.9052251	0.5103851
H	-1.8504064	3.4195235	-1.3610564
H	0.7000984	2.3682367	-1.2466622
C	-0.8459308	-2.4340308	-1.8479674
H	-1.4818388	-2.4042049	-2.7353175
H	0.1824671	-2.2059797	-2.1322934
H	-0.8829688	-3.4397895	-1.4253450
H	-0.6590818	-1.4706137	0.0534512
H	1.7250277	2.7618214	3.3114760
C	5.3060737	-1.8926516	1.9707067

C	5.4412993	-1.0365678	3.2278887
H	6.2134714	-2.4860182	1.8338988
H	4.4855757	-2.6002296	2.1031353
H	5.6007740	-1.6664088	4.1058927
H	6.2864805	-0.3495442	3.1402145
H	4.5429603	-0.4385258	3.3932973

TS_4c_1_tpssh_TZVP

Energy= -1665.8595016389581

C	3.8563479	-0.5629007	0.3833419
C	6.0662072	0.1252103	-1.1510426
C	3.6998430	0.3096264	-0.7003765
C	5.1087258	-1.0888953	0.7272421
C	6.2030992	-0.7401393	-0.0706591
C	4.8205651	0.6631316	-1.4526495
H	7.1786124	-1.1476583	0.1739324
H	4.6999392	1.3529851	-2.2794525
H	6.9303215	0.3844212	-1.7524035
N	2.7159981	-0.9287469	1.1785552
C	2.1292316	-2.1582886	1.1272651
H	2.5362962	-2.9261639	0.4890802
C	1.9698218	-0.1172848	2.0269681
C	1.0227068	-2.1662176	1.9587064
H	0.3735492	-3.0104883	2.1316706
C	0.9176177	-0.8807439	2.5197495
H	0.1782793	-0.5316684	3.2228759

C	2.3389450	1.2453719	2.3479843
C	2.3712745	0.9051017	-1.0858597
O	3.3765320	1.8098282	2.0365492
O	2.3381112	2.1209979	-1.4027858
O	1.3685660	1.8638347	3.0760503
O	1.3646545	0.1172930	-1.0879207
N	-0.2985693	2.1122066	-1.0644154
C	-0.5160868	0.8468681	-1.1944045
N	-1.2885815	-0.0640164	-1.3459015
C	-1.3226239	3.0656780	-0.5305571
C	-1.3314616	-1.4080794	-0.7564545
C	-2.2854922	2.3621482	0.4010175
C	-1.8263667	1.7314579	1.5618378
C	-3.6461004	2.3275703	0.0992307
C	-2.7175721	1.0670352	2.3971006
H	-0.7697383	1.7382914	1.8039299
C	-4.5408285	1.6700929	0.9419342
H	-4.0057819	2.8028145	-0.8078582
C	-4.0776874	1.0352541	2.0901395
H	-2.3511811	0.5651719	3.2859460
H	-5.5944142	1.6365645	0.6887904
H	-4.7680353	0.5024260	2.7338021
C	-2.7453032	-1.7081099	-0.3001328
C	-3.8505785	-1.3570676	-1.0771925
C	-2.9480192	-2.3832062	0.9038261
C	-5.1378843	-1.6809603	-0.6584289

H	-3.7019744	-0.8107678	-2.0021137
C	-4.2351973	-2.7126101	1.3233538
H	-2.0961570	-2.6429761	1.5234979
C	-5.3343097	-2.3615136	0.5426243
H	-5.9898061	-1.3936617	-1.2653767
H	-4.3787862	-3.2310571	2.2652361
H	-6.3379399	-2.6081174	0.8716152
C	-0.5644107	4.2132283	0.1352164
H	0.0028553	3.8538211	0.9961638
H	0.1246825	4.6812344	-0.5718862
H	-1.2777154	4.9653194	0.4745519
H	-1.8783768	3.4481125	-1.3899057
H	0.6810199	2.4166094	-1.2652704
C	-0.8266842	-2.4224946	-1.7872311
H	-1.4576228	-2.4082962	-2.6785770
H	0.2016997	-2.1856494	-2.0642495
H	-0.8577999	-3.4229851	-1.3504259
H	-0.6649049	-1.4198210	0.1069597
H	1.6953136	2.7504992	3.3074202
C	5.3170911	-1.9401049	1.9584421
C	5.4076471	-1.0886755	3.2352382
H	6.2415945	-2.5091173	1.8331192
H	4.5080553	-2.6646505	2.0680488
H	5.5608269	-1.7280202	4.1082030
H	6.2407901	-0.3842199	3.1711100
H	4.4912002	-0.5140264	3.3838448

TS_4c_1_blyp_TZVP

Energy= -1665.1915093399336

C	3.8546578	-0.6188107	0.4174095
C	6.1039797	-0.0355907	-1.1399752
C	3.7232292	0.2152797	-0.7132674
C	5.1079330	-1.1413951	0.8034407
C	6.2208526	-0.8452901	-0.0052492
C	4.8629715	0.5094799	-1.4795787
H	7.1944511	-1.2490532	0.2725239
H	4.7625200	1.1657223	-2.3419360
H	6.9799304	0.1796221	-1.7507399
N	2.6931982	-0.9537985	1.2156294
C	2.0938620	-2.1903481	1.1842960
H	2.4966208	-2.9755408	0.5569584
C	1.9499694	-0.1204221	2.0675013
C	0.9840133	-2.1795309	2.0267425
H	0.3266478	-3.0204122	2.2128404
C	0.8879946	-0.8803820	2.5754903
H	0.1453746	-0.5175221	3.2747268
C	2.3162299	1.2570693	2.3501093
C	2.4165407	0.8705794	-1.1128488
O	3.3439253	1.8305859	1.9879373
O	2.4297733	2.0684614	-1.4934856
O	1.3496883	1.8931748	3.1067798
O	1.3577455	0.1137329	-1.0381216

N	-0.2581943	2.1549709	-0.9471214
C	-0.4081920	0.8653408	-1.1275451
N	-1.2641909	0.0026765	-1.2599810
C	-1.3331836	3.0545403	-0.3881027
C	-1.2999766	-1.3747380	-0.7268061
C	-2.2903888	2.2849840	0.5134901
C	-1.8247706	1.6194779	1.6628341
C	-3.6540668	2.2161952	0.1912119
C	-2.7086307	0.8869185	2.4614247
H	-0.7674862	1.6468599	1.9183311
C	-4.5423068	1.4913413	0.9982184
H	-4.0210740	2.7123284	-0.7077159
C	-4.0709144	0.8219509	2.1331539
H	-2.3340777	0.3532338	3.3343136
H	-5.5943588	1.4279014	0.7238929
H	-4.7525390	0.2299416	2.7414518
C	-2.7195776	-1.7041759	-0.2713976
C	-3.8351621	-1.3750183	-1.0589469
C	-2.9217478	-2.3752624	0.9449230
C	-5.1276679	-1.7141262	-0.6406540
H	-3.6911763	-0.8304613	-1.9911006
C	-4.2139771	-2.7201506	1.3656087
H	-2.0674086	-2.6138564	1.5775956
C	-5.3217419	-2.3891240	0.5737763
H	-5.9847010	-1.4401470	-1.2557779
H	-4.3550492	-3.2301285	2.3184326

H	-6.3282187	-2.6449583	0.9041160
C	-0.6275589	4.2226486	0.3266588
H	-0.0417285	3.8613838	1.1813666
H	0.0426346	4.7548034	-0.3617389
H	-1.3795381	4.9315425	0.6924457
H	-1.8971010	3.4470542	-1.2451846
H	0.6693010	2.5380670	-1.1948013
C	-0.7813595	-2.3643085	-1.7928371
H	-1.4013972	-2.3187424	-2.6974473
H	0.2561409	-2.1202187	-2.0497880
H	-0.8198990	-3.3852594	-1.3917373
H	-0.6316132	-1.4210034	0.1402158
H	1.6785352	2.7968598	3.3001973
C	5.3034410	-1.9229922	2.0934619
C	5.4359723	-0.9900455	3.3237997
H	6.2128207	-2.5316098	1.9998776
H	4.4735850	-2.6196311	2.2591617
H	5.5874494	-1.5800384	4.2376913
H	6.2903732	-0.3101002	3.2050701
H	4.5348718	-0.3782345	3.4525224
